# Monosaccharide Binding to Synthetic Carbohydrate Receptor Microarrays

**DOI:** 10.1021/acs.jpcc.5c06186

**Published:** 2025-11-06

**Authors:** Milan A. Shlain, Kenneth Erzoah Ndede, Khushabu Thakur, Anthony J. Russo, Siddharth Pasari, Ishraq Nihal, Keidy L. Matos, Yerzhan S. Zholdassov, Mateusz Marianski, Adam B. Braunschweig

**Affiliations:** † 5924 Advanced Science Research Center at the Graduate Center The City University of New York, 85 St. Nicholas Terrace, New York, New York 1003, United States; ‡ Department of Chemistry, Hunter College, 695 Park Ave, New York, New York 10065, United States; § The PhD Program in Chemistry Graduate Center of the City University of New York, 365 Fifth Ave, New York, New York 10016, United States; ∥ The PhD Program in Biochemistry Graduate Center of the City University of New York, 365 Fifth Ave, New York, New York 10016, United States; ⊥ Hunter College High School, 71 E 94th St, New York, New York 10128, United States

## Abstract

A glycan detection platform, comprised of synthetic carbohydrate receptors (SCRs) immobilized onto polymer brushes, was prepared. **SCR043**, an alkene-containing SCR, was incorporated into grafted-from polymer brushes using hypersurface photolithography, resulting in microarrays of **SCR043**-functionalized polymer brushes, where brush height (*h*) and SCR grafting density (Γ) are controlled precisely at each feature in the array. The influence of *h* and Γ on the binding to five fluorescently labeled monosaccharides—*α*-glucose (**α-Gluc-FL**), α-galactose (**α-Gal-FL**), α-mannose (**α-Man-FL**), β-glucose (**β-Gluc-FL**), and β-galactose (**β-Gal-FL**)—in aqueous buffer was investigated using fluorescence microscopy. These experiments provided 9072 data points, each corresponding to an individual binding experiment, which were used to assess the effects of polymer *h*, Γ, monosaccharide structure, and monosaccharide concentration on binding avidity (*K*_d_). We demonstrate that SCR-based microarrays bind monosaccharides selectively as a result of cooperative, supramolecular interactions that occur within the multivalent polymer brushes. *K*_d_, Hill coefficients, 50% inhibition concentrations, and inhibition constants (*K*_i_) were calculated for the different monosaccharide**-SCR043** binding pairs and were compared with the binding energies calculated using Density Functional Theory. The SCR-functionalized polymer brush microarrays could detect monosaccharides at micromolar concentrations in aqueous buffers, with *K*_i_ as low as 5 μM for **α-Man-FL**. The strength of the monosaccharide–SCR interactions is attributed to the cluster-glycoside effects that can occur within the SCR-functionalized polymer brushes. This report represents the first demonstration that SCRs can function as effective glycan recognition elements in microarray formats.

## Graphical abstract

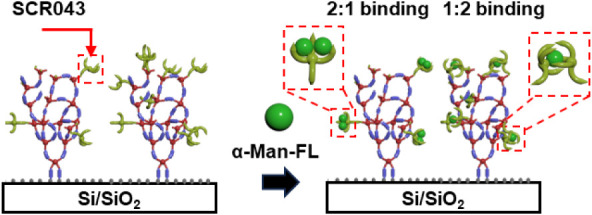

## Introduction

There is substantial interest in creating sensors for detecting the carbohydrate components of glycopeptides, glycoproteins, and glycopolymers—collectively known as glycans—which appear throughout nature,^1^ but challenges related to the sensitivity and selectivity of the detection schemes have hindered their adoption. The low sensitivity and selectivity of glycan-detection platforms arises in part from their inability to access cluster-glycoside effects, where the multivalency and cooperativity of dense arrays of glycan recognition elements that occur on biological interfaces work in tandem to strengthen binding avidities (*K*_d_) between glycans and the recognition element on the surface by as much as 10^6^ M.^2^ As a result, many carbohydrate detection platforms are often unable to bind glycans in biologically relevant media or at biologically relevant concentrations.^3^ In particular, lectin arrays, which involve glycan-binding proteins (GBPs) that have been appended onto a surface,^4–8^ are currently being explored for detecting glycans for cancer biomarker detection,^9^ pathogen profiling,^10^ and characterizing immune response.^11^ The advantage of microarrays over other methods to bind or analyze glycans is their high-throughput character—hundreds of different binding experiments can be carried out on a single chip. To detect binding between the arrays and their glycan targets, the glycans are modified with dyes^12^ that can be detected based on their fluorescence that can be measured by widely available scanners or fluorescence microscopes, although other signaling schemes, such as electrochemical^17^ or flourescent sandwich assays,^13^ also exist. Two limitations precluding the widespread use of glycan-binding arrays in science and biotechnology are that they rely on GBPs as the glycan recognition element, which have the typical availability and stability challenges associated with protein recognition elements, coupled with the weak and unselective binding associated with glycan-lectin associati. In addition, the immobilization chemistries used to adhere the GBPs to the surfaces lead to monolayers of GBPs with limited control over density. As such, these lectin arrays generally do not access cluster glycoside effects, which may result in low sensitivity and alter selectivity compared to what may occur in biointerfaces^14,15^ To increase the sensitivity of glycan-binding microarrays, there is a need for a glycan-binding recognition element where the density in three dimensions can be controlled precisely to exploit and manipulate cluster-glycoside effects. To increase the stability and availability of glycan-binding microarrays, small molecule carbohydrate receptors may offer a solution. Such arrays could be used for detecting the presence of glycans and for measuring and understanding the role of multivalency and cooperativity in glycan-binding. The need for such high-throughput and accurate glycan analysis platforms is particularly pressing now given the increased attention toward exploring and exploiting the roles of glycans in medicine, biotechnology, and materials.^16–18^

In an effort to address both the cluster-glycoside and the stability challenges in glycan microarrays, here we introduce a stable glycan-binding microarray platform that can explore quantitatively the relationships between interface structure and binding to achieve selective detection of monosaccharides. The glycan recognition elements in these arrays are composed of synthetic carbohydrate receptors^19–24^ (SCRs) that are immobilized onto polymer brushes, whose height (*h*) and SCR grafting density (Γ) can be varied precisely as a result of the polymer chemistry and printing methods used to prepare them. The ability of the SCR-modified polymer brush biorecognition element to bind carbohydrates is validated herein by examining the binding of this array to five fluorescein (FL)-labeled monosaccharides, including α-glucose (**α-Gluc-FL**), α-galactose (**α-Gal-FL**), α-mannose (**α-Man-FL**), β-glucose (**β-Gluc-FL**), and β-galactose (**β-Gal-FL**) in aqueous buffer (Figure 1A). Monosaccharides, as opposed to more complex glycans, are chosen for this study to validate that arrays containing SCRs as glycan recognition elements can indeed bind carbohydrates, and because the relative simplicity of the monosaccharides simplifies the analysis required to assess the effects of multivalency and cooperativity on binding. The fluorescence of the arrays was used to determine *K*_d_, inhibition constant (*K*_i_), 50% inhibition concentrations (*IC*_50_), and Hill cooperativity constants (*H*_c_). *K*_d_ values as low as 19 μM and *K*_i_ as low as 5 μM were measured between the **SCR043**-modified polymer brushes and **α-Man-FL**, and we find that **β-Gluc-FL**, **α-Gluc-FL,** and **α-Man-FL** bound the immobilized SCRs with cooperativity, whereas **α-Gal-FL** and **β-Gal-FL** did not bind within the detection limit of the array. Interestingly, we find that cooperativity varies with monosaccharide concentration and Γ because of the different possible stoichiometries (2:1, 1:1, and 1:2) of monosaccharide:SCR binding. These results are supported by Density Functional Theory (DFT) calculations of the supramolecular binding structures, and are consistent with previous studies on the binding between monosaccharides and SCRs carried out in organic solutions^19–22,24^ and in aqueous solution.^25^ These proof-of-concept studies demonstrate that SCR-functionalized brush polymers can bind monosaccharides with high sensitivity and statistically significant selectivity. The modularity of the immobilization chemistry results in the ability to vary the architecture of the biorecognition elements precisely so that the effects of cooperativity and multivalency on binding can be explored quantitatively, and these microarray sensors could serve as the basis for stable, sensitive, and selective sensors for the detection of glycans.

**Figure 1:**
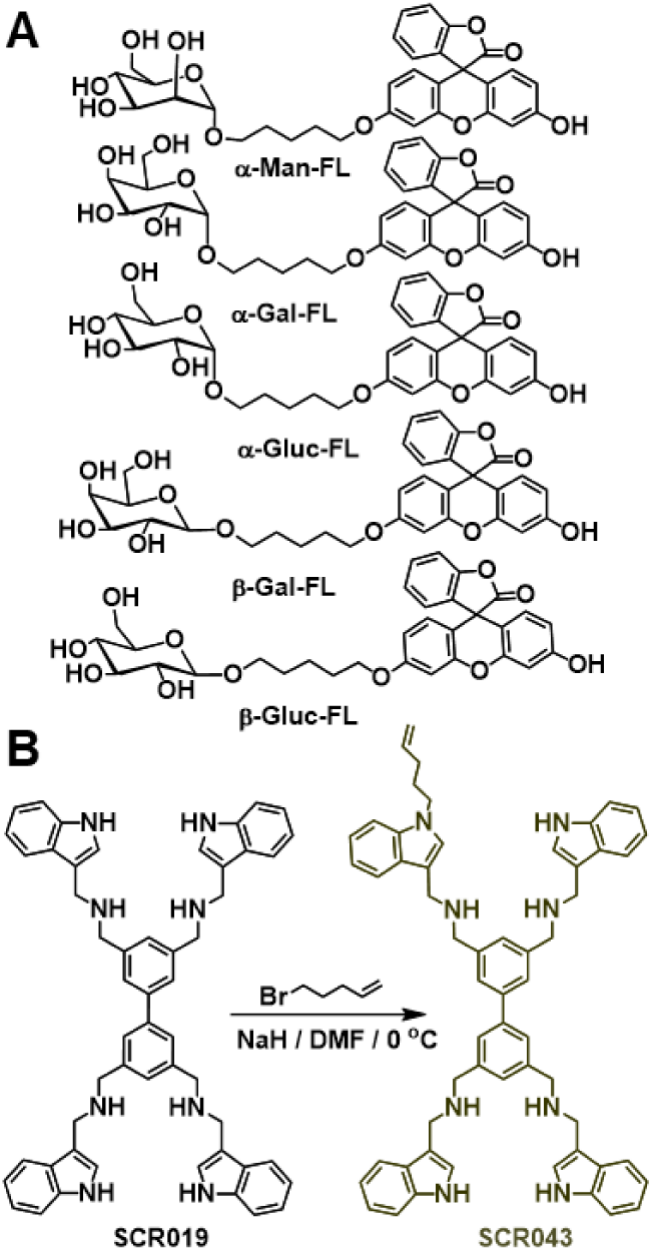
(A) Structures of the five flourescein (FL)-labeled monosaccharides whose binding to **SCR043**-functionalized polymer brushes was studied. (B) Synthesis of **SCR043**.

## Methods

### Synthesis of SCR043

NaH (29 mg, 1.2 mmol) was added to DMF (2 mL) in a Schlenk flask under Ar and cooled to 0 °C. A solution of **SCR019** ^20^ (1.2 g, 1.5 mmol) in DMF (50 mL) was added dropwise and stirred for 15 min. 5-Bromopent-1-ene (0.18 g, 1.2 mmol, 0.15 mL) in DMF (50 mL) was then added, and the reaction was stirred at room temperature for 16 h. The reaction was quenched with H_2_O (10 mL), extracted with CH_2_Cl_2_ (3 × 50 mL), dried over Na_2_SO_4_, and concentrated. Purification by column chromatography (CHCl_3_:MeOH:NH_3_ = 9:1:1) afforded **SCR043** as a pale-yellow solid in 31%. **SCR043** was characterized by 1H NMR spectroscopy, ^13^C NMR spectroscopy, and mass spectrometry, and all data are consistent with the proposed molecular structure ( for details).

### Synthesis of Fluorescein-Labeled Monosaccharides (α-Man-FL, α-Gal-FL, β-Gal-FL, α-Gluc-FL, and β-Gluc-FL)

Fluorescein-labeled monosaccharides were prepared via multistep synthesis from their per-acetylated precursors. Each monosaccharide was reacted with 5-bromopenten-1-ol using BF_3_·Et_2_O at −20 °C to give the 5 (5-bromopentyl) derivatives (compounds **2**, **5**, **8**, **11**, and **14**). Subsequent etherification with fluorescein in DMF (NaH, 0 °C to rt, 16 h) produced intermediates **3**, **6**, **9**, **12**, and **15**. Final deacetylation was achieved by treatment with NaOMe in MeOH at 0 °C for 1 h, followed by neutralization (1 M HCl, pH 6) and purification via SiO_2_ chromatography (H_2_O: MeOH: EtOAc = 1:1:8) to yield the FL-labeled sugars. The compounds were characterized by ^1^H NMR spectroscopy, ^13^C NMR spectroscopy, and mass spectrometry, 2D NMR spectroscopy, UV–vis, and fluorescence spectroscopy, and all data are consistent with the proposed molecular structures ( for details).

### Preparation of Thiol-Terminated Substrates

Si <100> wafers (500 nm oxide layer; NOVA Electronic Materials, STK8414-OX) were sliced, cleaned by immersion in piranha solution (3:1 H_2_SO_4_: H_2_O_2_) for 15 min, rinsed with Milli-Q water, and dried with a stream of air. Clean wafers were immersed in toluene containing (3-mercaptopropyl)­trimethoxysilane (4.5 mL, 0.02 M) at 37 °C for 4 h, rinsed sequentially with toluene, 1:1 toluene/ethanol, and ethanol, and cured at 105 °C for 18 h. The resulting thiol-terminated substrates were stored in MeOH at 4 °C. XPS (Physical Electronics VersaProbe II, Al, 1486.6 eV) confirmed successful modification by the presence of an S 2p peak at 164 eV (see for details).

### Printing of SCR043-Functionalized Polymer Brushes

Polymer brush microarrays were printed using a TERA-Fab Elite hypersurface photolithography instrument (TERA Print, LLC, USA). The system integrates a 405 nm LED (1.16 mW·mm^–2^), a DMD with 1024 × 768 mirrors, and a piezo-controlled *x–y–z* stage (1 μm precision). The photopolymerization mixture contained **SCR043** (0–500 μM), PETT (100 mM), EGDMA (1.3 M, inhibitor removed via alumina), and TPO (1 mM) in DMSO. Reactions were conducted in an Ar environment by drop-casting the solution onto thiol-terminated Si/SiO_2_ substrates, followed by exposure through the DMD at controlled intensity and time (1.27–6.33 mW·mm^– 2^, 9.6–1050 s).

After printing, substrates were washed with EtOH (∼5 mL) and air-dried. Feature heights were measured by profilometry using a Bruker Dektak-XT stylus profiler (12.5 μm tip, 1 mg force, 120 scans, Hills and Valleys setting). Profiles were analyzed using custom Python scripts. Chemical composition was confirmed by XPS, ToF-SIMS, and Raman spectroscopy. XPS revealed N 1s peaks at 399 eV indicative of SCR incorporation. ToF-SIMS (*m*/*z* = 130.02, [C_9_H_8_N]^+^) confirmed indole fragments. Raman spectra exhibited the indole C = C stretch near 1530 cm^–1^. Control polymer brushes printed without **SCR043** showed no N 1s signal, confirming covalent attachment (see for details).

### Binding and Imaging Protocol

Binding studies were performed with **α-Man-FL**, **α-Gluc-FL**, **β-Gluc-FL**, **α-Gal-FL**, and **β-Gal-FL** (10^– 3^–10^– 5.5^ M) in Tris buffer (20 mM, pH 7.4) containing 0.9 mM MnCl_2_, 0.5 mM CaCl_2_, and 0.01% Tween20. **SCR043**-patterned microarrays were incubated in glycan solution for 1 h at room temperature, then rinsed with Tris buffer containing 0.01% Tween-20 and Milli-Q water for 10 min. Fluorescence images were acquired using an Olympus BX60 fluorescence microscope (12 V 100 W halogen bulb) with 540–585 nm barrier and 575 nm filters. Images were analyzed using *ImageJ* to extract average feature intensities (see Supporting Information Section for details).

### Computational Methods

All DFT calculations were performed using the FHI-aims package at the PBE+vdW^TS^ level with dispersion corrections. Conformational sampling of **SCR019**–glycan complexes was carried out using CREST (GFN2-xTB) followed by single-point PBE0+MBD calculations in implicit water (PCM). Δ*E* values were calculated for 1:1, 1:2, and 2:1 complexes to evaluate cooperativity ().

## Results and Discussion

### Design and Synthesis of SCR043 and Fluorescein-Labeled Monosaccharides

SCRs are synthetic, supramolecular receptors that bind carbohydrates noncovalently,^20–24^ and are one type of a broader set of carbohydrate binding synthetic molecules that are commonly referred to as “synthetic lectins”.^19–24,26–29^ Recently SCRs have been shown to bind *N*-glycans on viral envelopes and in doing so, stop the progression of viral disease *in vivo*.^25^ SCRs possess a biaryl core to which four heterocyclic binding groups are linked by secondary amine, imine, or amide bonds, and bind to carbohydrates through H-bonding, π•••π interactions, and van der Waals forces. The selectivity of the SCRs for carbohydrates can be altered by changing the heterocyclic binding groups, linker, or valency. **SCR019** (Figure 1B), for example, binds mannosides strongly but binds other monosaccharides weakly or not at all in solution.^20,21^ **SCR019** has previously been shown to bind to β-mannosides with 1:1, 2:1, and 1:2 stoichiometries, with the 1:2 and 2:1 stoichiometries displaying significant cooperativity and are predicted by computational studies to bind α-glucosides and β-glucosides, but this finding could not be validated experimentally.^20^ However, the binding of **SCR019** to monosaccharides has only been studied in solution, at high concentrations (∼10^–3^ M), and in aprotic organic solvents that do not compete for H-bonds.^20^ Like many synthetic lectins, the binding of SCRs to glycans had not been demonstrated in aqueous solutions because the lack of cluster-glycoside effects renders the binding too weak to detect in the presence of the solvent competition that occurs in water. This challenge was recently overcome when it was shown that SCRs can bind to multivalent glycan-functionalized polymer brushes in aqueous solution.^25^ As such, we anticipated that by reversing this binding system—instead incorporating **SCR019** onto polymer brush arrays and exposing these arrays to glycans in solution—the multivalent and cooperative interactions would still occur and lead to binding of the glycans by the SCR-functionalized polymer brushes. By functionalizing **SCR019** with an alkene, the resulting SCR—**SCR043** (Figure 1B)—could be incorporated into multivalent polymer brushes using Hypersurface Photolithography^30–34^ (HP) in combination with the Grafted-To Grafted-From Radical Photopolymerization (GTGFRP)^32^ to create **SCR043**-functionalized polymer brushes with precise control over the *h* and Γ at each feature.

To this end **SCR043** was prepared (Figure 1B) in a single step from **SCR019** in 31% yield by harnessing the inherent nucleophilicity of the heterocyclic N of **SCR019** in the presence of 1.25 equiv of 5-bromopent-1-ene and NaH. The product was characterized by ^1^H NMR spectroscopy, ^13^C NMR spectroscopy, and high-resolution mass spectrometry, and all characterization data were consistent with the proposed structure of **SCR043** (see for details). So that binding between glycans and **SCR043**-functionalized polymer brushes could be measured using fluorescence microscopy, the five FL-labeled monosaccharides were prepared by multistep organic synthesis (**Scheme S2–S6**). The labeled monosaccharides were characterized by ^1^H NMR spectroscopy, ^13^C NMR spectroscopy, high resolution mass spectrometry, UV–vis spectroscopy, ^1^H–^13^C HSQC, ^1^H–^13^C HMBC, ^1^H–^1^H COSY, ^1^H–^1^H NOESY 2D NMR spectroscopies, and fluorescence spectroscopy (). All data are consistent with the proposed structure, and the optical characteristics of the FL-labeled monosaccharides were similar to those of FL, with λ_max,abs_ ≈ 495 nm and λ_max,em_ ≈ 518.

### Printing and Characterization of SCR043-Functionalized Polymer Brush Microarrays

The GTGFRP,^32^ which is a thiol-initiated radical photopolymerization,^33,35–37^ involves the simultaneous copolymerization of ethylene glycol dimethacrylate (EGDMA), pentaerythritol tetrakis (3-mercaptopropionate) (PETT), and an alkene-labeled recognition element (Figure 2A) from a thiol-terminated surface to form cross-linked polymer brushes that are functionalized covalently with the recognition elements. The value of this reaction for creating sensors was demonstrated previously by the preparation of glycan-modified polymer brushes that could bind GBPs with subfemtomolar avidities^32^ or when modified with *N*-glycans, could bind SCRs.^25^ Here, HP was used to create patterns of **SCR043**-functionalized polymer brushes, where the *h* and grafting density of **SCR043**, Γ, covalently incorporated into the polymer brushes, could be controlled independently at each feature in a microarray pattern.

**Figure 2:**
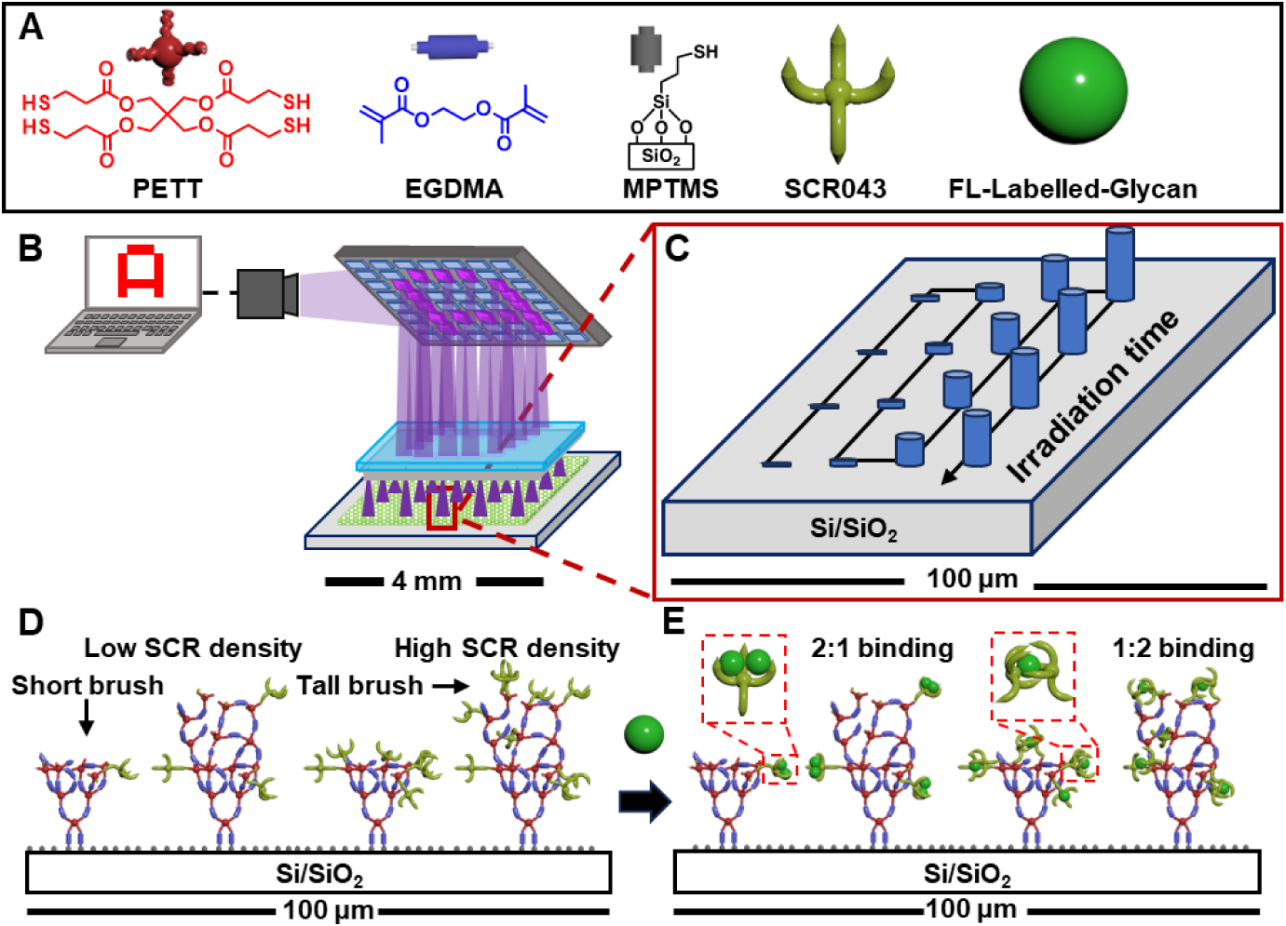
(A) Elements involved in the printing of **SR043**-functionalized polymer brushes. (B) The Hypersurface Photolithography (HP) printer combines a digital micromirror device, a fluid cell, and a surface-initiated grafted-from photopolymerizations to create patterns. (C) Microarrays of polymer brushes with varying heights, *h*, at each feature are prepared by varying the irradiation time, *t*, of each feature during printing. (D) **SCR043**-functionalized polymer brushes of different *h* and **SCR043** grafting density, Γ, are prepared by varying [**SCR043**] in the printing solution and *t*. (E) Binding of fluorescein (FL)-labeled monosaccharide to the array in (D) is dependent upon *h*, Γ, and [monosaccharide], resulting in 1:1, 2:1, or 1:2 monosaccharide:**SCR043** binding.

HP (Figure 2B) is a printing method^30,32,38^ involving a digital micromirror device (DMD) with 1024 × 768 individually controllable mirrors that spatiotemporally controls the delivery of light to the substrate. The reactive substrate is embedded within a fluid-cell containing a photochemically active reaction mixture that reacts with the functional groups on the substrate to drive a surface-initiated grafted-from photopolymerization. An advantage of HP is that a different printing condition can be used in each pixel of the pattern (each pixel corresponding to a mirror in the DMD),^30–32,34^ and so patterns of hundreds of **SCR043**-functionalized polymer brush features (Figure 2C), which can contain features with edge lengths as small as 2 μm and different *h* and Γ (Figure 2D) at each feature, can be prepared. Subsequently, the patterns are incubated into a FL-labeled monosaccharide solution to understand the effects of *h* and Γ on the binding between the **SCR043**-functionalized polymer brushes and monosaccharides (Figure 2E). Additionally, each reaction condition and in turn corresponding polymer brush, can be repeated many times on a surface, providing high fidelity data sets so the variance in *h* or fluorescence can be determined with high precision.

To print the polymer brush patterns (Figure 3A), EGDMA, PETT, **SCR043**, and diphenyl­(2,4,6-trimethylbenzoyl)­phosphine oxide (TPO) are dissolved in DMSO and introduced into the fluid cell of the HP printer and irradiated with patterns of 405 nm light, where the irradiation time, *t*, at each pixel could be independently varied to control feature *h*. After printing, the surfaces were washed with EtOH and dried under air to remove any physisorbed monomers. The presence of patterns was confirmed via optical microscopy (Figure 3B). The *h* of each feature was measured using profilometry (Figure 3C), and the dependence of *h* on *t* was observed. To demonstrate that **SCR043** was incorporated successfully into the polymers, X-ray photoelectron spectroscopy was performed on a **SCR043**-functionalized polymer brush substrate and compared to a substrate containing EGDMA-PETT brushes printed without **SCR043** included in the printing solution. A significant N 1s signal is observed only when **SCR043** is present during printing (Figure 3D), which is consistent with the proposed chemical structures of the polymers. Time-of-flight secondary ion mass spectrometry of the **SCR043**-functionalized polymer brushes (Figure 3E) had prominent fragments with *m*/*z* corresponding to the indole groups of **SCR043**, and this prominent peak is consistent with what had been observed previously for mass spectra of **SCR019** ^20^. These fragments were not observed if **SCR043** was not included in the printing solution, nor were they present in areas of the surface that did not contain the polymer brush features. In Raman microscopy spectroscopic maps (), peaks corresponding to the indole ring of **SCR043** ^39,40^ are only present when analyzing the **SCR043-**functionalized features. Although no single characterization method is sufficient to prove a particular chemical bond is present on a surface,^41,42^ taken together the only reasonable explanation for these data are that **SCR043** is successfully incorporated into the polymer brushes.

**Figure 3:**
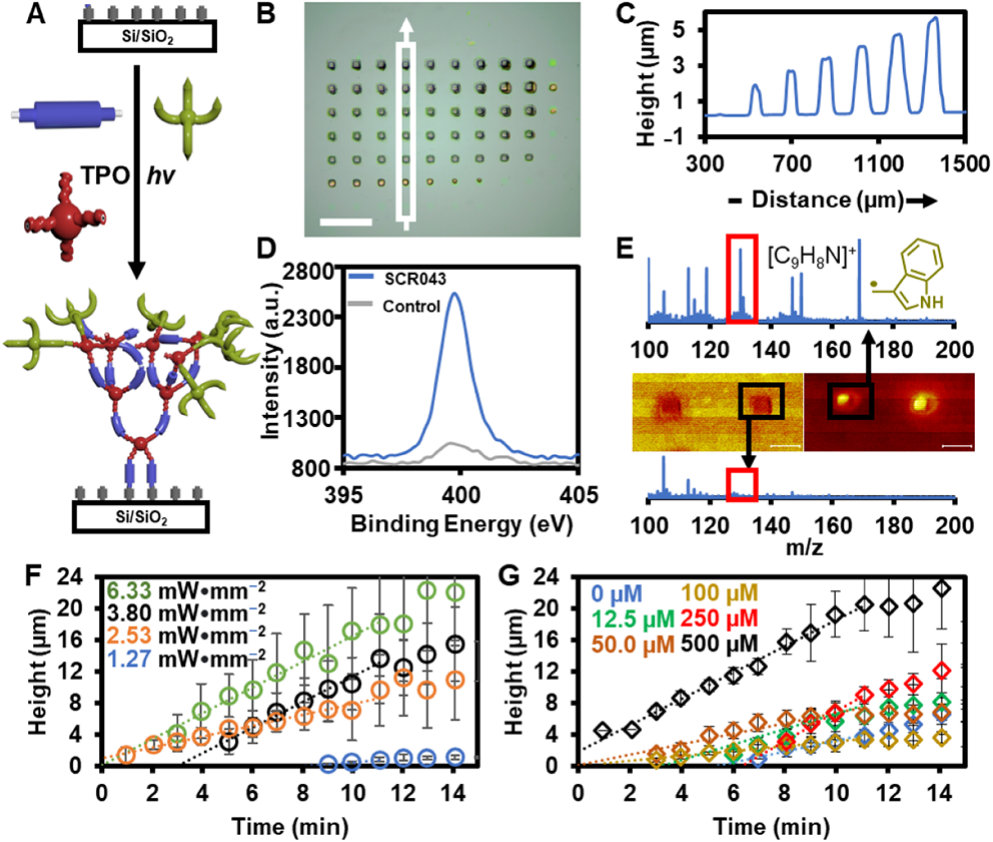
(A) Printing **SCR043-**functionalized polymer brush microarrays with the GTGFRP. (B) Optical microscopy image of **SCR043-**functionalized polymer brush microarray. Scale bar is 200 μm. (C) Profilometry trace of features noted by white box in (B). (D) X-ray photoelectron spectroscopy of patterns containing **SCR043-**functionalized brush polymers and polymer brush features (“control”), which were printed without **SCR043**. (E) Time-of-flight secondary ion mass spectrometry spectra of (middle left) control polymer brush features and (middle right) **SCR043**-functionalized polymer brush features. Total ion images are shown. Red boxes are at *m*/*z* = 130.02, corresponding to the fragment [C_9_H_8_N]^+^. Scale bars are 100 μm. (F) Effect of varying light intensity on brush growth. Error bars are one standard deviation from the mean. Growth rates of the polymer brushes are calculated based on slope of the best-fit linear regression (*R* ^2^ = 0.95–0.99) of the early *t* (1–11 min): *hv =* 1.27 mW·mm^–2^, rate = 4.7 ± 0.02 nm·s^–1^; *hv =* 2.53 mW·mm^–2^, rate = 12 ± 0.04 nm·s^–1^; 3.80 mW·mm^–2^ = 27 nm·sec^–1^ ± 0.1; *hv =* 6.3 mW·mm^–2^, rate = 27 ± 0.1 nm·s^–1^. (G) Effect of varying [**SCR043**] on brush growth rate. Error bars are one standard deviation from the mean. Growth rates of the polymer brushes are calculated based on slope of the best-fit linear regression (*R* ^2^ = 0.90–0.99) of the early *t* (1–11 min): [**SCR043**] = 0 μM, rate = 11 ± 0.1 nm·s^–1^; [**SCR043**] = 12.5 μM, rate = 16 ± 0.1 nm·s^–1^; [**SCR043**] = 50 μM, rate = 11 ± 0.1 nm·s^–1^; [**SCR043**] = 100 μM, 500 ± 0.02 nm·s^–1^; [**SCR043**] = 250 μM, rate = 32 ± 0.1 nm·s^–1^; [**SCR043**] = 500 μM, rate = 28 ± 0.1 nm·s^–1^.

The effect of different printing conditions on the growth rate of polymer brushes was explored systematically so that *h* and Γ could be controlled predictably and precisely at each pixel. A set of surfaces were patterned, where in each surface either light intensity (*hv*) or [**SCR043**] in the printing solution varied, while the other printing parameters are left unchanged. From each printing solution, a 10 × 10 pattern was printed, where *t* of each feature varied from 9.6 to 1050 s. Each of these patterns was repeated 4 times on a surface, so that each *h* that is reported for a given set of conditions is an average of 4 measurements. Polymer brush features with *h* from <1–39 μm were observed. Generally, *h* increased linearly with *t*, until reaching a plateau, and growth rates were determined by fitting the linear region of the resulting plots of *h* vs *t* (Figure 3F) to determine the slope, which is reported as the growth rate. As *hv* increases, the growth rate increases from 4.7 nm·sec^–1^ ± 0.1 for 1.27 mW·mm^–2^ to 27 nm·sec^–1^ ± 0.1 for 3.8 mW·mm^–2^, above which no increase in growth rate was observed with increasing *hv*. Next, polymer brushes were printed with *hv =* 2.53 mW·mm^–2^, and [**SCR043**] in the printing solution varying from 12.5 μM–500 μM (Figure 3G). Growth rate is dependent upon [**SCR043**], although not linearly. At [**SCR043**] = 0 μM, the polymerization rate is 11.3 nm·s^–1^ ± 0.1, and the polymerization rate of the polymers reaches a maximum of 31.5 nm·s^–1^ ± 0.1 at [**SCR043**] = 250 μM. As a result of these studies on polymer brush growth rate, an *h* range of 1–9 μm was achievable for all [**SCR043**] concentrations, and these conditions are used for printing the arrays that are used in subsequent binding studies between the **SCR043**-functionalized polymer brushes and the FL-labeled monosaccharides. It should be noted that in these studies, 1000 different printing conditions were tested to quantify growth rates so that the *h* and Γ could be controlled precisely at each pixel in the microarray, attesting to the remarkable ability of HP to rapidly screen the effects of different reaction conditions on printing outcomes.

### Binding of SCR-Functionalized Polymer Brushes to Fluorescein-Labeled Monosaccharides in Aqueous Buffers

The ability of **SCR043**-functionalized polymer brush microarrays to bind the FL-labeled monosaccharides in aqueous buffers was measured by fluorescence microscopy. The microarrays that were prepared for binding studies consisted of substrates that contained 9 repeats of a 4 × 4 pattern of polymer brush features, where each of the 16 features in a pattern had *h* in the range of 1–9 μm across all surfaces. The substrates were printed with [**SCR043**] = 500, 250, 100, 50.0, 12.5, or 0 μM in the printing solution to explore the effect of Γ, which is assumed to be proportional to the [**SCR043**] in the printing solution, on the binding characteristics of the polymers. Because [**SCR043**] affects polymerization rate (Figure 3G), *t* were adjusted so that the *h* of the 16 features all approximately matched between arrays printed with different [**SCR043**].

Binding between the arrays and the 5 labeled monosaccharides was studied as follows (Figure 4A): solutions of the FL-labeled monosaccharides were prepared in 20 mM Tris buffer (pH = 7.4, 0.01% Tween20) at concentrations of 10^–3^, 10^–3.5^, 10^–4^, 10^–4.5^, 10^–5^, and 10^–5.5^ M (monosaccharides were insoluble at concentrations >10^–3^ M). The arrays were immersed in solutions of the monosaccharide for 1 h at 25 °C, washed in Milli-Q water with 0.01% Tween20 for 10 min to remove any nonspecifically adsorbed monosaccharides, and dried under a stream of air. After washing, the fluorescence of the microarrays was measured by fluorescence microscopy. The fluorescence intensity of each feature, *I*, was reported as the ratio of the intensity of feature and the intensity of background. The fluorescence micrograph for the array printed at **SCR043** = 100 μM and exposed to the [**α-Man-FL**] = 10^–3^ M solution is shown in Figure 4B and C. The green features in the 4 × 4 array, with *I* of >1.2–5.4, are evidence of binding of **α-Man-FL** to the **SCR043**-functionalized polymer brushes, and confirmation that **SCR043**, when immobilized in a multivalent manner on polymer brushes, can indeed bind monosaccharides in aqueous buffers.

**Figure 4:**
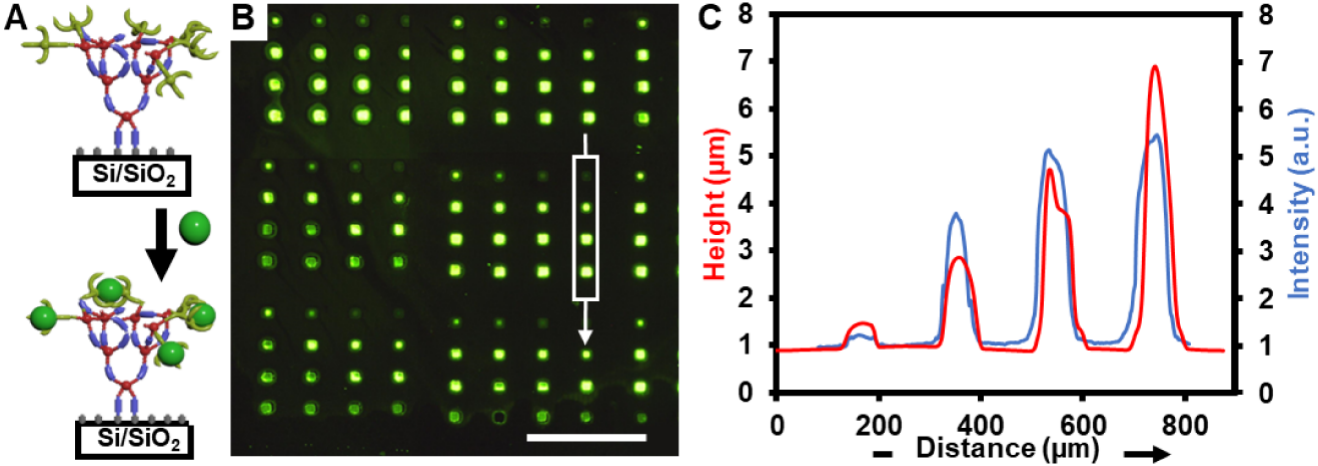
(A) Binding of **SCR043**-functionalized brush polymers to **α-Man-FL**. (B) Fluorescent micrograph showing binding between **SCR043**-functionalized brush polymers. [**SCR043**] = 100 μM), [**α-Man-FL**] = 10^–4^ M in Tris buffer 20 mM, MnCl_2_ 0.9 mM, CaCl_2_ 0.5 mM, pH = 7.4, 0.01% Tween20. Scale bar is 200 μm. (C) Height (red) and normalized fluorescence intensity (blue, arbitrary units) for the features indicated by the white box in (B).

A series of control experiments were carried out to confirm that this fluorescence pattern was the result of interactions between the monosaccharides and **SCR043** on the polymer brushes and not the result of nonspecific interactions. Polymer brush arrays printed with [**SCR043**] = 500 μM and 2.53 mW·mm^–2^ were exposed to solutions of FL (10^–4^ M–10^–3^ M), and *I* never exceeded the noise threshold (*I* = 1.2). Alternatively, polymer brush arrays that did not contain **SCR043** were exposed to solutions of **α-Man-FL**. For [**α-Man-FL**] = 10^–3^ M an *I* = 2.5 was observed for the polymer brushes of *h* = 7 μm that were printed without **SCR043**, which is substantially lower than the *I* = 6 for the **SCR043**-functionalized polymer brushes of the same *h*, suggesting that, even at the highest concentrations, if any nonspecific absorption occurs, it is substantially less than the specific binding that occurs when the **SCR043** is present. However, these high levels of nonspecific absorption were observed at glycan concentrations of 10^–3^ and 10^–3.5^ M. Taken together, these control experiments confirm that the observed fluorescence is the result of specific interactions between the monosaccharides and the immobilized **SCR043**.

The effect of varying Γ on binding between **SCR043**-functionalized brushes and **α-Man-FL** was analyzed. Arrays of 4 × 4 patterns of **SCR043**-functionalized brush polymers, with *h* = 1 – 8 μm and Γ = 500, 250, 100, 50, and 12.5 μM. **α-Man** binds **SCR019** cooperatively in solution,^20^ and it is anticipated that the ability to access these cooperative binding modes in **SCR043**-functionalized polymer brushes is dependent upon Γ. The binding between the **SCR043**-functionalized polymer brushes and **α-Man-FL** was indeed dependent upon Γ. *I* did not increase monotonically with increasing Γ (Figure 5A). Generally, for any given *h, I* was greatest when Γ = 100 μM followed by Γ = 50 μM, except for the tallest features, in which *I* was greatest when Γ = 500 μM, and this outlier is likely the result of greater nonspecific adhesion occurring in the largest polymers (Figure 5A). This observation—that binding does not increase monotonically with concentration of glycan-recognition elements on surfaces (Γ)—is consistent with other studies exploring the role of valency on glycan-GBP binding in microarrays, where it was also observed that arrays where GBPs or glycans are most densely immobilized do not necessarily bind most strongly.^14,43^ Since Γ = 100 μM and Γ = 50 μM both showed the greatest *I* throughout the [**α-Man-FL**] concentrations (Figure 5B), microarrays used to study the binding of the of the other monosaccharides to the polymer brushes were printed at Γ = 100 μM.

**Figure 5:**
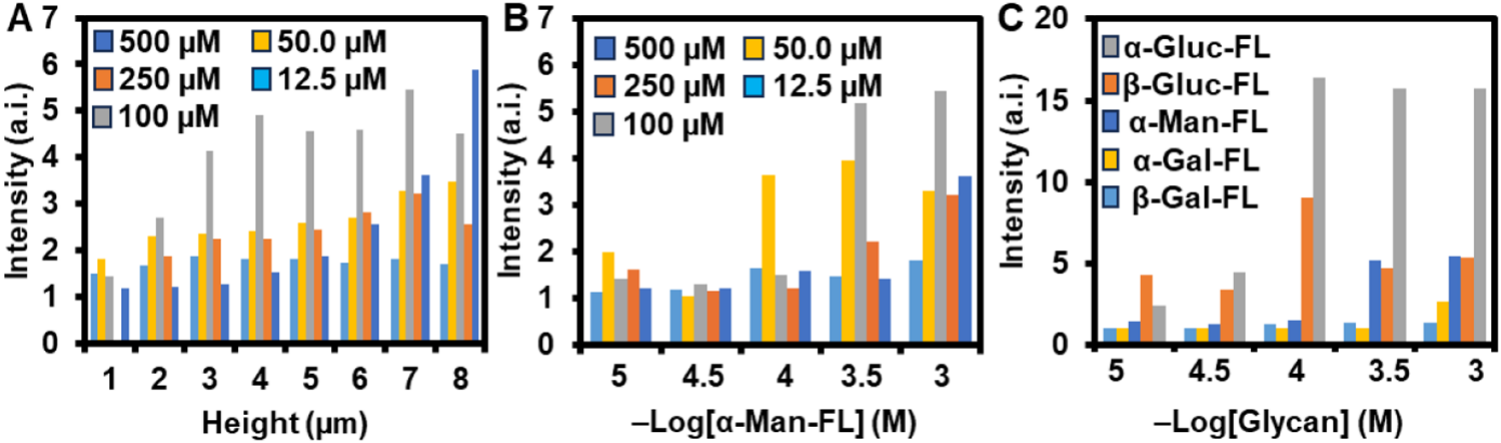
(A) Graph of *I vs h* for the binding of [**α-Man-FL**] = 10^–3^ M to polymer brushes prepared with varying [**SCR043**] in the printing solutions and in turn, Γ. The different colors represent different [**SCR043**] in the printing solution. (B) Graph of *I vs* [**α-Man-FL**] for the binding of varying SCR043-functionalized polymer brushes prepared with *h* = 7 μm. The different colors represent different [**SCR043**] in the printing solution. (C) Graph of *I vs* [monosaccharide] for the binding of constant Γ = 100 μM polymer brushes prepared with *h* = 7 μm. The different colors represent different monosaccharides.

Following this proof-of-concept demonstration, the binding of each of the 5 monosaccharides was measured at 6 concentrations (10^–3^–10^–5.5^ M) against **SCR043**-functionalized polymer brush arrays printed at Γ = 100 μM, with 16 different *h* in each array (). As each feature in an array can be considered a binding experiment, and the complete data set used to analyze binding between glycans and **SCR043**-functionalized polymer brushes is composed of 9072 unique binding experiments, each repeated 4 times to provide values with statistical significance. To organize the data, the *I* for each feature is matched to its *h*, Γ, and [glycan] to generate *I*_(*h*, glycan, log[glycan])_ for all features (Figure 4C). For example, the features printed with at Γ = 100 μM, *h* = 7 μm, when exposed to the glycan [**α-Man-FL**] = 10^–3^ M, has *I* = 4, so its *I*_(7, α‑Man‑FL, –3)_ = 4. Fluorescent micrographs, profilometry images, and tables of *h* and *I*_(*h*, glycan, log[glycan])_ are provided for all microarrays in the .

Binding was observed between the **SCR043**-functionalized brushes for **α-Man-FL** and both **α-Gluc-FL** and **β-Gluc-FL** but was below the noise threshold for **β-Gal-FL** and **α-Gal-FL**, except at the highest concentrations (10^–3^) and *h* > 3 μm, where control experiments indicate that substantial nonspecific aggregation occurs. Based on these data, we determined that the arrays did not bind galactosides within the detection limit of the array. Statistically significant differences in *I* and in turn binding, were observed between **α-Man-FL** and both galactosides, **α-Gal-FL** and **β-Gal-FL** at all glycan concentrations and *h*. For all experiments, *I*_(*h*, [**α‑Man‑FL**])_ > *I*_(*h*, [**α‑Gal‑FL**])_ when all other parameters besides monosaccharide structure were equal, indicating a clear selectivity for **α-Man** over galactosides. An unpaired *t*-test comparing the *I* values between [**α-Man-FL**] and [**α-Gal-FL**] fluorescence shows that there is a statistical difference in binding with 95% confidence. Moreover, this binding preference for mannosides over galactosides is similar to the trend that was observed for **SCR019** in CD_2_Cl_2_ ^20^ and DFT, where **SCR019** was reported to have bound both monosaccharides but with a preference for mannosides. As such these results demonstrate that arrays containing SCRs as the glycan recognition element have the selectivity to distinguish between monosaccharide diastereomers.

### Determination of *K*_d_s from Microarray Data Sets

*K*_d_, a commonly used measure of the binding avidity to microarrays,^32^ was calculated from the *I* of each of the total 9072 binding experiments that were carried out using the **SCR043**-functionalized microarrays and the five fluorescently labeled monosaccharides. *K*_d_ is determined from *I* using the Langmuir isotherm model^32^ (eq 1),

1 $$K_{d}=\left[L\right]\left(\frac{I_{\max}}{I}-1\right)$$

where [*L*] is glycan concentration, *I*_max_ is the maximum normalized fluorescence intensity for each glycan at a fixed *h* and Γ, and *I* is the normalized fluorescence intensity of the feature. The Langmuir isotherm was chosen for determining *K*_d_ because it is the most common model applied to microarrays,^31,32,38,44^ and so that the *K*_d_ reported here could be directly compared to binding on other glycan-binding microarrays. *K*_d_ ranged from 4 μM for Γ_(50.0, 6, [**α‑Man‑FL**], –4)_ (strongest binding) to 21 mM for Γ_(100, 1,[**α‑Man‑FL**], –3)_ (weakest binding). As such, 21 mM was determined to be the sensitivity limit of the array. Both **β-Gal-FL** and **α-Gal-FL** only show binding at glycan = 10^–3^ M–10^–4^ M, with only some features with fluorescence that is detectable above the noise floor of *I* ≥ 1.2 and do not show fluorescence at concentrations <10^–4^ M. Generally, when all other parameters are held constant, *K*_d_ follows the trend of [**α-Man-FL**] < [**α-Gluc-FL**] < [**β-Gluc-FL**] ≪ [**β-Gal-FL**] ∼ [**α-Gal-FL**].

*K*_d_, however, when calculated using the Langmuir isotherm, does not account for cooperativity and multivalency,^45^ and this limitation is reflected in the data. *K*_d_, in the absence of cooperative and multivalent binding, are expected to remain constant with varying *h* and [monosaccharide].^45^ *K*_d_ calculated from the SCR microarray data however, vary significantly with *h* and [monosaccharide] for [**α-Gluc-FL**], [**β-Gluc-FL**], and [**α-Man-FL**] (Figure 6A). As [monosaccharide] decreases, *K*_d_ for both short and tall features increase for all monosaccharides. As such, these data suggest that for mannosides and glucosides, some form of cluster glycoside effects exist and demonstrate that *K*_d_ alone is an incomplete descriptor for comparing the binding of glycans to the **SCR043**-modified polymer brush recognition elements in the arrays. An ideal descriptor of binding would be independent of *h* and [glycan], and to do so must be derived from a model that can account for cooperativity. When keeping all parameters constant, we see *K*_d_ becomes stronger as the *h* increases. For example, at [**α-Man-FL**] = 10^–3.5^ and Γ = 100 M, as *h* increases from 1 to 8 μm, *K*_d_ decreases from 0.8 mM to 15 μM. When [**α-Man-FL**] decreases from 10^–3^ to 10^–4^ (*h* = 1, Γ = 100 μM), *K*_d_ decreases from 2.8 mM to 0.4 mM.

**Figure 6:**
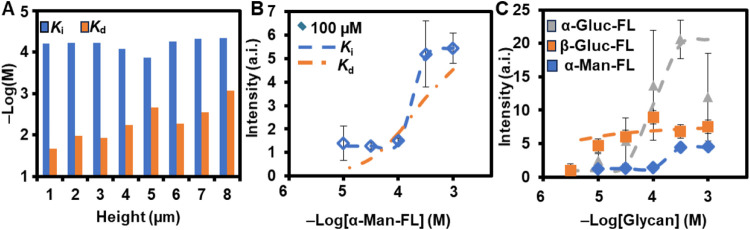
(A) Binding strength vs *h* with Γ = 100 μM and [**α-Gluc-FL**] = 10^–3^ M for both *K*_i_ and *K*_d_. (B) Hill plot for binding between **SCR043**-functionalized brush polymers and **α-Man-FL** with varying Γ, *h* = 7 μm (*R* ^2^ = 0.97–0.99). Error bars represent one standard deviation from the mean. (C) Hill plot for binding between **SCR043**-functionalized brush polymers and varying monosaccharides with Γ = 100 μM, *h* = 8 μm (*R* ^2^ = 0.94–0.99). Error bars represent one standard deviation from the mean.

### Determination of *K*_i_s from Microarray Data Sets

To account for cooperative and multivalent interactions occurring within the polymer brush features, *K*_i_—which is a measure of the binding strength that accounts for the presence of multiple cooperative binding interactions between a receptor and multiple ligands, and this has been shown to be an appropriate fitting model for systems involving multiple ligands appended to flexible polymers^46–49^ —was calculated using eq 2,

2 $$K_{i}=\frac{IC_{50}K_{d}}{\left[L\right]+K_{d}}$$

where *IC*_50_ is the 50% intensity of the binding curve that is calculated from fitting the experimental data (Figure 6B) of *I* vs −Log­[monosaccharide]. *K*_d_ is calculated from eq 1, and [*L*] is the concentration of monosaccharide. Figure 6B shows binding curves for **α-Man-FL** with Γ = 500 μM, *h* = 7 μm and Γ = 100 μM, *h* = 7 μm are fit to both eqs 1 and 2. When comparing the fits to the data, the latter provided a superior fit, with a much lower residual. When comparing *K*_d_ to *K*_i_ (Figure 6B), the former varies with *h*, whereas the latter is almost independent of *h* (Figure 6A). This ability to account for cooperative and multivalent interactions in eq 2 is embedded within the *IC*_50_ parameter.^49^ As such, *K*_i_ is a *h-*independent descriptor of the binding strength of monosaccharides to these multivalent **SCR043**-functionalized polymer brush microarrays.

Subsequently, all binding curves for the interactions between all monosaccharides to **SCR043**-functionaled brush polymers were fit using eq 2 to determine *K*_i_ (Figure 6C). *K*_i_ ranged from 5 μM for **α-Man-FL** to 500 μM for **β-Gluc-FL**. The *K*_d_ for **α-Gluc-FL** = 10^–3^ and Γ = 100 μM, ranges from 20 mM at *h* = 7, to 0.85 mM at *h* = 8, whereas *K*_i_ for the same binding pair is invariant and 72 ± 28 μM. **α-Man-FL** shows the strongest binding, with the average *K*_i_ of 55 ± 62 μM, followed by **α-Gluc-FL** with a *K*_i_ of 70 ± 40 μM, and **β-Gluc-FL** of 150 ± 153 μM. *K*_i_ for **β-Gal**-FL nor **α-Gal**-FL could be calculated as a binding isotherm was not measurable, and as such, the *K*_i_ was deemed to be below the sensitivity of the array. Finally, Δ*G* ^o^ of binding was calculated from *K*_i_ using the Gibbs equation (eq 3),^50^

$$\Delta G^{o}=-RT\ln K_{i}$$ 3

where *R* is the universal gas constant, and *T* is temperature (Table 1). The range for experimental Δ*G* ^o^ values are −5.2 to −5.8 kcal·mol^–1^. **α-Man-FL** is the strongest binder to **SCR043**-functionalized polymer brushes printed at Γ = 100 μM, at −5.8 ± 0.7 kcal·mol^–1^ followed by **α-Gluc-FL** at – 5.67 ± 0.3 kcal·mol^–1^, and then **β-Gluc-FL** at −5.2 ± 0.6 kcal·mol^–1^. Notably, both **β-Gal-FL**, and **α-Gal-FL** had fluorescence signals too low to determine *IC*_50_ and *K*_i_ because the fluorescence is below the detection limit of *I =* 1.2. From these Δ*G* ^o^ values derived from the analysis of the microarrays, the selectivity of these **SCR043**-functionalized polymer brush recognition elements is summarized as follows. The arrays bind **α-Man-FL > α-Gluc-FL > β-Gluc-FL > > β-Gal-FL ∼ α-Gal-FL.** This type of promiscuous binding that is observed in the polymer brushes in this microarray, where a glycan-recognition element binds several monosaccharide diastereomers, is common among lectins. A relevant example is the lectin Concanavalin A (ConA) that binds glucosides and mannosides, with a preference for α-mannosides.^51^ It is also interesting to note that another similarity between the SCR-monosaccharide binding and ConA-monosaccharide binding is that both are sensitively dependent on the density of the lectin receptors at interfaces.^8^

**Table 1:** Thermodynamic Binding Parameters between **SCR019** and Monosaccharides

**Glycan**	Δ ***E***_1:1_ **(kcal·mol** ^**– 1**^ **)**	Δ ***E***_2:1_ **(kcal·mol** ^**– 1**^ **)**	ΔΔ ***E***_2:1_ **(kcal·mol** ^**– 1**^ **)**	Δ ***E***_1:2_ **(kcal·mol** ^**– 1**^ **)**	ΔΔ ***E***_1:2_ **(kcal·mol** ^**– 1**^ **)**	***K***_**d**_ (μ**M)**	***K***_**i**_ (μ**M)**	Δ ***G*** ° **(kcal·mol** ^**– 1**^ **)**
α-Man	–15.6^a^	–38.9^b^	–23.3^c^	–35.5^d^	–19.9^e^	15^f^	55^g^	–5.81^h^
α-Gluc	–16.9^a^	–38.8^b^	–21.9^c^	–37.5^d^	–20.6^e^	130^f^	70^g^	–5.67^h^
β-Gluc	–12.1^a^	–36.9^b^	–24.8^c^	–36.9^d^	–24.8^e^	330^f^	150^g^	–5.21^h^
α-Gal	–18.1^a^	–45.9^b^	–27.8^c^	–35.2^d^	–17.1^e^	_^i^	_^i^	_^i^
β-Gal	–14.9^a^	–31.5^b^	–16.6^c^	–24.2^d^	–9.3^e^	_^i^	_^i^	_^i^

a Binding energy (Δ*E*_1:1_) in kcal·mol^–1^ for one **SCR019** and one monosaccharide (**SCR019:** monosaccharide).

b Binding energy (Δ*E*_2:1_) in kcal·mol^–1^ for two **SCR019** and one monosaccharide (**SCR019**_**2**_:monosaccharide).

c Difference in binding energy (ΔΔ*E*_2:1_) from Δ*E*_1:1_ to Δ*E*_2:1_.

d Binding energy (Δ*E*_1:2_) in kcal·mol^–1^ for one **SCR019** and two monosaccharides (**SCR019:**monosaccharide_2_).

e Difference in binding energy (ΔΔ*E*_1:2_) from Δ*E*_1:1_ to Δ*E*_1:2_.

f Dissociation constants (*K*_d_) (μM) for [monosaccharide] = 10^–3^ M, *h* = 7 μm, and **SCR043**. IC_50_ (μM) for various monosaccharides.

g Inhibition constants (*K*_i_) (μM) for [monosaccharide] = 10^–3^. *K*_i_ (μM) for [monosaccharide] = 10^–3^.

h Experimental △*G*° (kcal·mol^–1^) calculated from corresponding *K*_i_.

i Fluorescence below noise level of *I* = 1.2.

### Cooperativity in Binding to SCR043-Functionalized Polymer Brushes

*H*_c_ ^52,53^ is a measure of the cooperativity of binding in a multivalent system, where *H*_*c*_ < 1 signifies negative cooperativity, whereas *H*_*c*_ > 1 signifies positive cooperativity. *H*_c_ were determined using the best fit of the Log­[monosaccharide] vs *I* (e.g., Hill Plot) to the Hill equation (eq 4),

4 $$H_{c}=\log\left(\frac{\left(2I_{\min}-I_{\max}-I\right)\left[L\right]}{IC_{50}}\right)$$

where *I*_min_ is the minimum normalized fluorescence for a monosaccharide among features with the same *h* and Γ. The Hill plot for the binding of **α-Man-FL** and **SCR043**-functionalized brush polymers with *h =* 7 μm, Γ = 100 μM is provided in Figure 6B. Similarly, the Hill plots for the binding of Γ = 100 μM **SCR043**-functionalized brush polymers and **α-Man-FL**, **α-Gluc-FL**, and **β-Gluc-FL** with *h =* 8 μm are provided in Figure 6C. All *H*_c_ with *R* ^2^ ≥ 0.94 are provided in . For all three monosaccharides, *H*_c_ above 1 were obtained, indicating positive cooperativity. The largest value for **β-Gluc-FL** is *H*_c (4, 100, **β‑Gluc‑FL**)_ = 17.3. The largest value for **α-Man-FL** is *H*_c (1, 100, **α‑Man‑FL**)_ = 12.4. The largest value for **α-Gluc-FL** is *H*_c (4, 100, **α‑Gluc‑FL**)_ = 10.4. Interestingly, we observed that *H*_c_ varied with *h*, a phenomenon which will require further study to understand.

### Density Functional Theory Simulations of Binding of SCR019 to Monosaccharides

The cooperativity observed in the binding of **α-Man-FL, α-Gluc-FL,** and **β-Gluc-FL to the SCR043**-functionalized polymer brushes can be explained by considering the 2:1, 1:1, and 1:2 SCR:monosaccharide equilibria^22^ that occur between the **SCR043** and **α-Man-FL** that have been observed previously in the binding between **SCR019** and **α-Man**.^20^ To this end, the binding between **α-Man, α-Gluc**, **β-Gluc**, **β-Gal,** and **α-Gal** and **SCR019** were modeled using DFT. The fluorescent label of the monosaccharides has been replaced with *n*-butyl chain and the *N*-alkane of **SCR043** was not included in the calculations because control experiments showed that no binding occurred between the fluorescent dyes and the **SCR043**-functionalized polymer brush array (), and the large computational cost of including the additional atoms in the studies, which leaves the structure of **SCR019**. The details of the conformational search performed to find the lowest-energy structures are discussed in the , and here are only presented structures and binding energies (Δ*E*) of the most stable complexes. Briefly, for each stoichiometric complex, an initial population of conformers was generated using two conformational searches initiated from unique structures using the iMTD-GC algorithm^54^ implemented in CREST^55^ and GFN2-xTB energy function.^56^ The resulting conformers were merged and clustered, and at least 100 lowest-energy conformers for each system were reoptimized in FHI-aims^57^ at dispersion corrected PBE+vdW^TS^ level of theory.^58,59^ The Δ*E* of the 50 lowest energy conformers were then calculated as a single point at PBE0+MBD^60^ level of theory and solvent effects were accounted for using the PCM model.^61^ For nonstoichiometric complexes, eight independent conformational searches were performed, starting from the lowest energy stoichiometric complex with an additional monosaccharide or **SCR019** added to the system. The resulting structures were merged and clustered, and the 50 lowest energy conformers were subject to the same Δ*E* evaluation as the stoichiometric complexes. The structures and Δ*E* of complexes of **SCR019:α-Man** are shown in Figure 7 and all Δ*E* in Table 1. We observe that the axially oriented hydroxyl group at C2 of the **α-Man** binds between two arms of the receptor, similar to previous reports on the binding between SCRs and α-mannosides,^22^ whereas **α-Gal** forms a weaker, nonspecific complex. Interestingly, the predicted Δ*E* from DFT simulations of **α-Man** and **SCR019** is −15.6 kcal·mol^–1^, which is 2.5 kcal·mol^–1^ weaker than the Δ*E* of **α-Gal** (−18.1 kcal·mol^–1^). However, the **α-Man:SCR019** complex can further bind with another glycan to form **SCR019:α-Man**_**2**_, and the second binding event energy of **α-Man** is stronger by 4.3 kcal·mol^–1^ (Δ*E*_2:1_ = −19.9 kcal·mol^–1^) than the first Δ*E*, whereas the second Δ*E* of **α-Gal** to form **SCR019:α-Gal**_**2**_ (Δ*E*_2:1_ = –17.1 kcal·mol^–1^) is weaker by 0.9 kcal·mol^–1^. This is consistent with the nonstoichiometric binding between **SCR019:α-Man**_**2**_ complexes described previously.^20–22^ In contrast, **SCR019** would form preferably only 1:1 complex with **α-Gal**, as the second (Δ*E*_2:1_) is smaller than the first (Δ*E*_1:1_). The observed increase in **SCR019** Δ*E* can be assigned to additional glycan–glycan interactions between two **α-Man**, such as **SCR019:α-Man**_**2**_ and **SCR019**_**2**_ **:α-Man**, that are not formed in the complex with **α-Gal**. The calculations also predict an increase in Δ*E* by 7.7 kcal·mol^–1^ as a result of the Δ*E* of the second receptor in the **SCR019**_**2**_ **:α-Man,** a *H*_*c*_ > 1 complex, where the glycan occupies a pocket formed between two receptors. It should be noted that a similar increase for the Δ*E* by 9.7 kcal·mol^–1^ is observed for the binding of the second receptor in **SCR019**_**2**_ **:α-Gal** complex, which could indicate cooperative binding at low glycan concentrations. As such, these calculations are consistent with the observation that at high **SCR043:α-Man**, a *H*_*c*_ > 1 occurs because of the formation of **SCR043**_**2**_ **:α-Man-FL**, and at a low **SCR043:α-Man,** a *H*_*c*_ > 1 occurs because of the formation of **SCR043:α-Man-FL**_**2**_.

**Figure 7:**
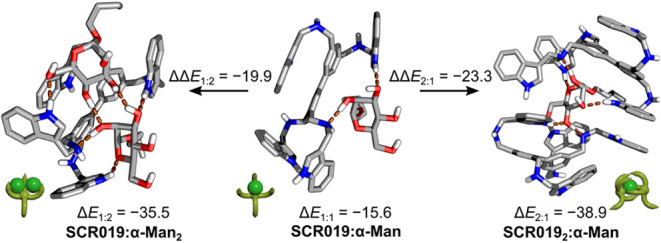
Binding energies, Δ*E*, and incremental binding energies, ΔΔ*E*, (kcal·mol^–1^) of **SCR019** with **α-Man**. The Δ*E* of the first monosaccharide to **SCR019** is shown in the central column, and the Δ*E* and ΔΔ*E* of the second monosaccharide and the second receptor are shown, respectively, in the left and right column. The brown dashes show H-bonds between the receptor and the monosaccharide.

Based on the thermodynamic data, several trends emerge for the binding of **SCR043**-functionalized polymer brushes to the different monosaccharide diastereomers. **α-Man** and **α-Gluc**/**β-Gluc** show Δ*E* transitions from one binding event of one glycan and SCR, to two glycans per single SCR in the DFT simulations, Δ*E*_2:1_ ranging from −21.9 to −24.8 kcal·mol^–1^ and Δ*G*° of −5.2 to −5.8 kcal·mol^–1^, with experimental *K*_d_ between 15–330 μM. This is notably weaker in comparison to the theoretical binding strength associated with Δ*E*_2:1_. In contrast, **β-Gal-FL**, and **α-Gal-FL** bind less strongly (*K*_d_ = 900 μM) despite having similarly favorable expected Δ*E*_2:1_. Among the measurable interactions, **α-Man-FL** demonstrates the strongest overall binding with a *K*_i_ of 55 μM, followed closely by **β-Gluc-FL** at 150 μM and **α-Gluc-FL** at 70 μM. These values also more so match the expected values associated with the theorized Δ*E*_2:1_. The glucose anomers show an interesting pattern where the α-anomer (*K*_i_ = 70 μM) is approximately twice as strong a binder as the β-anomer (*K*_i_ = 150 μM), suggesting that the axial glycosidic bond in **α-Gluc** provides more favorable interactions for binding. The data reveals that anomeric configuration influences binding thermodynamics.

## Conclusions

In conclusion, this study presents a significant advancement in the development of glycan-binding microarrays by establishing SCRs as effective glycan-binding recognition elements. By leveraging the high-throughput optimization capabilities of HP^30–34^ to develop printing conditions where *h* and Γ could be finely tuned at each feature, we are able to prepare surfaces that exploit cluster-glycoside effects to enhance the binding between monosaccharides and recognition elements, thereby enhancing both selectivity and sensitivity.

Detailed binding studies enabled by the large data sets revealed the important roles of cooperativity and multivalency in binding. This work demonstrates the importance of considering the idiosyncrasies of glycan-binding and considering how to capitalize on them to increase binding strength and specificity in glycan-binding microarray design. Importantly, the observed preferential binding of **SCR043** for mannosides over other glycans—and the presence of multiple binding equilibria—corroborates earlier observations from NMR studies,^20–22^ suggesting that the binding selectiviities in microarrays can be extrapolated from solution studies. It should be noted that binding between only β-mannosides but not α-mannosides and **SCR019** was observed in CD_2_Cl_2_, and **SCR019** did not bind galactosides at all, although the binding between α-mannosides and **SCR019** was predicted computationally.^23^

Furthermore, this study highlights the limitations of *K*_d_ as a descriptor for binding within these biomimetic scaffolds. *K*_d_s as low as 3.9 μM were observed, and, remarkably, monosaccharides bound to this array at micromolar concentrations. The microarrays can distinguish between mannosides and galactosides,^20–22^ with a confidence of 95% when subjected to an unpaired *t*-test. The counterintuitive result that tall brushes had higher *K*_d_s (weaker binding) than shorter brushes is a result of the failure of the Langmuir isotherm model to account for multivalency, and that *K*_i_ was less dependent on *h*. By determining *H*_c_, we found that 1:1, 2:1, and 1:2 **α-Man-FL**:**SCR043** cooperative binding modes all occur within the Γ polymer brushes, and the predominant binding ratio was dependent upon Γ, *h*, and the [glycan].

Given that SCRs with different selectivities and binding affinities exist, the strategy reported herein for preparing glycan-binding microarrays can be expanded to create multiplexed microarrays that could distinguish between a broad range of glycans and could lead to the wider adoption of glycan-binding microarrays in diagnostics and biological research. Although here the glycans are labeled to facilitate validation of the binding and analysis of the supramolecular interactions, like lectins^6,12,62–67^ and antibodies,^65,68,69^ SCRs can be incorporated into other transducer architectures that do not require labeling of the target glycan for detection. The SCR-labeled polymer brushes are a novel recognition element that could be used to detect glycan binding and determine *K*_d_ for unlabeled glycans, but doing so requires careful consideration of the experimental approach. To use these SCR-functionalized microarrays for diagnostic and research purposes, examining the binding against a broad range of *O*- and *N*-glycans will be necessary, but computational studies^23^ suggest that specificity in a biologically relevant context is achievable.

## Supplementary Material





## Notes

“ jp5c06186_si_001.pdf” is a word document containing all written and imaged materials associated with this work, including synthesis, spectral data, fluorescence images, height data, and DFT methods (PDF)
“ jp5c06186_si_002.zip” is a zip file containing all xyz files determined by DFT calculations (ZIP)

## References

[1] Gagneux, P. ; Hennet, T. ; Varki, A. Biological Functions of Glycans, In Essentials of Glycobiology, 4th ed.; Varki, A. ; Cummings, R. D. ; Esko, J. D. ; Stanley, P. ; Hart, G. W. ; Aebi, M. ; Mohnen, D. ; Kinoshita, T. ; Packer, N. H. ; Prestegard, J. H. ; Schnaar, R. L. ; Seeberger, P. H. Eds.; Cold Spring Harbor; 2022, pp. 79–92. 35536978

[2] Lundquist J.
J., Toone E. J. (2002). The cluster
glycoside effect. Chem. Rev..

[3] Oyelaran O., Gildersleeve J. C. (2009). Glycan
arrays: recent advances and future challenges. Curr. Opin. Chem. Biol..

[4] Temme J. S., Crainic J. A., Walker L. M., Yang W., Tan Z., Huang X., Gildersleeve J. C. (2022). Microarray-guided
evaluation of the
frequency, B-cell origins, and selectivity of human glycan-binding
antibodies reveals new insights and novel antibodies. J. Biol. Chem..

[5] Kiessling L. L., Grim J. C. (2013). Glycopolymer probes of signal transduction. Chem. Soc. Rev..

[6] Godula K., Bertozzi C. R. (2012). Density variant
glycan microarray for evaluating cross-linking
of mucin-like glycoconjugates by lectins. J.
Am. Chem. Soc..

[7] Dam T. K., Roy R., Page D., Brewer C. F. (2002). Thermodynamic binding parameters
of individual epitopes of multivalent carbohydrates to concanavalin
a as determined by “reverse” isothermal titration microcalorimetry. Biochemistry.

[8] Valles D. J., Zholdassov Y. S., Korpanty J., Uddin S., Naeem Y., Mootoo D. R., Gianneschi N. C., Braunschweig A. B. (2021). Glycopolymer
Microarrays with Sub-Femtomolar Avidity for Glycan Binding Proteins
Prepared by Grafted-To/Grafted-From Photopolymerizations. Angew. Chem., Int. Ed..

[9] Ribeiro J. P., Mahal L. K. (2013). Dot by dot: analyzing
the glycome using lectin microarrays. Curr.
Opin. Chem. Biol..

[10] Liao H. Y., Hsu C. H., Wang S. C., Liang C. H., Yen H. Y., Su C. Y., Chen C. H., Jan J. T., Ren C. T., Chen C. H. (2010). Differential receptor
binding affinities of influenza
hemagglutinins on glycan arrays. J. Am. Chem.
Soc..

[11] Tateno H., Mori A., Uchiyama N., Yabe R., Iwaki J., Shikanai T., Angata T., Narimatsu H., Hirabayashi J. (2008). Glycoconjugate microarray based on an evanescent-field
fluorescence-assisted detection principle for investigation of glycan-binding
proteins. Glycobiology.

[12] Hu S., Wong D. T. (2009). Lectin microarray. Proteomics:
Clin. Appl.

[13] Hyun J. Y., Pai J., Shin I. (2017). The Glycan Microarray Story from Construction to Applications. Acc. Chem. Res..

[14] Braunschweig A., Byrne J. P., Chiechi R., Diaz Fernandez Y., Gildersleeve J., Godula K., Hartmann L., Mahon C., Miura Y., Nelson A. (2019). Preparation
of multivalent
glycan micro- and nano-arrays: general discussion. Faraday Discuss.

[15] Hsu K. L., Gildersleeve J. C., Mahal L. K. (2008). A simple strategy for the creation
of a recombinant lectin microarray. Mol. BioSyst..

[16] Reily C., Stewart T. J., Renfrow M. B., Novak J. (2019). Glycosylation in health
and disease. Nat. Rev. Nephrol..

[17] Reily C., Stewart T. J., Renfrow M. B., Novak J. (2025). Publisher Correction:
Glycosylation in health and disease. Nat. Rev.
Nephrol..

[18] Djalali S., Yadav N., Delbianco M. (2024). Towards glycan foldamers and programmable
assemblies. Nat. Rev. Mater..

[19] Bravo M. F., Lema M. A., Marianski M., Braunschweig A. B. (2021). Flexible
Synthetic Carbohydrate Receptors as Inhibitors of Viral Attachment. Biochemistry.

[20] Bravo M. F., Palanichamy K., Shlain M. A., Schiro F., Naeem Y., Marianski M., Braunschweig A. B. (2020). Synthesis and Binding of Mannose-Specific
Synthetic Carbohydrate Receptors. Chem. - Eur.
J..

[21] Palanichamy K., Bravo M. F., Shlain M. A., Schiro F., Naeem Y., Marianski M., Braunschweig A. B. (2018). Binding Studies on a Library of Induced-Fit
Synthetic Carbohydrate Receptors with Mannoside Selectivity. Chem. - Eur. J..

[22] Rieth S., Miner M. R., Chang C. M., Hurlocker B., Braunschweig A. B. (2013). Saccharide receptor achieves concentration
dependent
mannoside selectivity through two distinct cooperative binding pathways. Chem. Sci..

[23] Tapia B., Yagudayeva G., Bravo M. F., Thakur K., Braunschweig A. B., Marianski M. (2022). Binding of synthetic carbohydrate receptors to enveloped
virus glycans: Insights from molecular dynamics simulations. Carbohydr. Res..

[24] Thakur K., Shlain M. A., Marianski M., Braunschweig A. B. (2021). Regiochemical
Effects on the Carbohydrate Binding and Selectivity of Flexible Synthetic
Carbohydrate Receptors with Indole and Quinoline Heterocyclic Groups. Eur. J. Org. Chem..

[25] Ezzatpour S., Thakur K., Ndede K. E., Buchholz D. W., Choi A., Imbiakha B., Carter J., Onofrei D., Eaton B., Postnikova E. (2025). Broad-spectrum Synthetic Carbohydrate Receptors
(SCRs) Inhibit Viral Entry Across Multiple Virus Families. Sci. Adv..

[26] Davis A. P. (2020). Biomimetic
carbohydrate recognition. Chem. Soc. Rev..

[27] Milanesi F., Roelens S., Francesconi O. (2024). Towards Biomimetic
Recognition of
Glycans by Synthetic Receptors. ChemPlusChem.

[28] Mazik M. (2012). Recent developments
in the molecular recognition of carbohydrates by artificial receptors. RSC Adv..

[29] Zhai W., Sun X., James T. D., Fossey J. S. (2015). Boronic Acid-Based Carbohydrate Sensing. Chem. - Asian J..

[30] Carbonell C., Valles D., Wong A. M., Carlini A. S., Touve M. A., Korpanty J., Gianneschi N. C., Braunschweig A. B. (2020). Polymer
brush hypersurface photolithography. Nat. Commun..

[31] Valles D. J., Naeem Y., Carbonell C., Wong A. M., Mootoo D. R., Braunschweig A. B. (2019). Maskless
Photochemical Printing of Multiplexed Glycan
Microarrays for High-Throughput Binding Studies. ACS Biomater. Sci. Eng..

[32] Valles D. J., Zholdassov Y. S., Korpanty J., Uddin S., Naeem Y., Mootoo D. R., Gianneschi N. C., Braunschweig A. B. (2021). Glycopolymer
Microarrays with Sub-Femtomolar Avidity for Glycan Binding Proteins
Prepared by Grafted-To/Grafted-From Photopolymerizations. Angew. Chem. Int. Ed..

[33] Wong A. M., Valles D. J., Carbonell C., Chambers C. L., Rozenfeld A. Y., Aldasooky R. W., Braunschweig A. B. (2019). Controlled-Height Brush Polymer Patterns
via Surface-Initiated Thiol-Methacrylate Photopolymerizations. ACS Macro Lett..

[34] Zholdassov Y. S., Valles D. J., Uddin S., Korpanty J., Gianneschi N. C., Braunschweig A. B. (2021). Orthogonal
Images Concealed Within a Responsive 6-Dimensional
Hypersurface. Adv. Mater..

[35] Bonda L., Valles D. J., Wigger T. L., Meisner J., Braunschweig A. B., Hartmann L. T.-I. (2023). Light-Activated Controlled Radical Polymerization. Macromolecules.

[36] Bian S., Zieba S. B., Morris W., Han X., Richter D. C., Brown K. A., Mirkin C. A., Braunschweig A. B. (2014). Beam pen
lithography as a new tool for spatially controlled photochemistry,
and its utilization in the synthesis of multivalent glycan arrays. Chem. Sci..

[37] Blawitzki L. C., Bartels N., Bonda L., Schmidt S., Monzel C., Hartmann L. (2024). Glycomacromolecules
to Tailor Crowded and Heteromultivalent
Glycocalyx Mimetics. Biomacromolecules.

[38] Valles D. J., Zholdassov Y. S., Braunschweig A. B. (2021). Evolution and applications of polymer
brush hypersurface photolithography. Polym.
Chem..

[39] Wu X., Canamares M. V., Kakoulli I., Sanchez-Cortes S. (2022). Chemical Characterization
and Molecular Dynamics Simulations of Bufotenine by Surface-Enhanced
Raman Scattering (SERS) and Density Functional Theory (DFT). J. Phys. Chem. Lett..

[40] Dieng S. D., Schelvis J. P. (2010). Analysis of measured
and calculated Raman spectra of
indole, 3-methylindole, and tryptophan on the basis of observed and
predicted isotope shifts. J. Phys. Chem. A.

[41] Zholdassov Y. S., Yuan L., Garcia S. R., Kwok R. W., Boscoboinik A., Valles D. J., Marianski M., Martini A., Carpick R. W., Braunschweig A. B. (2023). Acceleration of Diels-Alder reactions by mechanical
distortion. Science.

[42] Liu X., Carbonell C., Braunschweig A. B. (2016). Towards scanning probe lithography-based
4D nanoprinting by advancing surface chemistry, nanopatterning strategies,
and characterization protocols. Chem. Soc. Rev..

[43] Neu U., Khan Z. M., Schuch B., Palma A. S., Liu Y., Pawlita M., Feizi T., Stehle T. (2013). Structures of B-lymphotropic
polyomavirus VP1 in complex with oligosaccharide ligands. PLoS Pathog..

[44] Gordus A., MacBeath G. (2006). Circumventing the problems caused
by protein diversity
in microarrays: implications for protein interaction networks. J. Am. Chem. Soc..

[45] Latour R. A. (2015). The Langmuir
isotherm: a commonly applied but misleading approach for the analysis
of protein adsorption behavior. J. Biomed. Mater.
Res., Part A.

[46] Campbell, R. M. ; Dymshitz, J. ; Eastwood, B. J. ; Emkey, R. ; Greenen, D. P. ; Heerding, J. M. ; Johnson, D. ; Large, T. H. ; Littlejohn, T. ; Montrose, C. , Data Standardization for Results Management. In Assay Guidance Manual; Bethesda, 2004.

[47] Sigurskjold B. W. (2000). Exact analysis
of competition ligand binding by displacement isothermal titration
calorimetry. Anal. Biochem..

[48] Watanabe M., Nakamura-Nakayama M., Fujihara M., Kawasaki M., Nakano S., Kakuta H. (2022). Increased
Molecular Flexibility Widens the Gap between
K (i) and K (d) values in Screening for Retinoid X Receptor Modulators. ACS Med. Chem. Lett..

[49] Sanders, C. R. Biomolecular ligand-receptor binding studies: Theory, practice, and analysis; Vanderbilt University: Nashville, 2010; pp. 1–43.

[50] Cozzini P., Fornabaio M., Marabotti A., Abraham D. J., Kellogg G. E., Mozzarelli A. (2002). Simple, intuitive
calculations of free energy of binding
for protein-ligand complexes. 1. Models without explicit constrained
water. J. Med. Chem..

[51] Wu H., Kohler J. (2019). Photocrosslinking probes
for capture of carbohydrate
interactions. Curr. Opin. Chem. Biol..

[52] Stefan M. I., Le Novere N. (2013). Cooperative
binding. PLoS Comput.
Biol..

[53] Weiss J. N. (1997). The Hill
equation revisited: uses and misuses. FASEB
J..

[54] Pracht P., Bohle F., Grimme S. (2020). Automated exploration
of the low-energy
chemical space with fast quantum chemical methods. Phys. Chem. Chem. Phys..

[55] Pracht P., Grimme S., Bannwarth C., Bohle F., Ehlert S., Feldmann G., Gorges J., Muller M., Neudecker T., Plett C. (2024). CREST—A
program for the exploration of low-energy
molecular chemical space. J. Chem. Phys..

[56] Bannwarth C., Ehlert S., Grimme S. (2019). GFN2-xTB-An Accurate and Broadly
Parametrized Self-Consistent Tight-Binding Quantum Chemical Method
with Multipole Electrostatics and Density-Dependent Dispersion Contributions. J. Chem. Theory Comput..

[57] Blum V., Gehrke R., Hanke F., Havu P., Havu V., Ren X., Reuter K., Scheffler M. (2009). Ab initio molecular simulations with
numeric atom-centered orbitals. Comput. Phys.
Commun..

[58] Perdew J. P., Burke K., Ernzerhof M. (1996). Generalized
Gradient Approximation
Made Simple. Phys. Rev. Lett..

[59] Tkatchenko A., Scheffler M. (2009). Accurate molecular van der Waals interactions from
ground-state electron density and free-atom reference data. Phys. Rev. Lett..

[60] Perdew J. P., Ernzerhof M., Burke K. (1996). Rationale for mixing exact exchange
with density functional approximations. J. Chem.
Phys..

[61] Tomasi J., Mennucci B., Cammi R. (2005). Quantum Mechanical Continuum Solvation
Models. Chem. Rev..

[62] Dang K., Zhang W., Jiang S., Lin X., Qian A. (2020). Application
of Lectin Microarrays for Biomarker Discovery. ChemistryOpen.

[63] Hiono T., Matsuda A., Wagatsuma T., Okamatsu M., Sakoda Y., Kuno A. (2019). Lectin microarray analyses reveal host cell-specific glycan profiles
of the hemagglutinins of influenza A viruses. Virology.

[64] Hirabayashi J., Yamada M., Kuno A., Tateno H. (2013). Lectin microarrays:
concept, principle and applications. Chem. Soc.
Rev..

[65] Katrlik J., Svitel J., Gemeiner P., Kozar T., Tkac J. (2010). Glycan and
lectin microarrays for glycomics and medicinal applications. Med. Res. Rev..

[66] Singh A., Arango J. C., Shi A., d’Aliberti J. B., Claridge S. A. (2023). Surface-Templated Glycopolymer Nanopatterns
Transferred
to Hydrogels for Designed Multivalent Carbohydrate–Lectin Interactions
across Length Scales. J. Am. Chem. Soc..

[67] Zhou S.-M., Cheng L., Guo S.-J., Wang Y., Czajkowsky D. M., Gao H., Hu X.-F., Tao S.-C. (2015). Lectin RCA-I specifically binds to
metastasis-associated cell surface glycans in triple-negative breast
cancer. Breast Cancer Res..

[68] Sun H., Chen G. Y., Yao S. Q. (2013). Recent advances in microarray technologies
for proteomics. Chem. Biol..

[69] Uttamchandani M., Wang J., Yao S. Q. (2006). Protein
and small molecule microarrays:
powerful tools for high-throughput proteomics. Mol. BioSyst..

