# Supplementary material for: Monosaccharide Binding to Synthetic Carbohydrate Receptor Microarrays
Source: J Phys Chem C Nanomater Interfaces. 2025 Nov 6;129(46):20797–808. doi: 10.1021/acs.jpcc.5c06186 (PMC12641478; doi:10.1021/acs.jpcc.5c06186)
Supplement: Supplementary file 1 [file jp5c06186_si_001.pdf]

Supporting Information

Monosaccharide Binding to Synthetic Carbohydrate Receptor Microarrays

Milan A. Shlain[1,2,3], Kenneth Erzoah Ndede[1,2,3], Khushabu Thakur[1,2], Anthony J. Russo[1,5], Siddharth Pasari[1,5], Ishraq Nihal[1,2], Keidy L. Matos[1,2,3], Yerzhan S. Zholdassov[1,2,3], Mateusz Marianski[1,2,3,4], Adam B. Braunschweig*[1,2,3,4]

[1]Advanced Science Research Center, Graduate Center, City University of New York, 85 St. Nicholas Terrace, New York, NY 10031, USA

[2]Department of Chemistry, Hunter College, 695 Park Avenue, New York, NY 10065, USA

[3]PhD Program in Chemistry, Graduate Center, City University of New York, 365 5th Avenue, New York, NY 10016, USA

[4]PhD Program in Biochemistry, Graduate Center, City University of New York, 365 5th Avenue, New York, NY 10016, USA

[5]Hunter College High School, 71 E 94th St, New York, NY 10128, USA

Email: abraunschweig@gc.cuny.edu

Table of Contents

1. **Organic synthesis** S3
2. **2D NMR analysis** S43
3. **Preparation of thiol-terminated monolayers** S52
4. **Description of the printer**. S54
5. **Printing optimization of SCR043 functionalized polymer brushes** S56
6. **Chemical characterization of polymer brush surfaces** S80
7. **Binding Studies** S84
8. **Determination of K_ds** S200
9. **Determination of Hill coefficients (H_c)** S206
10. **Python code for profilometry and fluorescence data processing** S215
11. **Statistical validation** S216
12. **Computational Analysis** S218
13. **References** S220

1. Organic synthesis

General synthetic methods. Unless otherwise stated, all reagents, starting materials, and solvents were purchased from commercial sources and used as received. Solvents were purified using an MBraun solvent purification system, and all chromatography purification was performed on silica gel (60 Å, 70-230 mesh). Thin-layer chromatography (TLC) was carried out on pre-coated aluminum sheets with silica gel 60 (EMD 40-60 mm, 230-400 mesh with 254 nm dye) and visualized using UV-light for **SCR043** and Ce_2SO_4 stain for **2**, **3**, **α-Man-FL**, **5**, **6**, **α-Gal-FL**, **8**, **9**, **β-Gal-FL**, **11**, **12**, **α-Gluc-FL**, **14**, **15**, **β-Gluc-FL**, respectively. All reactions were conducted under an inert atmosphere of Ar using standard Schlenk techniques, unless otherwise indicated. Prior to use, reaction flasks were dried in an oven at 115 °C for 12 h. **SCR019**[1] ,**1**[2], **4**[2], **7**[2], **10**[2] and **13**[2] were synthesized following previously published literature procedures. Deuterated solvents were obtained from Cambridge Isotope Laboratories Inc. and used without further purification. NMR spectra were recorded on a Bruker AVANCE 300 MHz spectrometer, with the solvent residual signal used as an internal standard. Chemical shifts were reported in *δ* units as ppm, and signal multiplicities were abbreviated as follows: *s*, singlet; *d*, doublet; *t*, triplet; *m*, multiplet; *dd*, doublet of doublets. High-resolution electrospray ionization mass spectra were acquired using an Agilent Q-TOF system. Fluorescence emission spectra were acquired using a Shimadzu Spectrofluorophotometer RF-5301PC, while UV-Vis absorption spectra were obtained using a Shimadzu UV-1800 Spectrophotometer.

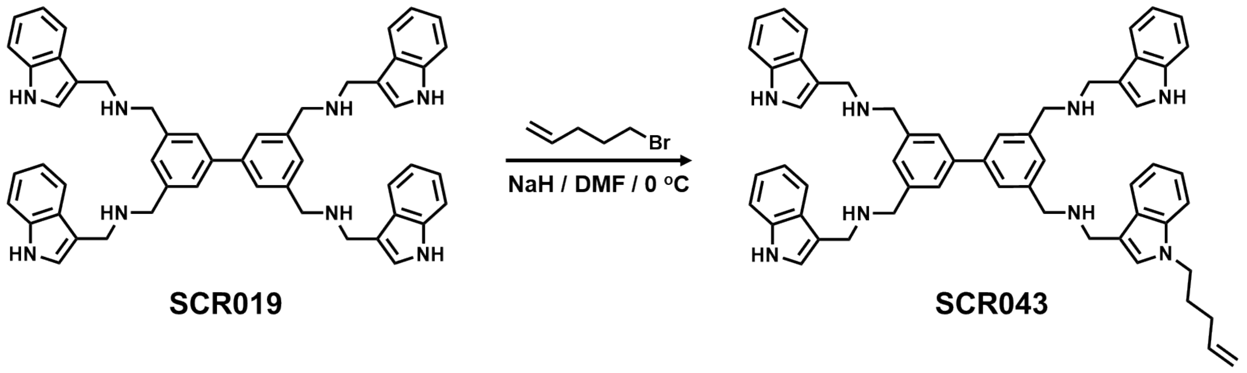

SCR019 **SCR043**

Scheme S1. Synthesis of **SCR043**.

Synthesis of SCR043. NaH (29 mg, 1.2 mmol) was added to 2 mL of DMF in a Schlenk flask under Ar. The flask was cooled to 0 °C over ice. **SCR019** (1.2 g, 1.5 mmol in DMF, 50 mL) was added dropwise over 5 min. The reaction mixture was stirred at 0 °C for another 15 min and then bromopentene (0.18 g, 1.2 mmol, 0.15 mL) dissolved in DMF (50 mL) was added dropwise to the reaction mixture, which was then stirred at room temperature for 16 h. The reaction mixture was quenched with H_2O (10 mL) and extracted with CH_2Cl_2 (50 mL x 3). Organic layers were collected and dried over Na_2SO_4 (anhydrous) and then evaporated under reduced pressure. The mixture was purified using column chromatography (SiO_2 $CHCl_3$: MeOH : NH_3 :: 9:1:1) to provide **SCR043** as a pale-yellow solid (31%, 0.30 g). ^{1}H NMR (300 MHz, $CDCl_3$) δ = 8.29 (3H, *s*), 7.78-7.55 (4H, *m*), 7.44 (4H, *t*, *J* = 10.22 Hz), 7.28 (4H, *s*), 7.23-6.97 (14H, *m*), 5.86-5.62 (1H, *m*), 5.10-4.78 (2H, *m*), 4.11-3.72 (18H, *m*), 2.14-1.75 (4H, *m*); ^{13}C NMR (75 MHz, $CDCl_3$) δ =141.15, 140.49, 137.37, 136.35, 127.91, 127.64, 127.04, 126.64, 125.91, 123.39, 121.65, 119.32, 119.09, 118.66, 115.58, 113.49, 111.43, 109.61, 53.51, 45.41, 43.89, 30.82, 29.16; m/z calcd for $C_{57}H_{58}N_8$ $[M+H]^+$: 855.4858, found 855.4850.

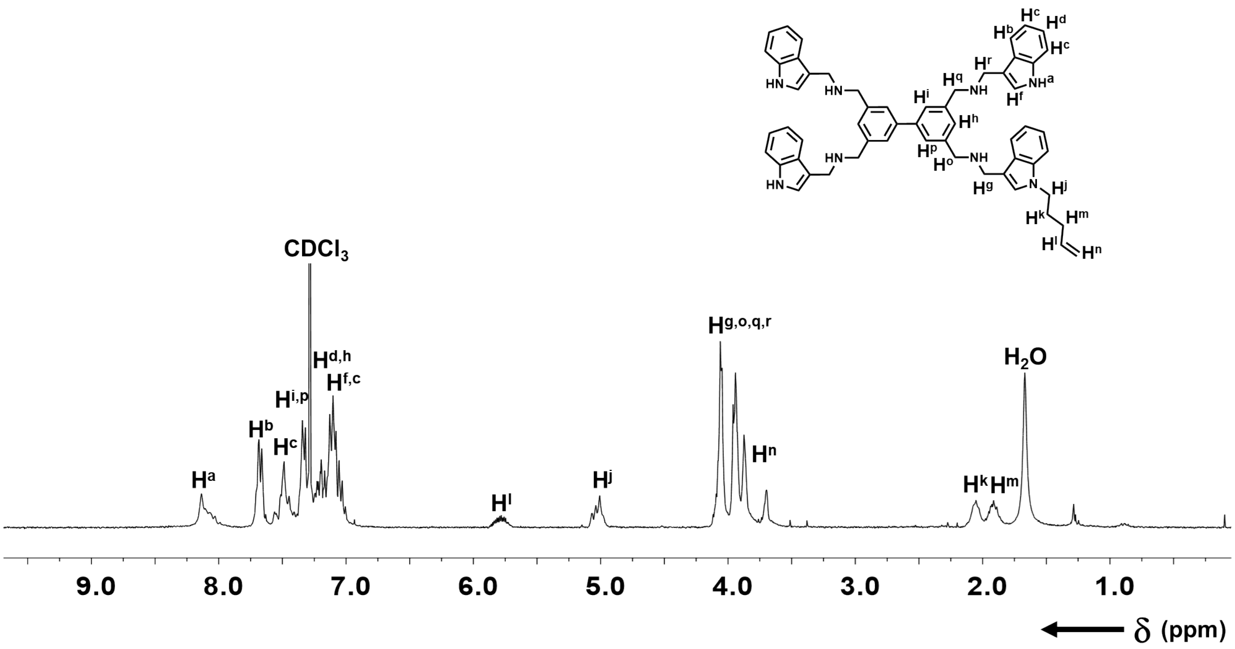

Figure S1. ^{1}H NMR of **SCR043** (300 MHz) 25 ^{o}C in $CDCl_3$.

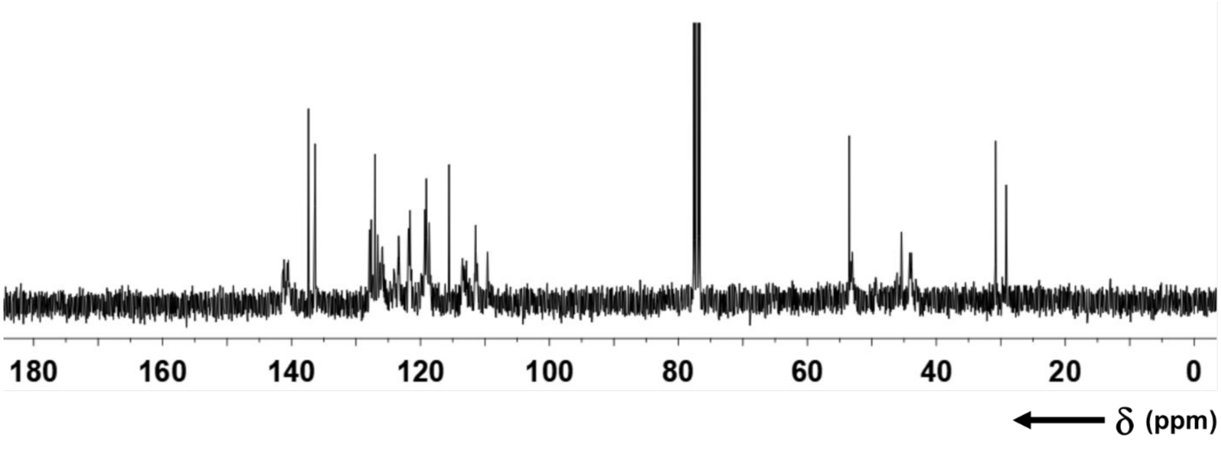

Figure S2. ^{13}C NMR of **SCR043** (75 MHz) 25 ^{o}C in $CDCl_3$.

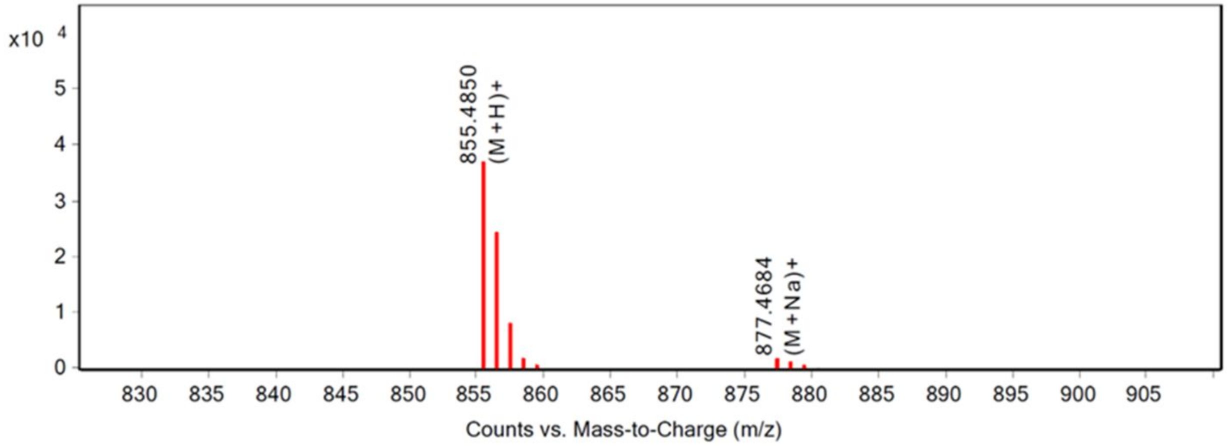

Figure S3. HR-MS of **SCR043**.

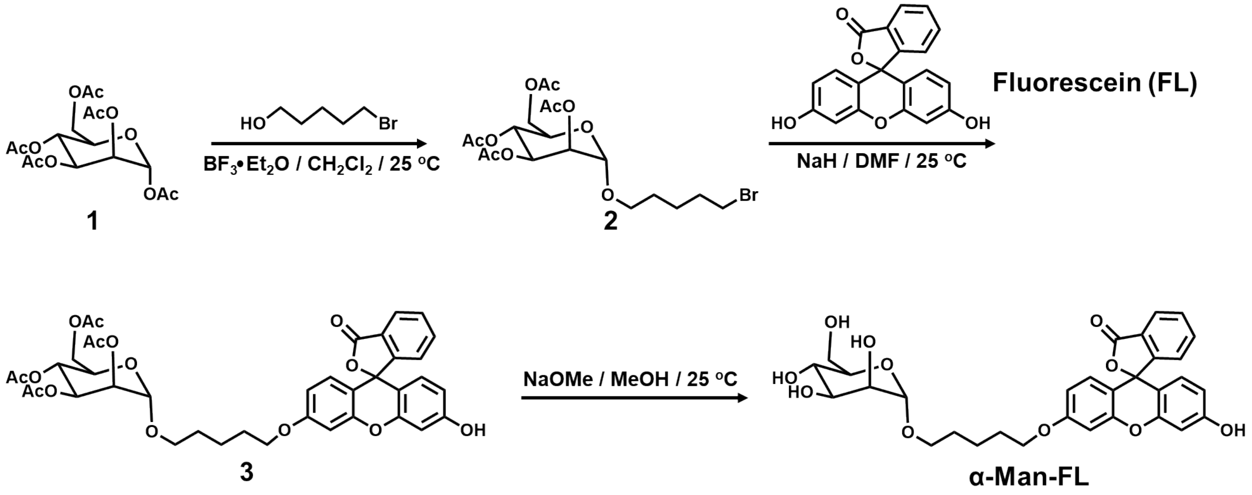

Scheme S2. Synthesis of **α-Man-FL**.

Synthesis of **(*2R,3R,4S,5S,6S*)-2-(acetoxymethyl)-6-((5-bromopentyl)oxy)tetrahydro-2*H*-pyran-3,4,5-triyl triacetate** (**2**). To a solution of **1** (5.0 g, 13 mmol) in anhydrous CH_2Cl_2 (80 mL) was added 5-bromo penten-1-ol (4.3 g, 26 mmol) dropwise. The mixture was cooled to –20 °C and treated with BF_3•Et_2O (21.0 g, 148 mmol) dropwise. After 30 min, the reaction mixture was warmed to room temperature and then stirred for 16 h. The mixture was then washed with saturated $NaHCO_3$(aq) (3 x 50 mL). The organic layer was dried over Na_2SO_4 (anhydrous) and concentrated under reduced pressure. The mixture

was purified using column chromatography (SiO_2 EtOAc: Hexanes:: 2:8) to provide **2** as a clear viscous liquid (40%, 2.0 g). ^1H NMR (300 MHz, $CDCl_3$) δ = 5.37-5.22 (1H, *m*), 5.28-5.21 (2H, *m*) 4.81 (1H, *d*, *J* = 0.85 Hz), 4.28 (1H, *dd*, *J* = 6.69, 5.36Hz), 4.10 (1H, *dd*, *J* = 12.21, 2.45 Hz), 4.02-3.94 (1H, *m*), 3.74-3.64 (1H, *m*), 3.51-3.39 (3H, *m*) 2.15 (3H, *s*), 2.10 (3H, *s*), 2.05 (3H, *s*), 1.99 (3H, *s*), 1.95-1.83 (2H, *m*), 1.70-1.58 (2H, *m*), 1.58-1.46 (2H, *m*); ^{13}C NMR (75 MHz, $CDCl_3$) δ = 170.55, 170.01, 169.84, 169.68, 97.54, 72.82, 69.61, 69.06, 68.45, 68.04, 66.19, 62.51, 33.48, 32.34, 28.38, 24.70, 20.87, 20.72, 20.66; m/z calcd for $C_{19}H_{29}BrO_{10}$ $[M+H]^+$: 499.0996, found 499.0997.

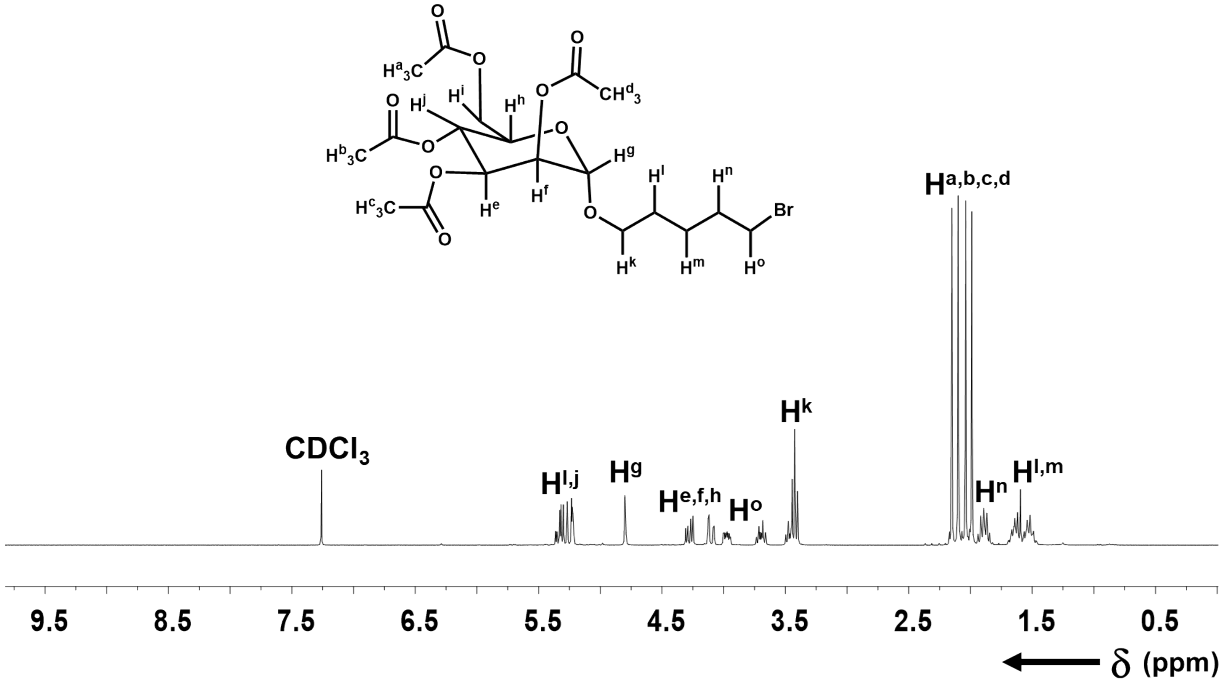

Figure S4. ^1H NMR of **2** (300 MHz), 25 ^oC in $CDCl_3$.

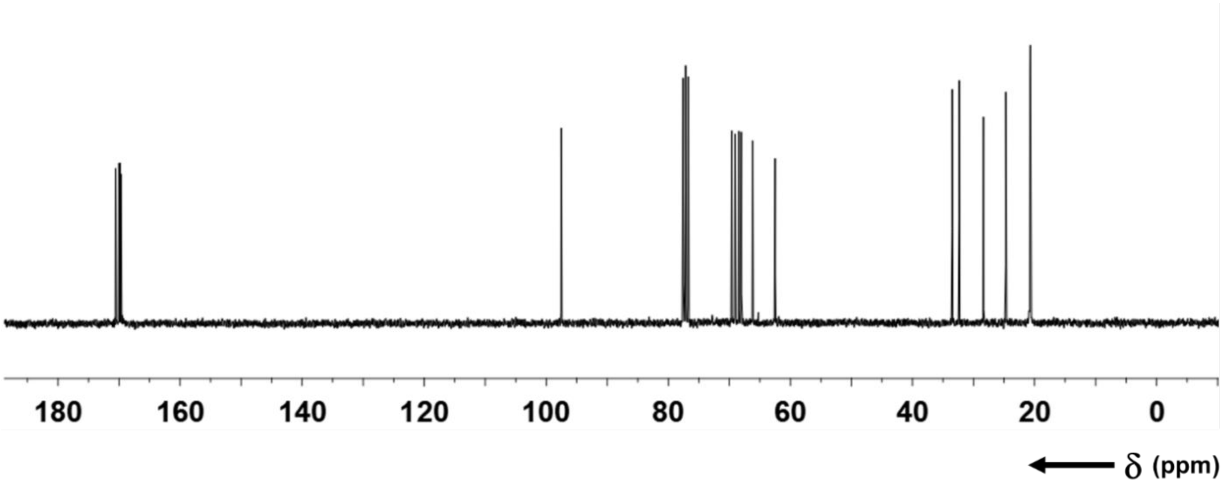

Figure S5. ^{13}C NMR of **2** (75 MHz) 25 °C in $CDCl_3$.

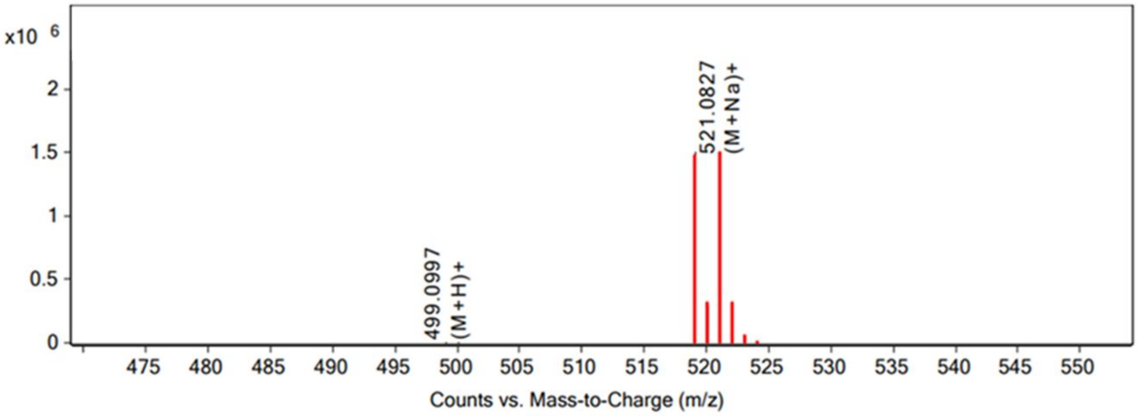

Figure S6. HR-MS of **2**.

Synthesis of **(*2R,3R,4S,5S,6S*)-2-(acetoxymethyl)-6-((5-((3'-hydroxy-3-oxo-3*H*-spiro[isobenzofuran-1,9'-xanthen]-6'-yl)oxy)pentyl)oxy)tetrahydro-2*H*-pyran-3,4,5-triyl triacetate (3)**. **2** (0.60 g, 1.2 mmol, 1.0 eq) was dissolved in DMF (30 mL) and stirred. To the solution was added fluorescein (0.40 g, 1.2 mmol, 1.0 eq). The reaction was then cooled to 0 °C, and NaH (0.040 g, 1.8 mmol, 1.5 eq) was added. The reaction was left at 0 °C for 30 min then warmed to room temperature and stirred for 16 h. DMF was removed under reduced pressure. The mixture was purified by column chromatography (SiO_2 EtOAc : Hexanes :: 5:5) to provide **3** as a red powder (38%, 230 mg). ^1H NMR (300

MHz, $CDCl_3$) δ = 8.24 (1H,*dd*, *J* = 7.5, 1.4 Hz), 7.77-7.61 (3H, *m*), 7.29 (1H, *dd*, *J* = 8.85, 2.45 Hz), 6.97 (2H, *d*, *J* = 9.2 Hz), 6.89-6.73 (3H, *m*), 5.37-5.16 (3H, *m*), 4.76 (1H, *d*, *J* = 1.1 Hz), 4.26 (1H, *dd*, *J* = 12.24, 5.07 Hz), 4.08 (2H, *dd*, *J* = 12.17, 2,19 Hz), 3.96 (4H, *t*, *J* = 6.2 Hz), 3.65-3.55 (1H, *m*), 3.38-3.27 (1H, *m*), 2.11 (3H, *s*), 2.06 (3H, *s*), 2.01 (3H, *s*), 1.97 (3H, *s*), 1.58-1.42 (2H, *m*), 1.42-1.29 (2H, *m*), 1.21-1.06 (2H, *m*); ^{13}C NMR (75 MHz, $CDCl_3$) δ = 175.41, 170.89, 170.34, 170.20, 169.90, 165.44, 157.76, 155.51, 134.21, 132.68, 131.34, 131.03, 130.68, 130.58, 129.99, 128.92, 122.12, 115.12, 103.89, 97.58, 69.76, 69.33, 68.51, 68.12, 66.28, 66.00, 65.53, 62.61, 60.53, 31.03, 29.78, 28.90, 28.12, 22.58, 21.15, 21.02, 20.86, 20.82, 14.29; m/z calcd for $C_{39}H_{40}O_{15}$ $[M+H]^+$: 749.2440, found 749.2442.

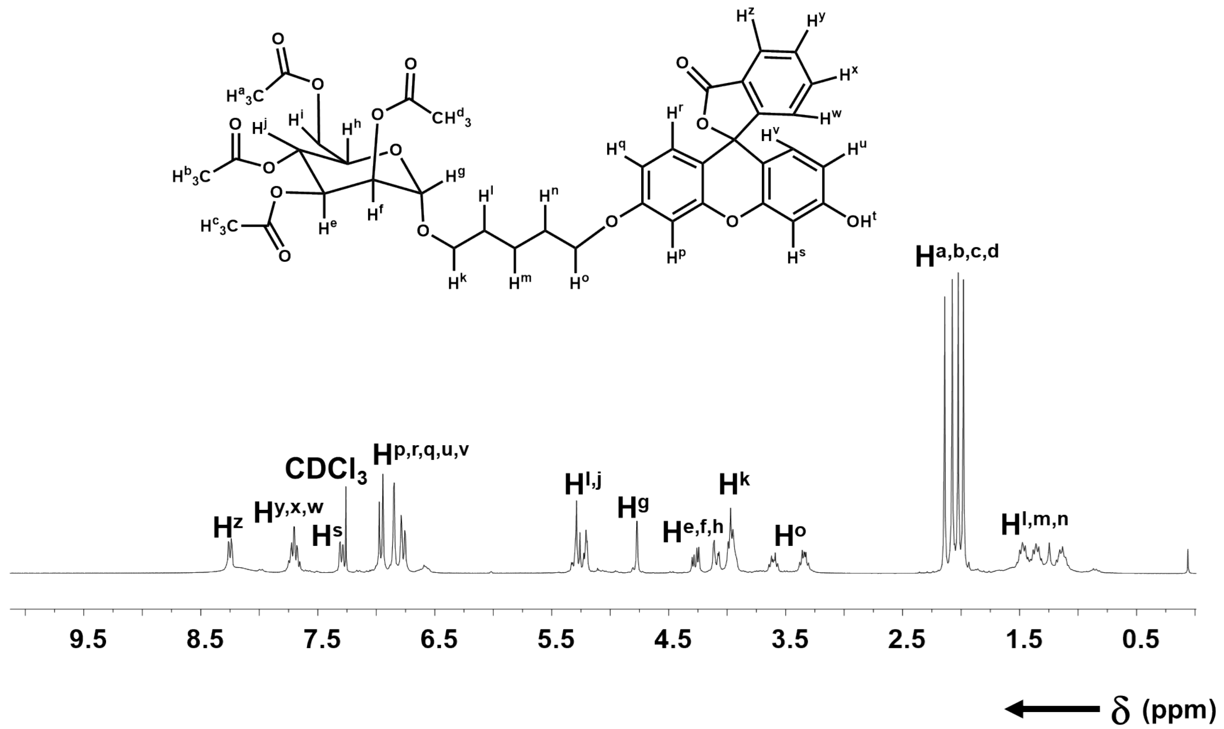

Figure S7. ^1H NMR of **3** (300 MHz) 25 °C in $CDCl_3$.

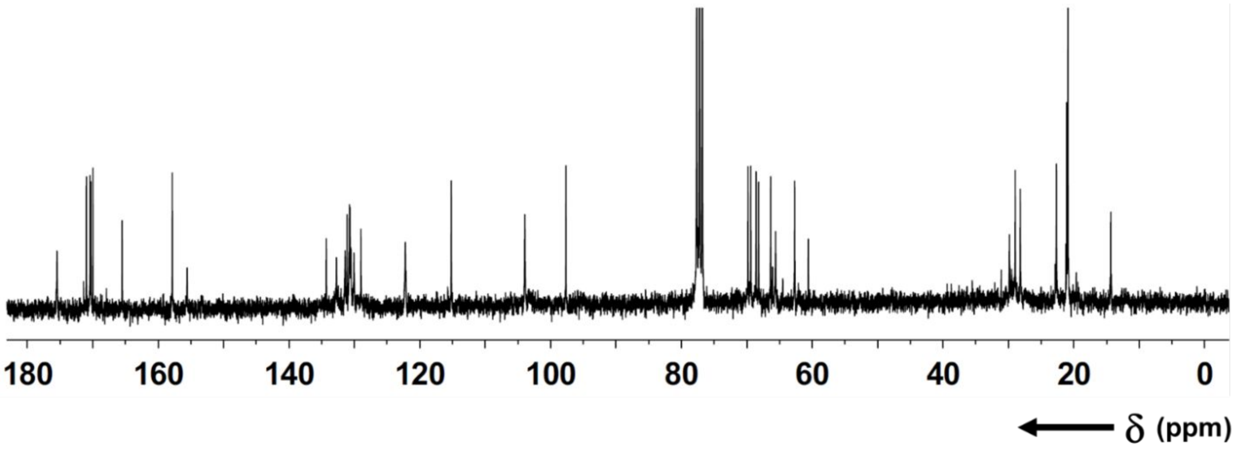

Figure S8. ^{13}C NMR of **3** (75 MHz) 25 ^{o}C in $CDCl_3$.

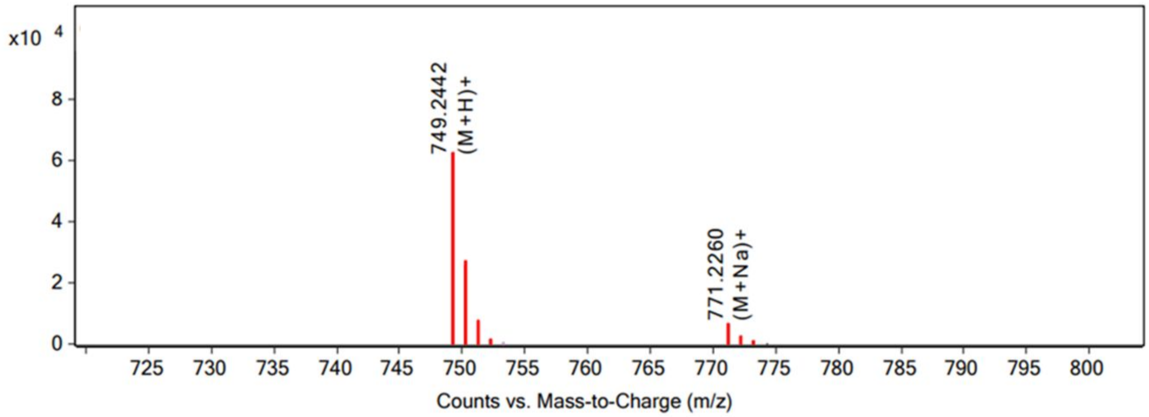

Figure S9. HR-MS of **3**.

Synthesis of **α-Man-FL. 3** (0.20 g, 0.30 mmol, 1.0 eq) was dissolved in MeOH (30 mL) and cooled to 0 °C. After 30 min, NaOMe (0.10 g, 1.6 mmol, 5.3 eq) was added, and the mixture was stirred for 1 h. 1M HCl(aq) was added until the solution reached a pH of 6. The reaction mixture was then concentrated under reduced pressure and purified by column chromatography (SiO_2 H_2O : MeOH: EtOAc :: 1:1:8) to provide **α-Man-FL** as a red powder (66%, 150 mg). ^{1}H NMR (300 MHz, MeOD) δ = 8.27 (1H, *dd, J* = 7.7, 1.3 Hz), 7.89-7.7.74 (3H, *m*), 7.42 (1H, *dd, J* = 7.37, 1.10 Hz), 6.97 (2H, *d, J* = 9.10 Hz), 6.66 (4H, *dd, J* = 11.60, 2.25 Hz), 4.74 (1H, *d, J* = 1.4 Hz), 3.99-3.35 (12H, *m*), 3.31-3.24 (1H, *m*),

1.47-1.34 (2H, *m*), 1.28-1.18 (2H, *m*), 1.08-0.93 (2H, *m*); ^{13}C NMR (75 MHz, MeOD) δ = 178.98, 167.24, 159.35, 156.85, 155.47, 135.13, 133.80, 132.18, 131.95, 131.78, 131.63, 131.07, 130.78, 123.43, 114.98, 104.51, 103.67, 101.46, 79.47, 74.40, 72.44, 72.09, 68.45, 68.26, 66.62, 62.49, 54.82, 30.10, 29.11, 23.64, 22.10; m/z calcd for $C_{31}H_{32}O_{11}$ $[M+H]^+$: 581.2017, found 581.2014; $\lambda_{max,abs}$ = 495 nm, ε = 3500 cm^{-1}M^{-1}, $\lambda_{max,em}$ = 518 nm.

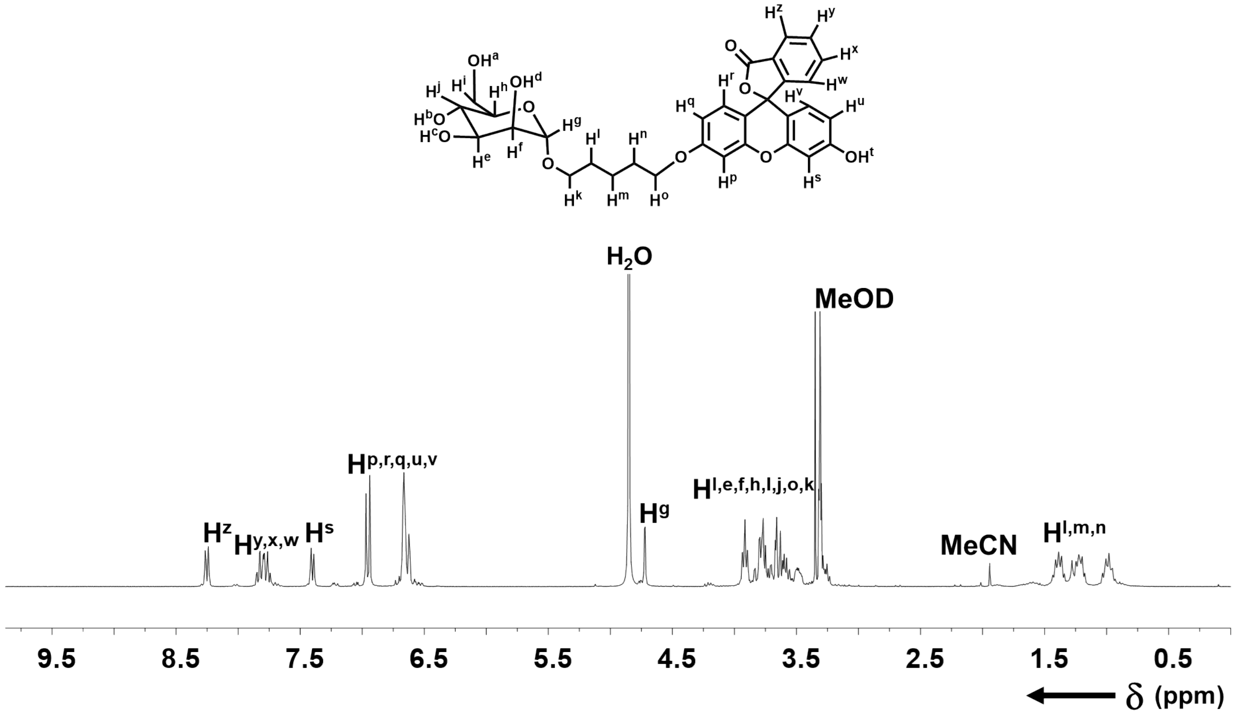

Figure S10. ^{1}H NMR of **α-Man-FL** (300 MHz), 25 ^{o}C in MeOD.

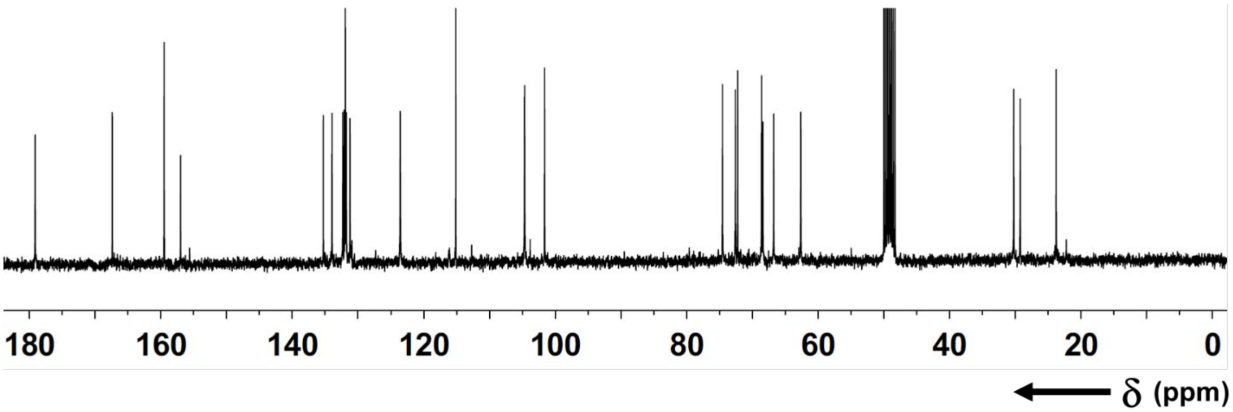

Figure S11. ^{13}C NMR of **α-Man-FL** (75 MHz), 25 ^{o}C in MeOD.

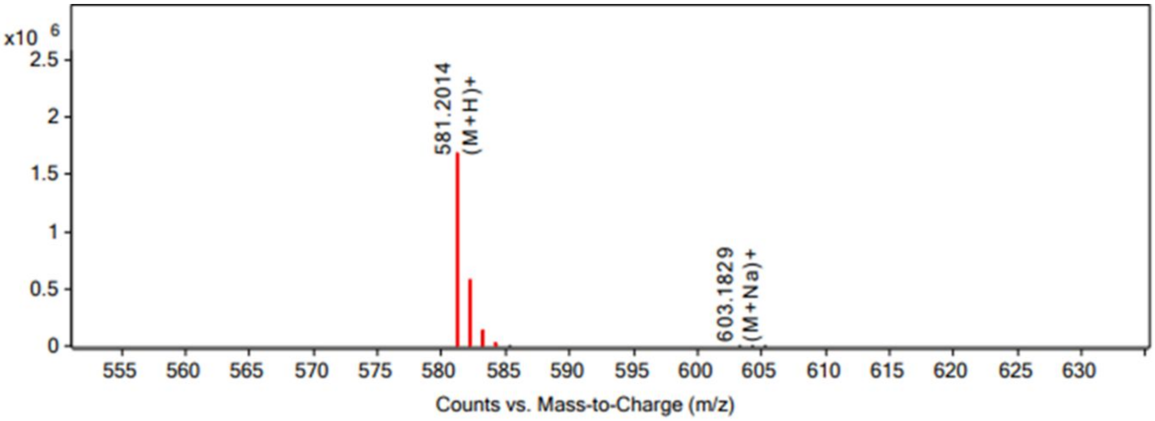

Figure S12. HR-MS of **α-Man-FL**.

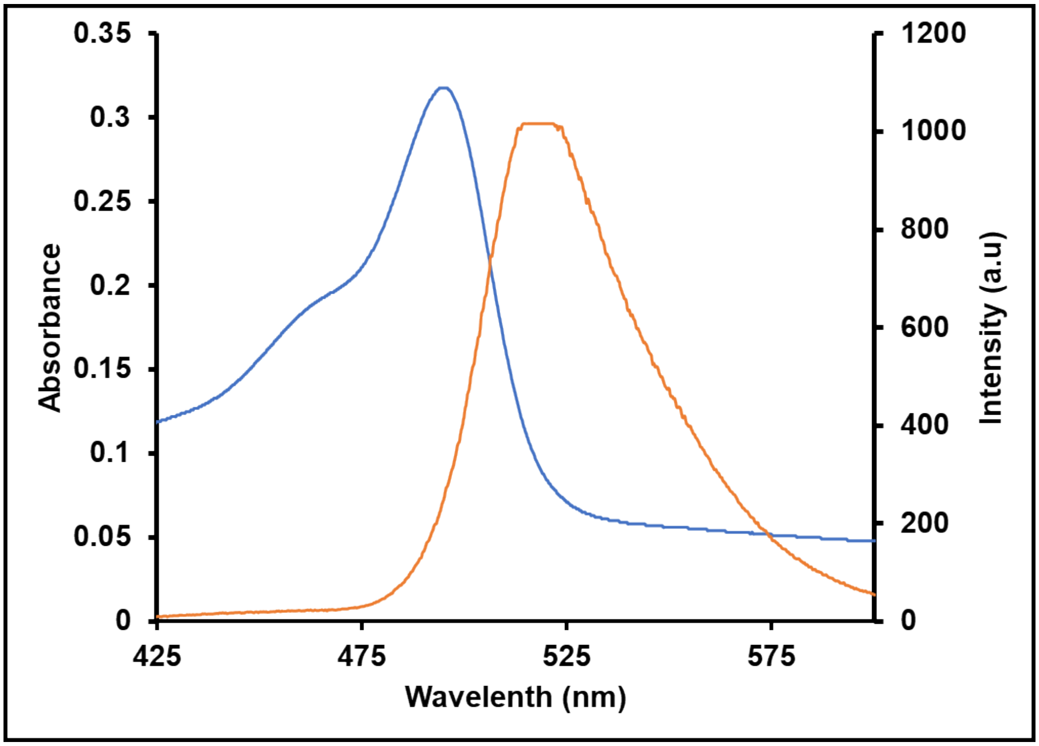

Figure S13. Absorption (blue) and fluorescence emission (orange) spectra (λ_{ex} = 425 nm) of **α-Man-FL**. Fluorophore was dissolved in a solution of Tris buffer 20 mM, pH = 7.4, 0.01% Tween20.

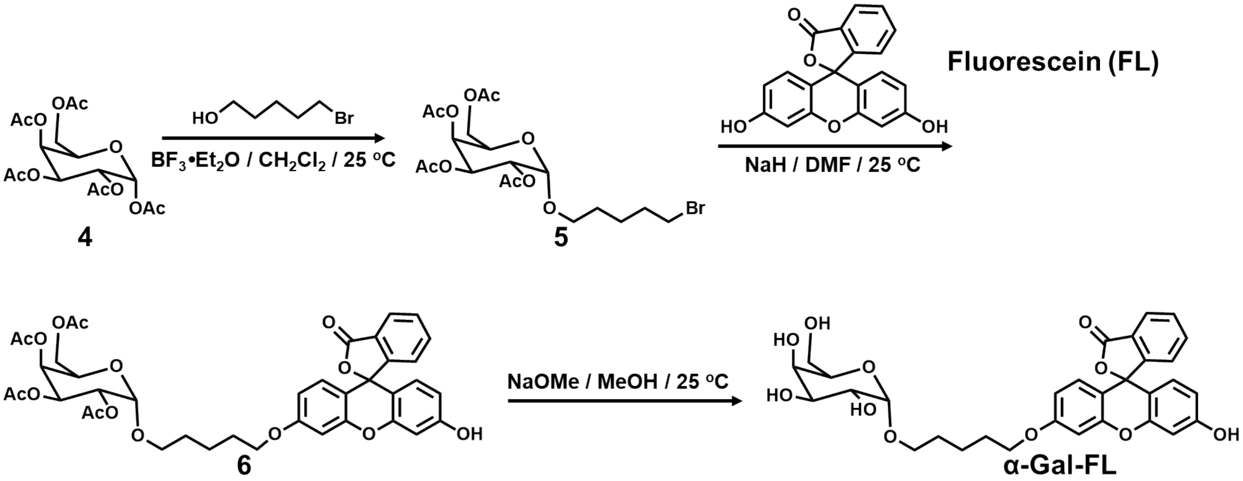

Scheme S3. Synthesis of **α-Gal-FL**

Synthesis of **(*2R,3S,4S,5R,6S*)-2-(acetoxymethyl)-6-((5-bromopentyl)oxy)tetrahydro-2*H*-pyran-3,4,5-triyl triacetate (5).** To a solution of **4** (5.0 g, 13 mmol) in anhydrous CH_2Cl_2 (80 mL) was added 5-bromo penten-1-ol (4.3 g, 26 mmol) dropwise. The mixture was cooled to –20 °C and treated with BF_3•Et_2O (21.0 g, 148 mmol) dropwise. After 30 min, the reaction mixture was warmed to room temperature and then stirred for 16 h. The mixture was then washed with saturated $NaHCO_3$(aq) (3 x 50 mL). The organic layer was dried over Na_2SO_4 (anhydrous) and concentrated under reduced pressure, which revealed the reaction produced both anomers in an α:β of approximately 50:50. The α-isomer & β-isomers were isolated in ~90% purity (by NMR analysis) using column chromatography (SiO_2 EtOAc : Hexanes :: 2:8) to provide **5** as a clear viscous liquid (13.0%,1.22 g). ^{1}H NMR (300 MHz, $CDCl_3$) δ = 5.40 (1H, *dd*, *J* = 3.30, 1.06 Hz), 5.35-5.25 (1H, *m*), 5.09-5.02 (2H, *m*), 4.17 (1H, *dd*, *J* = 10.05, 3.85 Hz), 4.11-4.01 (2H, *m*), 3.71-3.56 (2H, *m*), 3.48-3.32 (2H, *m*), 2.09 (3H, *s*), 2.02 (3H, *s*), 2.00 (3H, *s*), 1.93 (3H, *s*), 1.90-1.78 (2H, *m*), 1.64-13 (4H, *m*) ; ^{13}C NMR (75 MHz, $CDCl_3$) δ =169.95, 169.85, 169.59, 169.30, 95.86, 67.93, 67.82, 67.34, 65.96, 61.50, 33.32, 32.06, 31.53, 28.16, 24.52, 20.46, 20.37, 20.32, 20.30; m/z calcd for $C_{19}H_{29}BrO_{10}$ $[M+H]^+$: 499.0996, found 499.0988.

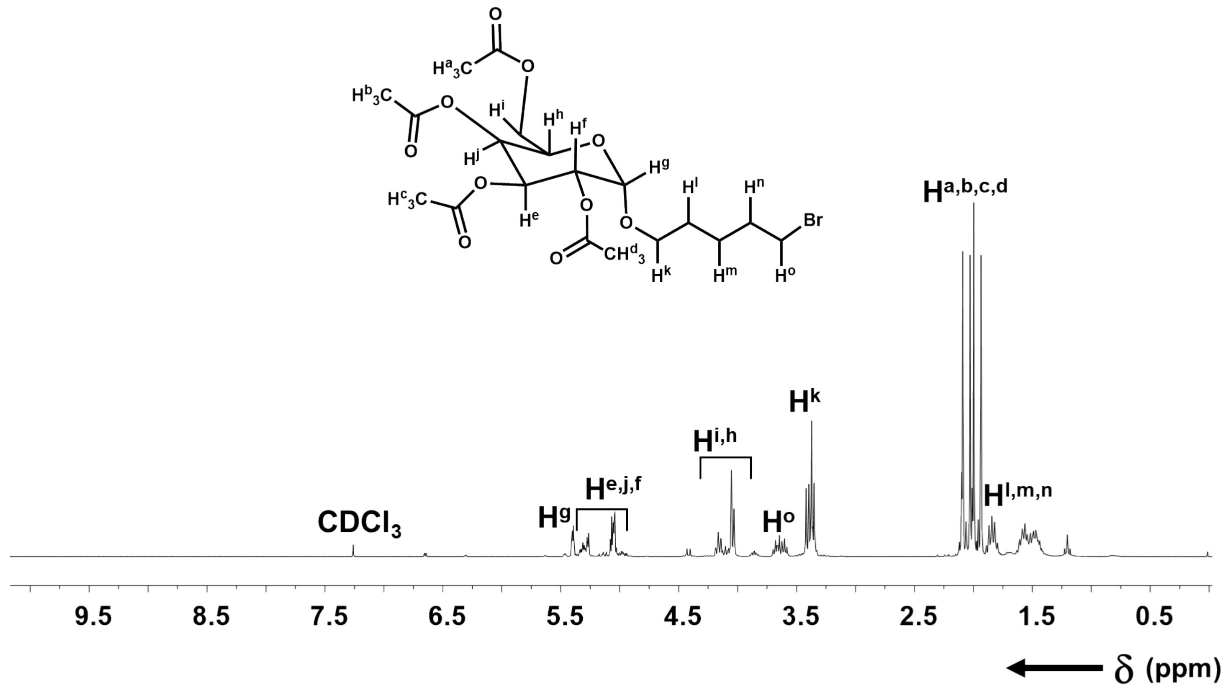

Figure S14. ^{1}H NMR of **5** (300 MHz), 25 ^{o}C in $CDCl_3$.

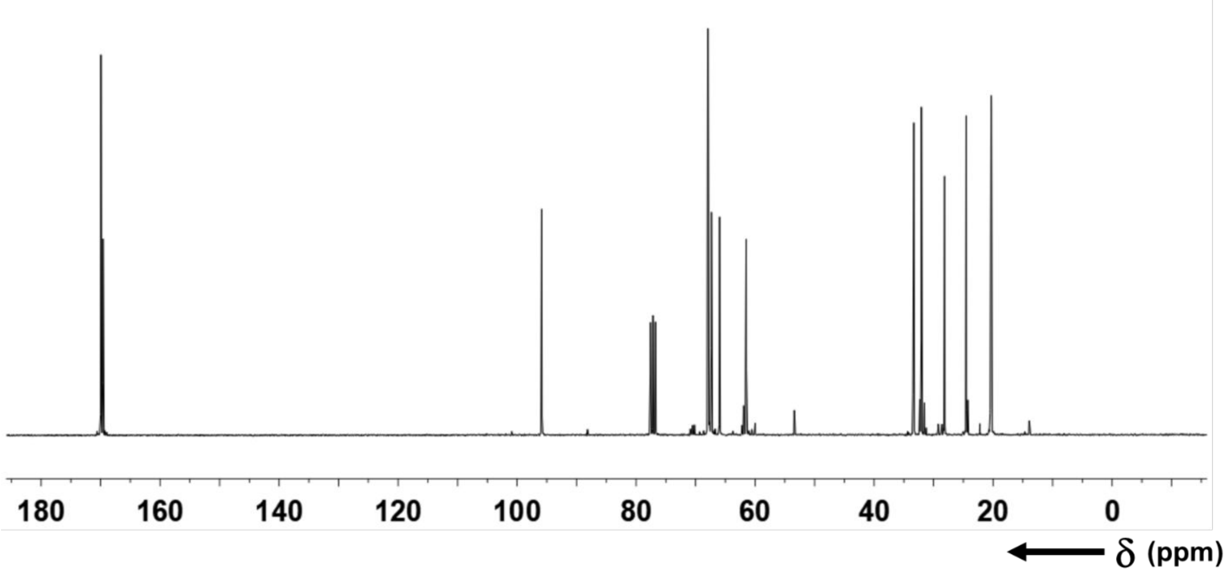

Figure S15. ^{13}C NMR of **5** (75 MHz), 25 ^{o}C in $CDCl_3$.

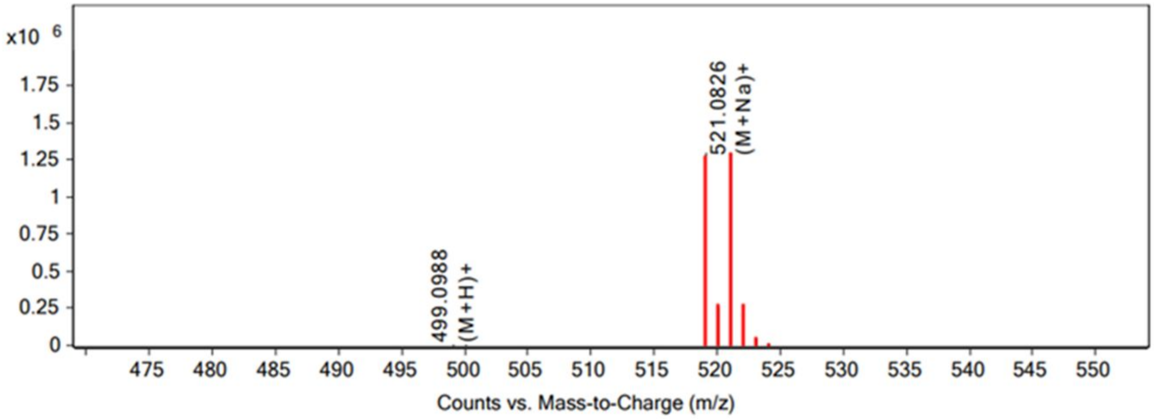

Figure S16. HR-MS of **5**.

Synthesis of **(*2R,3S,4S,5R,6S*)-2-(acetoxymethyl)-6-((5-((3'-hydroxy-3-oxo-3*H*-spiro[isobenzofuran-1,9'-xanthen]-6'-yl)oxy)pentyl)oxy)tetrahydro-2*H*-pyran-3,4,5-triyl triacetate (6)**. **5** (1.2 g, 2.5 mmol, 1.0 eq) was dissolved in DMF (30 mL) and stirred. To the solution was added fluorescein (0.80 g, 2.5 mmol, 1.0 eq). The reaction was then cooled to 0 °C, and NaH (0.1 g, 3.7 mmol, 1.5 eq) was added. The reaction was left at 0 °C for 30 min then warmed to room temperature and stirred for 16 h. DMF was removed under reduced pressure. The mixture was isolated by column chromatography (SiO_2 EtOAc : Hexanes :: 5:5) to provide **6** as a red powder (13%, 0.26 g). ^{1}H NMR (300 MHz, $CDCl_3$) δ = 8.24 (1H, *d, J* = 7.33 Hz), 7.79-7.61 (2H, *m*), 7.29 (1H, *d, J* = 7.11 Hz), 7.03-6.70(6H, *m*), 5.42 (1H, *d, J* = 2.77 Hz), 5.29 (1H, *dd, J* = 10.10, 3.30 Hz), 5.07 (2H, *dd, J* = 13.88, 3.44 Hz), 4.17 (1H, *t, J* = 6.5 Hz), 4.02 (2H, *d, J = 6.4 Hz*), 3.95 (2H, *t, J* = 6.2 Hz), 3.66-3.52 (1H, *m*), 3.38-3.24 (2H, *m*), 2.11 (3H, *s*), 2.00 (6H, *d, J* = 5.34 Hz), 1.95 (3H, *s*), 1.52-1.41(2H, *m*), 1.39-1.29 (2H, *m*), 1.19-1.05 (2H, *m*); ^{13}C NMR (75 MHz, $CDCl_3$) δ = 175.50, 170.58, 170.48, 170.37, 170.20, 165.36, 157.79, 155.54, 134.25, 132.67, 131.27, 130.63, 130.56, 130.45, 129.95, 122.12, 115.03, 103.86, 96.20, 77.36, 68.28, 68.17, 67.76, 66.21, 65.50, 61.82, 56.05, 29.75, 28.91, 28.06, 22.75, 22.52, 21.12, 20.84, 20.78, 20.76, 20.72, 14.26, 14.19; m/z calcd for $C_{39}H_{40}O_{15}$ $[M+H]^+$: 749.2440, found : 749.2440.

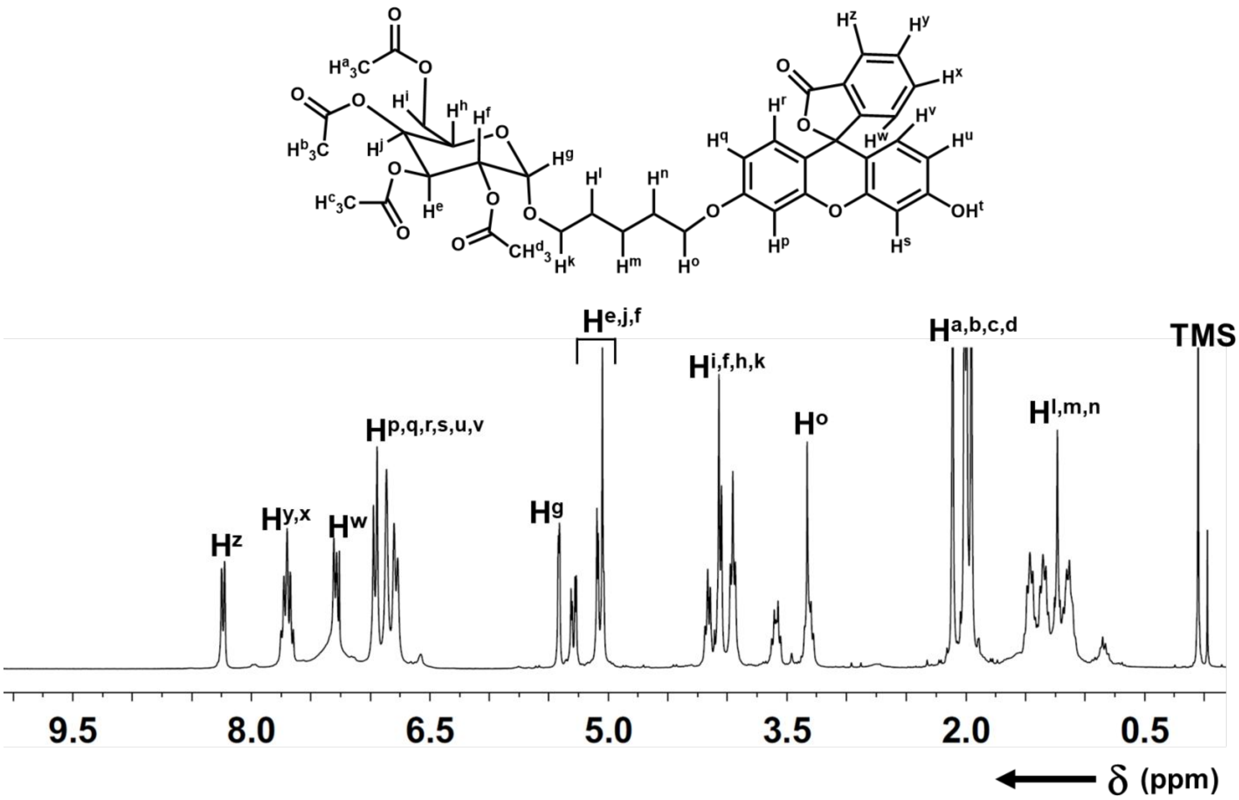

Figure S17. ^{1}H NMR of **6** (300 MHz), 25 ^{o}C in $CDCl_3$.

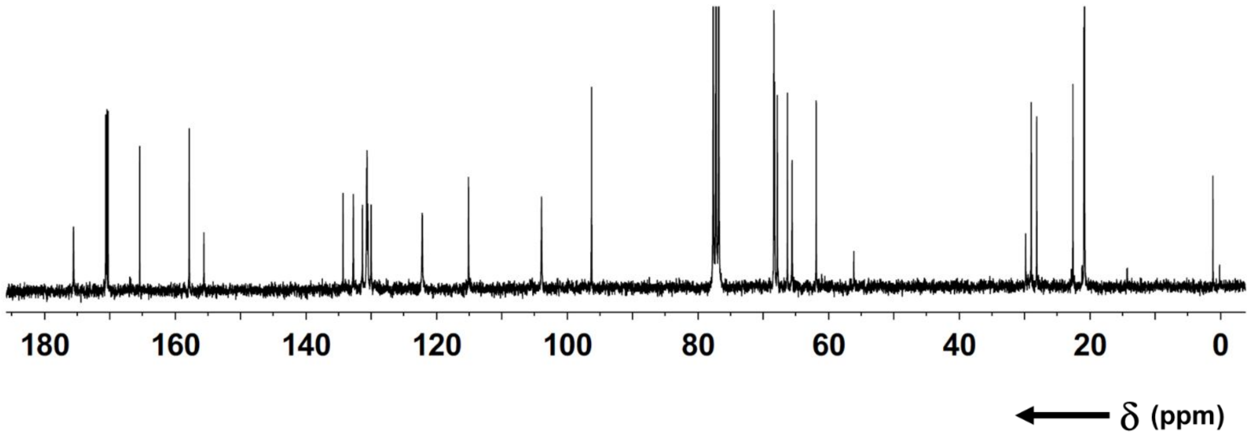

Figure S18*.* ^{13}C NMR of **6** (75 MHz), 25 ^{o}C in $CDCl_3$.

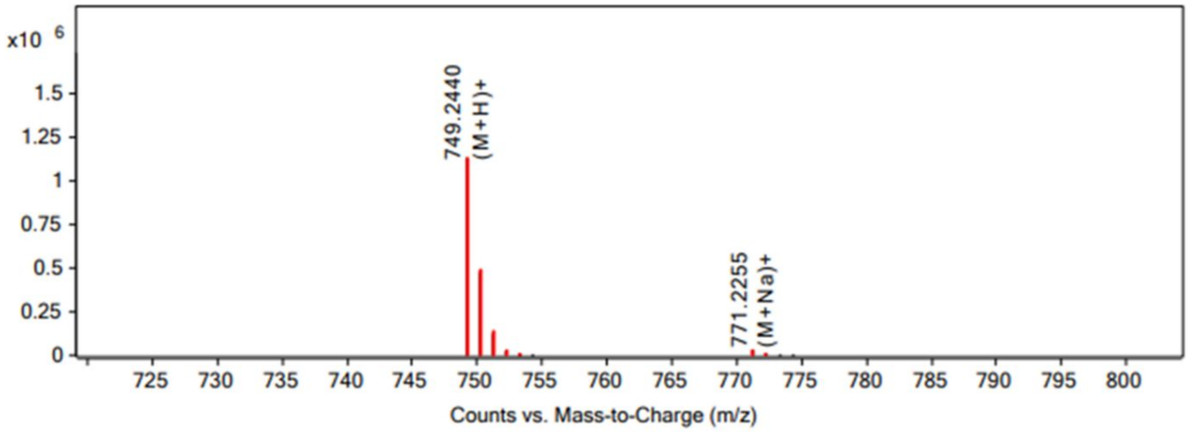

Figure S19. HR-MS of **6**.

Synthesis of **α-Gal-FL. 6** (0.3 g, 0.4 mmol, 1.0 eq) was dissolved in MeOH (30 mL) and cooled to 0 °C. After 30 min, NaOMe (0.10 g, 1.9 mmol, 5.3 eq) was added and the mixture was stirred for 1 h. 1M HCl(aq) was added until the solution reached a pH of 6. The reaction mixture was then concentrated under reduced pressure and purified by column chromatography (SiO_2 H_2O : MeOH : EtOAc :: 1:1:8) to provide **α-Gal-FL** as a red powder (40%, 82 mg). ^1H NMR (300 MHz, MeOD) δ = 8.28 (1H, *d, J* = 7.4 Hz), 7.91-7.73 (2H, *m*), 7.42 (1H, *d, J* = 6.09 Hz), 7.03 (2H, *d, J* = 8.90 Hz), 6.83-6.64 (4H, *m*), 4.81 (1H, *d, J* = 6.2 Hz), 4.01-3.85 (3H, *m*), 3.84-3.69 (5H, *m*), 3.66-3.55 (1H, *m*), 3.32-3.18 (1H, *m*), 1.48-1.30 (2H, *m*), 1.29-1.12 (2H, *m*), 1.05-0.89 (2H, *m*); ^{13}C NMR (75 MHz, MeOD) δ = 176.53, 166.95, 158.90, 156.70, 134.84, 133.90, 132.27, 132.00, 131.86, 131.59, 131.23, 122.70, 116.41, 104.38, 100.27, 79.44, 72.29, 71.45, 71.02, 70.22, 68.76, 66.66, 62.73, 36.96, 35.41, 32.67, 31.67, 30.10, 29.19, 23.58, 14.42; m/z calcd for $C_{31}H_{32}O_{11}$ $[M+H]^+$: 581.2018, found 581.2014; $\lambda_{max,abs}$ = 495 nm, ε = 3500 $cm^{-1}M^{-1}$, $\lambda_{max,em}$ = 519 nm. The stereochemical assignment was confirmed by ^1H – ^1H COSY, ^1H – ^{13}C HSQC, ^1H – ^{13}C HMBC, and ^1H – ^1H NOESY NMR spectroscopy, which taken together all support the structural assignment of **α-Gal-FL** as an α-galactoside (**Figures S24-S29**).

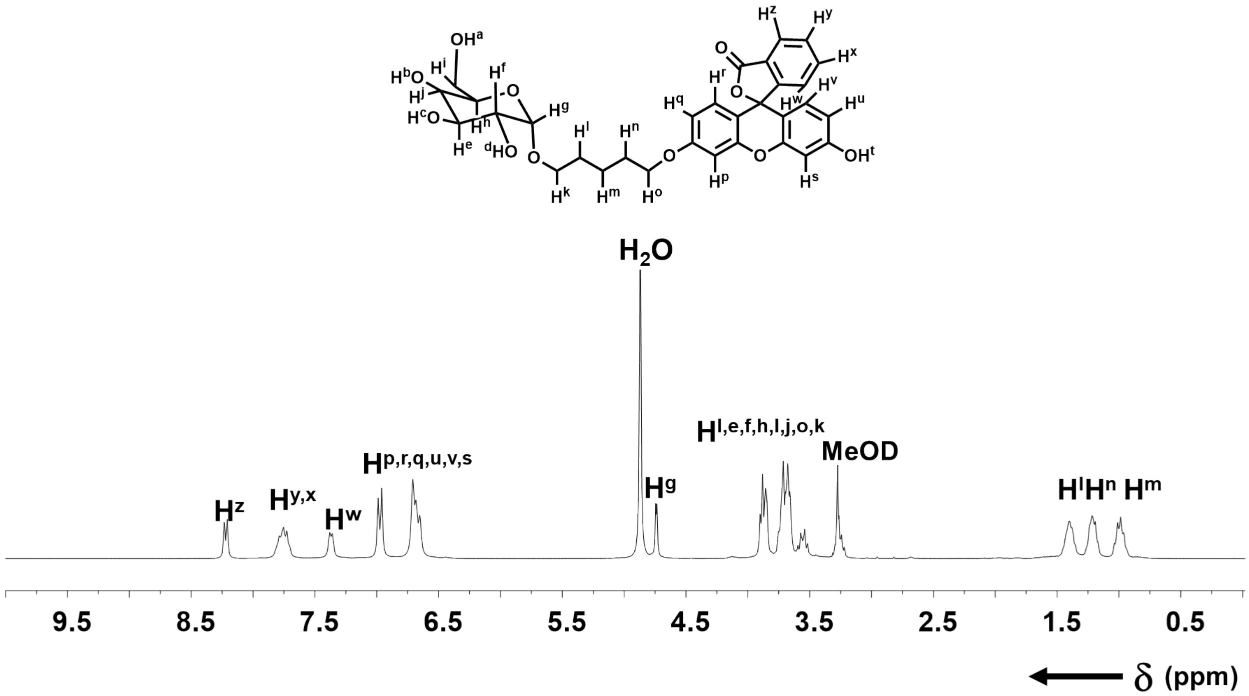

Figure S20. ^{1}H NMR (300 MHz) of **α-Gal-FL** at 25 °C in MeOD.

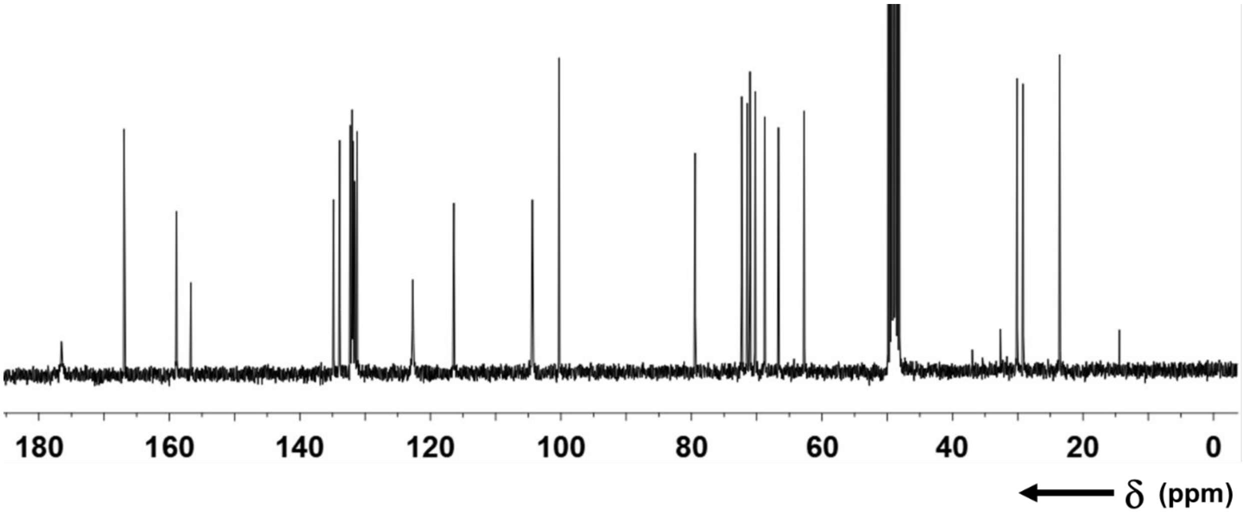

Figure S21. ^{13}C NMR (75 MHz) of **α-Gal-FL** at 25 °C in MeOD.

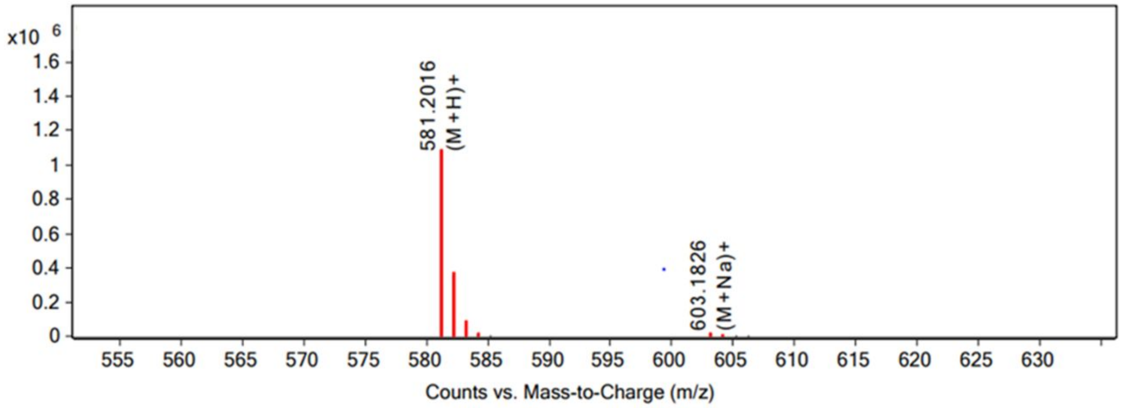

Figure S22. HR-MS of **α-Gal-FL**.

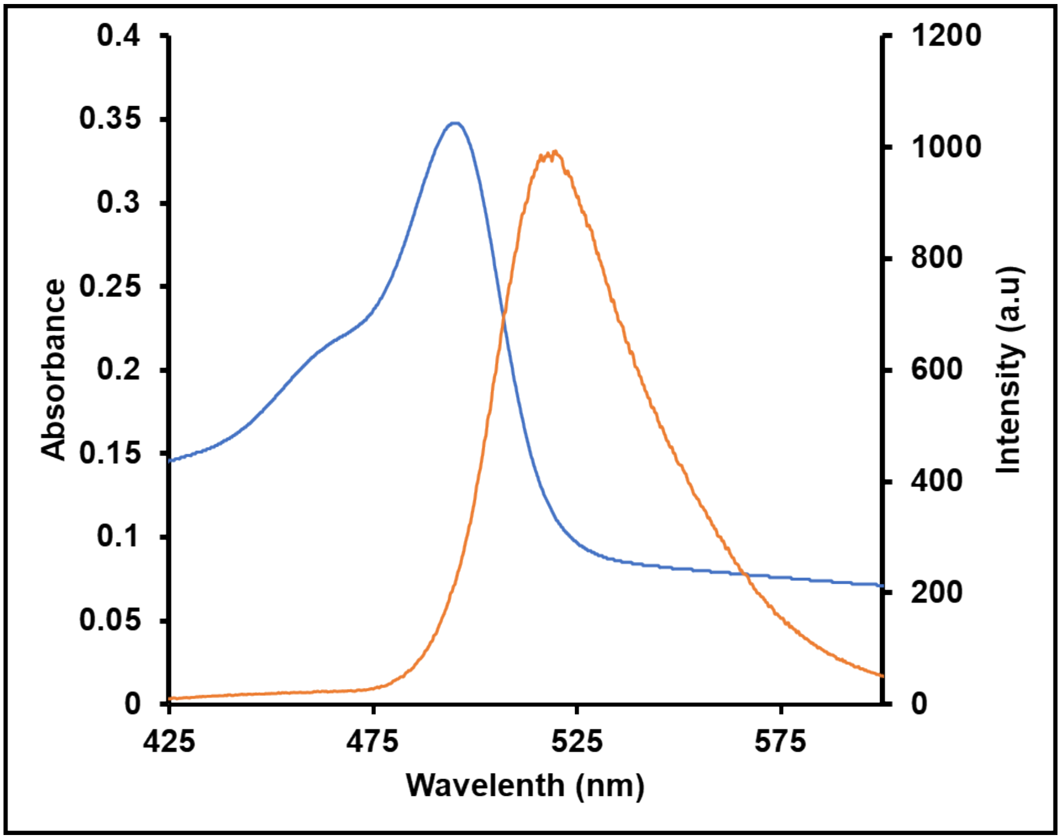

Figure S23. Absorption (blue) and fluorescence emission (orange) spectra (λ_{ex} = 425 nm) of **α-Gal-FL**. Fluorophore was dissolved in a solution of Tris buffer 20 mM, pH = 7.4, 0.01% Tween20.

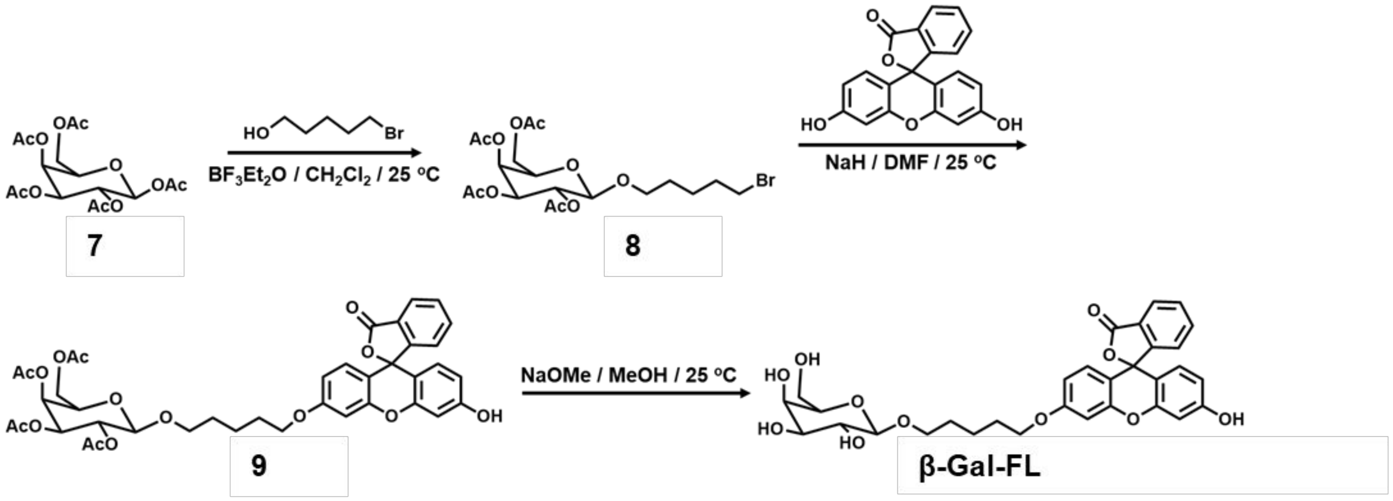

Scheme S4. Synthesis of **β-Gal-FL.**

Synthesis of **(2R,3S,4S,5R,6R)-2-(acetoxymethyl)-6-((5-bromopentyl)oxy)tetrahydro-2H-pyran-3,4,5-triyl triacetate (8).** To a solution of (**7**) (5.0 g, 12.8 mmol) in anhydrous CH_2Cl_2 was added 5-bromo penten-1-ol (4.3 g, 25.6 mmol) dropwise. The mixture was cooled to –20 °C and treated with BF_3•Et_2O (21.0 g, 148 mmol) dropwise. After 30 min, the reaction mixture was warmed to room temperature and then stirred for 16 h. The mixture was then washed with saturated $NaHCO_3$(aq) (3 x 50 mL). The organic layer was dried over Na_2SO_4 (anhydrous) and concentrated under reduced pressure. The crude product was purified which revealed the reaction produced both anomers in an α:β of approximately 50:50. The α-isomer & β-isomers were isolated in ~90% purity (by NMR analysis) using column chromatography (SiO_2 :: EtOAc: Hexanes :: 2:8) to provide **8** as a clear viscous liquid (9%, 0.8 g). ^{1}H NMR (300MHz, $CDCl_3$) δ = 5.32 (1H, *d, J* = 1.60 Hz), 5.19-5.07 (1H, *m*), 4.95 (1H, *dd, J = 3.25 Hz*), 4.40 (1H, *d, J* = 3.87 Hz), 4.20-3.96 (2H, *m*), 3.90-3.77 (2H, *m*), 3.48-3.28 (3H, *m*), 2.08 (3H, *s*), 1.99 (6H, *d, J* = 2.17 Hz), 1.91 (3H, *s*), 1.86-1.73 (2H, *m*), 1.59-1.49 (2H, *m*), 1.48-1.35 (2H, *m*) ; ^{13}C NMR (75 MHz, $CDCl_3$) δ = 173.62, 170.50, 170.38, 170.26, 170.15, 170.09, 170.03, 170.00, 169.86,

169.67, 169.37, 168.91, 105.45, 101.24, 101.12, 96.11, 89.65, 81.33, 79.94, 73.29, 72.00, 70.90, 70.57, 69.71, 69.39, 69.28, 69.13, 68.86, 68.72, 68.22, 68.07, 67.61, 67.38, 67.35, 67.32, 67.06, 66.42, 66.11, 62.61, 62.34, 61.78, 61.28, 60.35, 33.65, 32.57, 32.32, 29.63, 28.83, 28.54, 28.43, 24.89, 24.73, 24.57, 21.01, 20.85, 20.78, 20.66, 20.65, 20.56, 20.51, 20.41, 14.17 ; m/z calcd for $C_{19}H_{29}BrO_{10}$ $[M+H]^+$: 499.0996, found 499.1012.

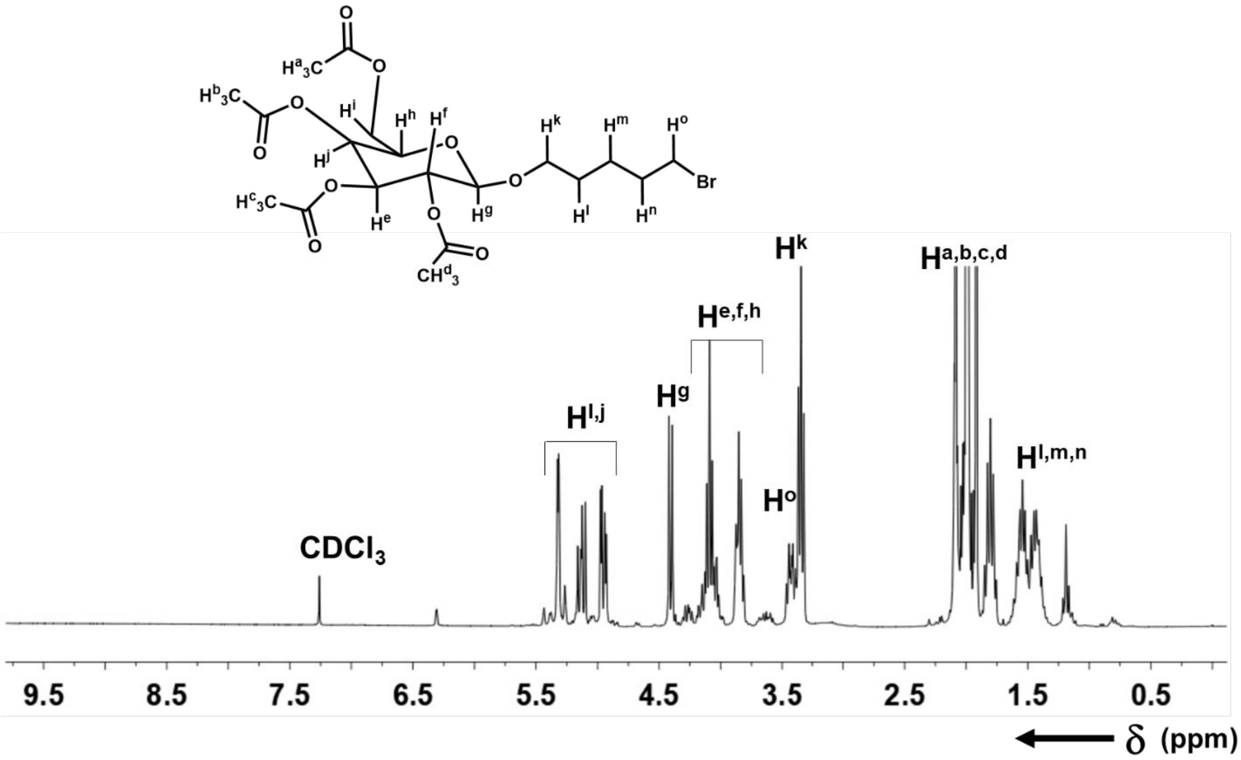

Figure S24. ^{1}H NMR of **8** (300 MHz), 25 ^{o}C in $CDCl_3$.

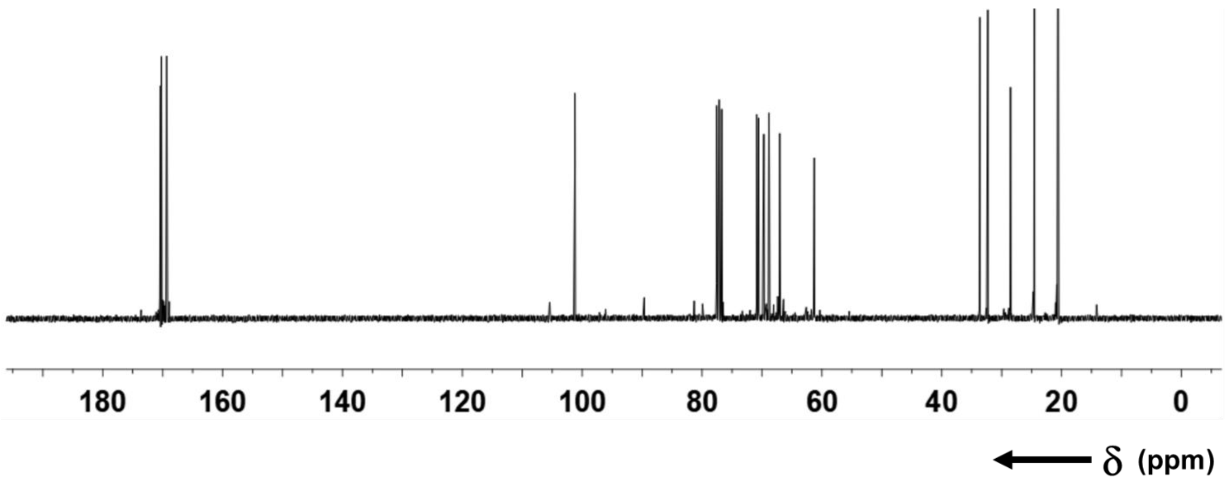

Figure S25. ^{13}C NMR of **8** (75 MHz), 25 ^{o}C in $CDCl_3$.

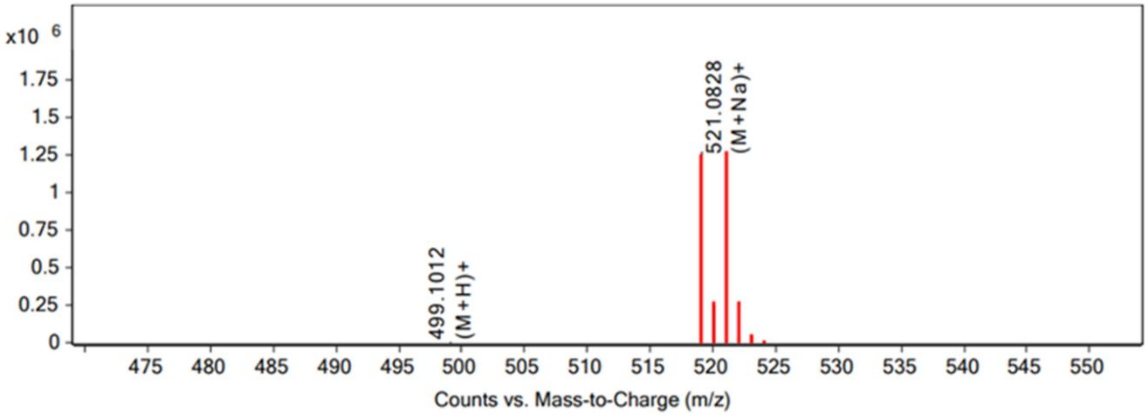

Figure S26. HR-MS of **8.**

Synthesis of **(2R,3S,4S,5R,6R)-2-(acetoxymethyl)-6-((5-((3'-hydroxy-3-oxo-3H-spiro[isobenzofuran-1,9'-xanthen]-6'-yl)oxy)pentyl)oxy)tetrahydro-2H-pyran-3,4,5-triyl triacetate (9)**. (**8**) (0.8 g, 1.6 mmol, 1.0 eq) was dissolved in DMF and stirred. To the solution was added fluorescein (0.5 g, 1.6 mmol, 1.0 eq). The reaction was then cooled down to 0 °C, and NaH (0.1 g, 2.5 mmol, 1.5 eq) was added. The reaction was left at 0 °C for 30 min then cooled to room temperature and stirred for 16 h. DMF was removed under reduced pressure. The crude product was purified by column chromatography (SiO_2 :: EtOAc: Hexanes:: 5:5) to provide **9** as a red powder (12%, 165 mg). ^{1}H NMR (300MHz, $CDCl_3$) δ = 8.25 (1H, *dd, J* = 3.24 Hz), 7.78-7.63 (2H, *m*), 7.30 (1H, *dd, J* = 3.38 Hz), 6.96 (2H, *dd, J* = 4.07 Hz), 6.85 (2H, *s*), 6.77 (2H, *dd, J* = 3.58 Hz), 5.37 (1H,

d, J = 1.30 Hz), 5.21-5.08 (1H, *m*), 5.07-4.95 (1H, *m*), 4.44 (1H, *d, J* = 3.91 Hz), 4.23-4.03 (2H, *m*), 4.02-3.85 (3H, *m*), 3.85-3.73 (1H, *m*), 3.41-3.27 (1H, *m*), 2.12 (3H, *s*), 1.99 (3H, *s*), 1.96 (3H, *s*), 1.53-1.37 (2H, *m*), 1.37-1.17 (2H, *m*), 1.15-0.96 (2H, *m*) ; ^{13}C NMR (75 MHz, $CDCl_3$) δ = 170.63, 170.63,170.44, 170.44, 170.31, 170.31, 169.70, 169.70, 165.46, 165.46, 157.73, 157.73, 155.26, 134.20, 134.20, 130.61, 130.61, 115.10, 115.10, 115.09, 115.09, 103.90, 101.42, 101.42, 71.06, 70.66, 69.10, 67.24, 61.40, 29.10, 28.09, 22.36, 20.90, 20.90, 20.84, 20.84, 20.82, 20.82, 20.75, 20.75; m/z calcd for $C_{39}H_{40}O_{15}$ $[M+H]^+$: 749.2441, found: 749.2440.

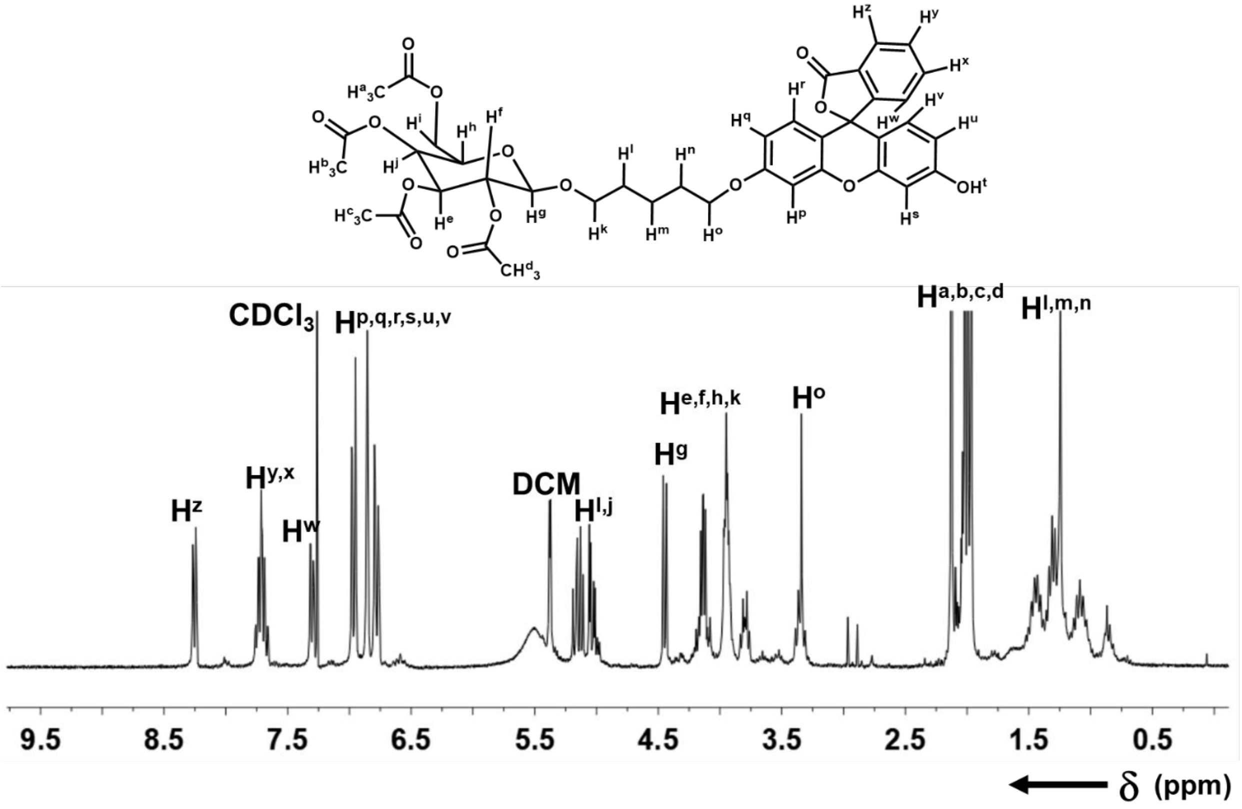

Figure S27. ^{1}H NMR of **9** (300 MHz), 25 ^{o}C in $CDCl_3$.

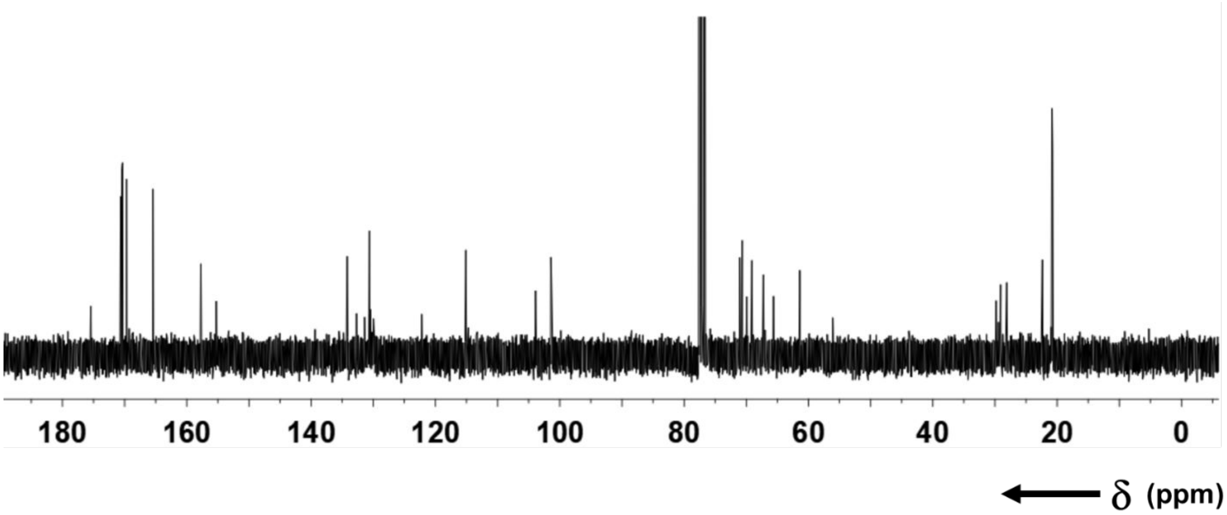

Figure S28*.* ^{13}C NMR of **9** (75 MHz), 25 ^{o}C in $CDCl_3$.

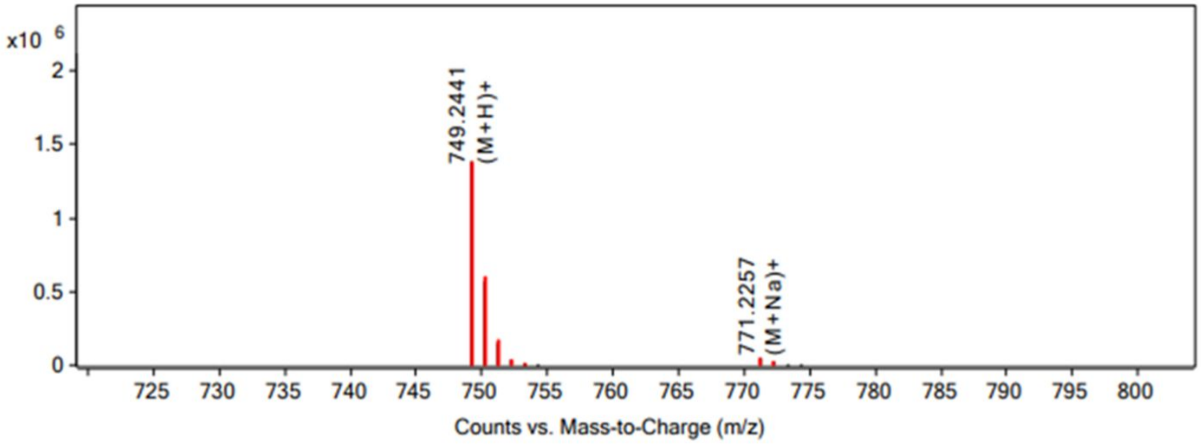

Figure S29. HR-MS of **9.**

Synthesis of **β-Gal-FL.** (**9**) (0.16 g, 0.22 mmol, 1.0 eq) was dissolved in MeOH (30 mL) and cooled to 0 °C. After 30 min, NaOMe (0.1 g, 1.2 mmol, 5.3 eq) was added and the mixture was stirred for 1 h. 1M HCl(aq) was added until the solution reached a pH of 6. The reaction mixture was then concentrated under reduced pressure and purified by column chromatography (SiO_2 :: H_2O: MeOH: EtOAc :: 1:1:8) to provide **β-Gal-FL** as a red powder (46%, 58 mg). ^1H NMR (300 MHz, MeOD) δ = 8.21 (1H, *d, J* = 3.77 Hz), 7.81-7.67 (2H, *m*), 7.35 (1H, *d, J* = 3.69 Hz), 6.96 (2H, *d, J* = 4.80 Hz), 6.74-6.61 (4H, *m*), 4.74 (1H, *d, J* = 1.47 Hz), 3.94-3.78 (3H, *m*), 3.78-3.60 (5H, *m*), 3.60-3.48 (1H, *m*), 3.32-3.18

(1H, *m*), 1.48-1.30 (2H, *m*), 1.29-1.12 (2H, *m*), 1.05-0.89 (2H, *m*) ; ^{13}C NMR (75 MHz, MeOD) δ = 134.88, 133.96, 132.33, 132.06, 131.92, 131.63, 131.29, 116.46, 104.90, 104.43, 76.44, 74.92, 72.51, 70.46, 70.35, 66.74, 62.58, 30.35, 29.21, 23.51; m/z calcd for $C_{31}H_{32}O_{11}$ $[M+H]^+$: 581.2018, found 581.2014.

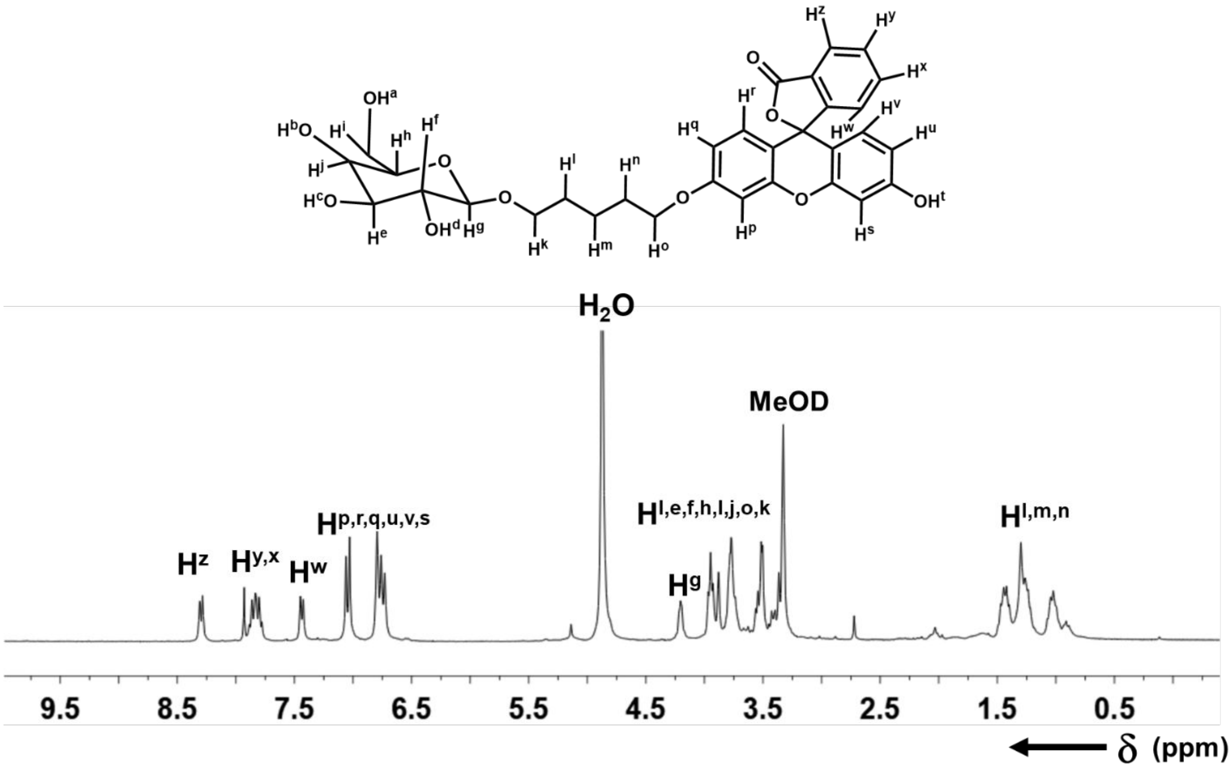

Figure S30. ^1H NMR of **β-Gal-FL** (300 MHz), 25 ^oC in MeOD.

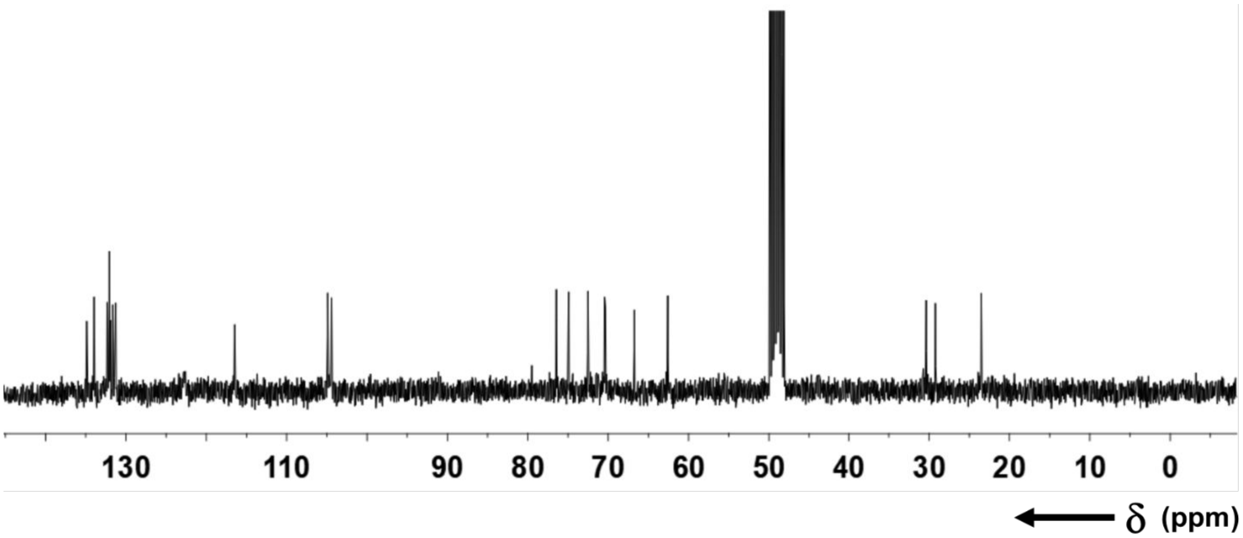

Figure S31. ^{13}C NMR of **β-Gal-FL** (75 MHz), 25 ^{o}C in MeOD.

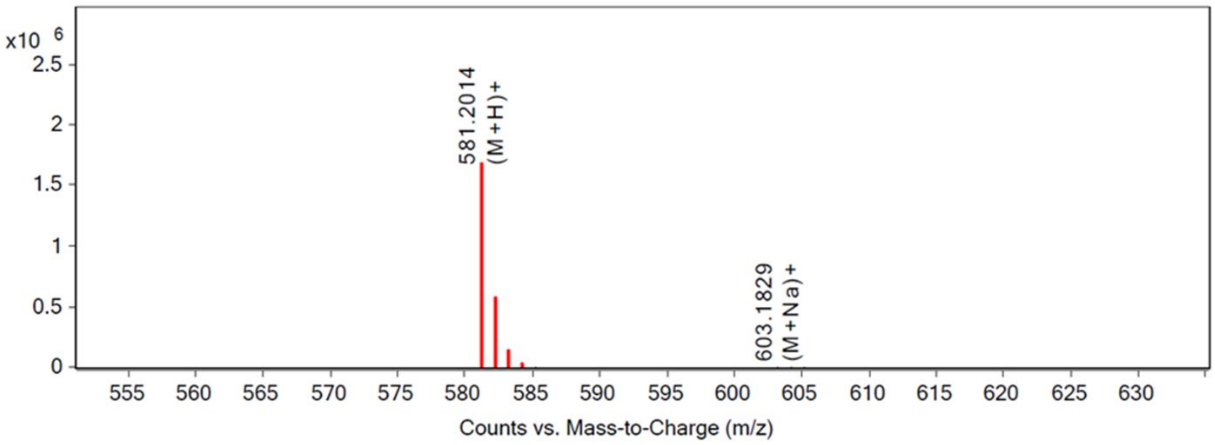

Figure S32. HR-MS of **β-Gal-FL.**

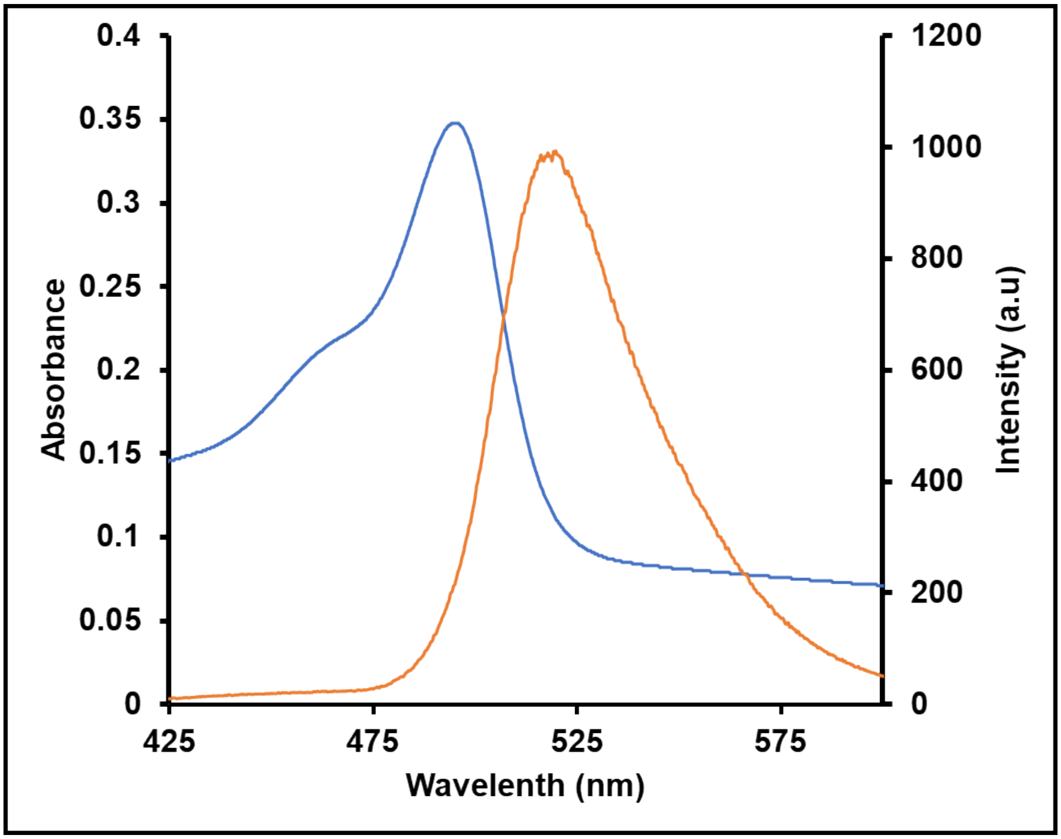

Figure S33. Absorption (blue) and fluorescence emission (orange) spectra (λ_{ex} = 425 nm) of **β-Gal-FL**. Fluorophore was dissolved in a solution of Tris buffer 20 mM, pH = 7.4, 0.01% Tween20.

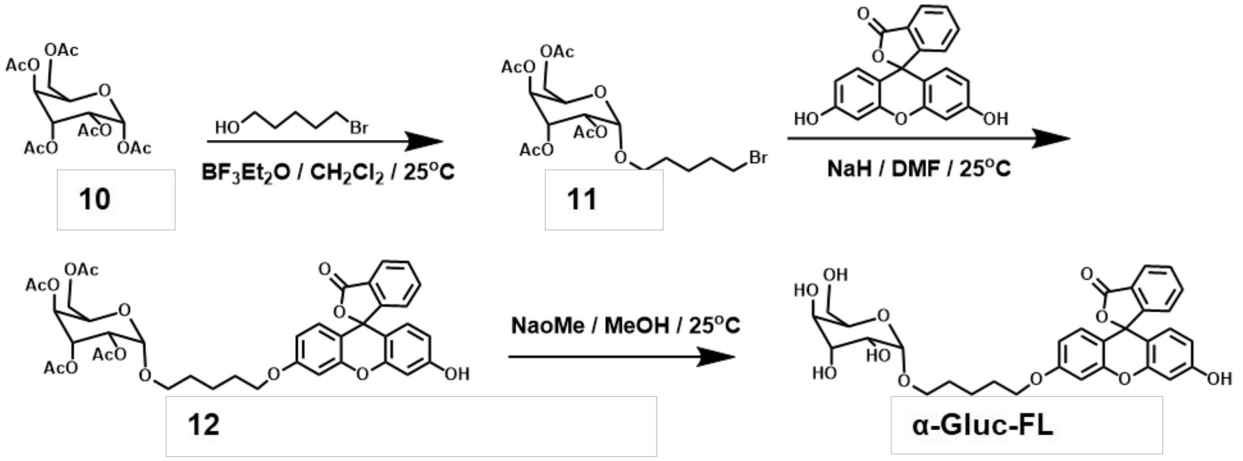

Scheme S5. Synthesis of **α-Gluc-FL.**

Synthesis of **(2R,3S,4R,5R,6S)-2-(acetoxymethyl)-6-((5-bromopentyl)oxy)tetrahydro-2H-pyran-3,4,5-triyl triacetate (11).** To a solution of (**10**) (5.0 g, 12.8 mmol) in anhydrous CH_2Cl_2 was added 5-bromo penten-1-ol (4.3 g, 25.6 mmol) dropwise. The mixture was cooled to -20 °C and treated with BF_3•Et_2O (21.0 g, 148 mmol) dropwise. After 30 min, the reaction mixture was warmed to room temperature and then stirred for 16 h. The mixture was then washed with saturated $NaHCO_3$(aq) (3 x 50 mL). The organic layer was dried over Na_2SO_4 (anhydrous) and concentrated under reduced pressure. The crude product was purified which revealed the reaction produced both anomers in an α:β of approximately 50:50. The α-isomer & β-isomers were isolated in ~90% purity (by NMR analysis) using column chromatography (SiO_2 :: EtOAc: Hexanes :: 2:8) to provide, **11** as a clear viscous liquid (5%, 0.5 g). ^{1}H NMR (300MHz, $CDCl_3$) δ = 5.47 (t, J = 9.8 Hz, 1H), 5.07 (dd, J = 11.1, 6.7 Hz, 2H), 4.84 (dt, J = 8.9, 4.4 Hz, 1H), 4.25 (dd, J = 12.3, 4.5 Hz, 1H), 4.18 – 4.05 (m, 2H), 4.00 (ddd, J = 10.1, 4.2, 2.2 Hz, 1H), 3.78 – 3.59 (m, 2H), 3.47 – 3.35 (m, 5H), 2.08 (d, J = 4.6 Hz, 5H), 2.06 (s, 3H), 2.04 (s, 1H), 2.03 (s, 4H), 2.00 (d, J = 2.6 Hz, 4H), 1.96 – 1.80 (m, 4H), 1.75 – 1.41 (m, 9H).

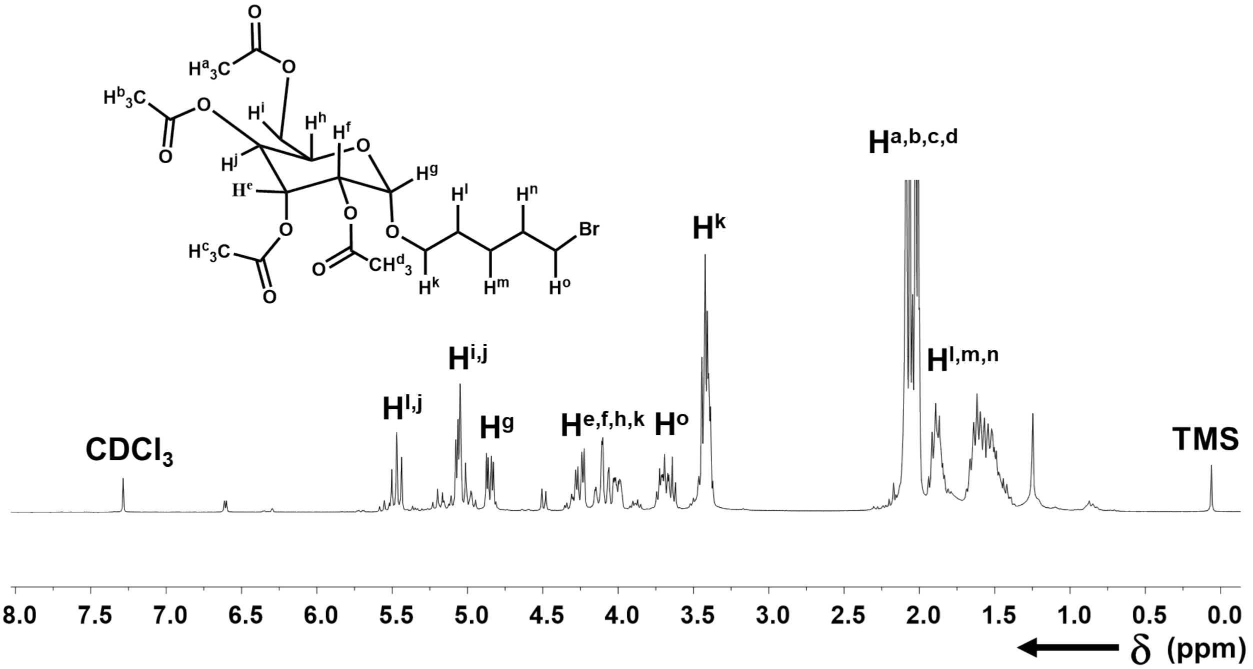

Figure S34. ¹H NMR of **11** (300 MHz), 25 °C in $CDCl_3$.

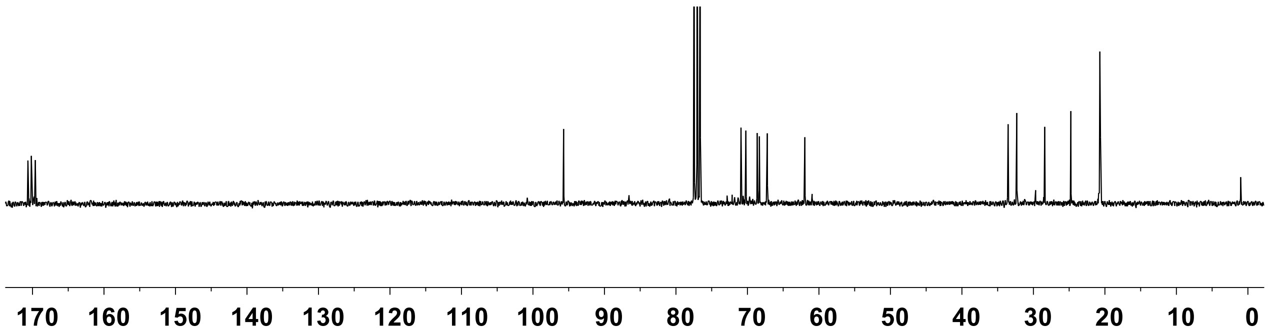

Figure S35. ¹³C NMR of **11** (75 MHz), 25 °C in $CDCl_3$.

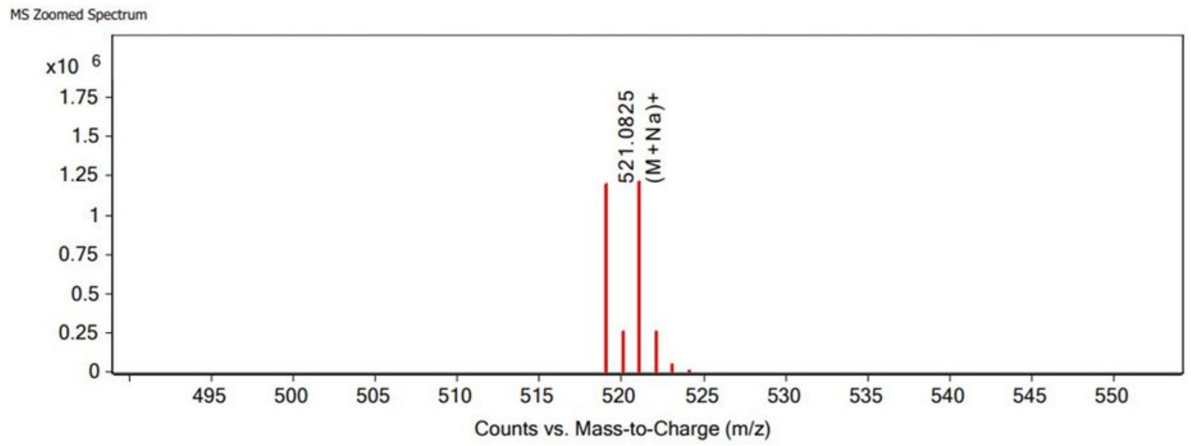

Figure S36. HR-MS of **11**.

Synthesis of **(2R,3S,4R,5R,6S)-2-(acetoxymethyl)-6-((5-((3'-hydroxy-3-oxo-3H-spiro[isobenzofuran-1,9'-xanthen]-6'-yl)oxy)pentyl)oxy)tetrahydro-2H-pyran-3,4,5-triyl triacetate (12)**. **11** (0.60 g, 1.2 mmol, 1.0 eq) was dissolved in DMF (30 mL) and stirred. To the solution was added fluorescein (0.40 g, 1.2 mmol, 1.0 eq). The reaction was then cooled to 0 °C, and NaH (0.040 g, 1.8 mmol, 1.5 eq) was added. The reaction was left at 0 °C for 30 min then warmed to room temperature and stirred for 16 h. DMF was removed under reduced pressure. The mixture was purified by column chromatography (SiO_2 EtOAc : Hexanes :: 5:5) to provide **12** as a red powder (24%, 198 mg). ^{1}H NMR (300 MHz, $CDCl_3$) δ = 8.28 (d, J = 7.4 Hz, 1H), 7.87 – 7.69 (m, 2H), 7.34 (dd, J = 16.4, 9.3 Hz, 1H), 6.99 (d, J = 9.2 Hz, 2H), 6.89 (s, 2H), 6.80 (d, J = 9.2 Hz, 2H), 5.47 (t, J = 9.8 Hz, 1H), 5.15 – 5.01 (m, 2H), 4.92 – 4.80 (m, 1H), 4.26 (dt, J = 14.3, 7.2 Hz, 1H), 4.15 – 4.04 (m, 2H), 3.99 (t, J = 6.3 Hz, 2H), 3.75 (dd, J = 18.0, 4.5 Hz, 2H), 3.36 (d, J = 7.6 Hz, 2H), 2.30 – 1.90 (m, 15H), 1.63 – 0.79 (m, 12H).

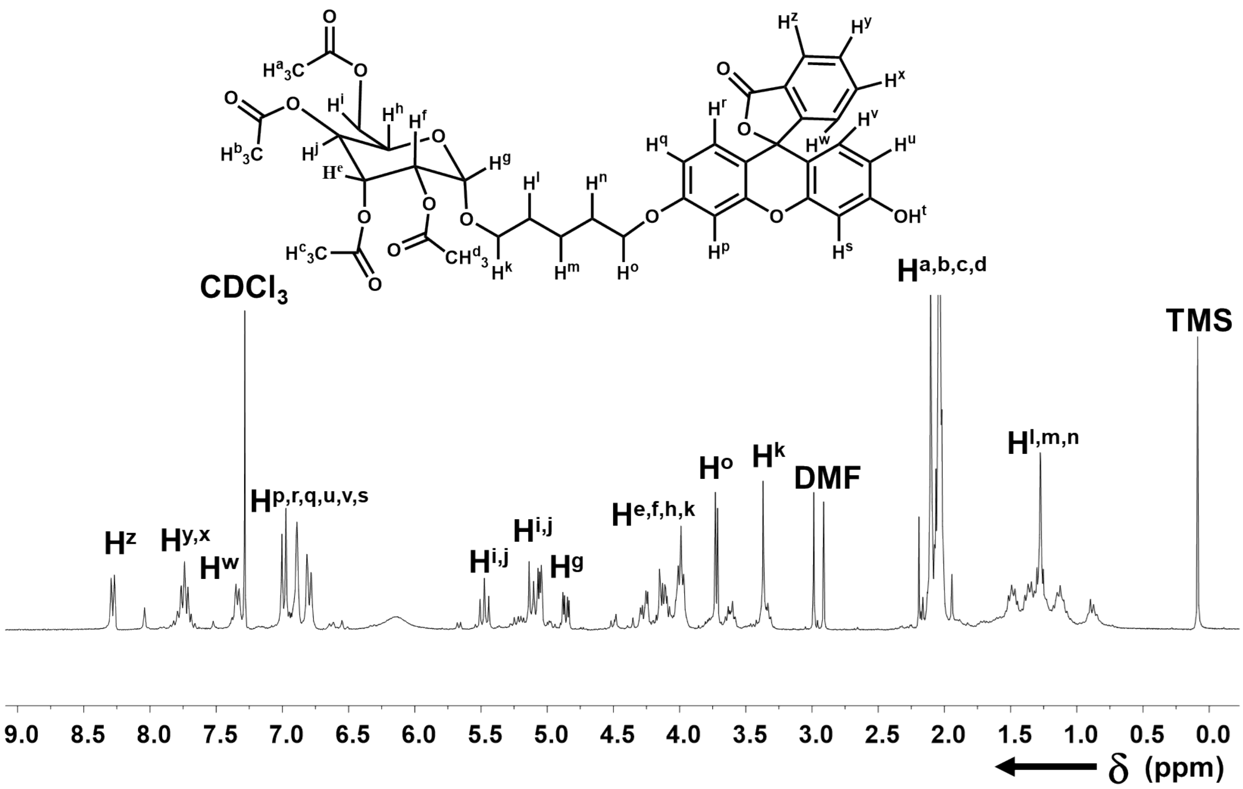

Figure S37*.* ^{1}H NMR of **12** (300 MHz), 25 ^{o}C in CDCl$_3$.

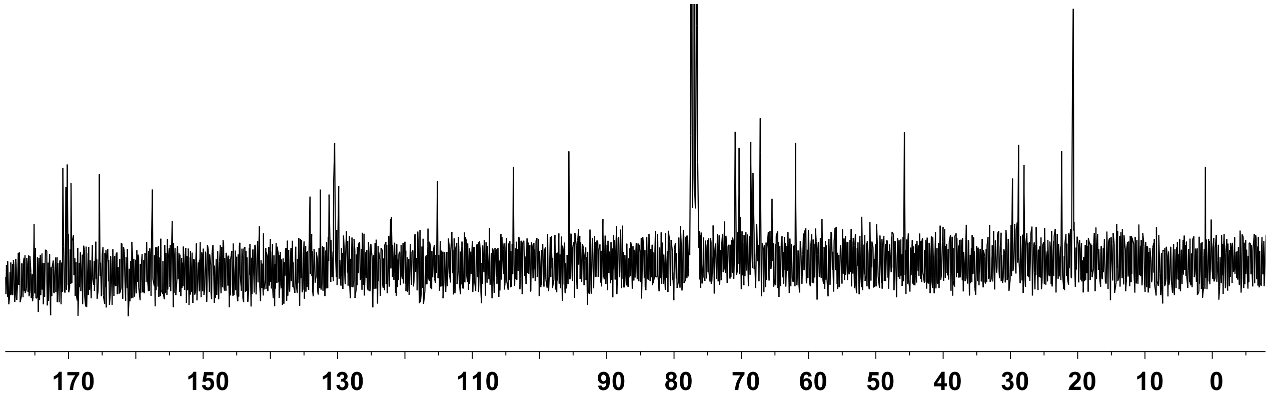

Figure S38*.* ^{13}C NMR of **12** (75 MHz), 25 ^{o}C in CDCl$_3$.

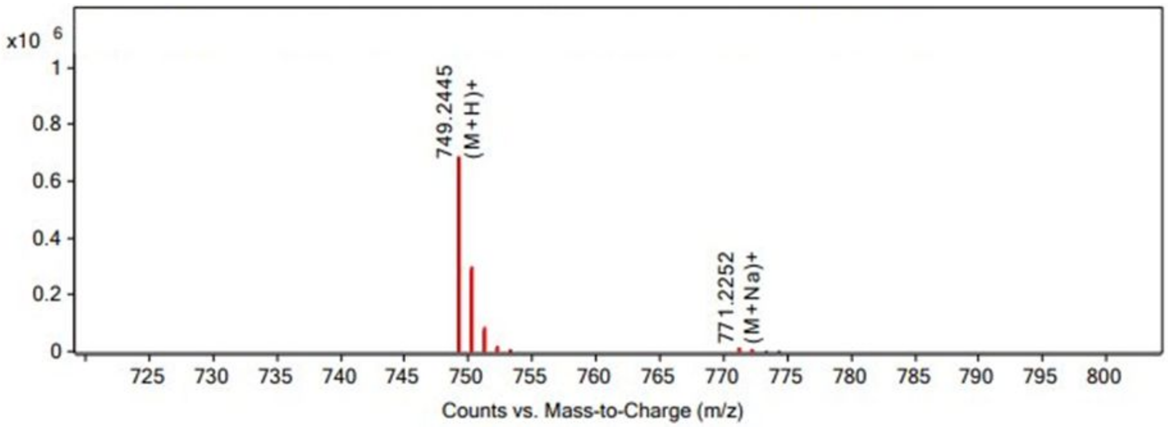

Figure S39. HR-MS of **12**.

Synthesis of **α-Gluc-FL. 12** (0.20 g, 0.30 mmol, 1.0 eq) was dissolved in MeOH (30 mL) and cooled to 0 °C. After 30 min, NaOMe (0.10 g, 1.6 mmol, 5.3 eq) was added, and the mixture was stirred for 1 h. 1M HCl(aq) was added until the solution reached a pH of 6. The reaction mixture was then concentrated under reduced pressure and purified by column chromatography (SiO_2 H_2O : MeOH: EtOAc :: 1:1:8) to provide **α-Gluc-FL** as a red powder (45%, 70 mg). ^{1}H NMR (300 MHz, MeOD) δ = 8.30 (d, J = 7.7 Hz, 1H), 7.83 (dt, J = 32.7, 7.5 Hz, 2H), 7.44 (d, J = 7.4 Hz, 1H), 7.04 (d, J = 9.2 Hz, 2H), 6.79 (s, 2H), 6.74 (d, J = 9.2 Hz, 2H), 4.76 (d, J = 3.7 Hz, 1H), 4.00 – 3.91 (m, 2H), 3.81 (td, J = 12.0, 3.7 Hz, 1H), 3.69 (dd, J = 11.8, 5.6 Hz, 1H), 3.67 – 3.59 (m, 2H), 3.44 – 3.38 (m, 1H), 3.37 (s, J = 7.8 Hz, 1H), 3.36 – 3.28 (m, J = 12.2, 6.6 Hz, 5H), 1.56 – 1.40 (m, 2H), 1.34 – 1.23 (m, 3H), 1.11 – 1.00 (m, 2H).

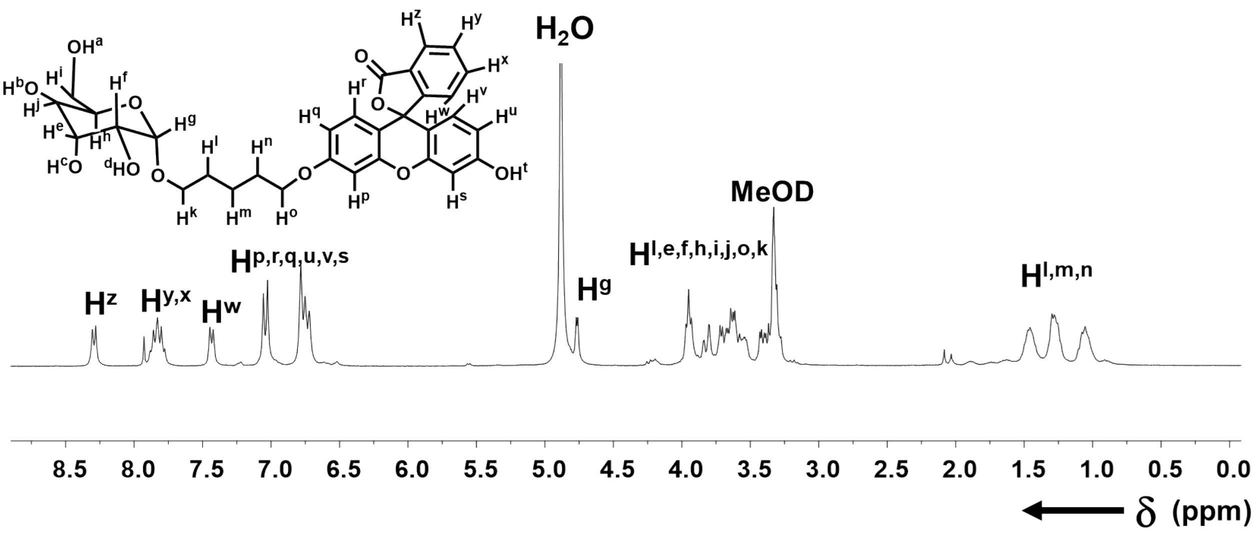

Figure S40*.* ^{1}H NMR of **α-Gluc-FL** (300 MHz), 25 ^{o}C in MeOD.

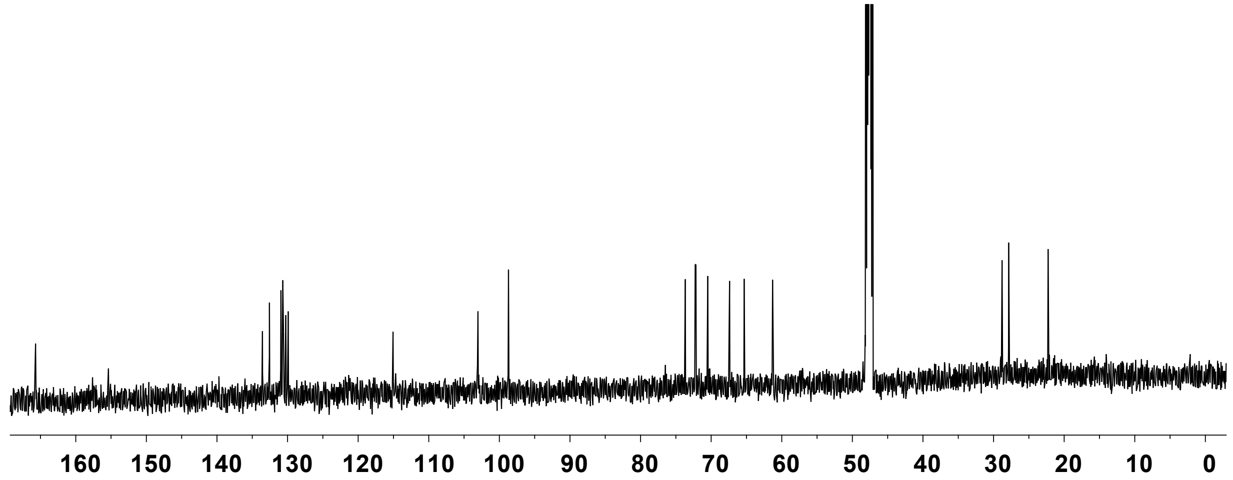

Figure S41*.* ^{13}C NMR of **α-Gluc-FL** (75 MHz), 25 ^{o}C in MeOD.

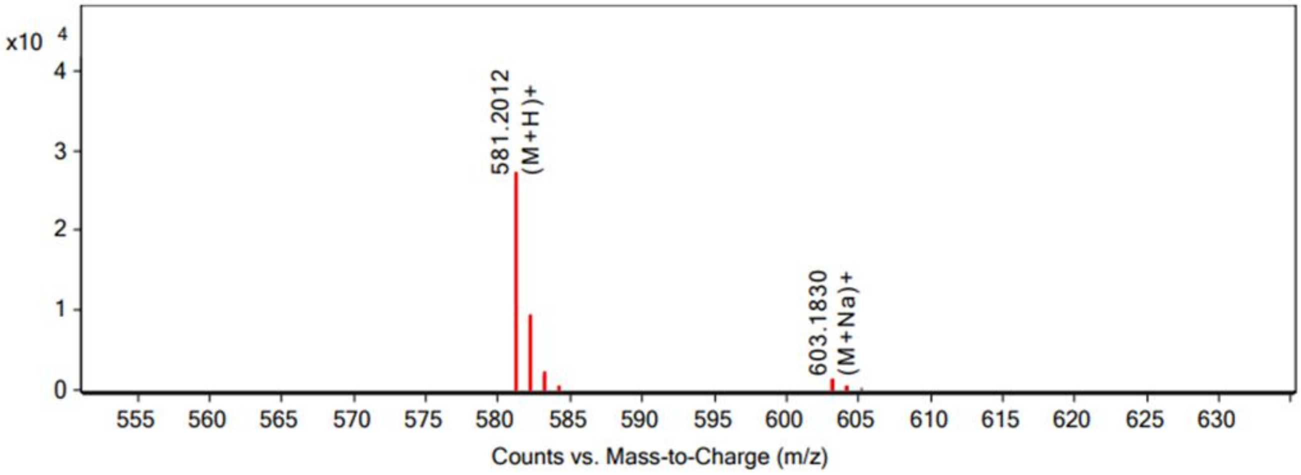

Figure S42. HR-MS of **α-Gluc-FL.**

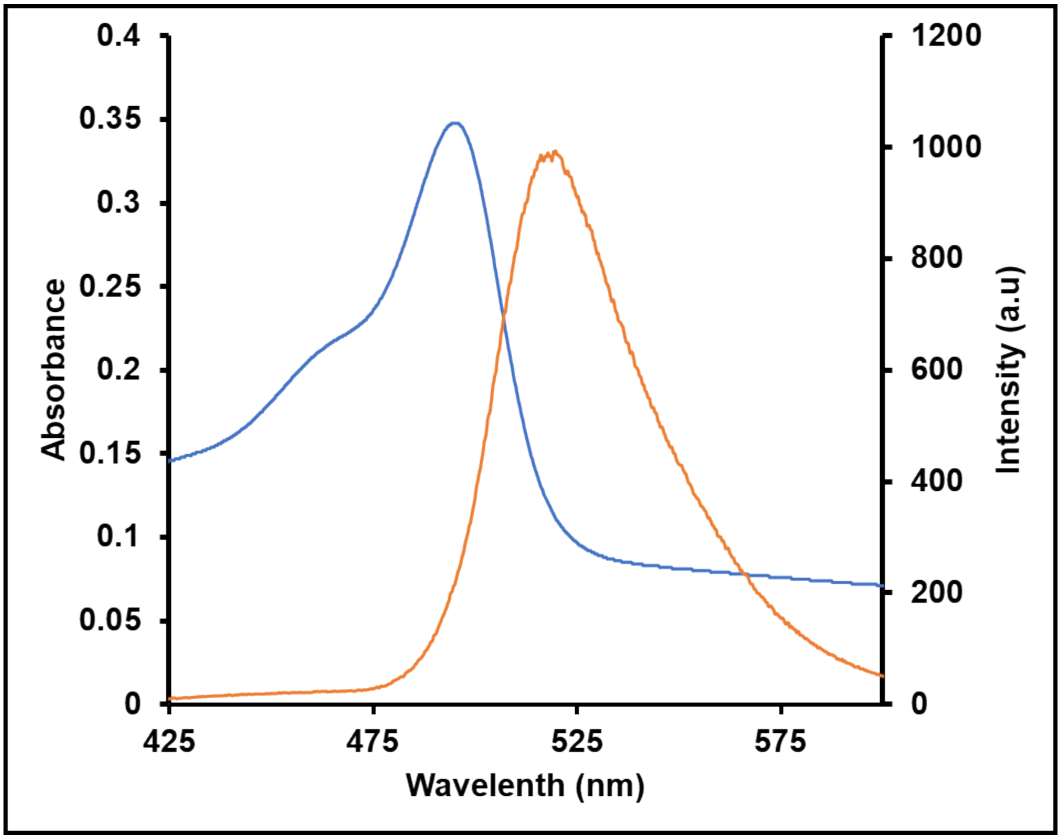

Figure S43. Absorption (blue) and fluorescence emission (orange) spectra (λ_{ex} = 425 nm) of **α-Gluc-FL**. Fluorophore was dissolved in a solution of Tris buffer 20 mM, pH = 7.4, 0.01% Tween20.

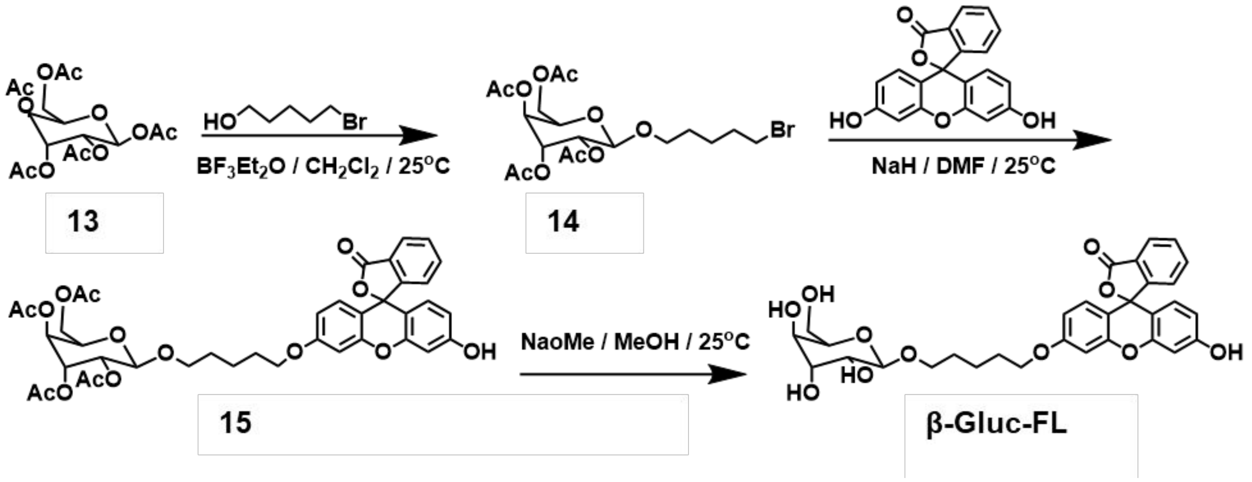

Scheme S6. Synthesis of **β-Gluc-FL.**

Synthesis of **(2R,3S,4R,5R,6R)-2-(acetoxymethyl)-6-((5-bromopentyl)oxy)tetrahydro-2H-pyran-3,4,5-triyl triacetate (14).** To a solution of (**13**) (5.0 g, 12.8 mmol) in anhydrous CH_2Cl_2 was added 5-bromo penten-1-ol (4.3 g, 25.6 mmol) dropwise. The mixture was cooled to -20 °C and treated with BF_3•Et_2O (21.0 g, 148 mmol) dropwise. After 30 min, the reaction mixture was warmed to room temperature and then stirred for 16 h. The mixture was then washed with saturated $NaHCO_3$(aq) (3 x 50 mL). The organic layer was dried over Na_2SO_4 (anhydrous) and concentrated under reduced pressure. The crude product was purified which revealed the reaction produced both anomers in an α:β of approximately 50:50. The α-isomer & β-isomers were isolated in ~90% purity (by NMR analysis) using column chromatography (SiO_2:: EtOAc: Hexanes:: 2:8) to provide, **13** as a clear viscous liquid (9%, 0.9 g). ^{1}H NMR (300MHz, $CDCl_3$) δ = 5.17 (q, J = 9.1 Hz, 1H), 5.06 (dd, J = 11.4, 7.8 Hz, 1H), 5.01 – 4.93 (m, 1H), 4.49 (d, J = 7.9 Hz, 1H), 4.25 (dd, J = 12.3, 4.7 Hz, 1H), 4.19 – 4.06 (m, 1H), 3.96 – 3.80 (m, 1H), 3.74 – 3.61 (m, 1H), 3.54 – 3.43 (m, 1H), 3.43 – 3.32 (m, 3H), 2.15 – 1.93 (m, 15H), 1.94 – 1.76 (m, 3H), 1.69 – 1.37 (m, 6H), 1.24 (s, J = 7.7 Hz, 1H).

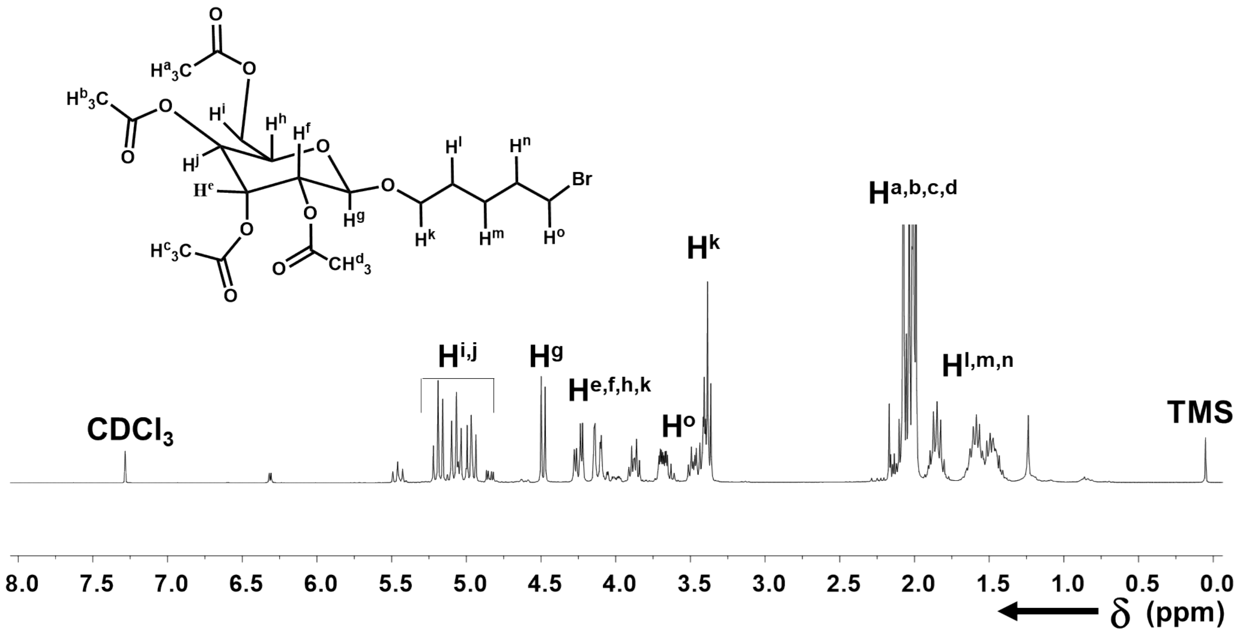

Figure S44. ^{1}H NMR of **14** (300 MHz), 25 ^{o}C in $CDCl_3$.

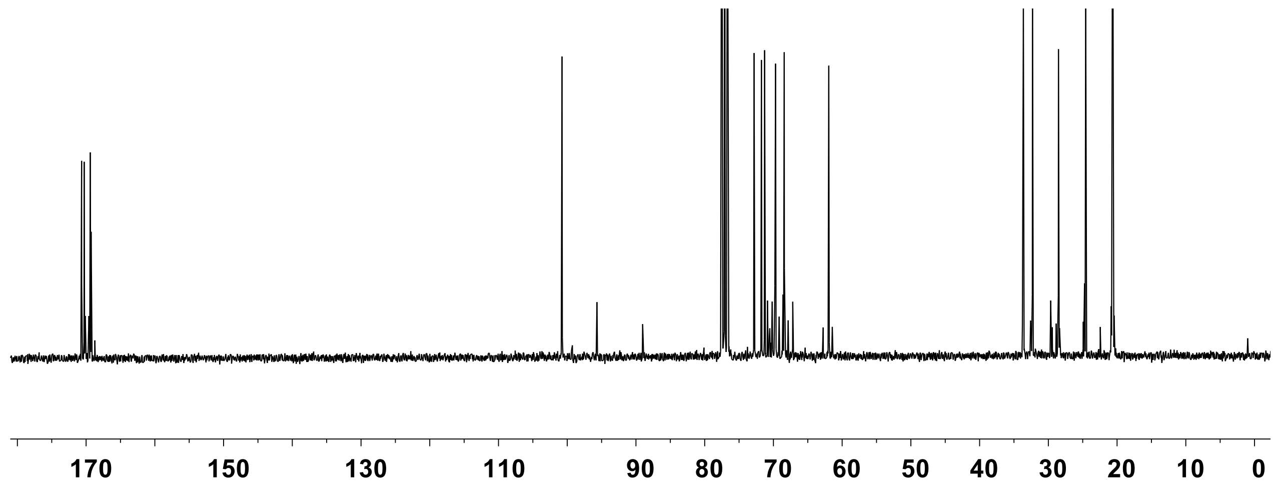

Figure S45. ^{13}C NMR of **14** (75 MHz), 25 ^{o}C in $CDCl_3$.

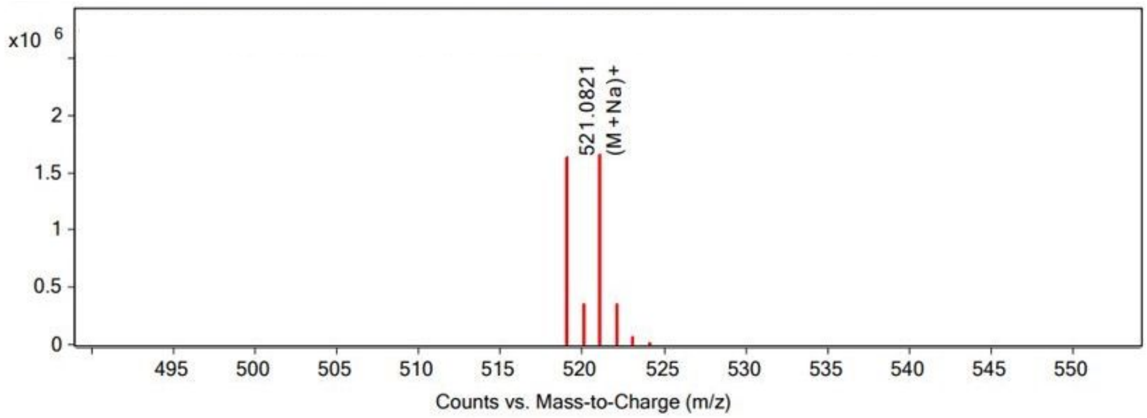

Figure S46. HR-MS of **14.**

Synthesis of **(2R,3S,4R,5R,6R)-2-(acetoxymethyl)-6-((5-((3'-hydroxy-3-oxo-3H-spiro[isobenzofuran-1,9'-xanthen]-6'-yl)oxy)pentyl)oxy)tetrahydro-2H-pyran-3,4,5-triyl triacetate (15)**. **14** (0.60 g, 1.2 mmol, 1.0 eq) was dissolved in DMF (30 mL) and stirred. To the solution was added fluorescein (0.40 g, 1.2 mmol, 1.0 eq). The reaction was then cooled to 0 °C, and NaH (0.040 g, 1.8 mmol, 1.5 eq) was added. The reaction was left at 0 °C for 30 min then warmed to room temperature and stirred for 16 h. DMF was removed under reduced pressure. The mixture was purified by column chromatography (SiO_2 EtOAc : Hexanes :: 5:5) to provide **15** as a red powder (25%, 440 mg). ^{1}H NMR (300 MHz, $CDCl_3$) *δ* = 8.44 – 8.12 (m, 1H), 7.91 – 7.63 (m, 3H), 7.33 (d, J = 7.2 Hz, 1H), 6.98 (t, J = 8.8 Hz, 2H), 6.91 (s, 1H), 6.83 (d, J = 9.2 Hz, 1H), 5.31 – 5.18 (m, 1H), 5.15 – 5.02 (m, 1H), 4.96 (dd, J = 9.5, 8.1 Hz, 1H), 4.50 (d, J = 7.9 Hz, 1H), 4.21 (ddd, J = 14.5, 12.4, 3.3 Hz, 2H), 3.97 (dd, J = 13.4, 7.2 Hz, 2H), 3.89 – 3.68 (m, 2H), 3.46 – 3.25 (m, 1H), 2.32 – 1.92 (m, 13H), 1.60 – 1.37 (m, 2H), 1.31 (dd, J = 15.9, 8.9 Hz, 3H), 1.19 – 0.98 (m, 2H).

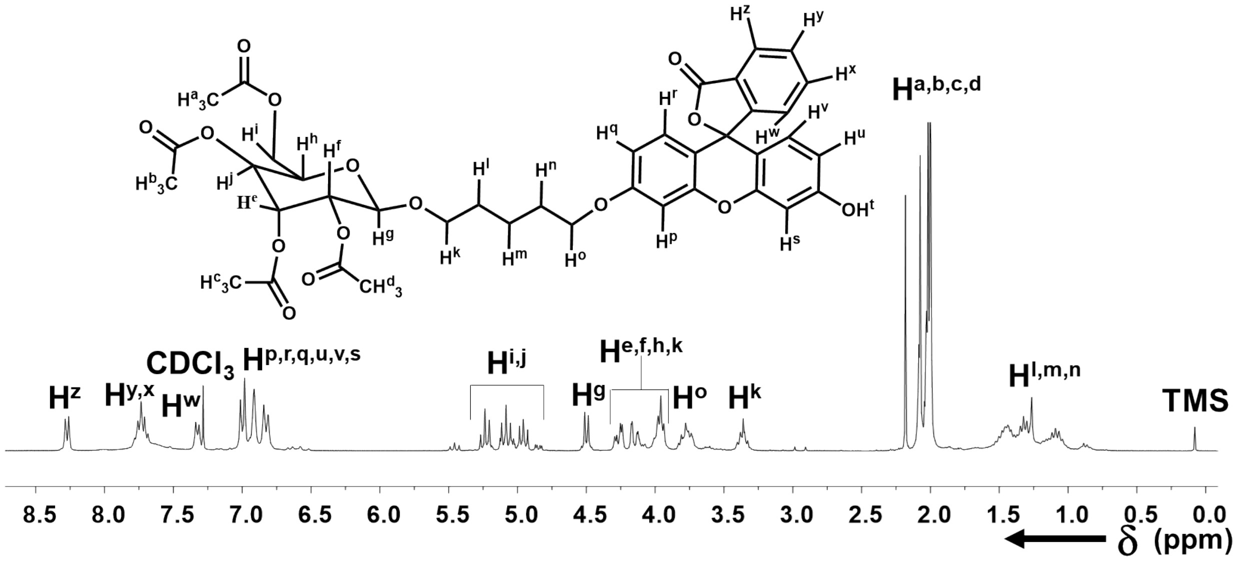

Figure S47. ^{1}H NMR of **15** (300 MHz), 25 ^{o}C in $CDCl_3$.

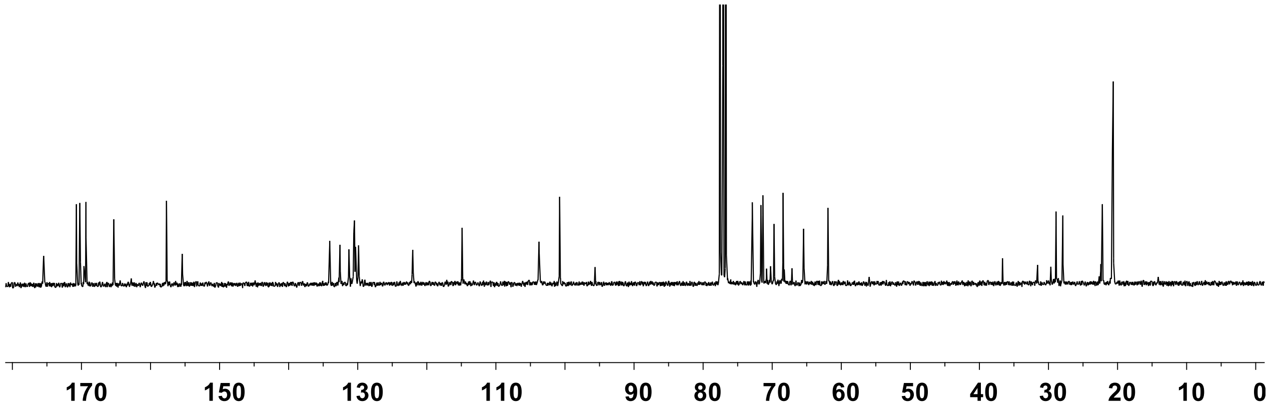

Figure S48. ^{13}C NMR of **15** (75 MHz), 25 ^{o}C in $CDCl_3$.

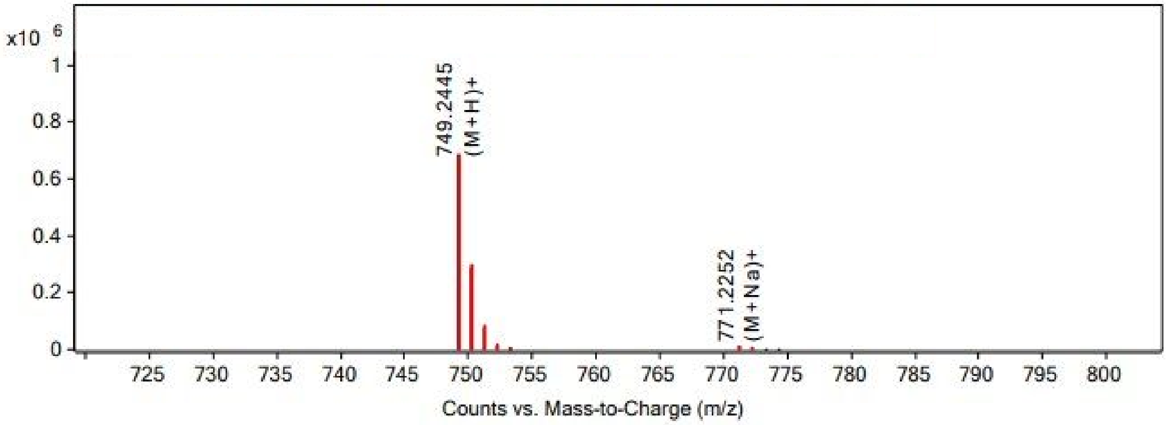

Figure S49. HR-MS of **15**.

Synthesis of **β-Gluc-FL. 15** (0.20 g, 0.30 mmol, 1.0 eq) was dissolved in MeOH (30 mL) and cooled to 0 °C. After 30 min, NaOMe (0.10 g, 1.6 mmol, 5.3 eq) was added, and the mixture was stirred for 1 h. 1M HCl(aq) was added until the solution reached a pH of 6. The reaction mixture was then concentrated under reduced pressure and purified by column chromatography (SiO_2 H_2O : MeOH: EtOAc :: 1:1:8) to provide **β-Gluc-FL** as a red powder (44%, 120 mg). ^{1}H NMR (300 MHz, MeOD) δ = 8.29 (d, J = 7.7 Hz, 1H), 7.82 (dt, J = 34.2, 7.3 Hz, 2H), 7.43 (d, J = 7.3 Hz, 1H), 7.03 (d, J = 9.1 Hz, 2H), 6.76 (s, 1H), 6.72 (d, J = 8.7 Hz, 2H), 4.24 (d, J = 7.7 Hz, 1H), 3.95 (t, J = 5.6 Hz, 2H), 3.89 (t, J = 11.1 Hz, 1H), 3.79 (dt, J = 15.7, 9.2 Hz, 1H), 3.69 (t, J = 13.9 Hz, 1H), 3.59 (ddd, J = 16.1, 13.0, 7.0 Hz, 1H), 3.47 – 3.34 (m, 2H), 3.34 – 3.28 (m, 4H), 3.17 (dd, J = 20.6, 12.1 Hz, 1H), 1.54 – 1.38 (m, 2H), 1.34 – 1.21 (m, 3H), 1.03 (dd, J = 14.6, 7.5 Hz, 2H).

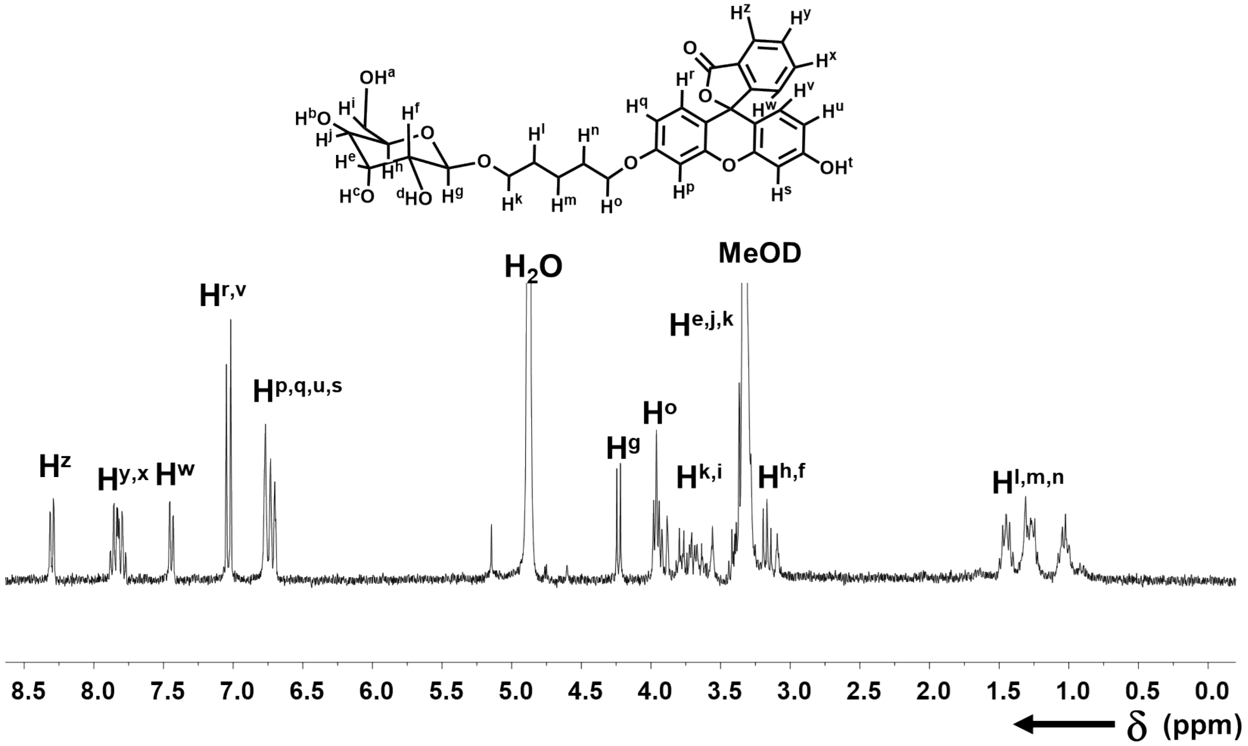

Figure S50. ^{1}H NMR of **β-Gluc-FL** (300 MHz), 25 ^{o}C in MeOD.

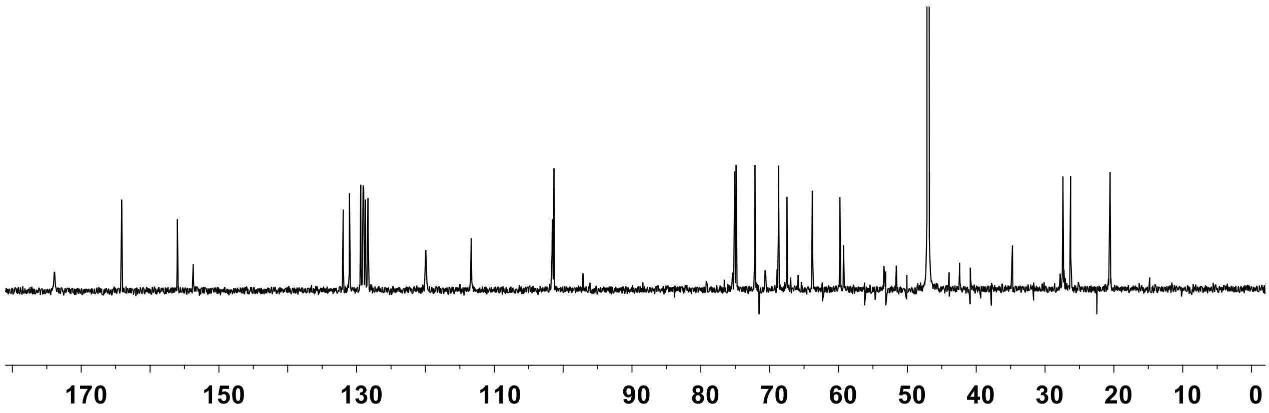

Figure S51. ^{13}C NMR of **β-Gluc-FL** (75 MHz), 25 ^{o}C in MeOD.

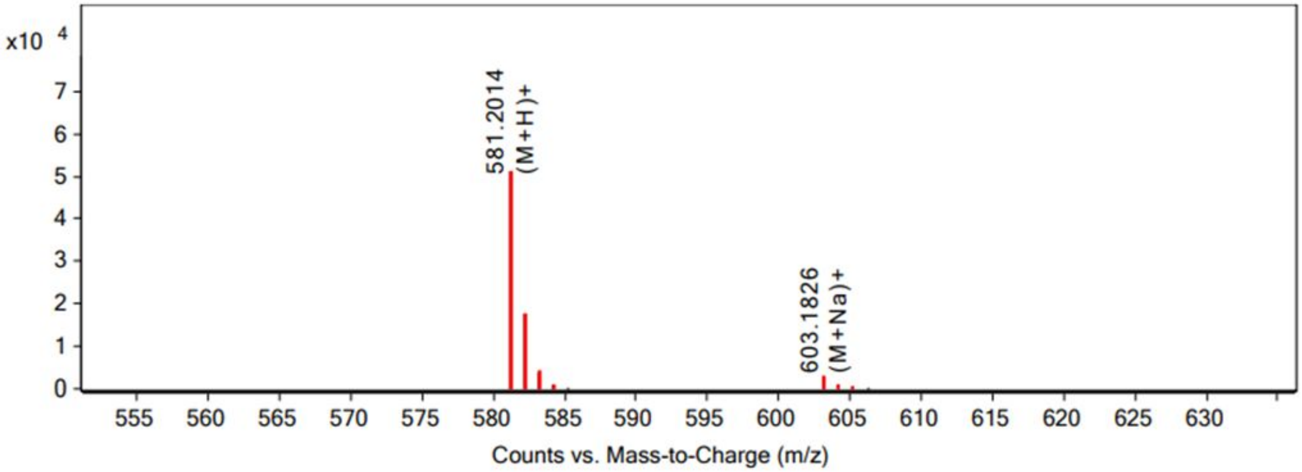

Figure S52. HR-MS of **β-Gluc-FL.**

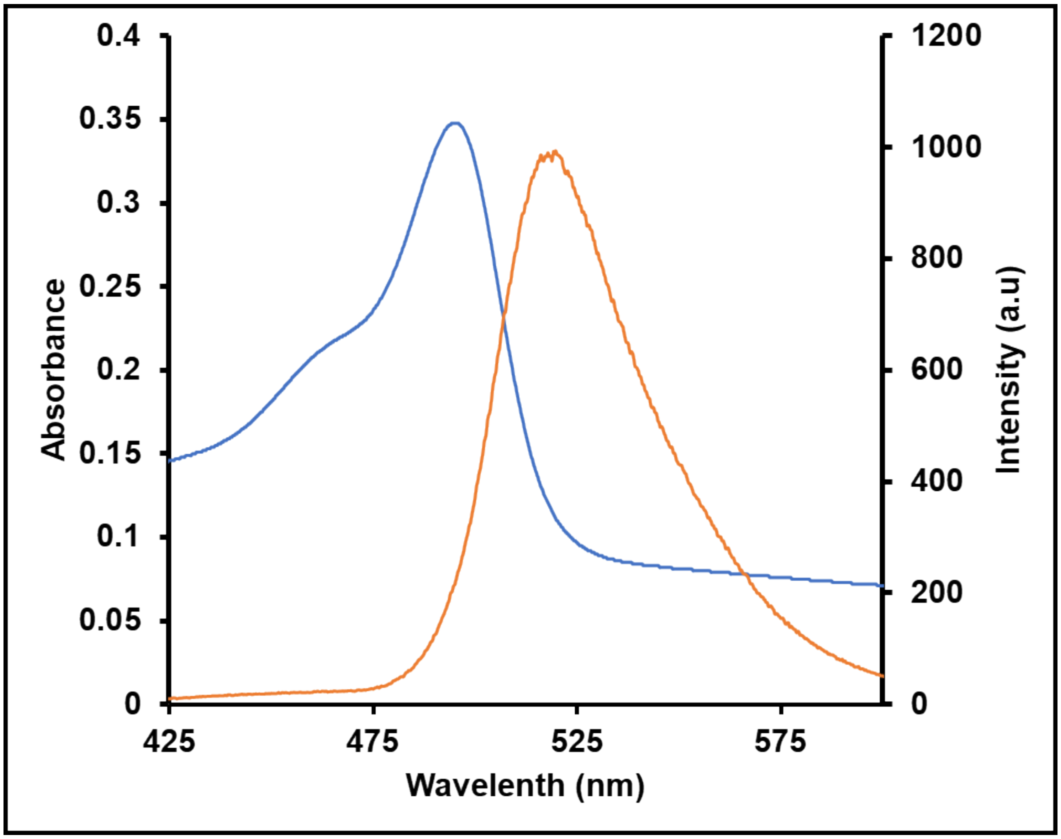

Figure S53. Absorption (blue) and fluorescence emission (orange) spectra (λ_{ex} = 425 nm) of **β-Gluc-FL**. Fluorophore was dissolved in a solution of Tris buffer 20 mM, pH = 7.4, 0.01% Tween20.

2. 2D NMR analysis

To identify the protons of **α-Gal-FL, α-Gluc-FL** and **β-Gluc-FL** ^1H – ^1H COSY, ^1H – ^{13}C HSQC, ^1H – ^{13}C HMBC, and ^1H – ^1H NOESY NMR experiments were performed.

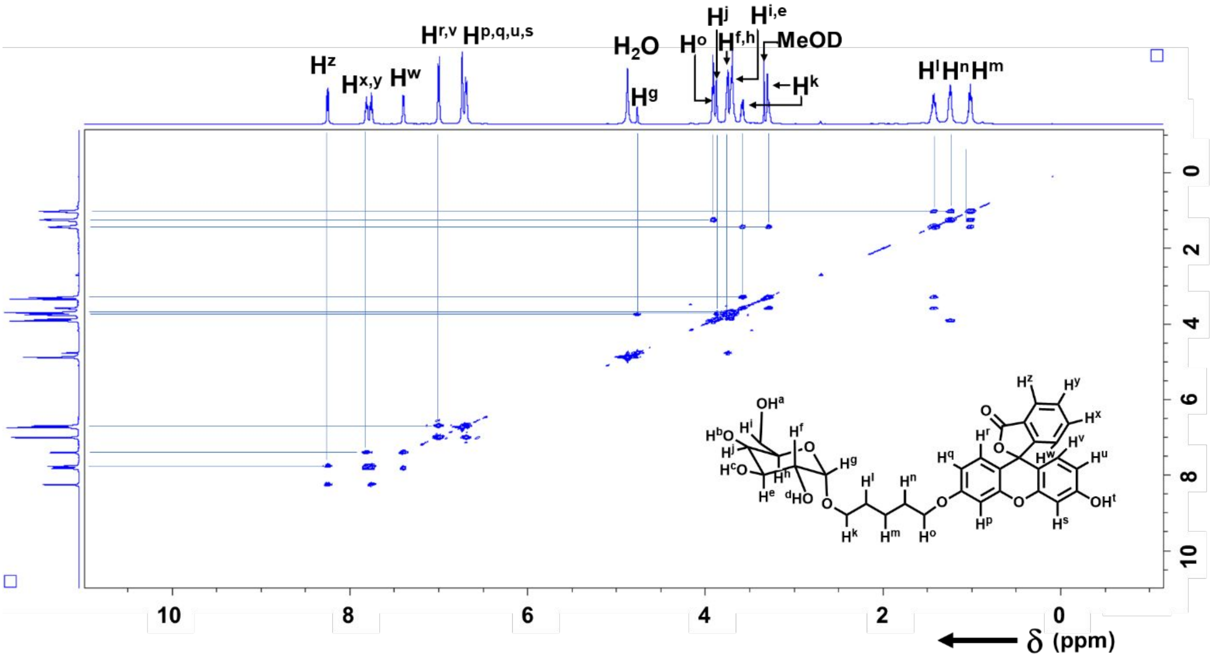

Figure S54. ^1H – ^1H COSY NMR of **α-Gal-FL** (700 MHz) at 25 °C in MeOD.

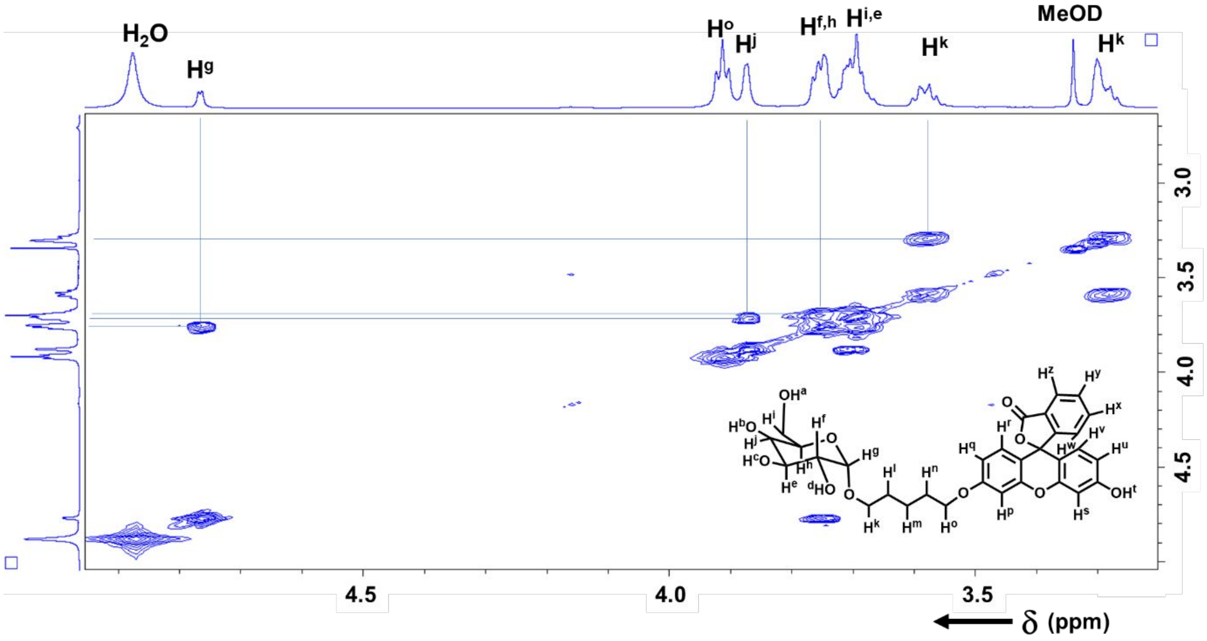

Figure S55. ^1H – ^1H COSY NMR of **α-Gal-FL** (700 MHz) at 25 °C in MeOD. Zoom in of anomeric region.

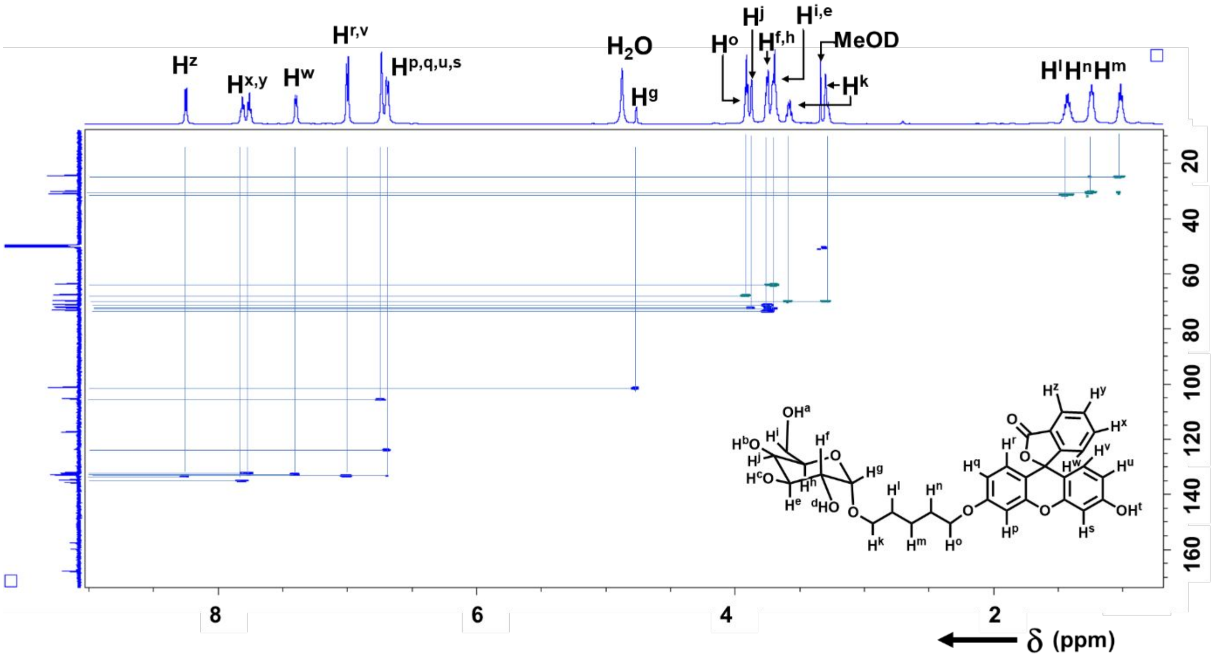

Figure S56. ^{1}H – ^{13}C HSQC NMR of **α-Gal-FL** (700 MHz) at 25 °C in MeOD.

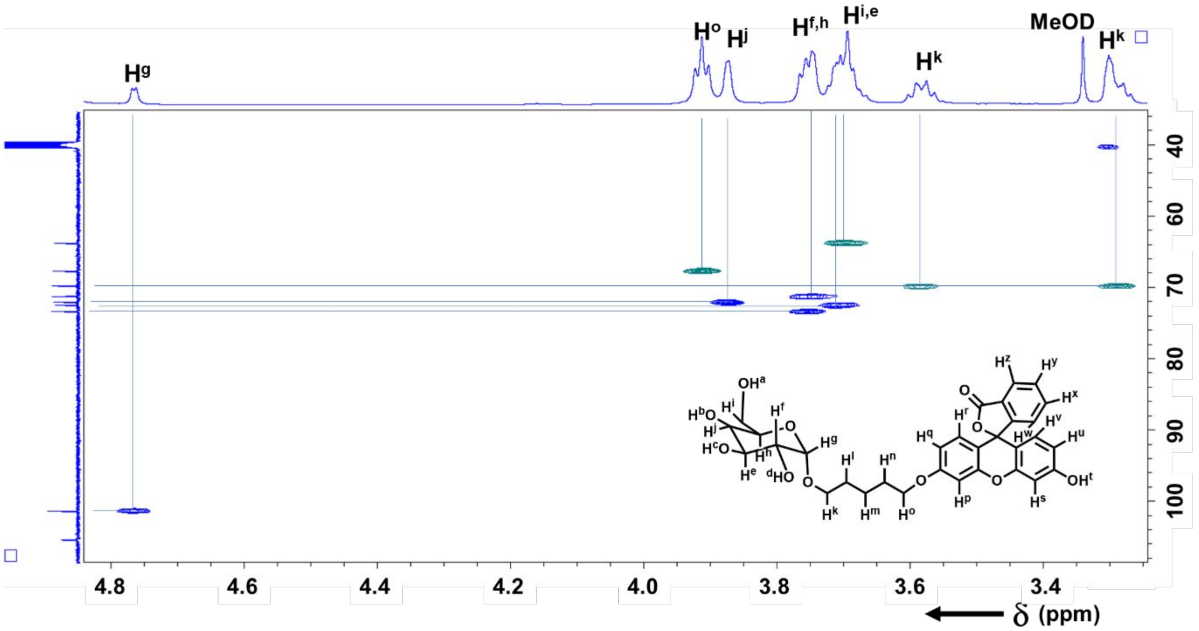

Figure S57. ^{1}H – ^{13}C HSQC NMR of **α-Gal-FL** (700 MHz) at 25 °C in MeOD. Zoom in of anomeric region.

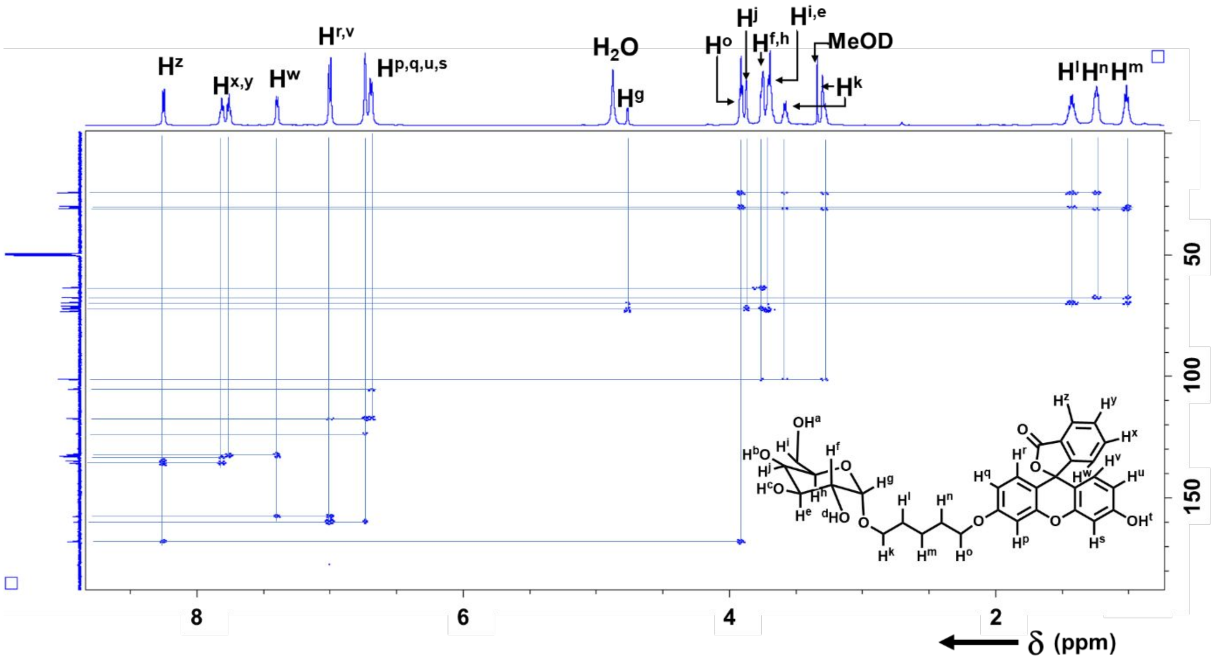

Figure S58. ^{1}H – ^{13}C HMBC NMR of **α-Gal-FL** (700 MHz) at 25 °C in MeOD.

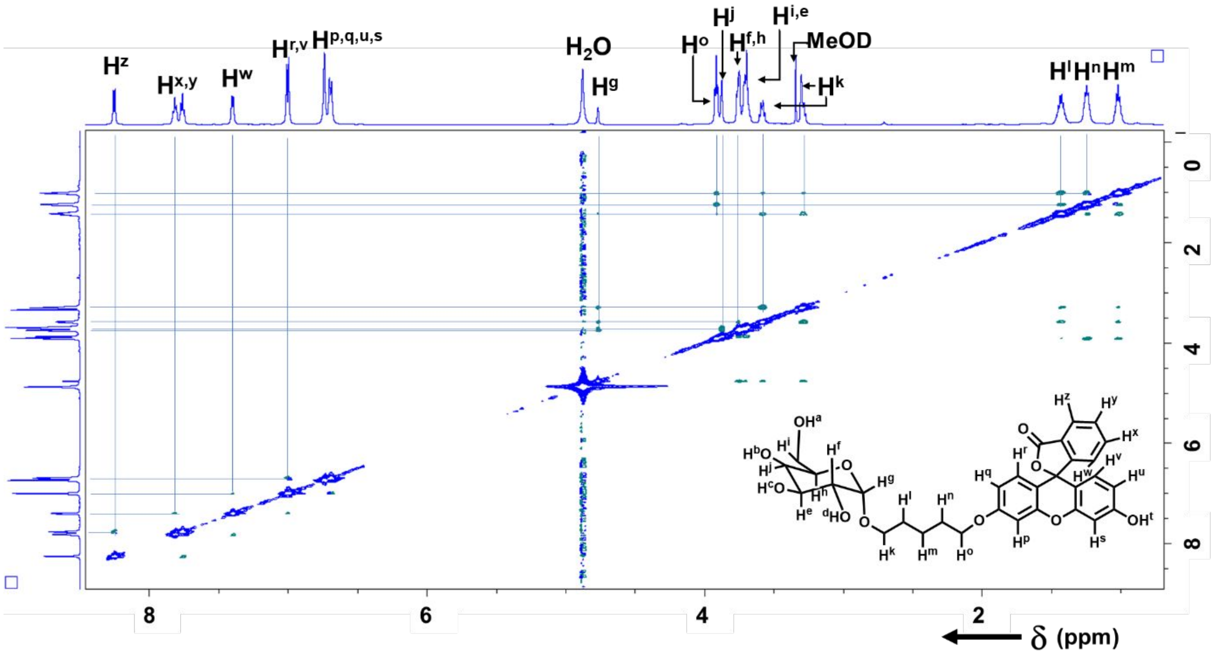

Figure S59. ^{1}H – ^{1}H NOESY NMR of **α-Gal-FL** (700 MHz) at 25 °C in MeOD.

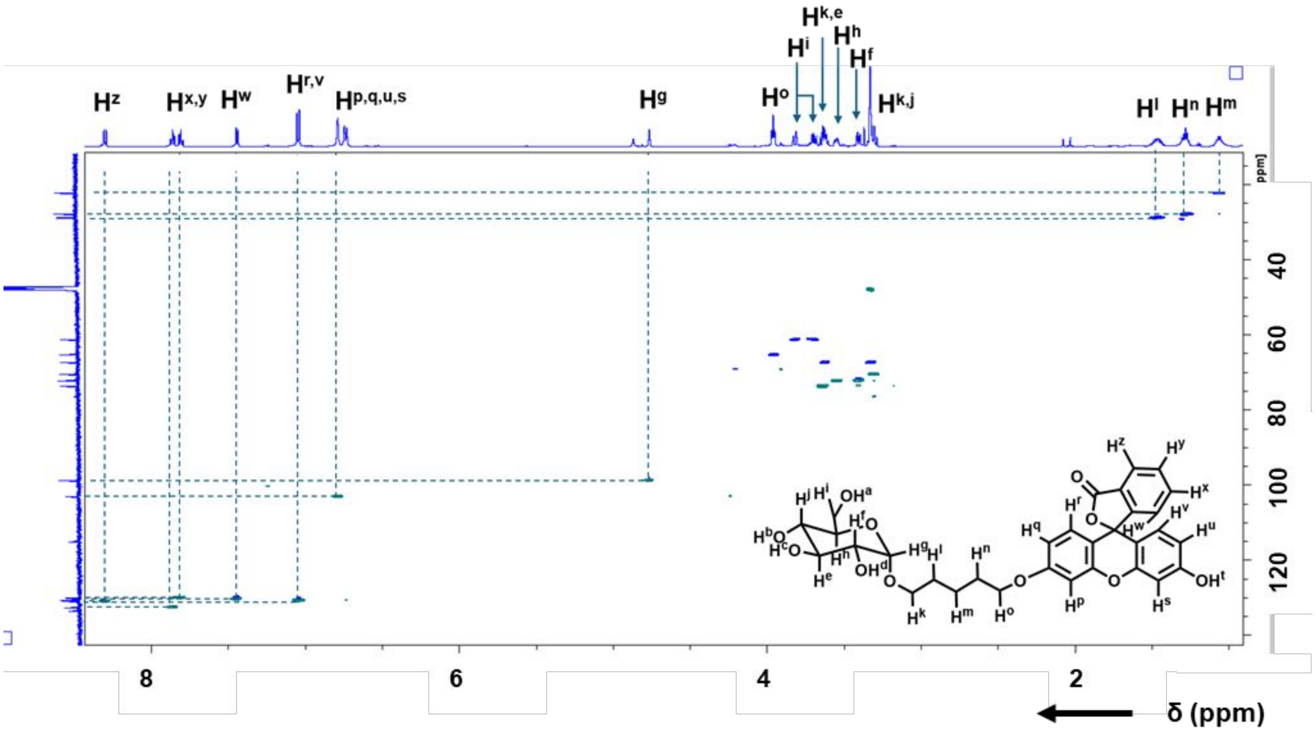

Figure S60*.* ^{1}H – ^{13}C HSQC NMR (600 MHz) at 25 ^{o}C in MeOD of **α-Gluc-FL**

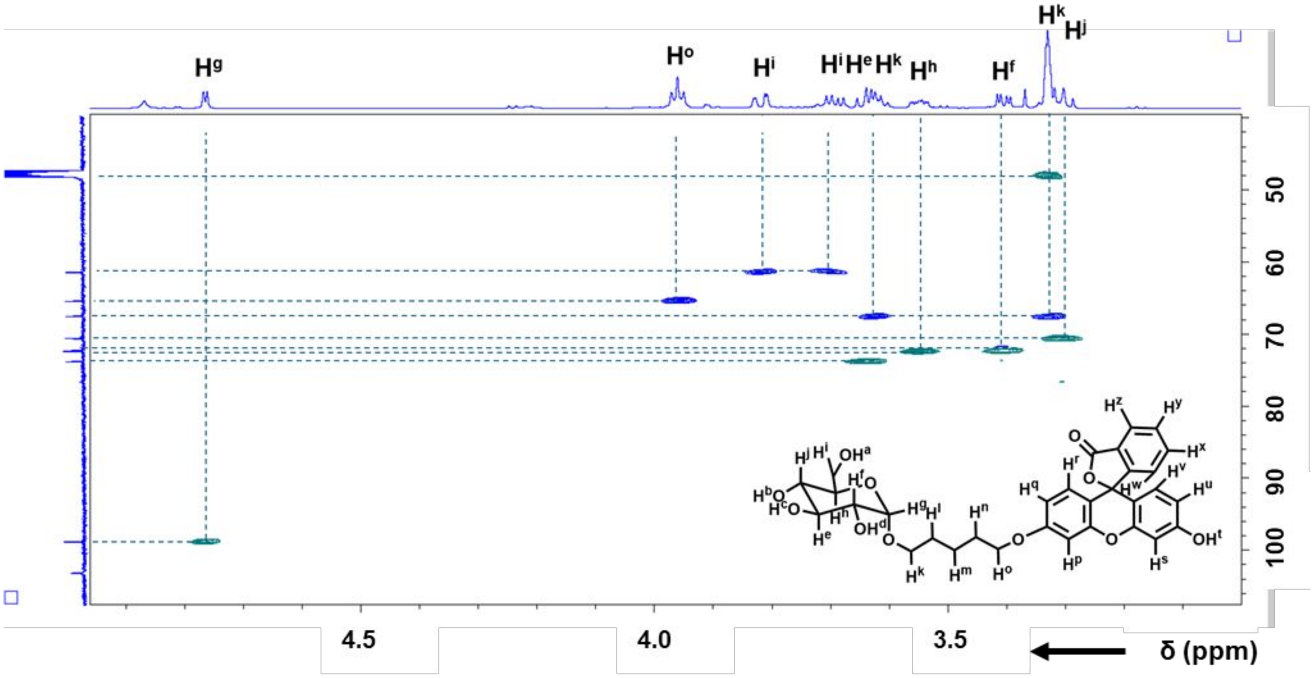

Figure S61*.* ^{1}H – ^{13}C HSQC NMR (600 MHz) at 25 ^{o}C in MeOD of **α-Gluc-FL**

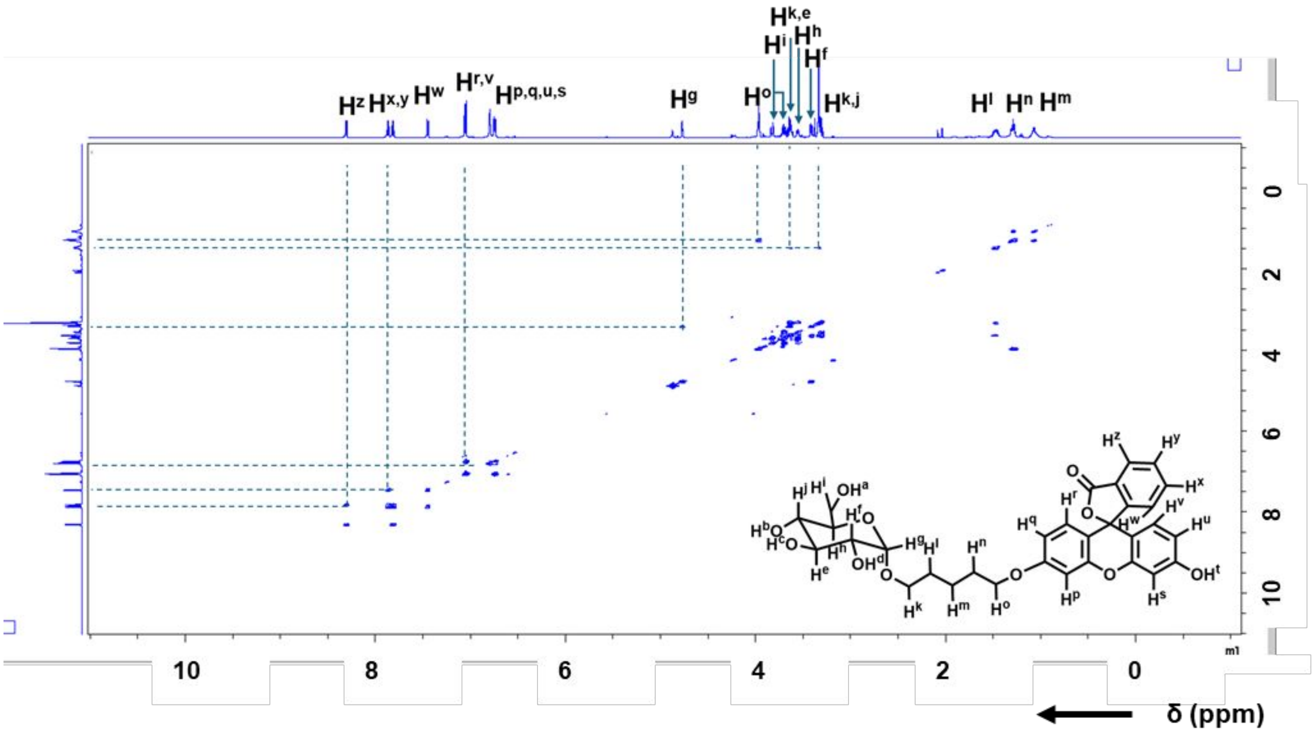

Figure S62. ^{1}H – ^{1}H COSY NMR (600 MHz) at 25 ^{o}C in MeOD of **α-Gluc-FL**

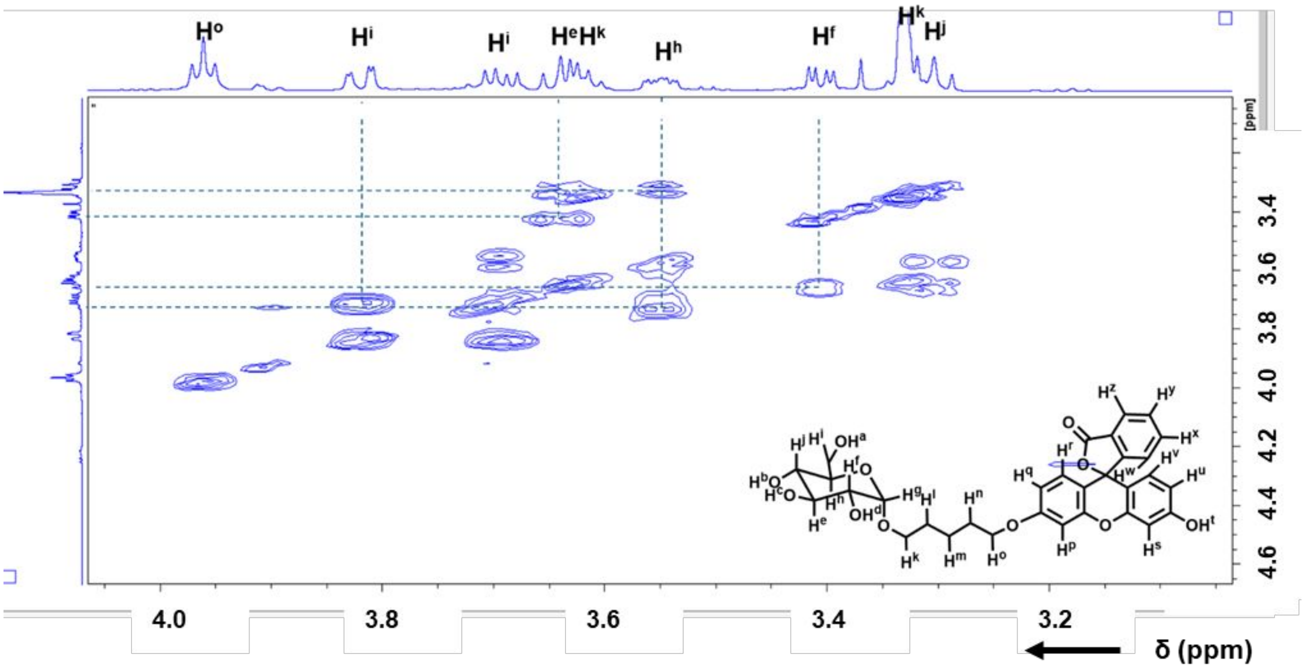

Figure S63. ^{1}H – ^{1}H COSY NMR (600 MHz) at 25 ^{o}C in MeOD of **α-Gluc-FL**

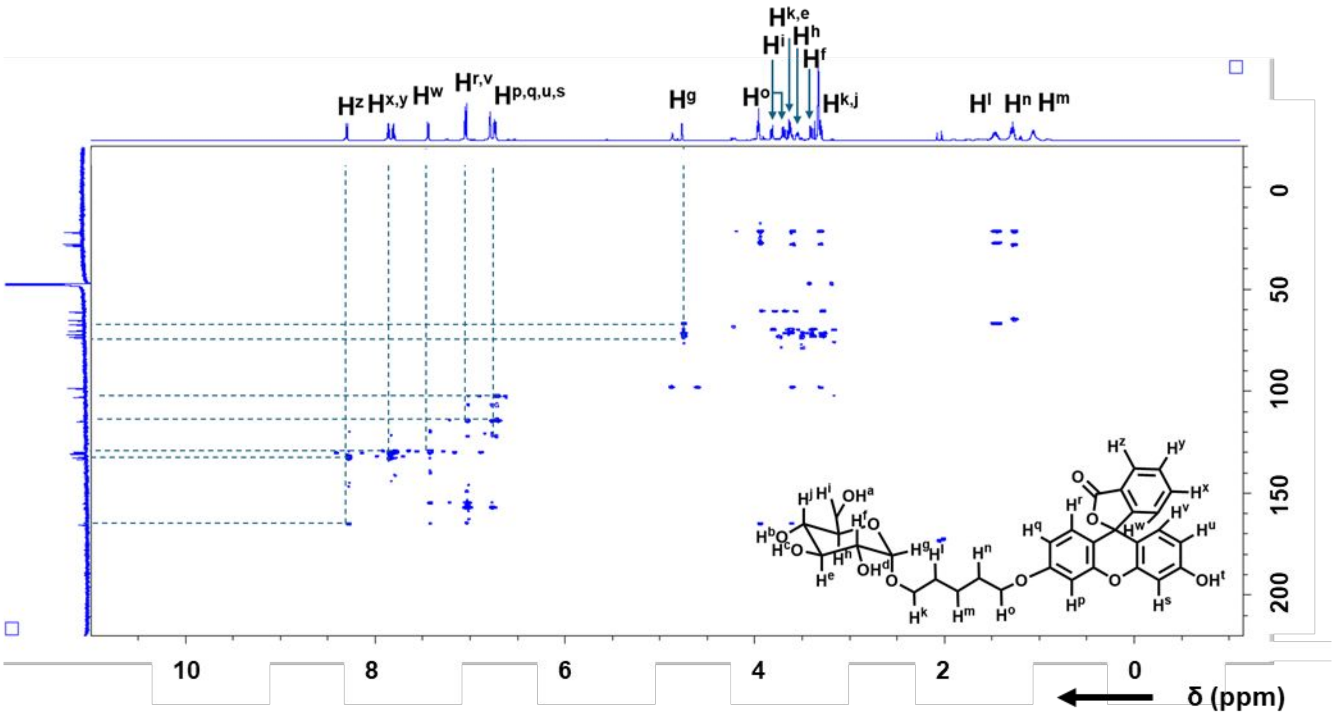

Figure S64. ^1H – ^{13}C HMBC NMR (600 MHz) at 25 °C in MeOD of **α-Gluc-FL**

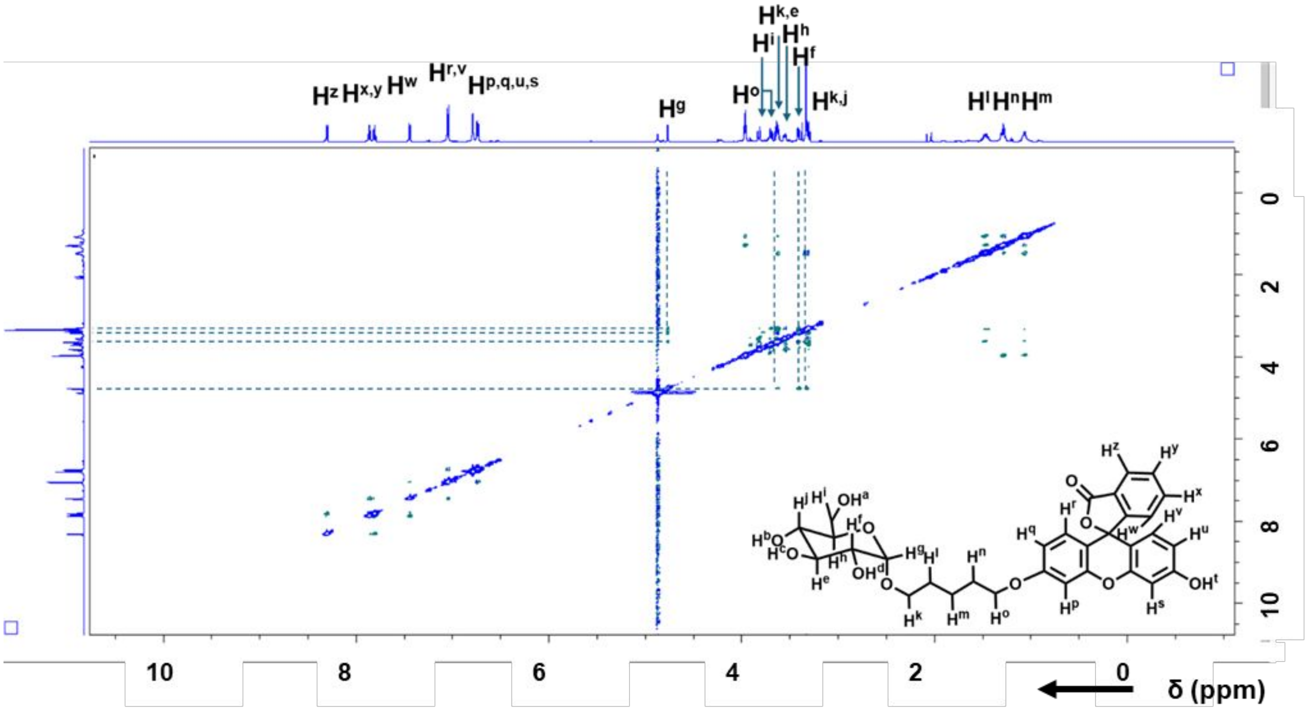

Figure S65. ^1H – ^1H NOESY NMR (600 MHz) at 25 °C in MeOD of **α-Gluc-FL**

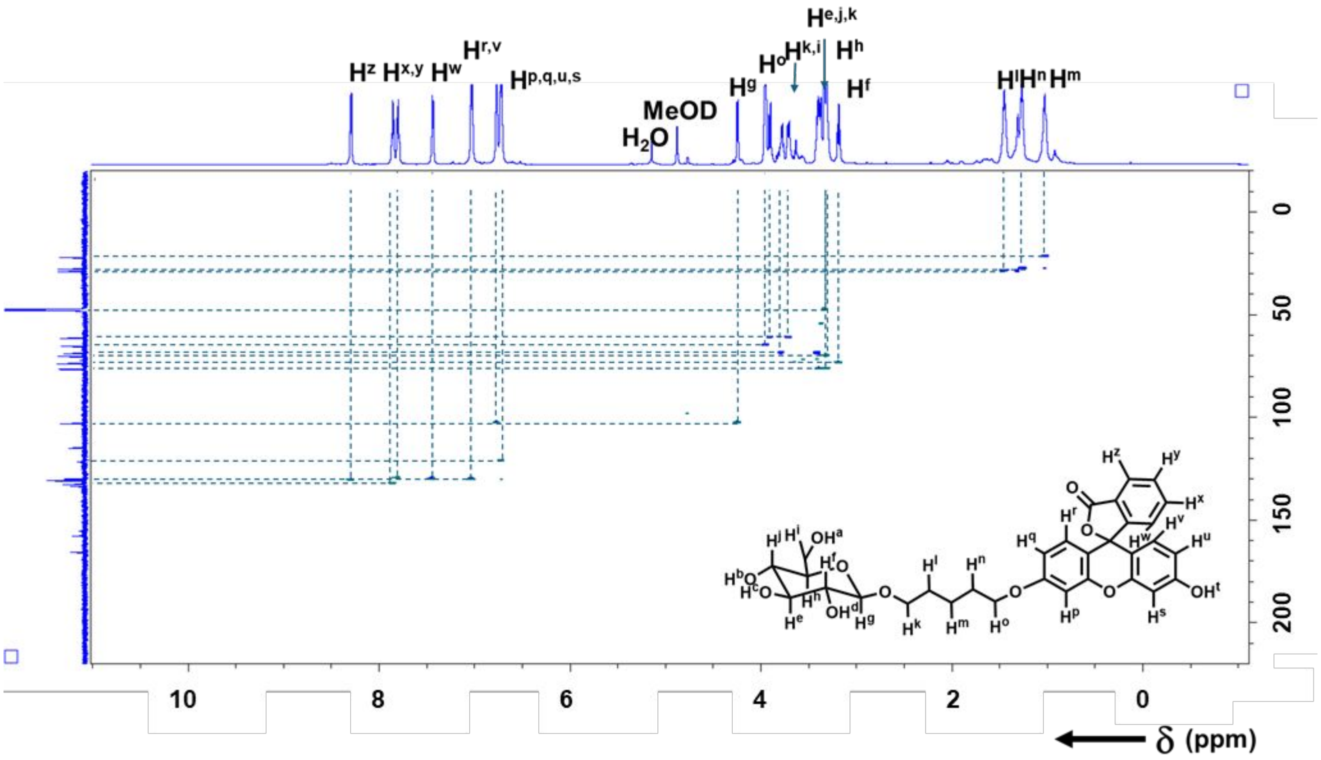

Figure S66. ^1H – ^{13}C HSQC NMR of **β-Gluc-FL**

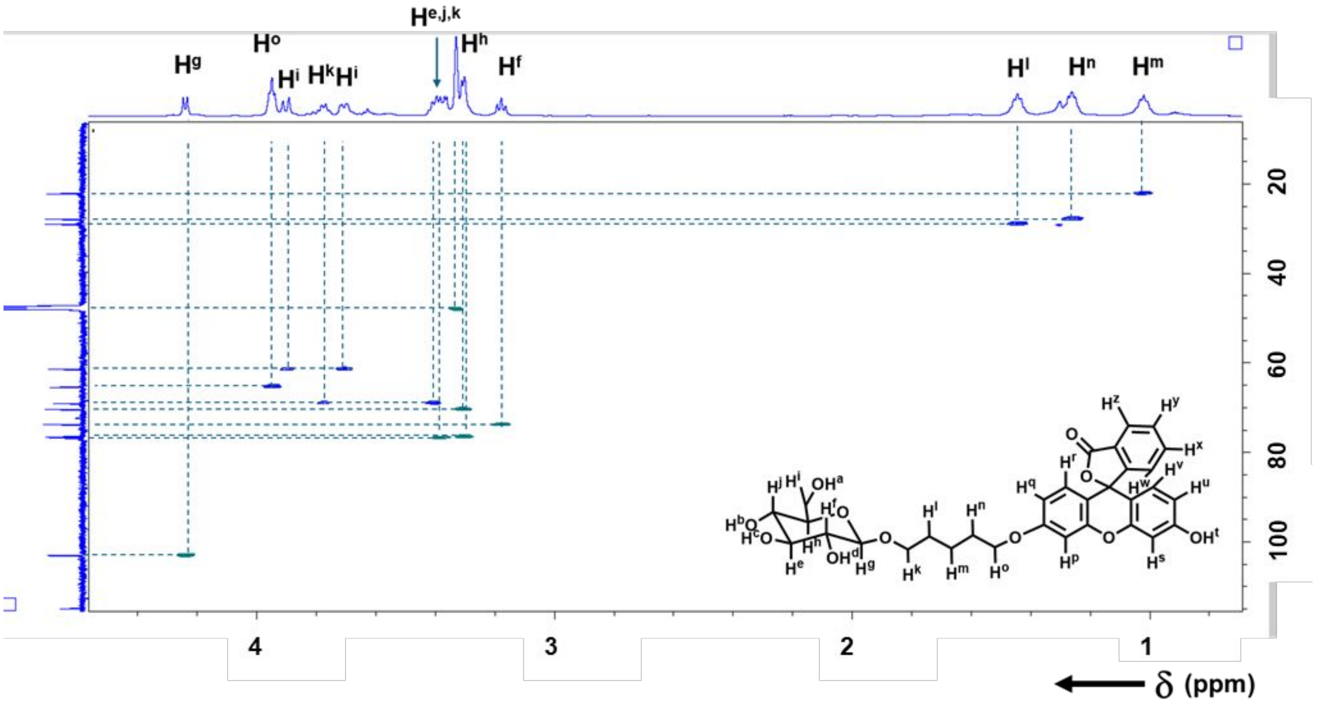

Figure S67. ^1H – ^{13}C HSQC NMR of **β-Gluc-FL**

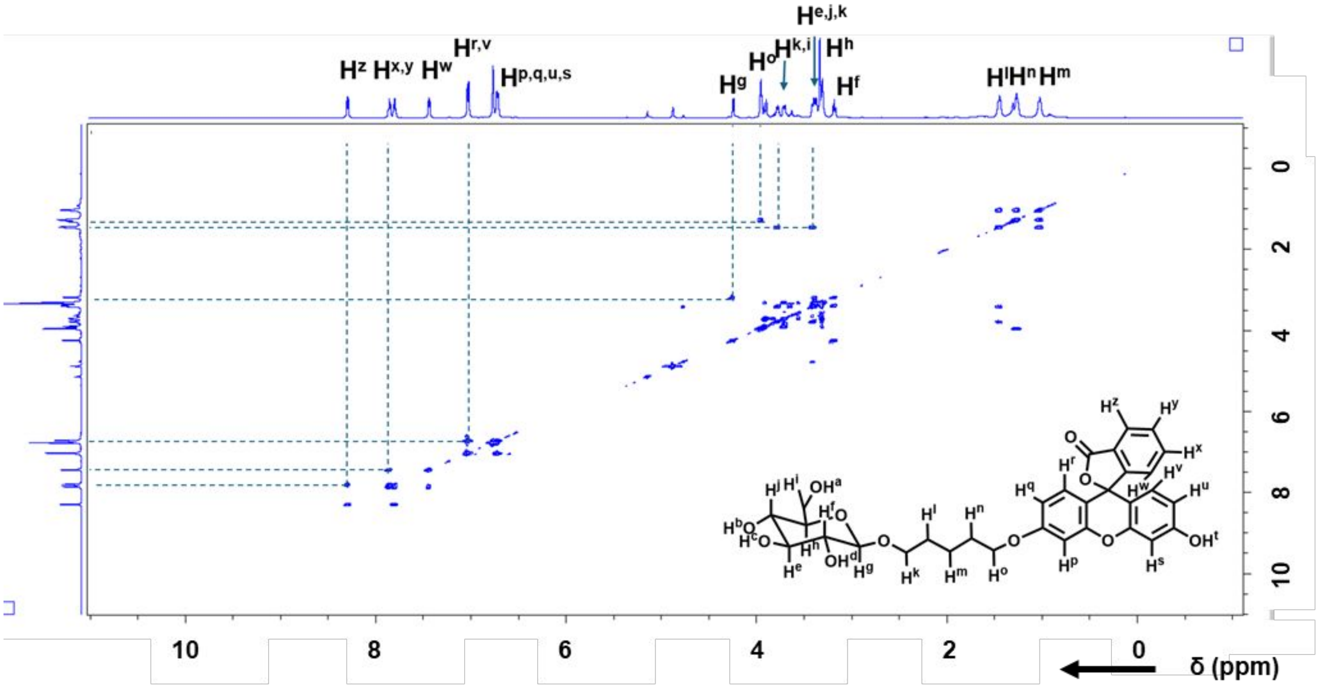

Figure S68. ^{1}H – ^{1}H COSY NMR (600 MHz) at 25 °C in MeOD of **β-Gluc-FL**

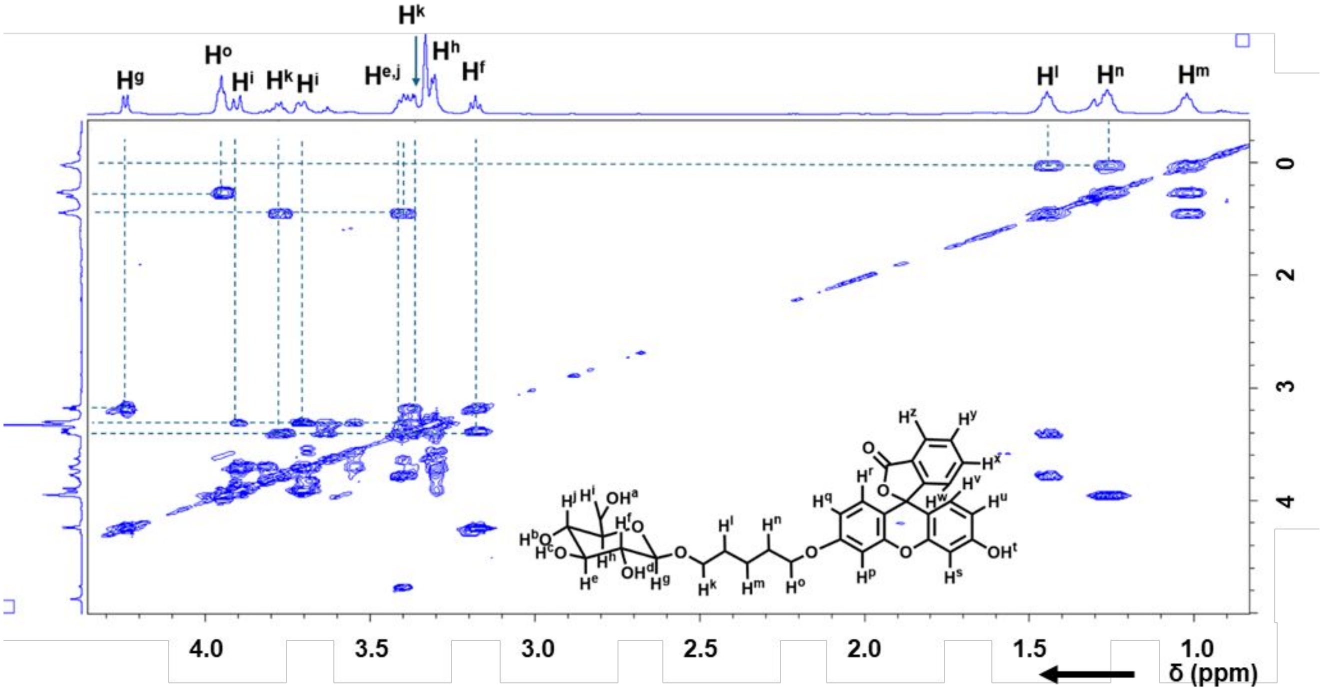

Figure S69. ^{1}H – ^{1}H COSY NMR (600 MHz) at 25 °C in MeOD of **β-Gluc-FL**

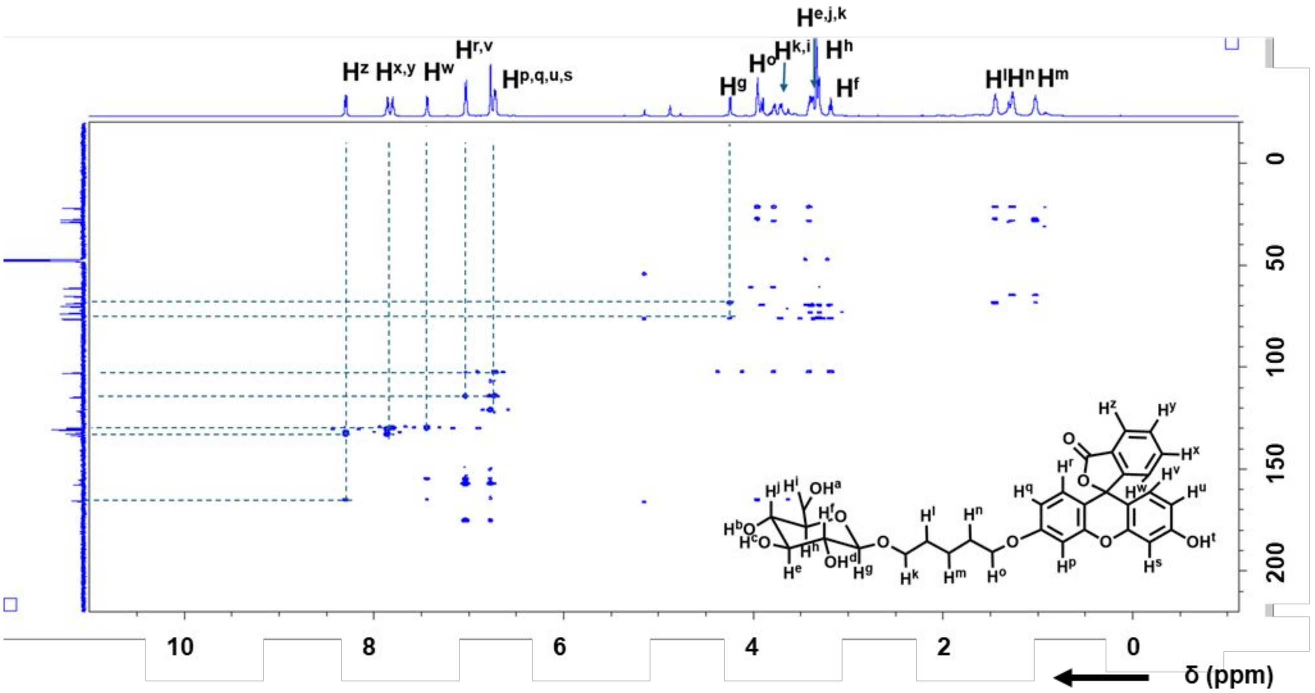

Figure S70. ^{1}H – ^{13}C HMBC NMR (600 MHz) at 25 ^{o}C in MeOD of **β-Gluc-FL**

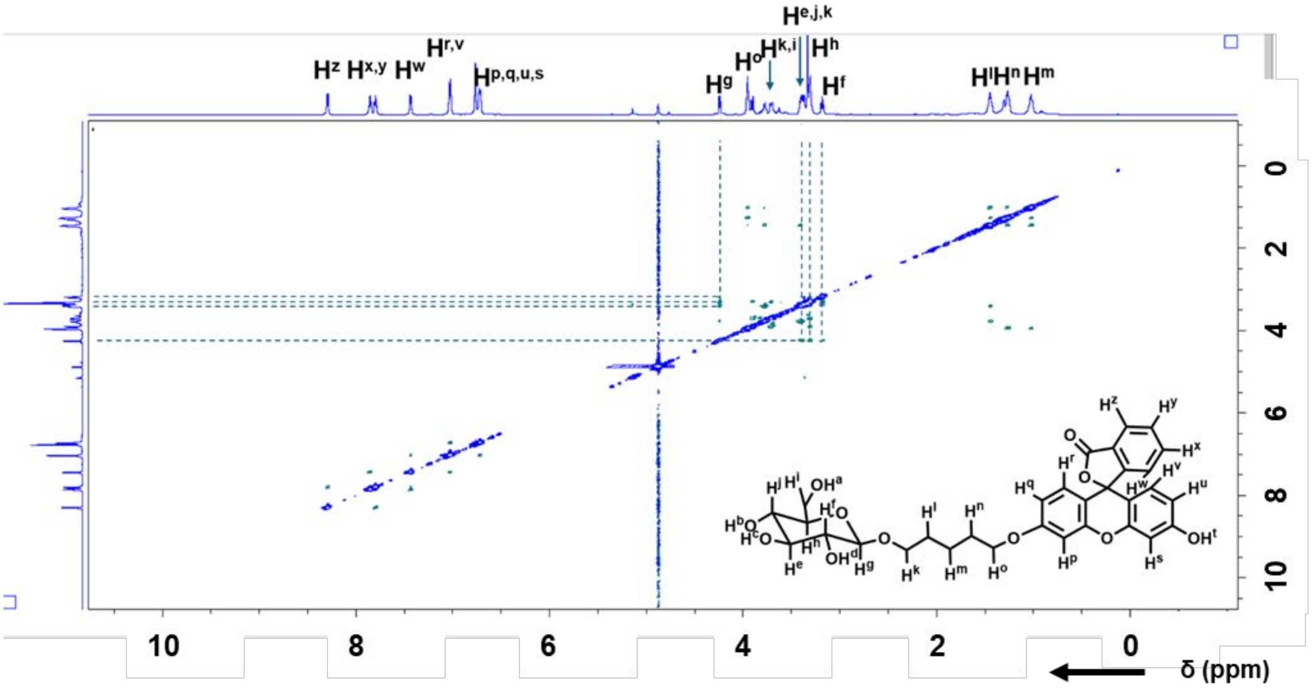

Figure S71. ^{1}H – ^{1}H NOESY NMR (600 MHz) at 25 ^{o}C in MeOD of **β-Gluc-FL**

3. Preparation of thiol-terminated monolayers

Thiol-terminated Si<100> substrates were prepared according to previously published procedures[3-6]. Briefly, Si<100> wafers with 500 nm oxide layer were purchased from NOVA Electronic Materials, USA (STK8414-OX), (3-mercaptopropyl)trimethoxysilane was purchased from Gelest (SIM6476.0-100GM), and all other chemicals were purchased from VWR. The wafers were diced into appropriately sized slides and cleaned by submerging in piranha solution (3:1 H_2SO_4:H_2O_2) for 15 min and then rinsing with MilliQ H_2O. Once dried, the wafers were placed in a 120 mL PhMe solution containing 4.5 mL (0.10 mol, 0.02 M) of (3-mercaptopropyl)trimethoxysilane and were heated in this solution to 37 °C in a H_2O bath for 4 h. The Si wafers were then rinsed with PhMe, a 50/50 (v/v) mixture of PhMe and EtOH, and then EtOH. Finally, the Si substrates were cured in an oven at 105 °C for 18 h and stored in MeOH at 4 °C until used (**Scheme S7**). To analyze the surface monolayer, XPS measurements were performed with a Physical Electronics VersaProbe II XPS using an Al monochromatic X-ray source (1486.6 eV) at 37.6 W and neutralizer gun operating at 2.0 V and 20 µA. Beam diameter was set to 200 µm, time per data point to 10 s at intervals of 0.125 eV, and pass energy at 29.35 eV and 20 scans for S2p. The S2p peak at 164 eV is evidence of thiol-terminated substrate formation (**Figure S72**).

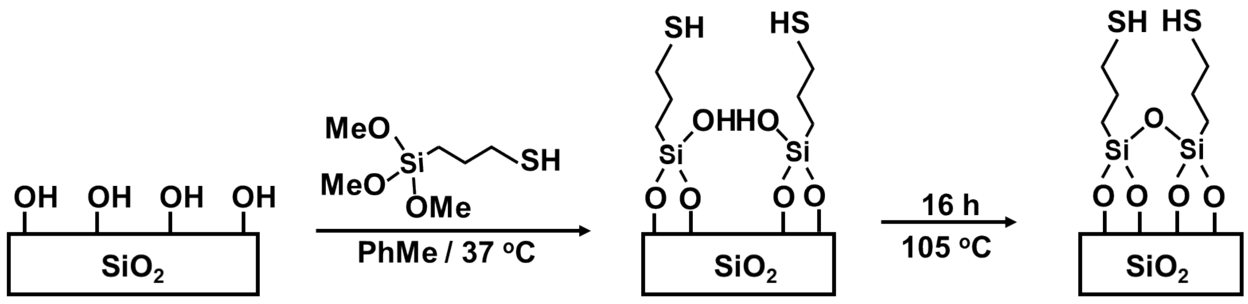

Scheme S7. Preparation of thiol-terminated substrates.

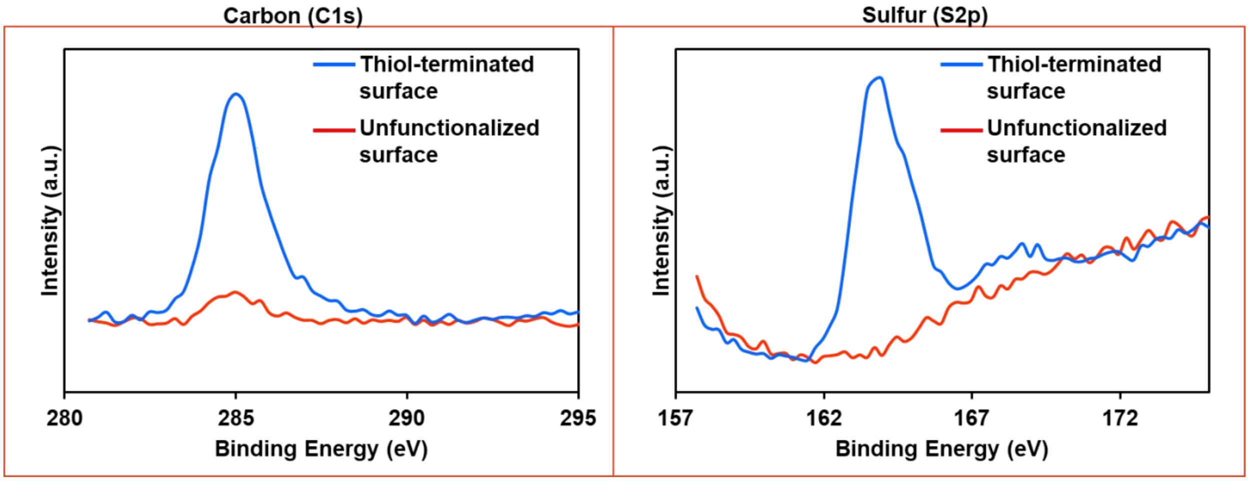

Figure S72. XPS data of unfunctionalized Si/SiO_2 surface *vs* thiol-terminated Si/SiO_2 surface**.**

4. Description of the printer.

The Hypersurface Photolithography printer used in these experiments was described previously,[2-6] and integrates microfluidics, a CPU-controlled digital micromirror device (DMD) with ~700,000 individually actuatable mirrors, and surface photochemistry[2, 7] to pattern **SCR043** microarrays with micrometer-scale feature edge-lengths and independent control over the feature height and **SCR043** grafting density (Γ([**SCR043**]) at each pixel. The LED, DMD, and piezo-stage are components of a TERA-Fab Elite or Elite instrument (TERA Print, LLC, USA). The DMD is equipped with a 1024 x 768 array of mirrors that can be individually turned on and off by a CPU to spatiotemporally control delivery of the LED light (405 nm, 1.16 mW/mm^2) to the substrate to create patterns. Light intensities were measured using a ThorLabs power meter (PM100D) with all DMD mirrors on and dividing by the illumination area (4.4 x 3.3 mm). The reaction occurs in a custom-built fluid cell with a glass coverslip to allow for passage of light to the reactive substrate below. Reaction solutions are delivered via micropipette tip delivered directly onto the surface within the glovebox before being sealed in an inert chamber. The substrate sits upon an *x, y, z* piezo-stage that has fine motor precision of 1 μm. The light intensity at each pixel is spatiotemporally modulated to create hypersurfaces and is controlled by tera-e-v2.0 software.

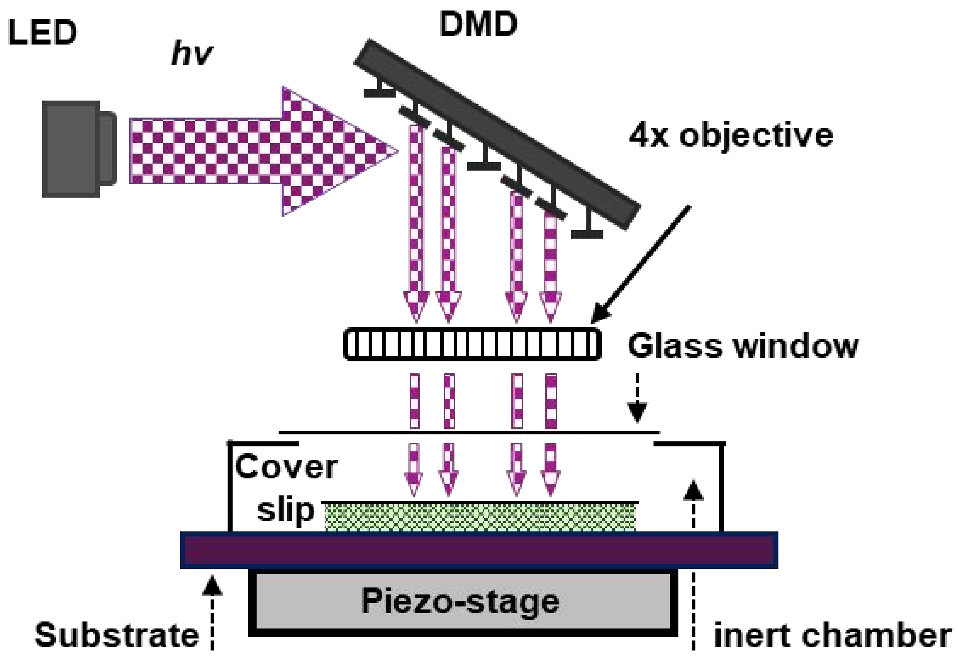

Figure S73. Photochemical printer for performing hypersurface photolithography.

5. Printing optimization of SCR043 functionalized polymer brushes

General methods

Grafted-to grafted-from radical photopolymerization (GTGFRP) reactions[2-6] from thiol-terminated SiO_2 surfaces to create **SCR043-**functionalized polymer brushes were performed with a DMSO solution containing **SCR043**, pentaerythritol tetrakis(3-mercaptopropionate) (PETT, Millipore Sigma, 381462-100ML), ethylene glycol dimethacrylate (EGDMA, Alfa Aesar, 44151-30), diphenyl(2,4,6 trimethylbenzoyl)phosphine oxide (TPO, TCI, D3358-25G), dimethyl sulfoxide (DMSO, Alfa Aesar) that were all used as received, with the exception of EGDMA, which was run through an alumina column prior to use to remove the inhibitor. Solutions were introduced to the surface via drop-casting for all experiments. All solutions were prepared in a glovebox; therefore no degassing techniques were utilized during the process. First, the polymerization reactions were carried out in the absence of **SCR043**. Then, the concentrations of **SCR043** and light intensities were varied to investigate the growth rates of the polymer brushes. After patterning the polymer brushes, each surface was washed with EtOH (~5 mL), dried under a stream of air, and characterized by profilometry on a Bruker Dektak-XT stylus profiler. Surfaces were mapped with Surface Mapping mode using a 12.5 μm tip, 1 mg of contact force, 120 scans, and Hills and Valleys setting. Profilometry was analyzed using in-house designed python script. (See pages S248-S261).

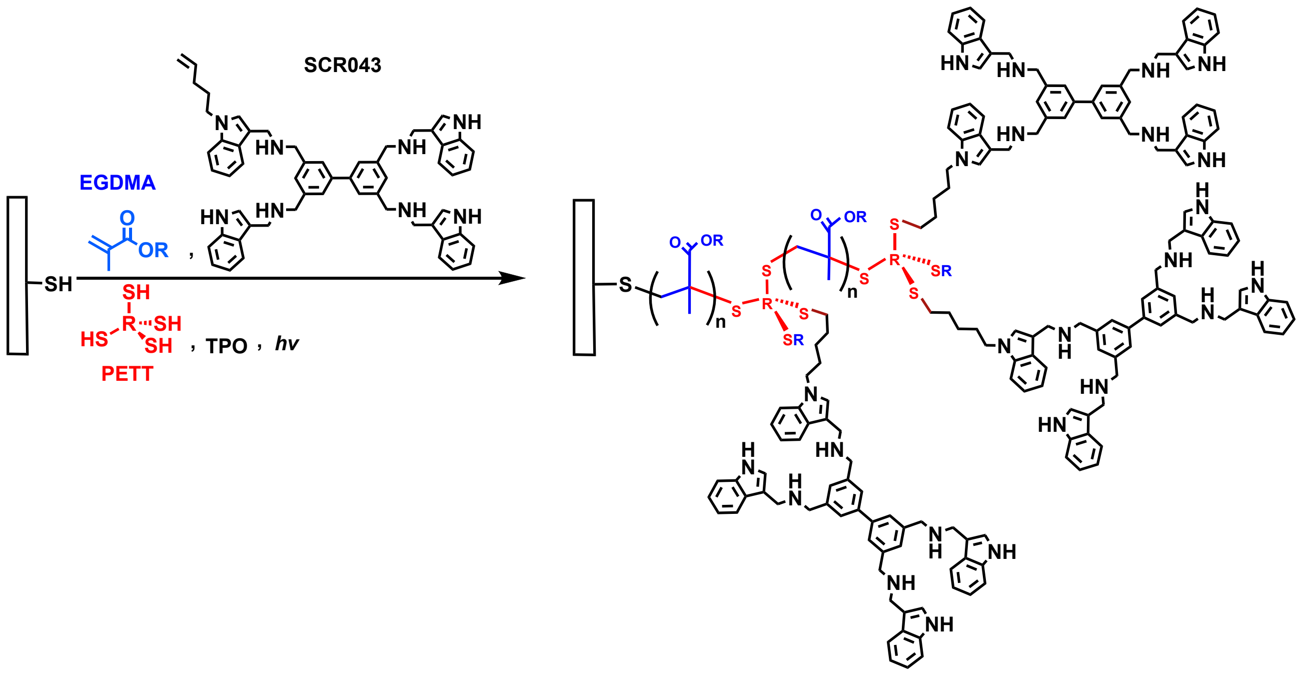

Scheme S8. GTGFRP reaction to create **SCR043-**functionalized polymer brushes from thiol-terminated Si/SiO_2 surfaces.

Table S1. Reaction conditions for studying how varying *hv* (light intensity) affects the growth rate of **SCR043-**functionalized polymer brushes.

Prints	TPO (mM)	PETT (mM)	EGDMA (mM)	Intensity (mW/mm^2)	[**SCR043**] (µM)
A	1	100	1300	1.27	500
B	1	100	1300	2.53	500
C	1	100	1300	3.80	500
D	1	100	1300	6.33	500

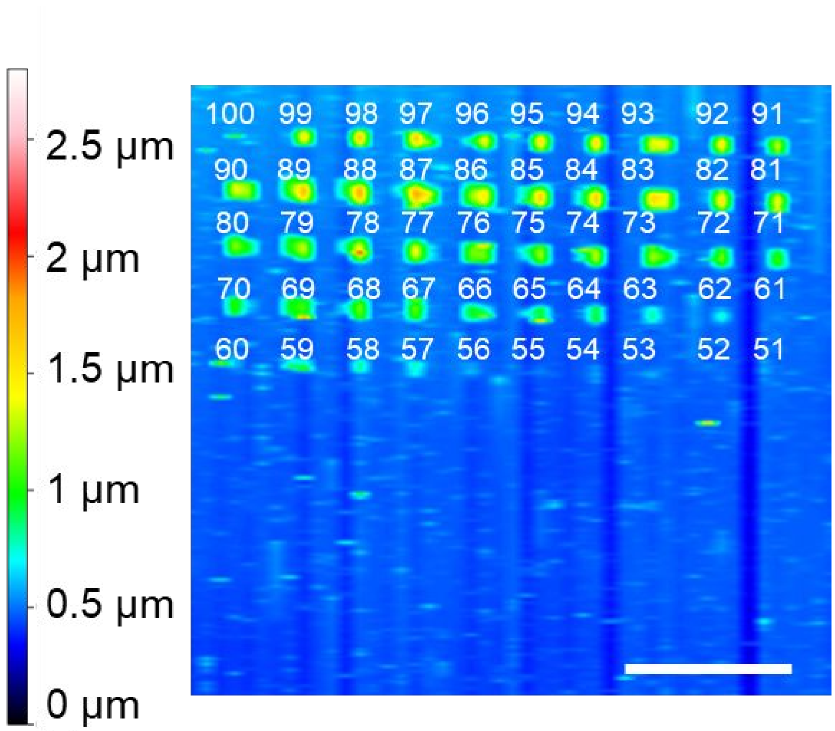

Figure S74. Profilometry image of patterns printed under conditions described in **Table S1A**, *hv* = 1.27 mW/mm^2. The scale bar is 200 µm.

Table S2. Heights of features printed under conditions in **Table S1A**. All heights and standard deviations are reported as averages of four repeats per point for all features at 4.5-seconds intervals of exposure time. No polymer growth was observed in feature(s) preceding the one reported in this table.

Feature number	Time (min)	Height (μm)	Standard deviation	Feature number	Time (min)	Height (μm)	Standard deviation
51	8.1	0.1	0.1	76	12.0	1.0	0.2
52	8.2	0.1	N/A	77	12.2	0.8	0.1
53	8.4	0.1	0.0	78	12.4	1.0	0.5
54	8.6	0.1	0.1	79	12.5	0.9	0.2
55	8.7	0.1	0.1	80	12.7	0.7	0.1
56	8.9	0.1	0.1	81	12.8	0.9	0.2
57	9.0	0.3	0.0	82	13.0	1.2	0.4
58	9.2	0.3	0.0	83	13.1	1.1	0.3
59	9.3	0.6	0.2	84	13.3	1.2	0.3
60	9.5	0.1	0.1	85	13.5	1.1	0.2
61	9.7	0.1	0.0	86	13.6	1.1	0.2
62	9.8	0.4	0.1	87	13.8	1.3	0.4
63	10.0	0.4	0.0	88	13.9	1.5	0.4
64	10.1	0.5	0.2	89	14.1	1.2	0.1
65	10.3	0.9	0.3	90	14.3	0.9	0.2
66	10.5	0.9	0.3	91	14.4	0.5	0.3
67	10.6	0.8	0.2	92	14.6	1.0	0.2
68	10.8	0.6	0.1	93	14.7	1.1	0.1
69	10.9	0.9	0.5	94	14.9	1.1	0.1
70	11.1	0.6	0.1	95	15.0	1.1	0.1
71	11.2	0.8	0.3	96	15.2	1.1	0.2
72	11.4	0.8	0.1	97	15.4	1.1	0.1
73	11.6	1.3	0.7	98	15.5	1.2	0.1
74	11.7	1.2	0.7	99	15.7	1.2	0.1
75	11.9	1.0	0.3	100	15.8	0.7	0.4

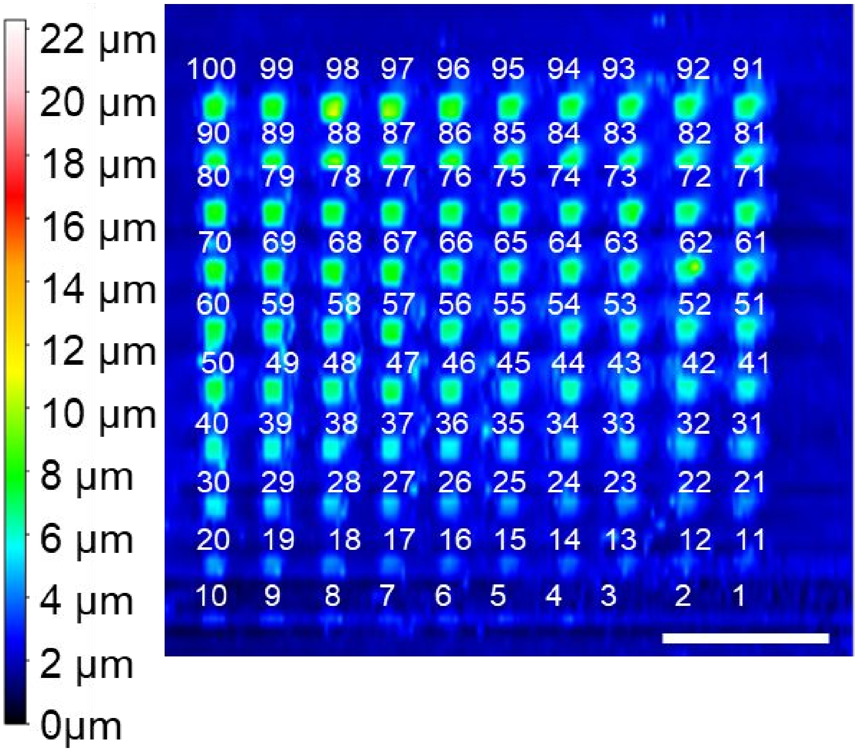

Figure S75. Profilometry image of patterns printed under conditions described in **Table S1B**, *hv* = 2.53 mW/mm^2. The scale bar is 200 µm.

Table S3. Heights of features printed under conditions in **Table S1B**. All heights and standard deviations are reported as averages of four repeats per point for all features at a 4.5-second intervals of exposure time. No polymer growth was observed in feature(s) preceding the one reported in this table.

Feature number	Time (min	Height (µm)	Standard deviation	Feature number	Time (min	Height (µm)	Standard deviation
2	0.3	1.1	N/A	51	8.1	5.8	0.8
3	0.5	0.9	N/A	52	8.2	6.5	1.8
4	0.6	1.0	0.8	53	8.4	6.9	1.8
5	0.8	1.6	0.1	54	8.6	7.3	2.1
6	1.0	1.4	0.3	55	8.7	7.1	2.3
7	1.1	1.5	0.6	56	8.9	7.0	1.5
8	1.3	1.7	0.7	57	9.0	7.1	1.0
9	1.4	1.9	0.5	58	9.2	6.6	0.9
10	1.6	2.3	0.8	59	9.3	8.8	3.5
11	1.7	2.2	0.5	60	9.5	6.5	1.1
12	1.9	2.3	0.7	61	9.7	6.3	0.8
13	2.1	2.6	0.7	62	9.8	7.0	1.2
14	2.2	4.1	3.0	63	10.0	7.1	1.7
15	2.4	2.8	0.7	64	10.1	7.5	1.8
16	2.5	2.8	0.6	65	10.3	7.4	1.5
17	2.7	2.9	0.5	66	10.5	8.0	2.4
18	2.9	2.8	0.6	67	10.6	11.7	4.6
19	3.0	3.2	0.8	68	10.8	9.3	4.5
20	3.2	3.3	0.6	69	10.9	11.1	5.5
21	3.3	3.3	0.4	70	11.1	9.3	5.2
22	3.5	3.3	0.4	71	11.2	8.3	1.9
23	3.6	4.1	1.2	72	11.4	7.3	2.1
24	3.8	3.5	0.5	73	11.6	10.5	3.1
25	4.0	3.7	0.6	74	11.7	8.3	2.6
26	4.1	3.8	0.7	75	11.9	8.6	2.6
27	4.3	3.9	0.7	76	12.0	13.3	5.6
28	4.4	4.1	0.6	77	12.2	13.4	6.1
29	4.6	4.3	0.7	78	12.4	11.8	5.6
30	4.8	5.2	1.6	79	12.5	12.4	6.1
31	4.9	4.4	0.6	80	12.7	9.8	7.0
32	5.1	5.8	2.4	81	12.8	7.2	1.0
33	5.2	4.7	0.8	82	13.0	7.7	3.3
34	5.4	4.3	0.8	83	13.1	9.8	4.9
35	5.5	4.6	0.7	84	13.3	10.1	5.4
36	5.7	4.7	1.0	85	13.5	11.1	5.6
37	5.9	4.8	1.0	86	13.6	13.0	6.3
38	6.0	4.9	0.8	87	13.8	13.3	6.8
39	6.2	5.0	0.7	88	13.9	12.2	4.9
40	6.3	5.3	0.7	89	14.1	11.7	5.8
41	6.5	5.1	0.8	90	14.3	9.9	6.5
42	6.7	5.4	1.3	91	14.4	8.1	2.1
43	6.8	5.7	1.9	92	14.6	8.2	1.9
44	7.0	6.5	2.2	93	14.7	10.2	5.4
45	7.1	5.5	1.0	94	14.9	11.5	5.2
46	7.3	5.5	0.8	95	15.0	11.7	4.9
47	7.4	5.9	0.5	96	15.2	13.6	7.1
48	7.6	5.7	0.9	97	15.4	13.7	6.0
49	7.8	6.0	0.8	98	15.5	11.5	4.8
50	7.9	6.1	0.8	99	15.7	11.1	5.0

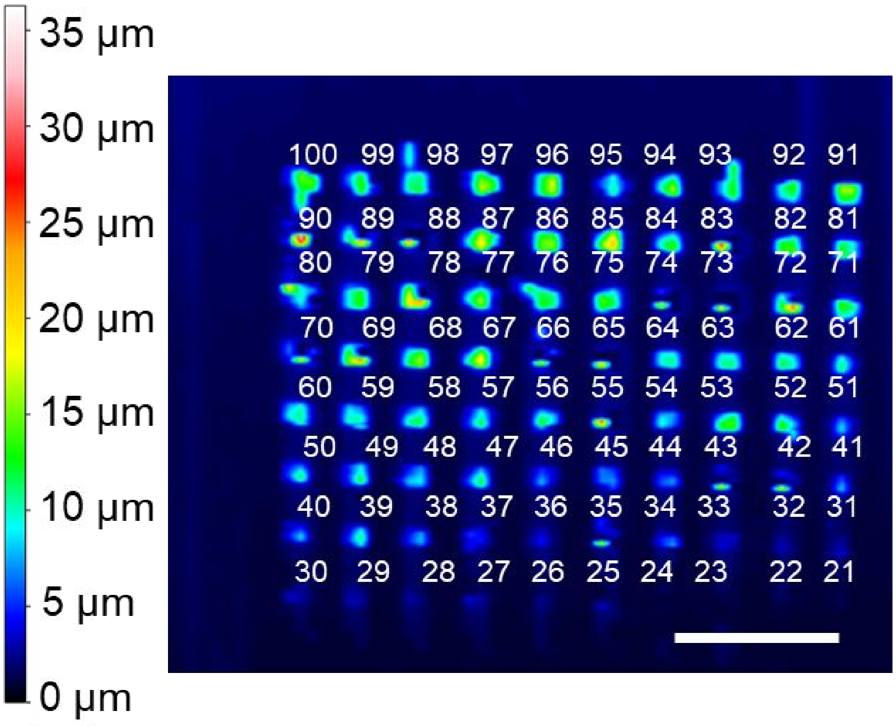

Figure S76. Profilometry image of patterns printed under conditions described in **Table S1C**, *hv* = 3.80 mW/mm[2]. The scale bar is 200 μm.

Table S4. Heights of features printed under conditions in **Table S1C**. All heights and standard deviations are reported as averages of four repeats per point for all features at a 4.5-second intervals of exposure time. No polymer growth was observed in feature(s) preceding the one reported in this table.

Feature number	Time (min)	Height (µm)	Standard deviation	Feature number	Time (min)	Height (µm)	Standard deviation
21	3.3	0.5	0.3	61	9.7	11.6	3.7
22	3.5	N/A	N/A	62	9.8	11.2	3.9
23	3.6	1.0	N/A	63	10.0	8.6	0.4
24	3.8	0.7	0.6	64	10.1	9.8	3.3
25	4.0	1.4	0.8	65	10.3	11.8	6.7
26	4.1	1.0	0.4	66	10.5	11.2	4.1
27	4.3	2.2	0.5	67	10.6	13.2	4.7
28	4.4	2.4	1.1	68	10.8	10.0	3.1
29	4.6	2.2	0.8	69	10.9	12.4	7.7
30	4.8	2.2	0.6	70	11.1	14.5	7.0
31	4.9	2.2	0.5	71	11.2	11.2	3.3
32	5.1	2.1	0.7	72	11.4	21.0	10.3
33	5.2	2.4	0.5	73	11.6	12.9	4.0
34	5.4	3.6	2.1	74	11.7	13.4	6.3
35	5.5	7.1	6.2	75	11.9	11.6	2.1
36	5.7	3.8	1.5	76	12.0	10.5	2.7
37	5.9	4.2	0.6	77	12.2	13.6	1.8
38	6.0	5.0	0.9	78	12.4	14.4	5.9
39	6.2	5.2	2.8	79	12.5	10.9	1.8
40	6.3	5.0	1.4	80	12.7	16.4	5.0
41	6.5	4.3	1.2	81	12.8	14.2	3.2
42	6.7	9.4	6.2	82	13.0	15.3	4.4
43	6.8	8.4	5.5	83	13.1	16.6	7.1
44	7.0	5.5	0.5	84	13.3	12.7	1.1
45	7.1	5.4	0.6	85	13.5	13.5	4.5
46	7.3	5.8	0.7	86	13.6	11.1	1.9
47	7.4	8.5	2.5	87	13.8	13.4	4.0
48	7.6	6.4	0.7	88	13.9	17.2	6.4
49	7.8	7.7	2.5	89	14.1	14.4	5.6
50	7.9	7.5	1.9	90	14.3	22.5	8.9
51	8.1	6.3	0.9	91	14.4	15.4	2.2
52	8.2	9.6	2.8	92	14.6	14.4	3.8
53	8.4	8.6	2.8	93	14.7	12.3	1.1
54	8.6	11.9	8.6	94	14.9	13.7	3.3
55	8.7	12.3	9.1	95	15.0	11.7	3.7
56	8.9	9.9	2.1	96	15.2	14.3	1.2
57	9.0	9.3	1.2	97	15.4	15.0	1.7
58	9.2	8.5	1.1	98	15.5	11.4	2.8
59	9.3	7.9	1.2	99	15.7	12.6	3.3
60	9.5	8.7	1.1	100	15.8	12.1	3.5

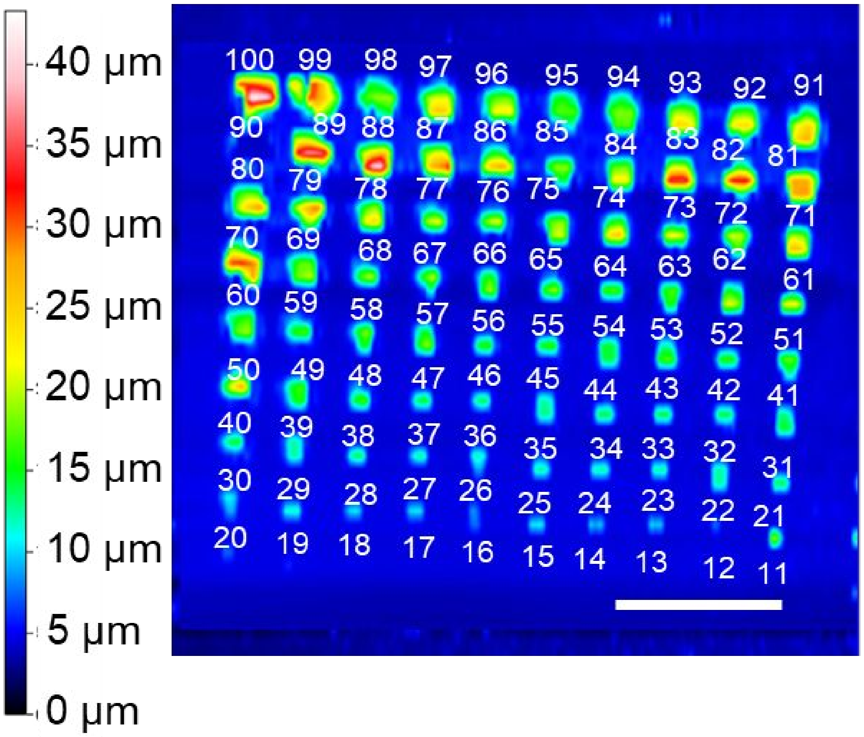

Figure S77. Profilometry image of patterns printed under conditions described in **Table S1D**, *hv* = 6.33 mW/mm². The scale bar is 200 µm.

Table S5. Heights of features printed under conditions in **Table S1D**. All heights and standard deviations are reported as averages of four repeats per point for all features at a 4.5-second intervals of exposure time. No polymer growth was observed in feature(s) preceding the one reported in this table.

Feature number	Time (min)	Height (μm)	Standard deviation	Feature number	Time (min)	Height (μm)	Standard deviation
11	1.7	6.7	6.2	56	8.9	13.4	5.1
12	1.9	N/A	N/A	57	9.0	15.0	2.8
13	2.1	2.6	N/A	58	9.2	12.5	3.6
14	2.2	N/A	N/A	59	9.3	13.6	3.4
15	2.4	1.1	N/A	60	9.5	11.8	6.7
16	2.5	0.7	N/A	61	9.7	18.5	7.5
17	2.7	5.9	N/A	62	9.8	22.5	11.2
18	2.9	1.8	0.5	63	10.0	20.5	9.7
19	3.0	2.2	0.8	64	10.1	16.1	5.6
20	3.2	3.0	1.6	65	10.3	15.2	5.7
21	3.3	6.3	3.2	66	10.5	15.2	5.4
22	3.5	9.3	4.3	67	10.6	14.6	4.5
23	3.6	6.0	2.3	68	10.8	15.4	4.9
24	3.8	6.7	1.7	69	10.9	15.2	3.7
25	4.0	6.8	1.8	70	11.1	14.8	3.6
26	4.1	6.6	1.9	71	11.2	21.7	6.0
27	4.3	6.4	2.6	72	11.4	25.7	9.9
28	4.4	6.7	1.8	73	11.6	18.0	5.6
29	4.6	7.1	2.1	74	11.7	20.7	9.0
30	4.8	6.8	3.2	75	11.9	21.2	0.8
31	4.9	9.5	3.4	76	12.0	15.4	10.2
32	5.1	14.6	8.3	77	12.2	16.0	3.2
33	5.2	9.1	1.9	78	12.4	20.7	9.9
34	5.4	7.3	4.2	79	12.5	14.7	7.1
35	5.5	8.3	1.3	80	12.7	21.8	6.1
36	5.7	9.3	2.7	81	12.8	25.1	9.4
37	5.9	8.8	2.4	82	13.0	22.6	4.8
38	6.0	8.9	2.3	83	13.1	22.3	6.9
39	6.2	8.7	2.1	84	13.3	24.3	6.9
40	6.3	8.9	1.8	85	13.5	25.6	10.1
41	6.5	14.7	6.3	86	13.6	21.1	11.5
42	6.7	13.1	4.5	87	13.8	22.6	6.7
43	6.8	11.5	3.1	88	13.9	22.6	7.1
44	7.0	11.5	2.9	89	14.1	31.6	9.2
45	7.1	10.7	2.6	90	14.3	24.0	10.8
46	7.3	10.7	3.7	91	14.4	16.9	17.3
47	7.4	10.9	3.4	92	14.6	15.4	9.5
48	7.6	10.8	2.6	93	14.7	17.7	9.0
49	7.8	11.8	2.0	94	14.9	11.2	10.5
50	7.9	12.9	1.8	95	15.0	19.0	5.5
51	8.1	19.9	7.5	96	15.2	20.5	3.4
52	8.2	19.0	10.1	97	15.4	21.1	2.9
53	8.4	15.8	6.2	98	15.5	18.0	6.4
54	8.6	13.0	4.0	99	15.7	19.8	4.8
55	8.7	11.7	5.6	100	15.8	23.2	11.0

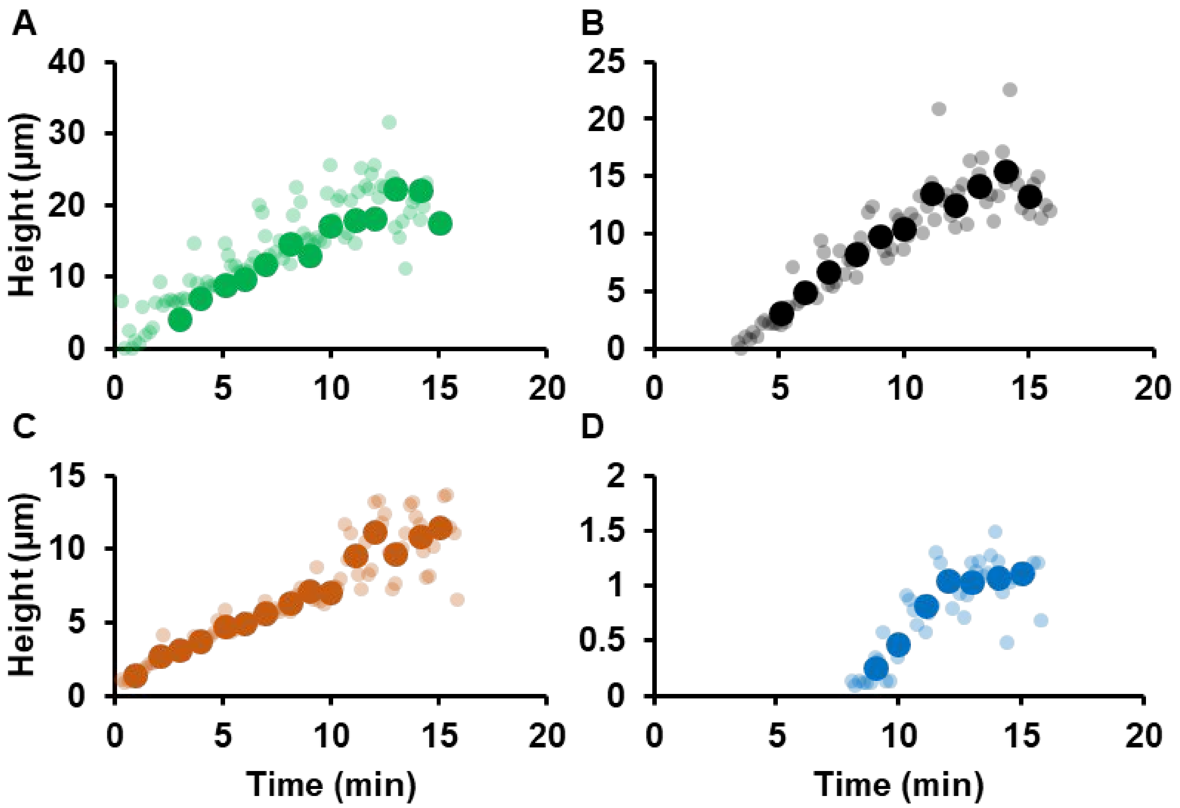

Figure S78. Analysis of the profilometry data shown in **Figures S74 – S77**. Heights are reported as the average of all features within ±0.5-minute intervals of exposure time (dark dots). Varying light intensity (*hv*) on height of six different prints. *hv* = (A) 6.33 mW•mm^{-2}, (B) 3.80 mW•mm^{-2}, (C) 2.53 mW•mm^{-2}, (D) 1.27 mW•mm^{-2} and all error bars are reported as one standard deviation from the mean. Transparent dots show all individual heights for the given graph.

Table S6. Reaction conditions for studying how varying [**SCR043**] affects the growth rate of **SCR043-**functionalized polymer brushes.

prints	TPO (mM)	PETT (mM)	EGDMA (mM)	Intensity (mW/mm^2)	[**SCR043**] (µM)
A	1	100	1300	2.53	500
B	1	100	1300	2.53	250
C	1	100	1300	2.53	100
D	1	100	1300	2.53	50.0
E	1	100	1300	2.53	12.5
F	1	100	1300	2.53	0

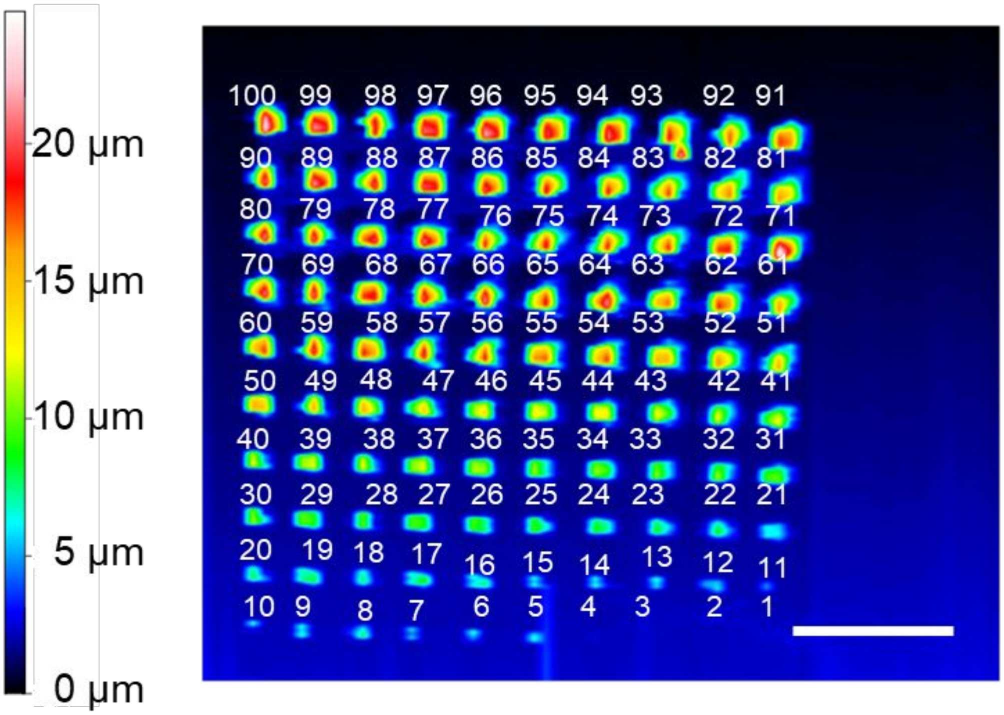

Figure S79. Profilometry image of patterns printed under conditions described in **Table S6A**, [**SCR043**] = 500 µM. The scale bar is 200 µm.

Table S7. Heights of features printed under conditions in **Table S6A**. All heights and standard deviations are reported as averages of four repeats per point for all features at a 4.5-second intervals of exposure time.

Feature number	Time (min)	Height (µm)	Standard deviation	Feature number	Time (min)	Height (µm)	Standard deviation
1	0.2	0.4	N/A	51	8.1	15.4	1.9
2	0.3	2.9	N/A	52	8.2	16.2	1.5
3	0.5	4.4	N/A	53	8.4	16.0	2.3
4	0.6	2.9	3.6	54	8.6	17.5	1.9
5	0.8	4.1	0.7	55	8.7	13.7	6.2
6	1.0	4.6	0.4	56	8.9	17.1	2.3
7	1.1	4.6	0.9	57	9.0	14.5	8.5
8	1.3	5.9	0.1	58	9.2	17.1	1.8
9	1.4	5.0	0.2	59	9.3	18.6	0.4
10	1.6	4.4	N/A	60	9.5	19.5	4.3
11	1.7	3.0	2.9	61	9.7	18.7	2.6
12	1.9	4.0	2.2	62	9.8	18.7	1.9
13	2.1	4.3	1.3	63	10.0	18.6	2.7
14	2.2	4.5	0.4	64	10.1	20.1	3.1
15	2.4	5.7	1.9	65	10.3	18.9	2.4
16	2.5	6.3	0.5	66	10.5	20.2	3.1
17	2.7	7.5	0.4	67	10.6	19.2	1.7
18	2.9	6.5	0.8	68	10.8	19.3	3.6
19	3.0	7.2	0.7	69	10.9	18.8	1.9
20	3.2	6.7	0.2	70	11.1	23.8	4.6
21	3.3	6.7	1.2	71	11.2	21.5	3.4
22	3.5	7.6	0.9	72	11.4	20.6	4.4
23	3.6	8.0	1.0	73	11.6	20.4	4.0
24	3.8	8.4	0.8	74	11.7	20.4	4.2
25	4.0	8.6	0.9	75	11.9	20.7	4.5
26	4.1	9.2	1.0	76	12.0	21.2	4.7
27	4.3	9.1	1.1	77	12.2	18.8	2.2
28	4.4	9.4	0.3	78	12.4	20.9	4.5
29	4.6	9.6	0.7	79	12.5	18.9	2.7
30	4.8	10.0	0.9	80	12.7	18.7	12.8
31	4.9	9.8	0.8	81	12.8	19.7	4.7
32	5.1	10.2	0.3	82	13.0	23.8	9.2
33	5.2	10.2	0.7	83	13.1	21.1	4.5
34	5.4	10.7	1.1	84	13.3	20.7	4.4
35	5.5	10.8	0.7	85	13.5	21.3	5.2
36	5.7	11.1	1.4	86	13.6	21.5	5.1
37	5.9	10.9	1.4	87	13.8	19.7	2.8
38	6.0	11.5	0.4	88	13.9	20.6	5.3
39	6.2	11.8	0.8	89	14.1	20.3	2.6
40	6.3	12.2	1.4	90	14.3	22.9	5.2
41	6.5	11.9	0.8	91	14.4	22.7	5.1
42	6.7	11.9	0.7	92	14.6	29.9	9.8
43	6.8	12.2	1.2	93	14.7	24.3	4.4
44	7.0	12.8	1.3	94	14.9	25.4	7.6
45	7.1	12.8	0.8	95	15.0	22.8	5.2
46	7.3	13.4	1.6	96	15.2	25.6	9.1
47	7.4	13.5	0.5	97	15.4	23.8	6.0
48	7.6	14.4	0.8	98	15.5	23.3	5.1
49	7.8	15.1	0.9	99	15.7	26.3	6.5
50	7.9	15.9	2.1	100	15.8	23.7	N/A

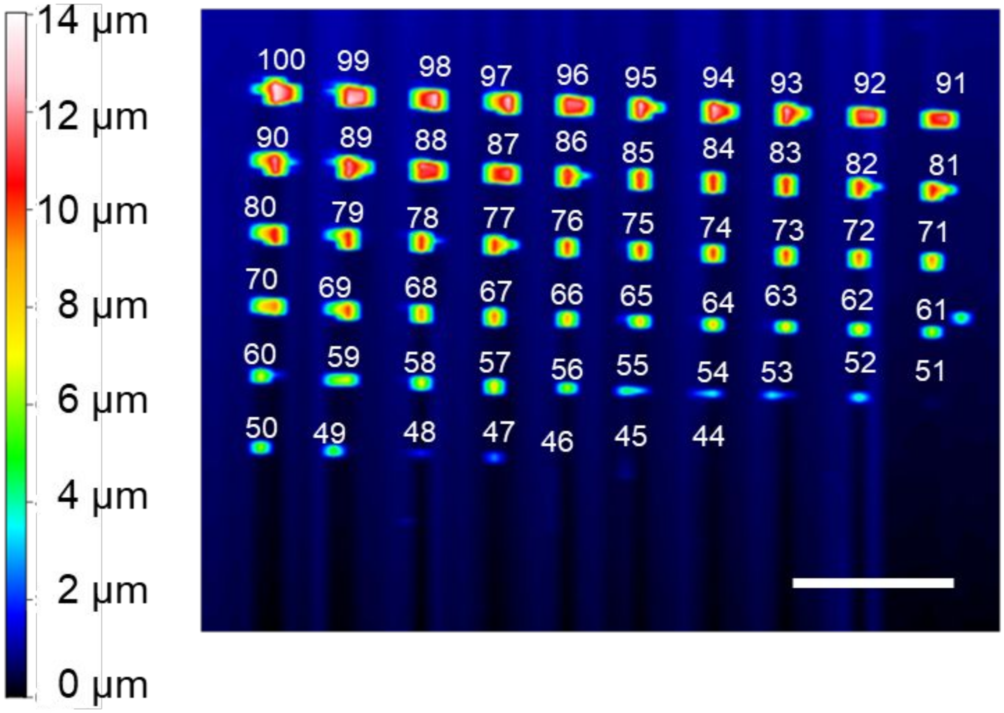

Figure S80. Profilometry image of patterns printed under conditions described in **Table S6B**, [**SCR043**] = 250 µM. The scale bar is 200 µm.

Table S8. Heights of features printed under conditions in **Table S6B**. All heights and standard deviations are reported as averages of four repeats per point for all features at a 4.5-second intervals of exposure time. No polymer growth was observed in feature(s) preceding the one reported in this table.

Feature number	Time (min)	Height (μm)	Standard deviation	Feature number	Time (min)	Height (μm)	Standard deviation
44	7.0	0.5	N/A	72	11.4	8.8	1.2
45	7.1	N/A	N/A	73	11.6	8.9	1.2
46	7.3	2.4	N/A	74	11.7	9.3	1.0
47	7.4	0.7	0.8	75	11.9	9.3	0.9
48	7.6	1.9	2.2	76	12.0	9.7	0.3
49	7.8	3.0	2.8	77	12.2	9.9	0.4
50	7.9	3.4	3.2	78	12.4	10.0	0.7
51	8.1	2.6	2.1	79	12.5	11.8	3.6
52	8.2	3.4	1.3	80	12.7	9.4	1.0
53	8.4	3.8	0.5	81	12.8	9.6	1.3
54	8.6	3.7	1.2	82	13.0	10.5	1.0
55	8.7	5.7	0.6	83	13.1	10.2	1.2
56	8.9	5.2	1.8	84	13.3	11.0	0.7
57	9.0	6.4	0.9	85	13.5	10.3	0.7
58	9.2	6.3	0.9	86	13.6	10.5	0.9
59	9.3	6.2	3.8	87	13.8	10.7	0.9
60	9.5	4.7	3.0	88	13.9	10.7	1.0
61	9.7	6.5	1.1	89	14.1	11.8	1.9
62	9.8	6.4	1.5	90	14.3	18.0	14.3
63	10.0	6.7	1.5	91	14.4	10.4	2.3
64	10.1	6.6	1.5	92	14.6	12.3	2.5
65	10.3	7.5	1.0	93	14.7	11.2	1.5
66	10.5	8.4	0.9	94	14.9	11.4	1.0
67	10.6	10.0	2.3	95	15.0	11.1	1.1
68	10.8	9.0	0.6	96	15.2	11.4	1.2
69	10.9	9.0	0.4	97	15.4	11.2	1.2
70	11.1	8.6	0.7	98	15.5	11.7	1.3
71	11.2	8.4	1.2	99	15.7	12.2	1.2
				100	15.8	7.4	6.0

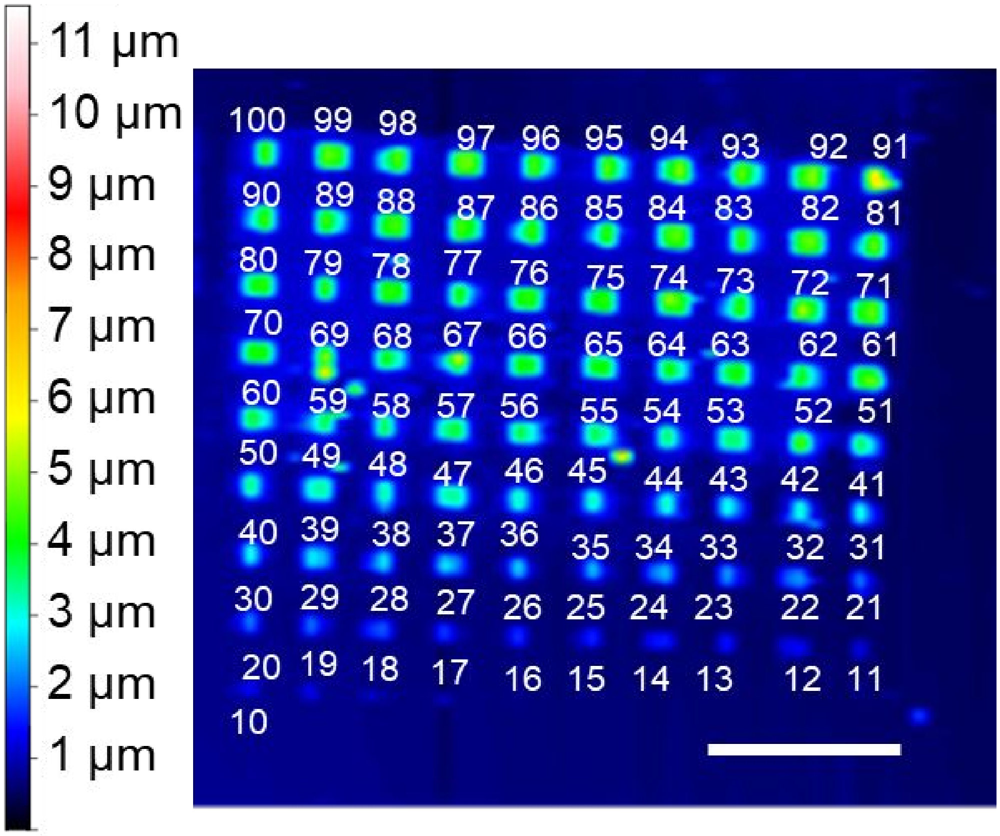

Figure S81. Profilometry image of patterns printed under conditions described in **Table S6C**, [**SCR043**] = 100 µM. The scale bar is 200 µm.

Table S9. Heights of features printed under conditions in **Table S6C**. All heights and standard deviations are reported as averages of four repeats per point for all features at a 4.5-second intervals of exposure time. No polymer growth was observed in feature(s) preceding the one reported in this table.

Feature number	Time (min)	Height (µm)	Standard deviation	Feature number	Time (min)	Height (µm)	Standard deviation
10	1.6	0.1	N/A	55	8.7	2.6	0.2
11	1.7	0.2	N/A	56	8.9	2.6	0.2
12	1.9	N/A	N/A	57	9.0	2.6	0.2
13	2.1	N/A	N/A	58	9.2	2.7	0.2
14	2.2	0.5	0.2	59	9.3	3.1	0.9
15	2.4	0.0	N/A	60	9.5	2.8	0.1
16	2.5	0.3	0.0	61	9.7	3.7	1.2
17	2.7	0.6	0.2	62	9.8	3.0	0.3
18	2.9	0.8	0.7	63	10.0	2.8	0.2
19	3.0	0.7	0.1	64	10.1	2.8	0.3
20	3.2	0.7	0.2	65	10.3	2.9	0.1
21	3.3	0.7	0.0	66	10.5	2.8	0.2
22	3.5	1.2	0.5	67	10.6	3.5	1.4
23	3.6	0.9	0.1	68	10.8	3.0	0.4
24	3.8	0.9	0.2	69	10.9	3.9	1.9
25	4.0	1.4	0.9	70	11.1	3.2	0.5
26	4.1	1.1	0.2	71	11.2	2.9	0.8
27	4.3	1.2	0.1	72	11.4	3.5	0.7
28	4.4	1.2	0.1	73	11.6	3.2	0.3
29	4.6	1.4	0.0	74	11.7	3.3	0.6
30	4.8	1.5	0.0	75	11.9	2.9	0.5
31	4.9	1.5	0.1	76	12.0	3.1	0.2
32	5.1	1.6	0.2	77	12.2	3.7	1.6
33	5.2	1.6	0.3	78	12.4	3.4	0.7
34	5.4	2.1	0.6	79	12.5	2.9	0.4
35	5.5	1.8	0.2	80	12.7	3.5	0.6
36	5.7	1.9	0.2	81	12.8	2.6	1.7
37	5.9	1.9	0.5	82	13.0	3.3	0.2
38	6.0	2.0	0.2	83	13.1	3.7	0.7
39	6.2	1.9	0.1	84	13.3	3.9	1.3
40	6.3	2.1	0.2	85	13.5	3.8	1.1
41	6.5	2.3	0.3	86	13.6	3.5	0.6
42	6.7	2.6	0.5	87	13.8	3.2	0.2
43	6.8	2.4	0.2	88	13.9	3.5	0.4
44	7.0	2.2	0.2	89	14.1	3.4	0.4
45	7.1	2.5	0.4	90	14.3	3.4	0.3
46	7.3	2.4	0.3	91	14.4	4.1	1.5
47	7.4	2.3	0.3	92	14.6	4.4	1.5
48	7.6	2.5	0.3	93	14.7	4.1	0.9
49	7.8	2.3	0.1	94	14.9	5.3	2.8
50	7.9	2.5	0.2	95	15.0	5.5	3.3
51	8.1	3.0	0.8	96	15.2	5.9	4.3
52	8.2	2.9	0.7	97	15.4	4.8	2.4
53	8.4	2.5	0.1	98	15.5	5.8	3.8
54	8.6	2.6	0.2	99	15.7	4.9	2.0

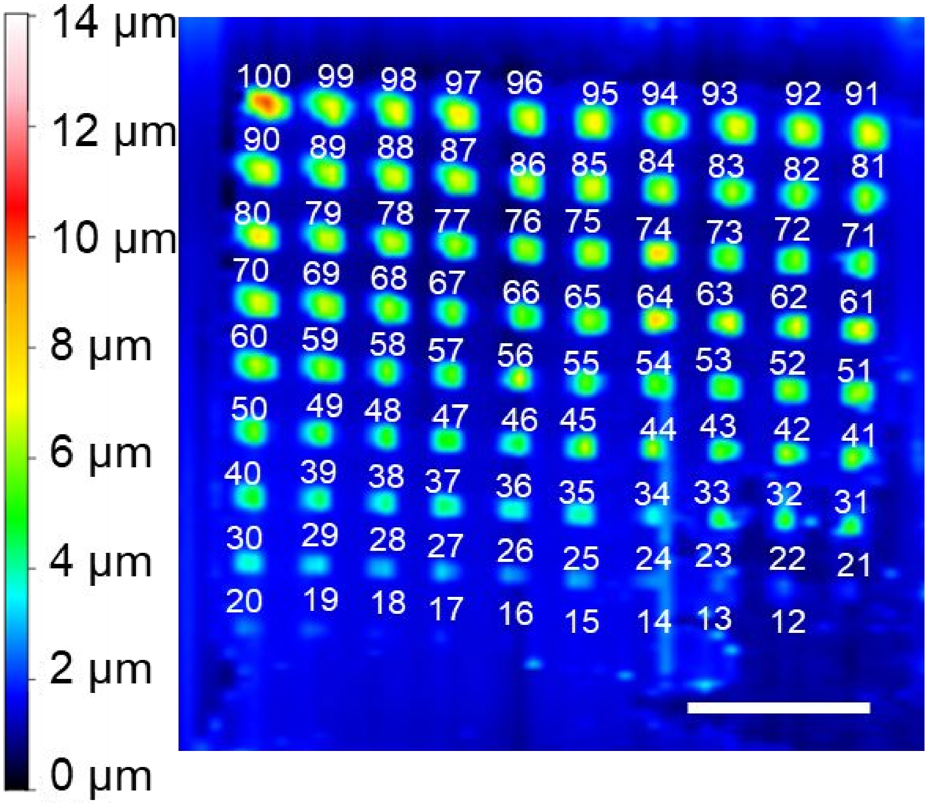

Figure S82. Profilometry image of patterns printed under conditions described in **Table S6D**, [**SCR043**] = 50.0 μM. The scale bar is 200 μm.

Table S10. Heights of features printed under conditions in **Table S6D**. All heights and standard deviations are reported as averages of four repeats per point for all features at a 4.5-second intervals of exposure time. No polymer growth was observed in feature(s) preceding the one reported in this table.

Feature number	Time (min)	Height (µm)	Standard deviation	Feature number	Time (min)	Height (µm)	Standard deviation
12	1.9	0.5	N/A	56	8.9	6.5	1.1
13	2.1	1.0	N/A	57	9.0	6.1	1.0
14	2.2	1.3	N/A	58	9.2	5.7	0.8
15	2.4	1.1	N/A	59	9.3	6.2	1.3
16	2.5	1.4	N/A	60	9.5	7.2	0.9
17	2.7	1.4	0.6	61	9.7	6.8	0.7
18	2.9	1.2	0.4	62	9.8	6.5	1.2
19	3.0	1.3	0.4	63	10.0	6.7	1.1
20	3.2	1.3	0.3	64	10.1	7.3	1.4
21	3.3	1.3	0.2	65	10.3	6.4	1.5
22	3.5	2.7	2.3	66	10.5	6.8	1.4
23	3.6	1.5	0.5	67	10.6	6.7	1.2
24	3.8	1.6	0.4	68	10.8	6.3	0.8
25	4.0	1.9	0.7	69	10.9	5.6	0.3
26	4.1	2.1	0.6	70	11.1	6.4	0.3
27	4.3	2.5	0.7	71	11.2	7.4	1.5
28	4.4	2.5	0.8	72	11.4	5.6	0.4
29	4.6	2.6	0.6	73	11.6	6.7	1.3
30	4.8	3.5	2.2	74	11.7	6.9	0.9
31	4.9	4.7	1.0	75	11.9	7.3	2.2
32	5.1	4.1	1.3	76	12.0	7.1	1.4
33	5.2	4.1	1.1	77	12.2	6.5	1.2
34	5.4	3.7	1.0	78	12.4	6.2	0.5
35	5.5	4.0	1.2	79	12.5	6.3	0.8
36	5.7	3.6	0.7	80	12.7	6.2	0.8
37	5.9	4.3	1.2	81	12.8	6.6	1.0
38	6.0	4.2	1.1	82	13.0	6.0	0.2
39	6.2	4.0	0.7	83	13.1	6.5	0.7
40	6.3	4.8	1.2	84	13.3	6.6	0.8
41	6.5	6.4	1.2	85	13.5	8.0	4.0
42	6.7	5.0	0.9	86	13.6	6.9	1.8
43	6.8	5.1	0.8	87	13.8	6.4	0.8
44	7.0	6.3	1.8	88	13.9	6.3	0.5
45	7.1	5.3	0.8	89	14.1	6.1	0.3
46	7.3	5.1	0.9	90	14.3	6.5	0.7
47	7.4	5.4	1.0	91	14.4	8.2	2.2
48	7.6	5.9	1.4	92	14.6	7.2	1.2
49	7.8	5.8	1.1	93	14.7	6.5	0.6
50	7.9	5.5	1.3	94	14.9	6.4	0.7
51	8.1	6.6	1.9	95	15.0	7.9	3.3
52	8.2	5.6	0.3	96	15.2	7.1	1.9
53	8.4	5.6	1.2	97	15.4	6.6	0.9
54	8.6	6.0	1.2	98	15.5	7.2	1.1
55	8.7	6.3	1.4	99	15.7	6.8	0.3

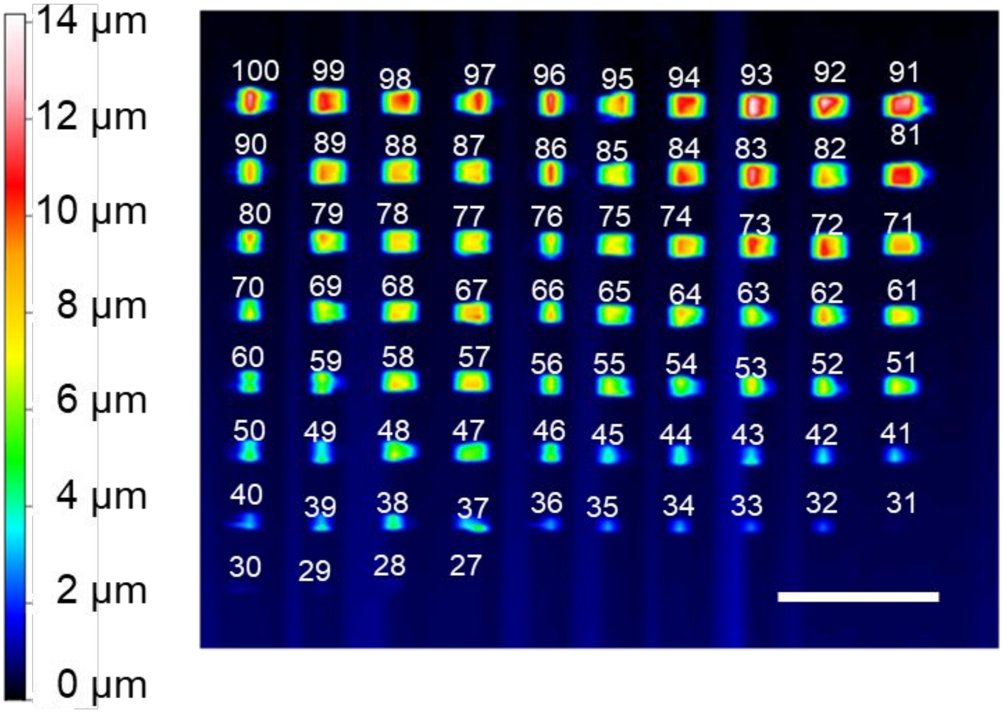

Figure S83. Profilometry image of patterns printed under conditions described in **Table S6E**, [**SCR043**] = 12.5 μM. The scale bar is 200 μm.

Table S11. Heights of features printed under conditions in **Table S6E**. All heights and standard deviations are reported as averages of four repeats per point for all features at a 4.5-second intervals of exposure time. No polymer growth was observed in feature(s) preceding the one reported in this table.

Feature number	Time (min)	Height (µm)	Standard deviation	Feature number	Time (min)	Height (µm)	Standard deviation
27	4.3	0.4	0.4	64	10.1	6.0	2.1
28	4.4	0.7	0.4	65	10.3	5.5	1.8
29	4.6	1.0	0.6	66	10.5	6.9	2.5
30	4.8	1.0	0.6	67	10.6	7.2	2.6
31	4.9	0.6	0.5	68	10.8	7.0	2.4
32	5.1	1.7	0.4	69	10.9	7.8	2.3
33	5.2	1.9	0.5	70	11.1	6.3	1.8
34	5.4	2.1	0.6	71	11.2	7.5	1.1
35	5.5	2.0	0.6	72	11.4	9.0	2.0
36	5.7	2.3	0.3	73	11.6	7.3	3.1
37	5.9	3.0	1.5	74	11.7	7.4	2.4
38	6.0	2.9	1.3	75	11.9	8.0	1.3
39	6.2	3.0	1.1	76	12.0	9.3	4.3
40	6.3	2.8	0.8	77	12.2	7.5	2.3
41	6.5	2.7	0.4	78	12.4	6.8	2.1
42	6.7	3.2	0.4	79	12.5	7.5	2.5
43	6.8	3.7	0.9	80	12.7	7.2	2.9
44	7.0	3.9	1.5	81	12.8	7.8	2.4
45	7.1	4.1	1.0	82	13.0	7.8	2.1
46	7.3	4.4	1.8	83	13.1	10.2	4.9
47	7.4	4.0	1.5	84	13.3	8.6	2.9
48	7.6	4.6	2.1	85	13.5	7.8	2.6
49	7.8	4.5	1.8	86	13.6	11.0	6.6
50	7.9	6.5	2.0	87	13.8	8.6	3.2
51	8.1	5.3	1.3	88	13.9	9.0	4.5
52	8.2	5.8	1.4	89	14.1	8.3	2.8
53	8.4	6.2	1.3	90	14.3	7.8	1.7
54	8.6	6.2	0.7	91	14.4	9.0	2.8
55	8.7	5.0	1.9	92	14.6	9.5	2.8
56	8.9	5.1	1.9	93	14.7	10.0	2.7
57	9.0	5.2	2.1	94	14.9	7.6	2.7
58	9.2	6.3	2.3	95	15.0	8.1	2.4
59	9.3	6.2	1.8	96	15.2	11.9	2.5
60	9.5	5.8	2.1	97	15.4	8.6	2.8
61	9.7	5.9	1.4	98	15.5	7.8	2.8
62	9.8	7.8	1.5	99	15.7	7.6	3.1
63	10.0	6.4	0.9	100	15.8	8.9	4.4

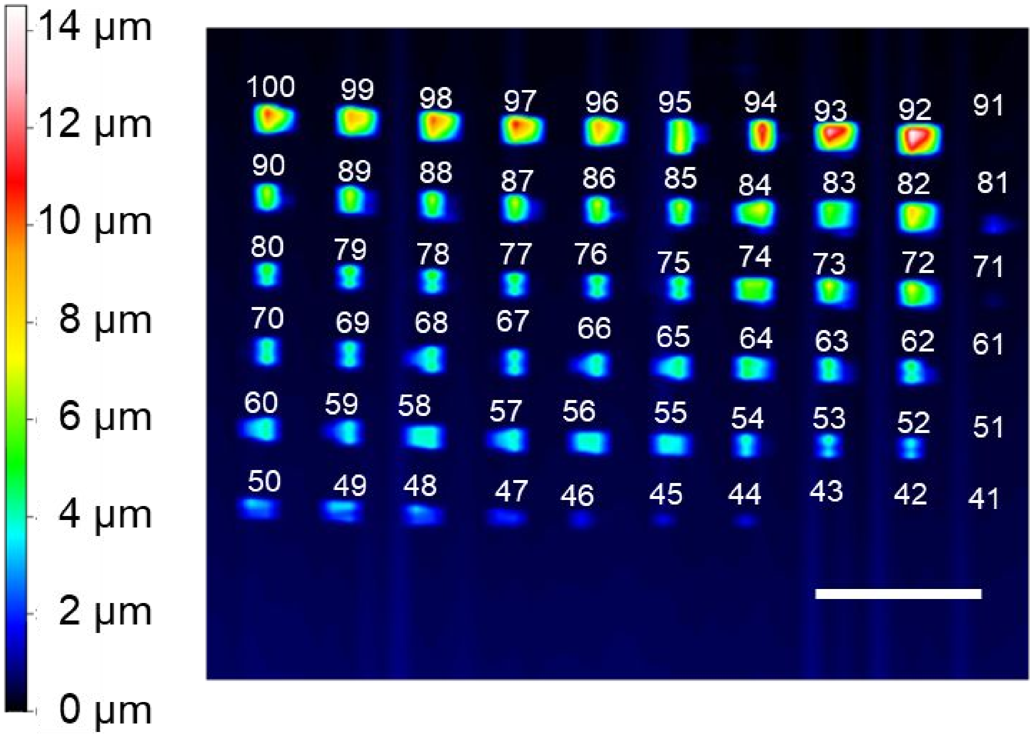

Figure S84. Profilometry image of patterns printed under conditions described in **Table S6F**, [**SCR043**] = 0 µM. The scale bar is 200 µm.

Table S12. Heights of features printed under conditions in **Table S6F**. All heights and standard deviations are reported as averages of four repeats per point for all features at a 4.5-second intervals of exposure time. No polymer growth was observed in feature(s) preceding the one reported in this table.

Feature number	Time (min)	Height (µm)	Standard deviation	Feature number	Time (min)	Height (µm)	Standard deviation
41	6.5	0.2	0.2	71	11.2	3.5	1.2
42	6.7	1.0	N/A	72	11.4	4.4	2.1
43	6.8	0.7	0.4	73	11.6	4.3	2.0
44	7.0	1.0	0.2	74	11.7	3.9	1.5
45	7.1	1.0	0.1	75	11.9	3.7	1.2
46	7.3	1.1	0.2	76	12.0	3.7	1.2
47	7.4	1.2	0.3	77	12.2	4.7	1.4
48	7.6	1.7	0.8	78	12.4	4.4	1.9
49	7.8	1.8	0.8	79	12.5	6.8	4.8
50	7.9	1.4	1.2	80	12.7	5.3	0.8
51	8.1	1.7	1.2	81	12.8	4.4	1.7
52	8.2	3.5	1.5	82	13.0	5.5	2.2
53	8.4	3.0	1.1	83	13.1	4.8	1.3
54	8.6	2.8	1.2	84	13.3	5.1	1.8
55	8.7	2.5	1.0	85	13.5	5.0	1.6
56	8.9	2.6	1.0	86	13.6	5.4	1.6
57	9.0	2.7	1.0	87	13.8	6.1	2.8
58	9.2	2.5	0.8	88	13.9	5.8	2.2
59	9.3	2.6	0.7	89	14.1	5.6	1.5
60	9.5	2.4	0.9	90	14.3	6.2	2.2
61	9.7	2.2	0.8	91	14.4	7.5	5.8
62	9.8	4.7	2.9	92	14.6	9.8	2.7
63	10.0	3.3	1.2	93	14.7	9.1	2.3
64	10.1	3.1	1.1	94	14.9	8.4	0.7
65	10.3	3.0	1.0	95	15.0	9.3	1.0
66	10.5	3.1	1.1	96	15.2	9.7	1.2
67	10.6	3.3	1.2	97	15.4	14.7	9.0
68	10.8	3.1	1.1	98	15.5	18.6	12.3
69	10.9	4.4	1.0	99	15.7	13.2	10.1
70	11.1	4.2	0.9	100	15.8	4.4	2.8

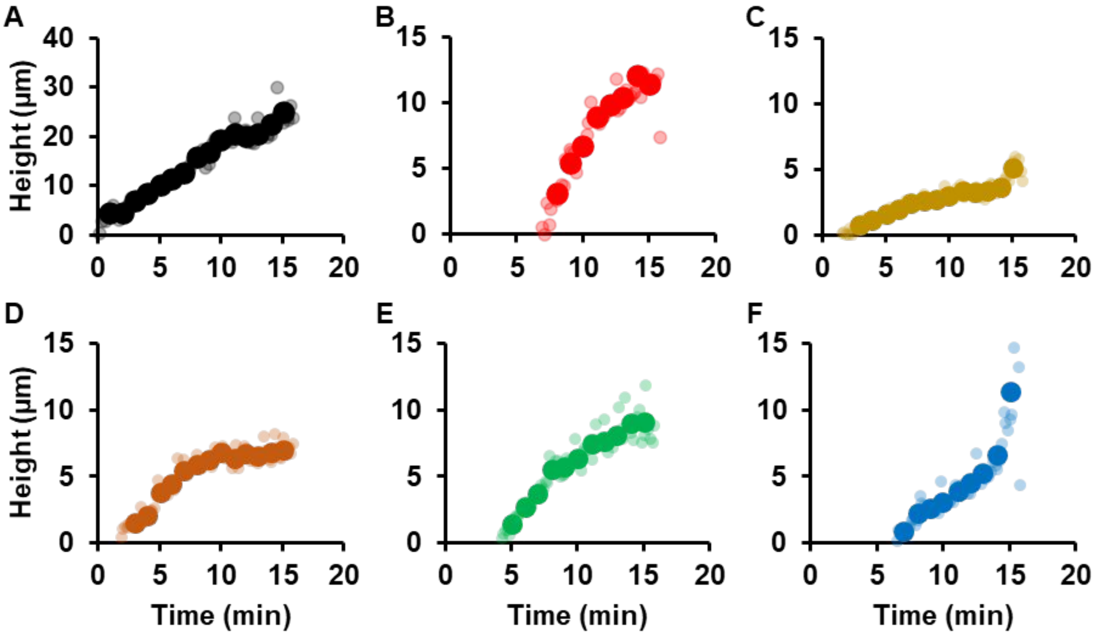

Figure S85. Analysis of the profilometry data shown in **Figures S79 – S84**. Heights are reported as the average of all features within ±0.5-minute intervals of exposure time (dark dots). Varying concentration of **SCR043** on height of six different prints. [**SCR043**] = (A) 500 µM, (B) 250 µM, (C) 100 µM, (D) 50.0 µM, (E) 12.5 µM, (F) 0 µM, and all error bars are reported as one standard deviation from the mean. Transparent dots show all individual heights for the given graph.

6. Chemical characterization of polymer brush surfaces

X-ray photoelectron spectroscopy (XPS)

Two surfaces were patterned to examine the chemical composition of the polymer brushes by XPS. One surface was patterned without **SCR043** ([EGDMA] = 1300 mM, [TPO] = 1.0 mM, [PETT] = 100 mM and *hv* = 2.53 mW mm^{-2}), and one with **SCR043** ([EGDMA] = 1300 mM, [TPO] = 1.0 mM, [PETT] = 100 mM, [**SCR043**] = 500 μM, and *hv* = 2.53 mW mm^{-2}). After patterning the polymers, the surfaces were washed with EtOH (~5 mL), then dried under a stream of air. XPS data of N1s and C1s were taken with a Physical Electronics VersaProbe II XPS using an Al monochromatic X-ray source (1486.6 eV) at 37.6 W and Neutralizer gun operating at 2.0 V and 20 μA. Beam diameter was set to 200 μm, time per data point to 10 s at intervals of 0.125 eV, and pass energy at 29.35 eV and 20 scans of N1s.

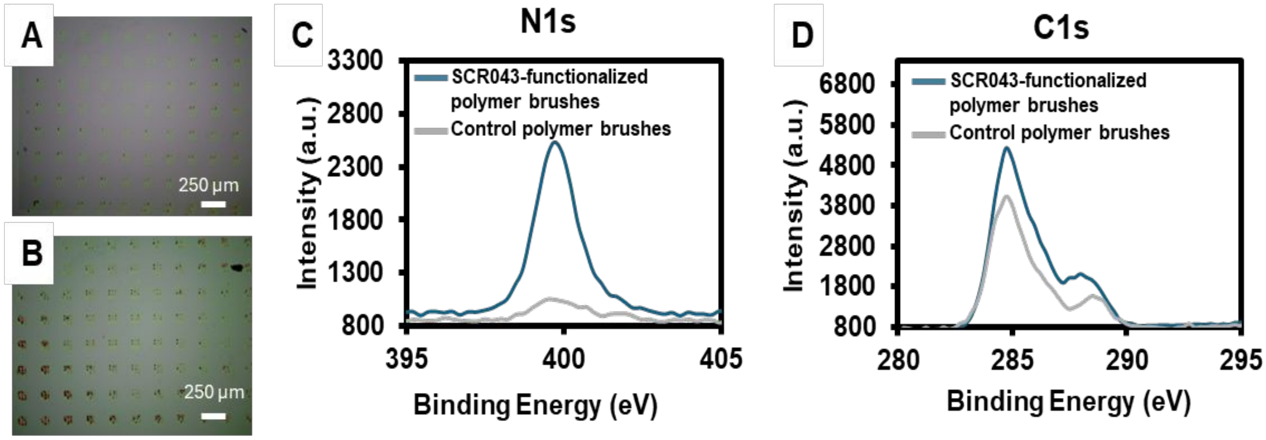

Figure S86. (A) Optical image of surface patterned without **SCR043**-functionalized polymer brushes ("control polymer brushes"). (B) Optical image of surface patterned with **SCR043**-functionalized polymer brushes. (C) XPS spectrum of **SCR043**-functionalized polymer brushes showing an increase in nitrogen signal (blue) compared to non-**SCR043**-functionalized polymer brush signal (grey). (D) XPS spectrum of **SCR043**-functionalized polymer brushes showing an increase in carbon signal (blue) compared to non-**SCR043**-functionalized polymer brush signal (grey).

Time of Flight Secondary Ion Mass Spectrometry (ToF-SIMS)

Two surfaces were patterned to examine the chemical composition of the polymer brushes by ToF-SIMS. One surface was patterned without **SCR043** ([EGDMA] = 1300 mM, [TPO] = 1.0 mM, [PETT] = 100 mM, and *hv* = 2.53 mW mm^{-2}), and one with **SCR043** ([EGDMA] = 1300 mM, [TPO] = 1.0 mM, [PETT] = 100 mM, [**SCR043**] = 500 µM, and *hv* = 2.53 mW mm^{-2}). After patterning the polymers, the surfaces were washed with EtOH, then dried under a stream of air. ToF-SIMS data were taken using a triple focusing time-of-flight (TRIFT) mass spectrometer (Physical Electronics, USA) using a 30 kV LMIG with a Bi^+ emitter. Data were collected from a 600 x 600 µm scan area over 30 cycles. The data were analyzed using the software TOF-DR (Physical Electronics, USA).

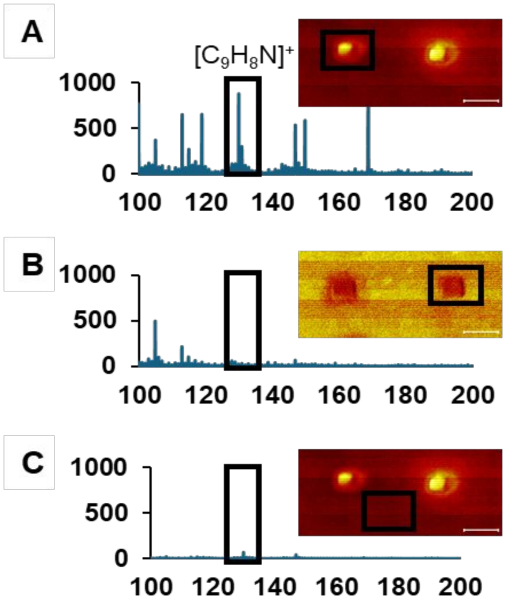

Figure S87. ToF-SiMs of **SCR043**-functionalized polymer brushes compared to non-**SCR043**-functionalized polymer brushes. (A) **SCR043**-functionalized polymer brushes and corresponding total ion mass spectrum. Box on **SCR043** spectrum is 130.02 m/z. The scale bar is 100 µm. (B) Total ion mass spectrum image of non-**SCR043**-functionalized polymer brushes and corresponding mass spectrum. Box on non-**SCR043** spectrum is 130.02 m/z. The scale bar is 100 µm. (c) Total ion mass spectrum of thiol-terminated surface where there is no photopolymerization and corresponding mass spectrum. The black box on spectrum is at 130.02 m/z. The scale bar is 100 µm.

Raman microscopy

Six surfaces were prepared to examine the chemical composition of the polymer brushes by Raman microscopy. One surface was a freshly cut SiO_2 wafer, one surface had a [**SCR043**] = 500 μM solution drop-casted onto a SiO_2 wafer and air dried, one surface had an EGDMA drop-casted onto a SiO_2 wafer and air dried, one surface had PETT drop-casted onto a SiO_2 wafer and air dried, one surface was patterned without **SCR043** ([EGDMA] = 1300 mM, [TPO] = 1.0 mM, [PETT] = 100 mM and *hv* = 2.53 mW mm^{-2}), and one with **SCR043** ([EGDMA] = 1300 mM, [TPO] = 1.0 mM, [PETT] = 100 mM, [**SCR043**] = 500 μM, and *hv* = 2.53 mW mm^{-2}). After patterning the polymers, the surfaces were washed with EtOH (~5 mL), then dried under a stream of air. Raman measurements were taken on the WITec (Oxford Instruments) Confocal Raman Microscope alpha300R. 3000 scans were taken per substrate with a 50X Zeiss EC Epiplan-Neofluar NA 0.8 DIC lens and a laser excitation wavelength of 633 nm.

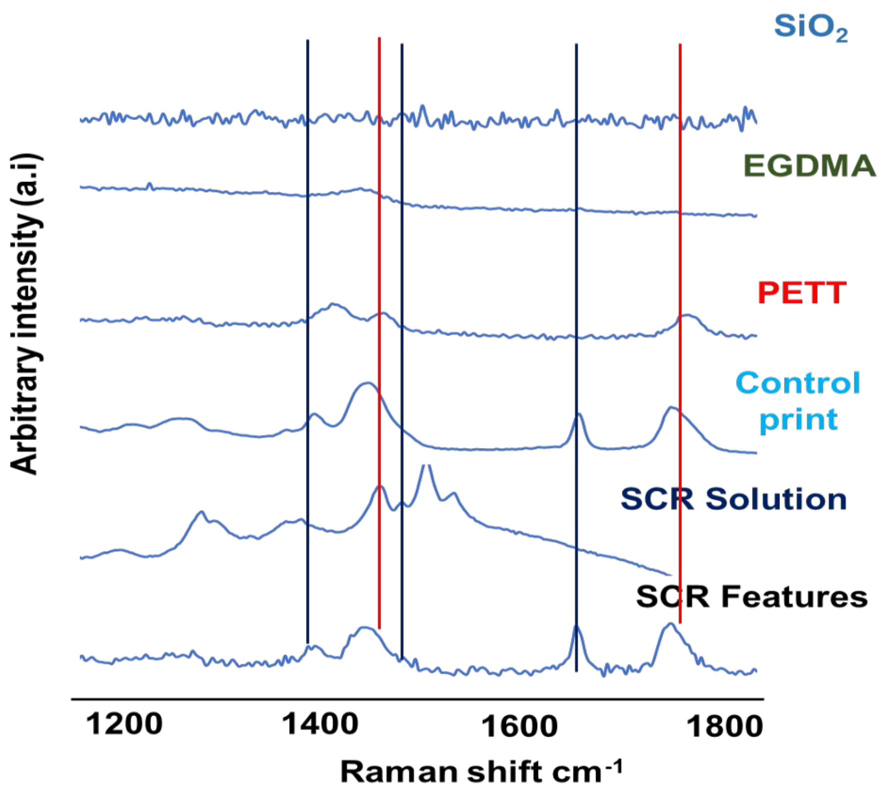

Figure S88. The top graph (“SiO_2”) shows a freshly cut SiO_2 wafer. Second from the top graph (“EGDMA”) had an EGDMA drop-casted onto a SiO_2 wafer showing a peak at 1411 corresponding to a CH_2 bond. Third from top (“PETT”) shows the PETT drop-casted onto a SiO_2 wafer and air-dried, giving peaks at 1411 and 1450 corresponding to a CH_2 bond. Middle spectrum (“Control Print”) comes from a surface patterned without **SCR043** ([EGDMA] = 1300 mM, [TPO] = 1.0 mM, [PETT] = 100 mM and *hv* = 2.53 mW mm^{-2}) showing peaks at 1411 and 1450 corresponding to CH_2 bonds, 1640 for C=C bond, and 1725 corresponding to C=O bond[8]. The second from the bottom graph (“SCR solution”) shows [**SCR043**] = 500 μM solution drop-casted onto a SiO_2 wafer and air-dried showing peaks at 1463-1490, 1550, and 1608 corresponding to a C-C benzene stretching, pyrrole ring stretching, and benzene stretching respectively[9]. The bottom graph (“SCR Features”) shows a printed pattern containing **SCR043** ([EGDMA] = 1300 mM, [TPO] = 1.0 mM, [PETT] = 100 mM, [**SCR043**] = 500 μM, and *hv* = 2.53 mW mm^{-2}) with peaks at 1411 and 1450 corresponding to a CH_2 bond, 1463-1490, and 1550 for a C-C benzene stretching, pyrrole ring stretching, 1640 for a C=C bond and 1725 corresponding to a C=O bond[9].

7. Binding Studies

General methods

SCR043-functionalized polymers were printed into 4 x 4 patterns on thiol-terminated surfaces at varying concentrations of **SCR043** (500 μM, 250 μM, 100 μM, 50.0 μM, 12.5 μM, 0 μM) and varying time, *t* (4 min –12 min) with 30-second intervals. Light intensity was kept constant at 2.53 mW/mm^2 for all prints. After patterning the polymer brushes, each surface was washed with DMSO and EtOH and then dried under a stream of air. The prints were subsequently incubated with varying concentrations of **α-Man-FL** [10^{-3} M, $10^{-3.5}$ M,10^{-4} M, $10^{-4.5}$ M,10^{-5} M, $10^{-5.5}$ M]. Fluorophores were dissolved in a solution of Tris buffer 20 mM, pH = 7.4, 0.01% Tween20 and incubated with the patterned substrates for one hour. The surfaces were washed for 10 min in fresh solutions of MilliQ water with 0.01% Tween20 and lightly dried under a stream of air. The fluorescence images of the surfaces post-incubation are shown in the figures **S89-S202** below.

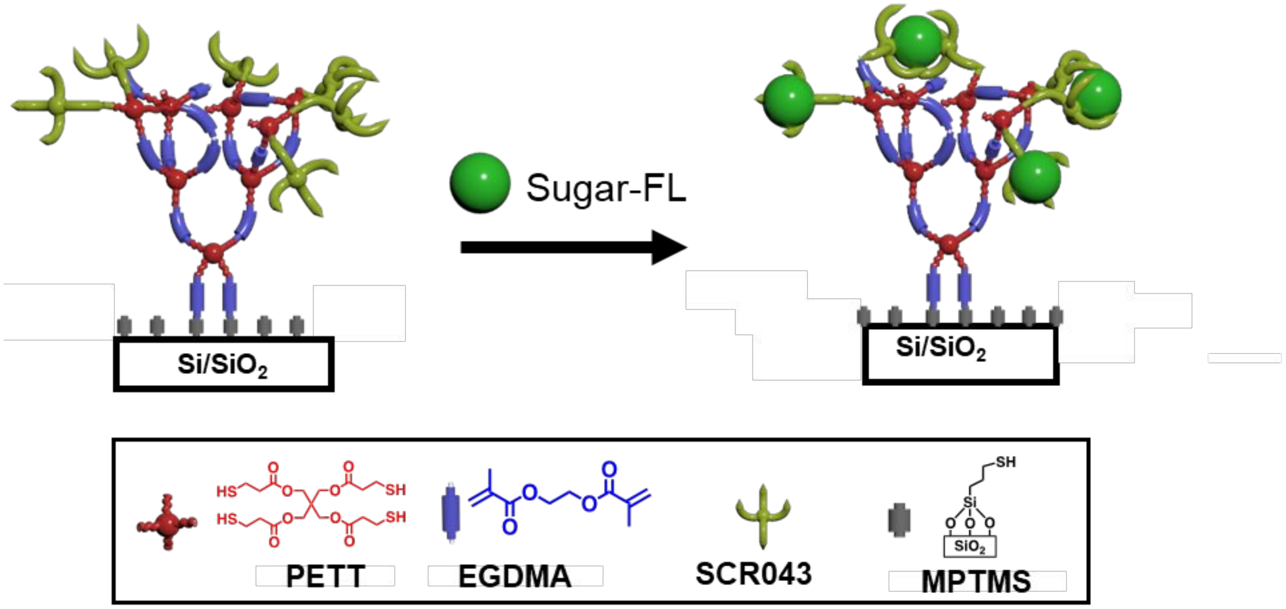

Scheme S9. Binding of **SCR043**-functionalized polymer brushes with fluorescent sugars.

Table S13. Experimental table showing the various binding studies.

SCR043	α-Man	10^{-3} M	$10^{-3.5}$ M	10^{-4} M	$10^{-4.5}$ M	10^{-5} M	$10^{-5.5}$ M	10^{-6} M
500μM		—	—	—	—	—	—	—
250μM		—	—	—	—	—	—	—
100μM		—	—	—	—	—	—	—
50μM		—	—	—	—	—	—	—
12.5μM		—	—	—	—	—	—	—
0μM		—	—	—	—	—	—	—
SCR043	Flu	10^{-3} M	$10^{-3.5}$ M	10^{-4} M	$10^{-4.5}$ M	10^{-5} M	$10^{-5.5}$ M	10^{-6} M
500μM		—	—	—	—	—	—	—
SCR043	α-Gal	10^{-3} M	$10^{-3.5}$ M	10^{-4} M	$10^{-4.5}$ M	10^{-5} M	$10^{-5.5}$ M	10^{-6} M
100μM		—	—	—	—	—	—	—
SCR043	α-Gluc	10^{-3} M	$10^{-3.5}$ M	10^{-4} M	$10^{-4.5}$ M	10^{-5} M	$10^{-5.5}$ M	10^{-6} M
100μM		—	—	—	—	—	—	—
SCR043	β-Gluc	10^{-3} M	$10^{-3.5}$ M	10^{-4} M	$10^{-4.5}$ M	10^{-5} M	$10^{-5.5}$ M	10^{-6} M
100μM		—	—	—	—	—	—	—
SCR043	β-Gal	10^{-3} M	$10^{-3.5}$ M	10^{-4} M	$10^{-4.5}$ M	10^{-5} M	$10^{-5.5}$ M	10^{-6} M
100μM		—	—	—	—	—	—	—

Table S14. Binding conditions for surfaces printed under conditions shown in **Table S13**.

TPO (mM)	PETT (mM)	EGDMA (mM)	Intensity (mW/mm2)	[**SCR043**] (μM)	[α-**Man-FL**] (M)
1	100	1300	2.53	500	10^{-3}

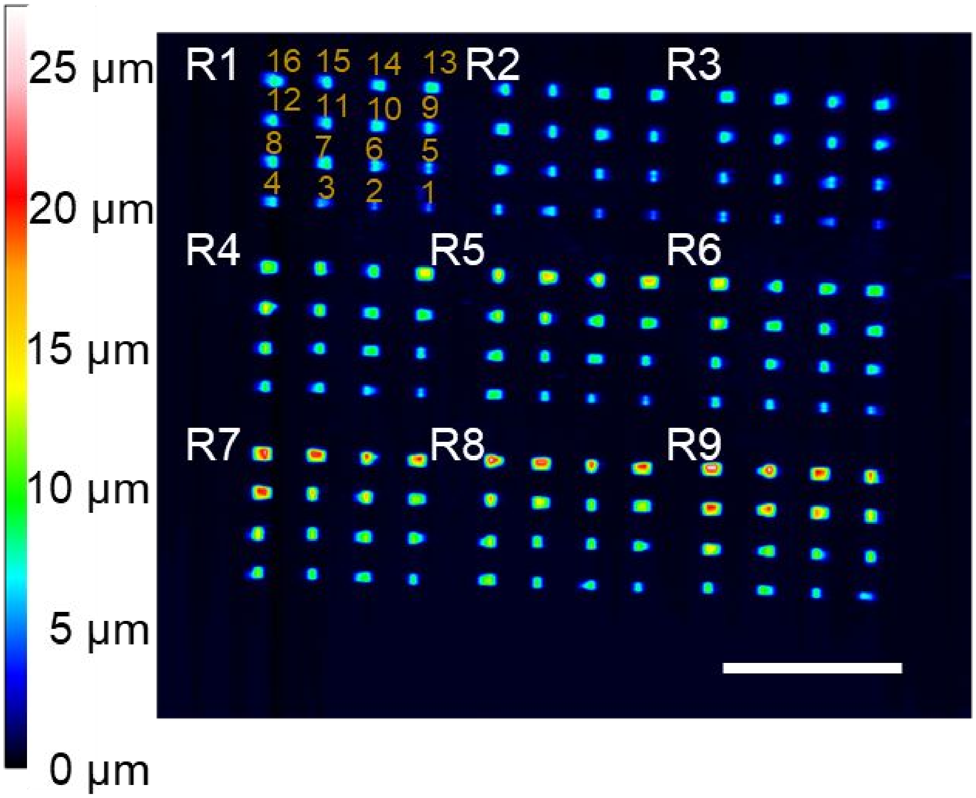

Figure S89. Profilometry image of patterns printed using [TPO] = 100 mM, [EGDMA] = 1300 mM. The pattern printed contains 9 repeats (R1-R9) of 16 different time points. The scale bar is 200 μm.

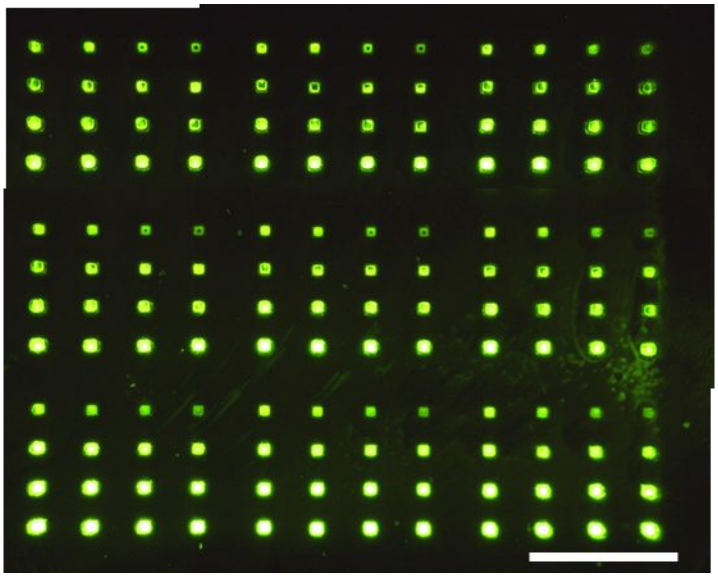

Figure S90. Fluorescence image of patterns printed using [TPO] = 100 mM, [EGDMA] = 1300 mM. Incubation was performed for 1 hour. The pattern printed is 9 repeats (R1-R9) of 16 different time points. The scale bar is 200 μm.

Table S15. Fluorescence intensity (*I*) and heights (*h*) of features printed under conditions in **Table S14**, and shown in **Figure S89** and **Figure S90**, # = feature number.

#	Time (min)	R1	*I*	R2	*I*	R3	*I*	R4	*I*	R5	*I*	R6	*I*	R7	*I*	R8	*I*	R9	*I*	Average Height (μm)
1	4.5	4.3	2.2	4.3	2.2	4.2	2.3	5.8	2.0	5.3	2.0	5.9	2.0	7.9	2.0	7.7	1.8	6.7	2.0	5.8
2	5	4.7	2.6	5.3	2.7	5.1	2.5	7.0	2.3	6.7	2.3	7.3	2.4	9.6	2.5	8.5	2.3	9.0	2.4	7.0
3	5.5	5.3	3.0	6.6	3.3	5.3	3.1	8.2	3.1	7.3	3.0	7.7	3.0	10.1	3.1	8.7	3.1	10.5	3.0	7.7
4	6	7.4	3.6	6.2	3.8	5.6	3.6	8.3	3.6	8.7	3.4	7.4	3.4	11.5	3.3	11.8	3.2	10.7	3.2	8.6
5	6.5	6.1	4.6	6.7	4.3	6.9	4.3	6.9	3.8	8.3	3.5	8.4	3.7	10.4	3.7	10.6	3.1	10.4	2.6	8.3
6	7	7.2	5.2	7.4	4.5	6.7	4.9	9.2	3.9	8.2	3.5	8.6	3.7	11.5	3.6	9.7	3.2	11.2	3.3	8.9
7	7.5	8.6	5.7	6.9	4.9	6.5	4.6	9.7	3.9	8.5	3.8	9.2	3.7	12.9	3.6	10.6	2.8	11.4	3.3	9.3
8	8	8.8	6.2	7.7	5.3	7.0	5.2	12.5	3.9	10.0	3.8	9.6	3.8	14.3	3.4	13.1	3.2	15.3	3.4	10.9
9	8.5	7.4	6.2	7.4	5.8	7.4	6.7	8.9	4.6	9.2	4.2	8.6	3.9	9.8	3.5	11.5	3.4	13.7	3.2	9.3
10	9	7.6	6.7	7.9	6.2	8.4	7.0	8.3	4.5	10.3	4.7	9.3	4.5	15.2	3.8	11.1	3.5	17.3	4.3	10.6
11	9.5	7.8	7.3	7.8	6.6	8.3	6.7	10.5	4.7	13.4	5.3	9.6	4.6	16.8	4.3	13.7	4.0	18.7	4.9	11.8
12	10	8.5	8.0	8.0	6.7	8.5	6.8	13.1	5.3	12.5	5.8	13.0	5.2	20.5	5.0	17.0	4.6	20.6	5.2	13.5
13	10.5	8.1	7.3	7.9	7.2	7.6	8.2	13.3	6.4	14.7	6.0	8.1	5.8	18.9	5.2	20.9	5.3	17.9	5.1	13.0
14	11	7.9	7.4	8.0	7.1	8.4	7.9	10.1	6.4	13.7	6.5	8.4	6.1	18.5	5.5	22.8	5.7	19.8	5.9	13.1
15	11.5	8.3	8.0	8.2	7.5	7.6	7.9	11.9	6.2	16.0	6.9	9.3	6.3	21.7	6.0	23.3	6.1	26.0	6.6	14.7
16	12	8.5	9.0	8.0	7.4	7.8	7.7	11.1	6.8	16.7	6.9	14.2	6.7	23.6	6.4	24.6	6.1	25.7	6.7	15.6

Table S16. Binding conditions for surfaces printed under conditions shown in **Table S13**.

TPO (mM)	PETT (mM)	EGDMA (mM)	Intensity (mW/mm^2)	[**SCR043**] (μM)	[α-**Man-FL**] (M)
1	100	1300	2.53	250	10^{-3}

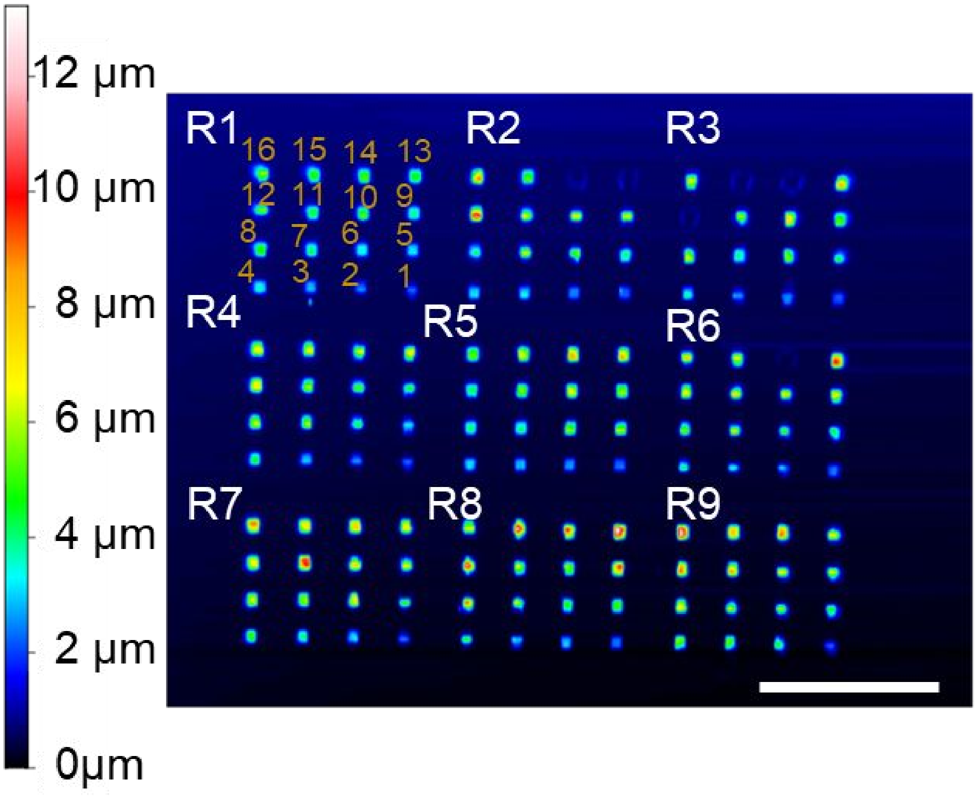

Figure S91. Profilometry image of patterns printed using [TPO] = 100 mM, [EGDMA] = 1300 mM. The pattern printed contains 9 repeats (R1-R9) of 16 different time points. The scale bar is 200 μm.

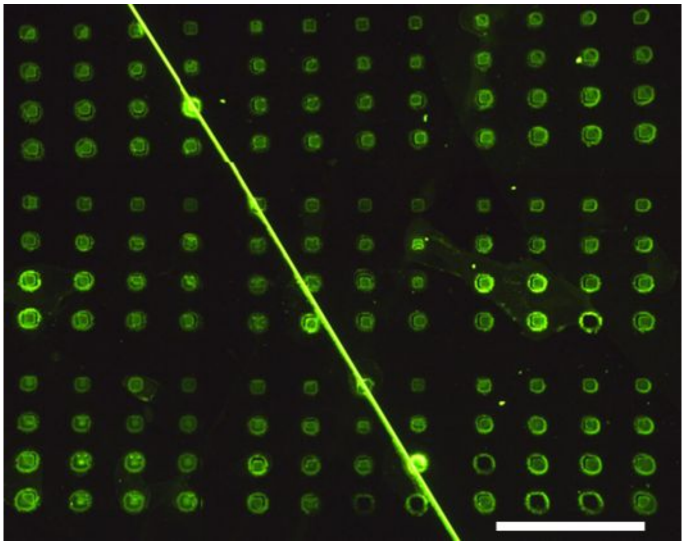

Figure S92. Fluorescence image of patterns printed using [TPO] =100 mM, [EGDMA] = 1300 mM. Incubation was performed for 1 hour. The pattern printed is 9 repeats (R1-R9) of 16 different time points. The scale bar is 200 μm.

Table S17. # = Fluorescence intensity (*I*) and heights (*h*) of features printed under conditions in **Table S16**, and shown in **Figure S91** and **Figure S92**, # = feature number.

#	Time (min)	R1	*I*	R2	*I*	R3	*I*	R4	*I*	R5	*I*	R6	*I*	R7	*I*	R8	*I*	R9	*I*	Average height (μm)
1	4.5	1.9	1.4	1.8	1.5	1.9	2.0	2.0	1.4	2.0	1.5	2.3	2.1	2.4	1.5	2.3	1.6	2.3	1.8	2.1
2	5	2.1	2.1	2.0	4.8	2.3	2.0	2.4	1.6	2.3	1.5	2.8	2.3	3.0	3.2	1.7	1.5	4.9	2.0	2.6
3	5.5	2.4	1.6	3.1	1.7	2.3	2.0	2.7	1.8	2.5	1.9	3.2	2.1	3.9	1.9	3.0	1.6	5.4	2.0	3.2
4	6	3.4	2.0	2.7	1.6	3.6	2.2	4.4	2.0	2.9	4.5	4.1	1.7	4.8	2.1	4.8	1.8	6.0	2.4	4.1
5	6.5	2.7	1.6	3.4	2.5	4.0	2.6	3.1	2.5	5.7	3.0	3.9	2.7	5.2	2.2	5.1	1.8	4.9	2.1	4.2
6	7	3.0	1.7	3.7	1.8	5.3	2.7	4.3	2.2	4.8	1.9	5.0	2.6	7.2	2.1	1.4	1.9	6.0	2.6	4.5
7	7.5	3.7	2.1	6.0	2.1	3.1	2.7	6.0	2.5	3.8	2.1	6.0	2.4	5.6	2.0	6.5	1.8	6.0	2.1	5.2
8	8	4.5	2.3	3.8	2.3	6.2	2.5	6.2	2.2	3.8	2.0	5.5	2.8	5.8	2.1	9.6	2.0	6.9	2.4	5.8
9	8.5	3.4	2.0	6.1	6.9	4.7	2.6	5.0	2.4	5.6	2.1	6.6	3.1	7.0	7.0	9.2	2.1	6.7	2.4	6.0
10	9	4.8	2.9	5.3	2.0	6.9	2.8	5.0	2.6	4.8	2.5	7.6	3.1	7.6	2.2	1.6	2.0	7.9	2.9	5.7
11	9.5	3.8	2.9	7.0	2.2	5.1	2.7	5.9	3.4	5.4	3.4	6.7	4.6	10.3	2.2	6.4	2.1	8.4	2.5	6.6
12	10	5.2	3.1	10.6	3.3	N/A	1.8	6.9	4.5	4.0	2.1	7.0	4.4	6.4	2.2	11.3	2.2	9.1	2.7	7.6
13	10.5	4.0	2.7	N/A	2.4	7.5	2.3	7.2	2.2	8.4	2.3	9.2	2.9	7.0	2.7	11.8	2.4	7.4	2.8	7.8
14	11	4.6	2.9	N/A	1.3	N/A	1.9	6.3	2.3	5.3	2.4	N/A	2.8	7.6	2.4	4.7	2.1	8.7	2.9	6.2
15	11.5	4.5	2.7	4.8	1.9	N/A	2.1	6.7	2.9	6.2	4.5	6.4	4.7	7.0	2.4	12.0	2.3	7.8	2.8	6.9
16	12	5.4	3.2	8.5	2.5	5.9	2.9	6.8	4.2	5.0	2.2	8.3	2.6	9.0	2.2	6.2	2.2	12.5	2.8	7.5

Table S18. Binding conditions for surfaces printed under conditions shown in **Table S13**.

TPO (mM)	PETT (mM)	EGDMA (mM)	Intensity (mW/mm^2)	[**SCR043**] (μM)	[α-**Man-FL**] (M)
1	100	1300	2.53	100	10^{-3}

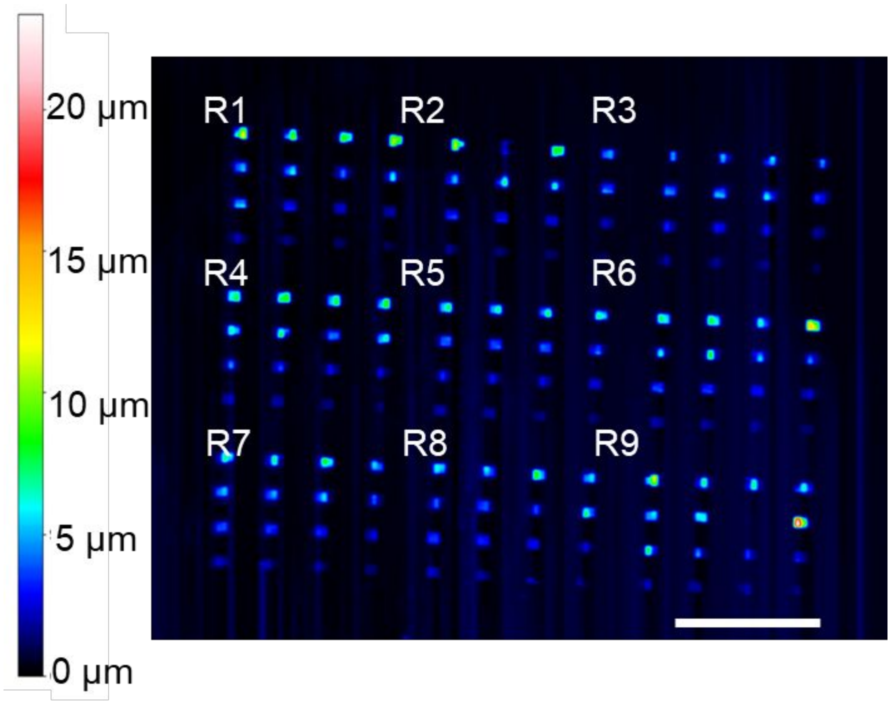

Figure S93. Profilometry image of patterns printed using [TPO] = 100 mM, [EGDMA] = 1300 mM. The pattern printed is 9 repeats (R1-R9) of 16 different time points. The scale bar is 200 μm.

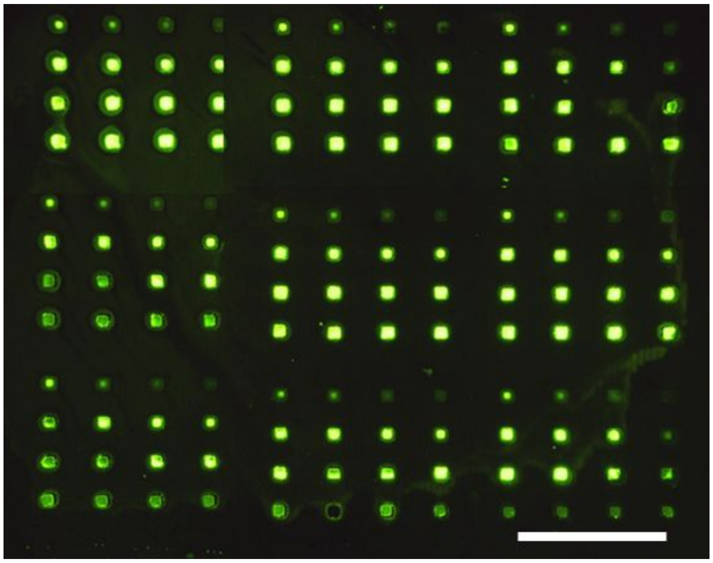

Figure S94. Fluorescence image of patterns printed using [TPO] = 100mM, [EGDMA = 1300mM. Incubation was performed for 1 hour. The pattern printed is 9 repeats (R1-R9) of 16 different time points. The scale bar is 200 μm.

Table S19. Fluorescence intensity (*I*) and heights (*h*) of features printed under conditions in **Table S18**, and shown in **Figure S93** and **Figure S94**, # = feature number.

#	Time (min)	R1	*I*	R2	*I*	R3	*I*	R4	*I*	R5	*I*	R6	*I*	R7	*I*	R8	*I*	R9	*I*	Average Height (μm)
1	3.5	1.9	2.4	1.6	1.7	1.6	2.1	1.8	2.4	1.8	2.6	1.7	2.5	2.3	2.5	2.1	2.9	2.1	2.7	1.9
2	4	1.4	1.6	1.3	1.5	1.3	1.6	1.5	1.7	1.4	1.8	1.4	1.6	2.1	1.8	1.5	1.9	1.1	1.6	1.5
3	4.5	1.0	1.3	1.0	1.3	1.0	1.5	1.3	1.5	1.2	1.4	1.1	1.4	1.4	1.6	2.0	1.4	1.7	1.2	1.3
4	5	0.9	1.2	0.8	1.2	0.8	1.1	1.0	1.4	0.9	1.2	0.8	1.6	1.0	1.4	1.4	1.2	1.1	1.1	1.0
5	5.5	5.5	2.7	3.6	4.1	2.8	4.8	4.2	4.3	3.1	4.9	3.9	5.0	3.5	5.2	3.2	5.4	7.5	5.0	4.2
6	6	3.1	4.2	3.0	4.2	2.5	4.4	3.5	4.4	2.8	4.3	3.2	4.6	3.2	4.9	2.9	4.8	4.6	4.6	3.2
7	6.5	2.2	3.5	2.2	3.7	2.3	4.0	2.8	3.9	2.5	3.9	2.2	4.0	2.7	4.6	2.6	4.2	2.6	3.8	2.5
8	7	1.8	2.7	2.0	3.0	1.6	1.3	2.3	3.6	1.9	3.3	2.0	3.2	2.5	4.0	2.3	3.6	2.9	1.5	2.1
9	7.5	5.1	2.9	5.2	4.8	4.2	5.7	6.3	2.6	3.8	6.0	6.4	5.6	4.8	4.7	3.9	6.2	5.5	5.3	5.0
10	8	6.2	2.7	6.1	3.6	4.7	5.8	7.7	2.7	4.0	5.7	7.9	5.5	5.0	6.2	3.6	5.9	5.7	5.0	5.7
11	8.5	4.1	4.2	6.7	4.1	5.7	3.8	4.2	5.0	3.9	5.5	5.1	5.5	5.6	6.1	3.6	5.9	0.2	N/A	4.4
12	9	6.1	4.3	4.5	5.2	3.3	2.0	6.4	5.1	4.2	5.3	4.3	5.3	4.0	6.0	7.1	5.7	22.7	2.9	7.0
13	9.5	12.1	2.7	10.4	2.6	4.5	1.6	7.6	2.7	7.4	5.1	7.1	5.7	6.5	5.9	5.6	6.2	11.6	4.0	8.1
14	10	9.3	2.5	2.8	N/A	4.9	1.6	8.5	2.9	7.0	5.5	7.5	6.2	6.6	6.3	5.7	6.3	6.6	5.1	6.6
15	10.5	8.7	2.3	8.5	2.7	5.6	1.6	8.0	3.2	7.3	5.8	6.0	6.1	8.3	6.5	8.4	5.9	6.2	5.8	7.4
16	11	10.1	2.2	4.4	2.0	4.3	1.6	8.7	3.1	6.8	5.7	13.6	5.1	5.3	6.1	5.2	5.7	5.9	4.7	7.1

Table S20. Binding conditions for surfaces printed under conditions shown in **Table S13**.

TPO (mM)	PETT (mM)	EGDMA (mM)	Intensity (mW/mm^2)	[**SCR043**] (µM)	[α-**Man-FL**] (M)
1	100	1300	2.53	50.0	10^{-3}

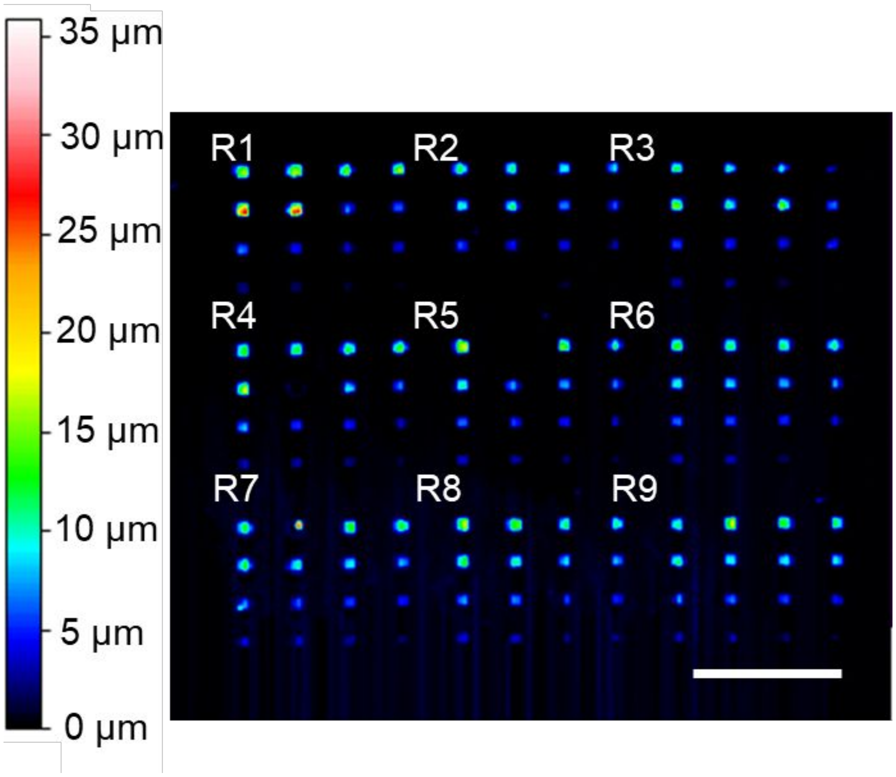

Figure S95. Profilometry image of patterns printed using [TPO] = 100 mM, [EGDMA] = 1300 mM. The pattern printed contains 9 repeats (R1-R9) of 16 different time points. The scale bar is 200 µm.

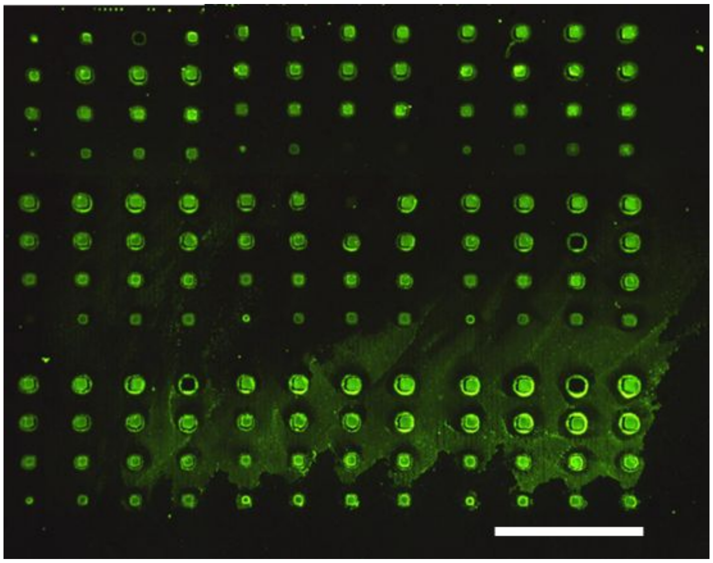

Figure S96. Fluorescence image of patterns printed using [TPO] =100 mM, [EGDMA] =1300 mM. Incubation was performed for 1 hour. The pattern printed is 9 repeats (R1-R9) of 16 different time points. The scale bar is 200 μm.

Table S21. Fluorescence intensity (*I*) and heights (*h*) of features printed under conditions in **Table S20**, and shown in **Figure S95** and **Figure S96**, # = feature number.

#	Time (min)	R1	*I*	R2	*I*	R3	*I*	R4	*I*	R5	*I*	R6	*I*	R7	*I*	R8	*I*	R9	*I*	Average Height (μm)
1	3.4	1.6	1.3	3.0	2.2	3.1	2.2	1.5	1.0	1.7	1.6	2.0	1.6	0.8	1.0	1.0	1.2	1.3	1.2	1.8
2	3.9	1.1	1.4	1.7	2.2	2.3	2.3	1.1	1.5	1.2	1.4	1.5	1.6	0.8	1.4	0.8	1.3	0.1	1.2	1.2
3	4.4	0.9	1.7	1.4	2.5	1.7	2.5	0.8	1.6	1.0	1.6	1.1	1.8	0.5	1.6	0.8	1.0	0.7	1.4	1.0
4	4.9	0.6	2.0	0.9	2.4	1.0	2.4	0.4	1.8	0.8	1.9	0.9	2.0	0.3	1.7	0.2	1.1	0.2	1.8	0.6
5	5.4	4.9	1.8	5.0	2.3	7.9	2.6	4.9	1.7	3.6	1.8	4.0	2.0	1.7	1.8	1.9	1.7	1.9	1.9	4.0
6	5.9	3.6	2.1	5.6	2.7	6.4	3.2	2.2	2.1	2.5	2.1	2.7	2.4	1.4	2.2	1.5	2.0	1.8	2.0	3.1
7	6.4	3.3	2.2	3.8	2.9	5.0	3.7	1.7	2.5	2.1	2.3	2.3	2.9	1.3	2.6	1.3	2.3	1.5	2.4	2.5
8	6.9	1.8	2.4	3.3	2.9	4.4	3.3	1.4	2.4	1.6	2.4	2.0	2.9	1.1	2.7	2.1	2.6	1.1	2.6	2.1
9	7.4	6.2	2.1	7.0	2.7	7.3	3.6	5.1	2.2	5.1	2.5	3.9	2.5	2.5	1.9	2.5	2.6	2.6	2.7	4.7
10	7.9	6.1	2.3	5.4	3.1	7.6	3.8	5.1	2.5	4.5	2.5	6.7	2.8	2.2	2.6	2.2	2.4	3.4	3.1	4.8
11	8.4	5.9	2.5	5.6	3.1	7.4	4.2	3.7	2.7	4.7	2.5	5.0	1.8	2.0	2.7	2.1	2.4	2.2	2.7	4.3
12	8.9	5.8	3.0	4.8	3.6	6.4	3.9	2.7	2.7	3.7	2.7	4.4	2.8	1.8	2.8	1.9	2.5	1.9	2.6	3.7
13	9.4	6.2	2.5	7.0	3.1	7.5	3.5	5.0	2.4	6.1	2.4	4.9	2.6	2.7	1.4	2.8	2.0	0.0	2.4	4.7
14	9.9	6.3	3.0	6.8	3.5	7.8	4.1	5.5	3.0	6.0	2.4	7.2	2.8	3.5	1.8	2.6	2.2	2.2	2.6	5.3
15	10.4	6.6	3.1	7.4	3.5	7.2	2.5	5.2	3.1	4.8	1.1	6.8	3.0	4.0	1.2	2.6	2.4	2.3	2.8	5.2
16	10.9	6.1	2.1	6.8	3.7	7.0	3.9	5.3	3.1	4.4	3.1	5.5	3.3	2.3	2.2	2.4	2.6	2.3	3.0	4.7

Table S22. Binding conditions for surfaces printed under conditions shown in **Table S13**.

TPO (mM)	PETT (mM)	EGDMA (mM)	Intensity (mW/mm2)	[**SCR043**] (μM)	[α-**Man-FL**] (M)
1	100	1300	2.53	12.5	10^{-3}

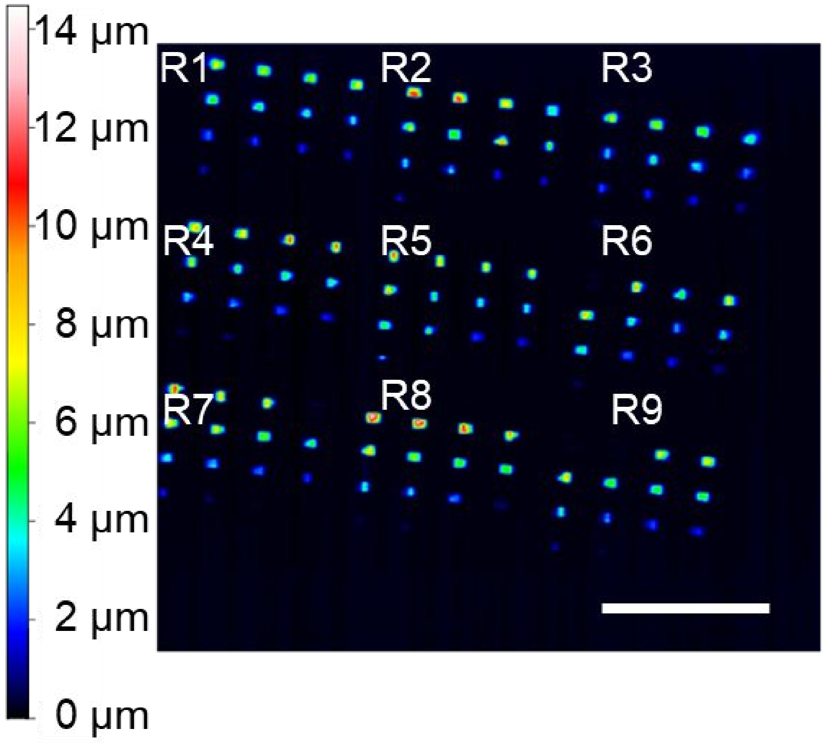

Figure S97. Profilometry image of patterns printed using [TPO] = 100 mM, [EGDMA] = 1300 mM. The pattern printed is 9 repeats (R1-R9) of 16 different time points. The scale bar is 200 μm.

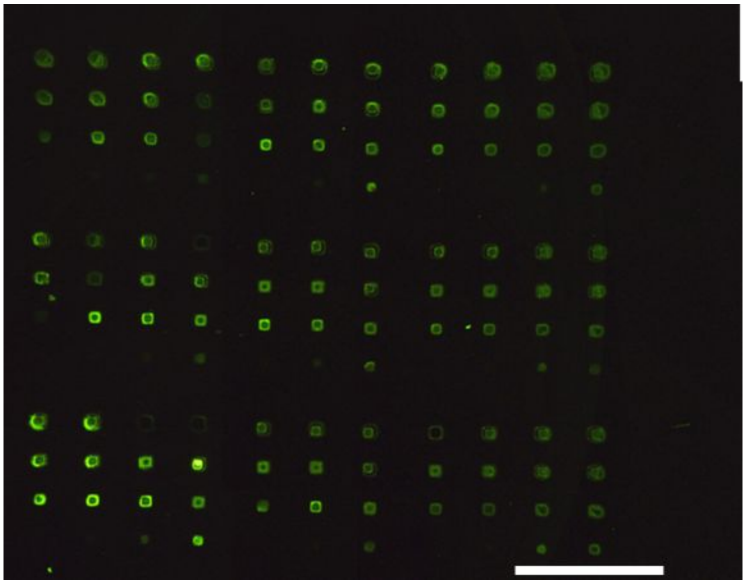

Figure S98. Fluorescence image of patterns printed using [TPO] =100 mM, [EGDMA] = 1300 mM. Incubation was performed for 1 hour. The pattern printed is 9 repeats (R1-R9) of 16 different time points. The scale bar is 200 μm.

Table S23. Fluorescence intensity (*I*) and heights (*h*) of features printed under conditions in **Table S22**, and shown in **Figure S97** and **Figure S98**, # = feature number.

#	Time (min)	R1	*I*	R2	*I*	R3	*I*	R4	*I*	R5	*I*	R6	*I*	R7	*I*	R8	*I*	R9	*I*	Average Height (μm)
1	3.4	N/A	2.0	N/A	1.3	N/A	1.3	N/A	1.2	N/A	1.4	N/A	1.2	N/A	1.1	N/A	1.5	N/A	1.3	N/A
2	3.9	N/A	1.1	N/A	1.2	N/A	1.3	N/A	1.1	N/A	1.2	N/A	1.2	N/A	1.1	N/A	1.1	N/A	1.1	N/A
3	4.4	N/A	1.0	N/A	1.0	N/A	1.0	N/A	1.0	N/A	1.0	N/A	1.0	0.5	1.0	0.5	1.0	0.4	1.0	0.5
4	4.9	1.2	1.0	0.7	1.0	0.6	1.0	N/A	1.0	N/A	1.0	N/A	1.0	1.4	1.0	0.3	1.0	1.1	1.0	0.9
5	5.4	1.1	1.9	1.1	1.8	0.9	1.5	0.9	2.0	1.6	1.8	0.9	1.5	2.1	1.2	0.7	1.8	1.2	1.5	1.2
6	5.9	1.4	2.5	1.8	1.8	1.5	1.5	1.2	2.3	1.9	1.9	1.8	1.4	2.4	1.7	2.4	1.8	2.2	1.5	1.9
7	6.4	2.1	2.8	1.9	2.0	2.0	1.3	1.8	2.5	3.3	2.1	2.3	1.5	3.4	1.9	4.0	2.2	2.4	1.5	2.6
8	6.9	2.5	2.4	2.7	1.5	2.1	1.4	1.7	1.1	2.8	2.3	4.3	1.6	3.7	1.2	4.5	1.8	4.2	1.6	3.2
9	7.4	3.6	3.1	5.4	1.6	2.7	1.5	2.9	1.8	4.1	1.7	4.3	1.7	4.9	1.2	5.4	1.9	4.1	1.7	4.2
10	7.9	4.1	2.3	4.2	1.9	3.8	1.6	3.5	2.0	4.2	1.8	3.0	1.7	5.6	2.0	5.4	2.0	4.8	1.7	4.3
11	8.4	4.2	2.6	4.8	2.0	4.3	1.6	4.0	1.3	5.0	1.9	4.1	1.7	7.1	1.8	6.0	1.6	5.3	1.8	5.0
12	8.9	5.0	2.3	5.8	1.9	3.4	1.7	5.8	1.9	5.7	1.9	8.7	1.6	7.5	1.5	8.2	1.9	9.0	1.8	6.6
13	9.4	6.9	1.1	3.9	1.6	4.4	1.6	10.4	1.1	8.6	1.7	7.8	1.6	N/A	2.1	10.1	1.9	6.6	1.7	7.3
14	9.9	5.9	1.1	7.1	1.6	5.5	1.6	12.1	1.8	8.5	1.6	5.1	1.6	8.2	2.2	11.2	2.0	6.8	1.8	7.8
15	10.4	6.3	2.5	12.6	1.6	5.7	1.6	7.7	1.4	8.1	1.7	8.9	1.6	9.0	1.7	14.0	1.6	N/A	2.0	9.0
16	10.9	7.2	2.6	11.6	1.6	5.8	1.3	7.3	2.0	11.7	1.7	N/A	1.5	11.2	1.6	13.4	1.3	N/A	1.9	9.7

Table S24. Binding conditions for surfaces printed under conditions shown in **Table S13**.

TPO (mM)	PETT (mM)	EGDMA (mM)	Intensity (mW/mm^2)	[**SCR043**] (μM)	[α-**Man-FL**] (M)
1	100	1300	2.53	0	10^{-3}

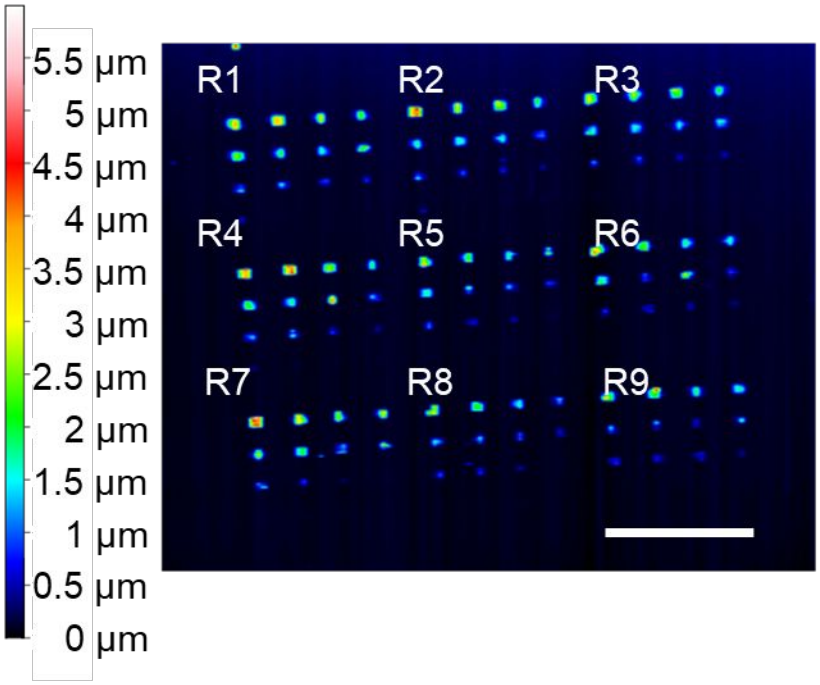

Figure S99. Profilometry image of patterns printed using [TPO] = 100 mM, [EGDMA] = 1300 mM. The pattern printed contains 9 repeats (R1-R9) of 16 different time points. The scale bar is 200 μm.

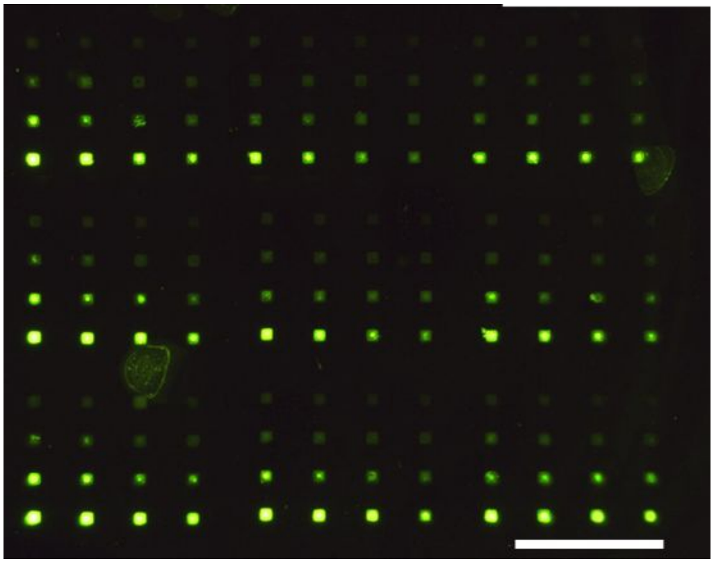

Figure S100. Fluorescence image of patterns printed using [TPO] = 100 mM, [EGDMA] = 1300 mM. Incubation was performed for 1 hour. The pattern printed is 9 repeats (R1-R9) of 16 different time points. The scale bar is 200 μm.

Table S25. Fluorescence intensity (*I*) and heights (*h*) of features printed under conditions in **Table S24**, and shown in **Figure S99** and **Figure S100**, # = feature number.

#	Time (min)	R1	*I*	R2	*I*	R3	*I*	R4	*I*	R5	*I*	R6	*I*	R7	*I*	R8	*I*	R9	*I*	Average Height (μm)
1	3.4	8.6	2.0	N/A	1.7	9.2	2.6	9.4	2.5	5.0	2.8	7.4	3.1	4.9	2.1	7.0	2.5	7.7	2.6	7.4
2	3.9	8.4	2.4	7.9	2.9	12.3	3.2	6.2	2.9	4.5	2.7	6.7	2.6	5.2	1.5	8.1	1.7	11.5	2.6	7.9
3	4.4	9.1	2.3	11.1	2.8	11.1	2.9	N/A	2.3	7.2	2.3	14.9	2.2	5.6	1.4	12.1	2.7	9.4	2.8	10.0
4	4.9	9.0	2.1	11.9	1.3	16.1	2.3	6.6	2.1	10.0	1.9	6.4	1.3	10.7	1.4	29.3	2.7	27.8	2.6	14.2
5	5.4	11.7	2.7	12.1	3.5	13.1	3.1	8.8	3.2	8.5	3.6	7.3	2.6	6.3	3.6	32.0	4.3	11.1	3.3	12.3
6	5.9	12.8	3.1	11.2	3.6	13.2	1.3	8.1	2.5	3.1	4.0	10.6	3.8	15.6	3.6	14.9	4.0	11.5	3.3	11.2
7	6.4	13.4	3.1	13.0	4.0	9.6	3.8	14.8	2.7	14.1	3.8	10.6	3.4	11.8	3.4	35.6	3.7	14.4	2.7	15.2
8	6.9	14.0	3.0	10.5	3.4	13.3	3.9	15.8	2.7	3.6	3.1	12.9	3.2	9.7	1.6	10.9	3.0	14.2	2.8	11.7
9	7.4	11.7	2.6	12.1	2.2	13.9	1.7	14.1	2.6	24.8	3.8	12.0	4.1	41.8	3.5	17.0	4.5	14.5	3.8	18.0
10	7.9	13.7	2.8	11.5	3.7	N/A	1.8	16.6	3.5	15.9	3.7	11.2	4.2	16.1	3.3	17.8	4.2	10.0	3.6	14.1
11	8.4	12.2	3.0	15.0	3.7	N/A	1.6	17.7	3.3	10.8	3.7	9.3	3.9	12.0	3.4	22.5	3.9	13.0	3.1	14.1
12	8.9	7.9	3.1	12.2	3.6	15.0	3.7	15.5	3.2	12.2	2.6	15.6	3.8	15.2	2.5	37.3	3.5	12.9	2.9	16.0
13	9.4	14.5	2.3	23.1	2.9	18.7	1.7	11.9	2.9	11.6	3.9	12.2	4.1	14.0	2.9	15.0	4.6	41.7	3.9	18.1
14	9.9	16.4	2.3	16.1	3.3	17.7	4.1	14.4	3.1	14.5	4.0	14.4	4.2	17.4	3.0	17.8	4.3	14.5	3.6	15.9
15	10.4	15.2	2.4	16.5	3.2	16.1	3.8	16.2	3.0	19.1	3.7	13.1	4.2	13.5	3.0	18.5	3.9	16.7	3.2	16.1
16	10.9	17.3	1.8	17.8	3.4	N/A	4.2	15.2	2.7	17.6	3.7	N/A	4.1	13.8	2.5	28.7	3.4	17.7	2.8	18.3

Table S26. Binding conditions for surfaces printed under conditions shown in **Table S13**.

TPO (mM)	PETT (mM)	EGDMA (mM)	Intensity (mW/mm^2)	[**SCR043**] (μM)	[α-**Man-FL**] (M)
1	100	1300	2.53	500	$10^{-3.5}$

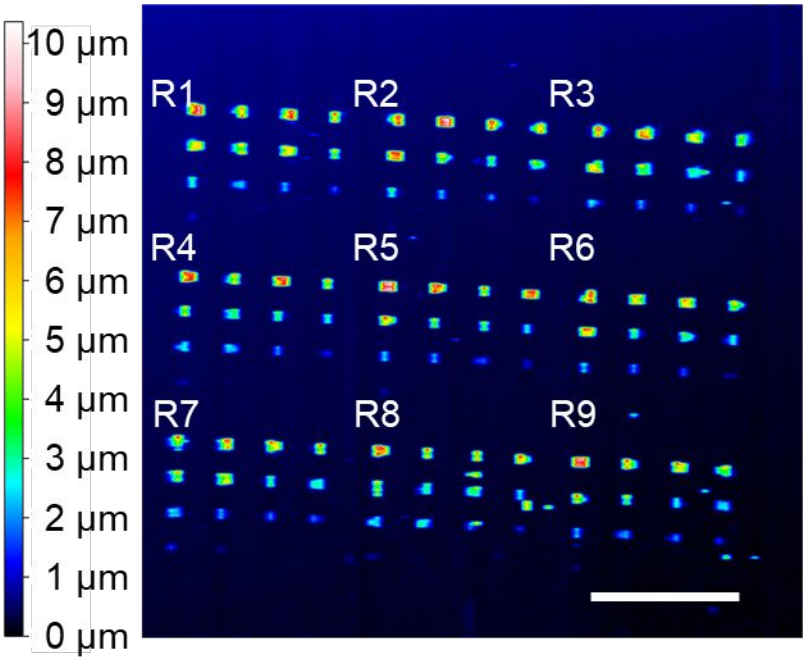

Figure S101. Profilometry image of patterns printed using [TPO] = 100 mM, [EGDMA] = 1300 mM. The pattern printed is 9 repeats (R1-R9) of 16 different time points. The scale bar is 200 μm.

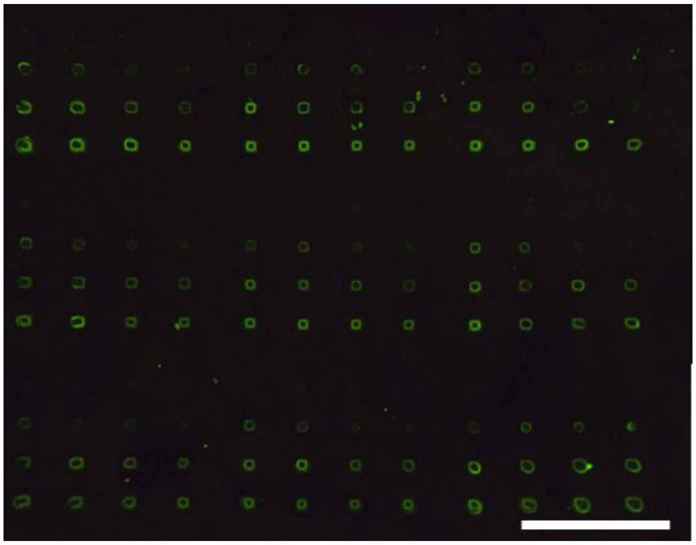

Figure S102. Fluorescence image of patterns printed using [TPO] = 100 mM, [EGDMA] = 1300 mM. Incubation was performed for 1 hour. The pattern printed is 9 repeats (R1-R9) of 16 different time points. The scale bar is 200 µm.

Table S27. Fluorescence intensity (*I*) and heights (*h*) of features printed under conditions in **Table S26**, and shown in **Figure S101** and **Figure S102**, # = feature number.

#	Time (min)	R1	*I*	R2	*I*	R3	*I*	R4	*I*	R5	*I*	R6	*I*	R7	*I*	R8	*I*	R9	*I*	Average Height (µm)
1	4.5	N/A	N/A	N/A	1.0	N/A	1.0	N/A	N/A	N/A	1.1	N/A	1.1	N/A	N/A	N/A	1.0	N/A	1.0	N/A
2	5	N/A	N/A	N/A	1.0	N/A	1.0	N/A	N/A	N/A	1.1	N/A	1.1	N/A	N/A	N/A	1.0	N/A	1.1	N/A
3	5.5	N/A	N/A	N/A	1.0	N/A	1.1	N/A	N/A	N/A	1.1	N/A	1.1	N/A	N/A	N/A	1.0	N/A	1.0	N/A
4	6	N/A	N/A	N/A	1.0	N/A	1.0	N/A	N/A	N/A	1.0	N/A	1.1	N/A	N/A	N/A	1.1	N/A	1.0	N/A
5	6.5	1.5	1.0	2.2	1.0	2.3	1.1	2.0	1.1	1.9	1.1	2.1	1.1	1.8	1.1	2.2	1.1	2.1	1.1	2.0
6	7	1.6	1.1	1.7	1.1	1.8	1.1	1.7	1.1	1.8	1.1	2.0	1.1	1.9	1.1	2.9	1.2	1.7	1.1	1.9
7	7.5	1.4	1.1	1.8	1.1	1.6	1.2	1.4	1.2	1.1	1.2	1.5	1.3	1.9	1.2	1.5	1.3	1.5	1.1	1.5
8	8	0.4	1.2	0.8	1.2	1.2	1.2	0.3	1.3	0.7	1.3	0.9	1.3	0.9	1.4	1.3	1.2	1.5	1.2	0.9
9	8.5	5.4	1.3	7.4	1.2	5.4	1.3	5.8	1.2	4.9	1.2	5.1	1.3	5.5	1.2	5.6	1.4	4.0	1.1	5.4
10	9	4.3	1.3	4.3	1.3	4.5	1.3	2.9	1.3	2.9	1.3	2.9	1.4	4.2	1.4	3.2	1.3	4.1	1.2	3.7
11	9.5	3.2	1.6	2.7	1.5	5.3	1.6	3.1	1.3	2.6	1.3	2.8	1.3	2.6	1.7	2.6	1.5	2.6	1.5	3.1
12	10	2.3	1.4	4.2	1.3	5.3	1.5	2.5	1.4	2.2	1.4	2.3	1.5	2.2	1.7	2.2	1.8	2.5	1.6	2.9
13	10.5	7.3	1.5	6.4	1.4	9.0	1.5	8.2	1.5	9.0	1.5	7.4	1.5	8.2	1.7	7.6	1.6	4.7	1.6	7.5
14	11	6.5	1.3	8.7	1.3	5.8	1.3	4.9	1.5	8.9	1.5	4.0	1.5	4.6	1.9	6.2	1.7	6.2	1.9	6.2
15	11.5	4.9	1.3	6.5	1.4	8.3	1.4	6.0	1.7	6.2	1.6	7.3	1.5	4.8	2.0	6.3	1.8	4.9	1.8	6.1
16	12	3.9	1.5	5.9	1.5	5.9	1.5	3.8	1.6	8.0	1.6	3.8	1.7	4.3	2.0	6.1	1.8	3.8	1.9	5.0

Table S28. Binding conditions for surfaces printed under conditions shown in **Table S13**.

TPO (mM)	PETT (mM)	EGDMA (mM)	Intensity (mW/mm^2)	[**SCR043**] (μM)	[α-**Man-FL**] (M)
1	100	1300	2.53	250	$10^{-3.5}$

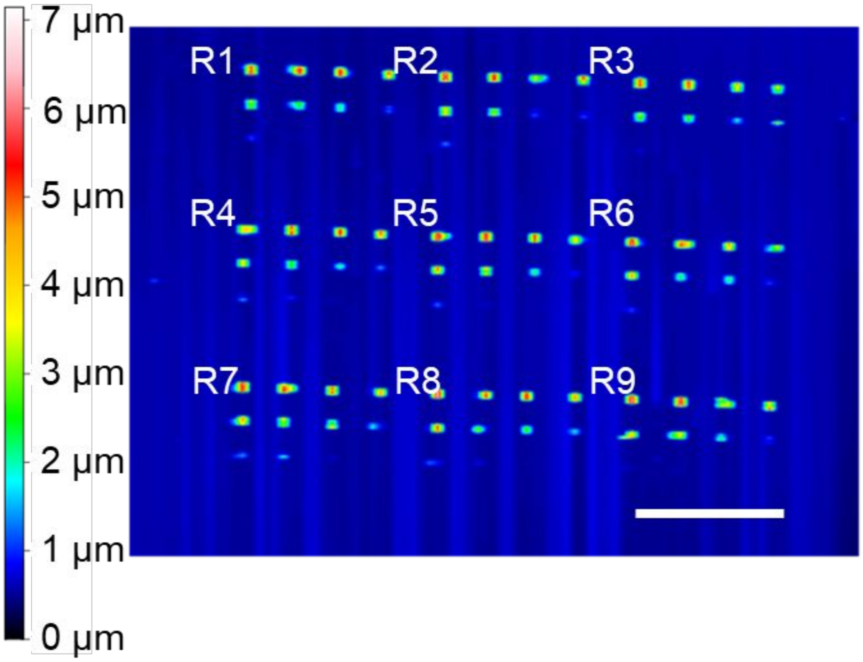

Figure S103. Profilometry image of patterns printed using [TPO] = 100 mM, [EGDMA] = 1300 mM. The pattern printed is 9 repeats (R1-R9) of 16 different time points. The scale bar is 200 μm.

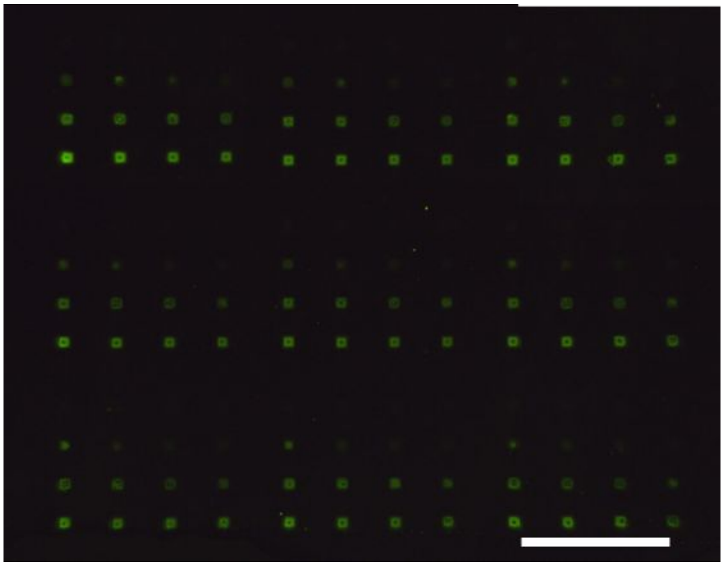

Figure S104. Fluorescence image of patterns printed using [TPO] = 100 mM, [EGDMA] = 1300 mM. Incubation was performed for 1 hour. The pattern printed is 9 repeats (R1-R9) of 16 different time points. The scale bar is 200 µm.

Table S29. Fluorescence intensity (*I*) and heights (*h*) of features printed under conditions in **Table S28**, and shown in **Figure S103** and **Figure S104**, # = feature number.

#	Time (min)	R1	*I*	R2	*I*	R3	*I*	R4	*I*	R5	*I*	R6	*I*	R7	*I*	R8	*I*	R9	*I*	Average Height (µm)
1	4.5	N/A	1.0	N/A	1.1	N/A	N/A	N/A	1.0	N/A	1.0	N/A	1.1	N/A	1.0	N/A	1.0	N/A	1.1	N/A
2	5	N/A	1.1	N/A	1.1	N/A	N/A	N/A	1.0	N/A	1.0	N/A	1.1	N/A	1.0	N/A	1.0	N/A	1.1	N/A
3	5.5	N/A	1.1	N/A	1.1	N/A	N/A	N/A	1.0	N/A	1.1	N/A	1.1	N/A	1.0	N/A	1.0	N/A	1.0	N/A
4	6	N/A	1.1	N/A	1.1	N/A	N/A	N/A	1.0	N/A	1.1	N/A	1.1	N/A	1.0	N/A	1.0	N/A	1.1	N/A
5	6.5	N/A	1.1	N/A	1.1	N/A	1.0	N/A	1.1	N/A	1.1	N/A	1.2	N/A	1.1	N/A	1.1	N/A	1.1	N/A
6	7	N/A	1.1	N/A	1.1	N/A	1.1	N/A	1.1	N/A	1.1	N/A	1.2	N/A	1.1	N/A	1.1	N/A	1.1	N/A
7	7.5	N/A	1.1	N/A	1.1	N/A	1.1	N/A	1.2	N/A	1.2	N/A	1.2	N/A	1.2	N/A	1.2	N/A	1.2	N/A
8	8	N/A	1.3	N/A	1.2	N/A	1.2	N/A	1.2	N/A	1.2	N/A	1.3	N/A	1.2	N/A	1.2	N/A	1.3	N/A
9	8.5	0.7	1.3	0.3	1.3	N/A	1.2	3.9	1.3	3.6	1.4	3.4	1.4	3.7	1.3	4.3	1.3	3.6	1.3	2.9
10	9	1.7	1.3	0.4	1.4	1.0	1.3	1.8	1.3	2.6	1.4	1.9	1.5	3.1	1.4	2.3	1.3	2.2	1.3	1.9
11	9.5	1.9	1.4	2.3	1.4	2.5	1.3	1.6	1.4	1.5	1.5	1.3	1.5	1.5	1.5	2.2	1.5	2.0	1.5	1.9
12	10	2.3	1.5	3.6	1.5	2.9	1.5	0.3	1.5	N/A	1.5	0.6	1.6	0.7	1.7	1.1	1.5	1.1	1.6	1.6
13	10.5	3.6	1.5	2.9	1.5	2.9	1.4	5.2	1.5	5.2	1.5	3.4	1.6	5.4	1.6	5.6	1.6	5.1	1.6	4.4
14	11	5.3	1.6	2.8	1.6	3.5	1.6	4.4	1.6	5.3	1.6	3.8	1.7	4.9	1.7	4.5	1.6	4.6	1.8	4.3
15	11.5	5.4	1.6	5.0	1.6	4.4	1.7	4.1	1.7	4.2	1.6	4.8	1.8	2.8	2.0	4.9	1.7	3.6	1.8	4.4
16	12	4.3	1.8	4.9	1.8	4.1	1.8	2.8	1.9	3.4	1.7	4.4	1.8	2.9	2.3	4.0	1.8	3.8	1.8	3.9

Table S30. Binding conditions for surfaces printed under conditions shown in **Table S13**.

TPO (mM)	PETT (mM)	EGDMA (mM)	Intensity (mW/mm^2)	[**SCR043**] (μM)	[α-**Man-FL**] (M)
1	100	1300	2.53	100	$10^{-3.5}$

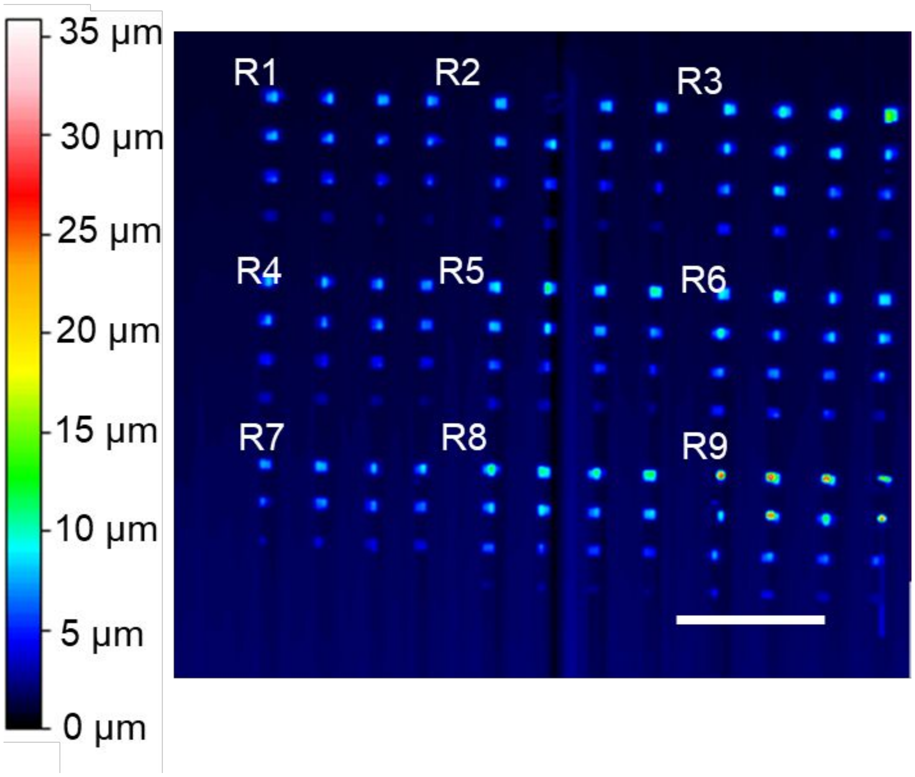

Figure S105. Profilometry image of patterns printed using [TPO] = 100 mM, [EGDMA] = 1300 mM. The pattern printed is 9 repeats (R1-R9) of 16 different time points. The scale bar is 200 μm.

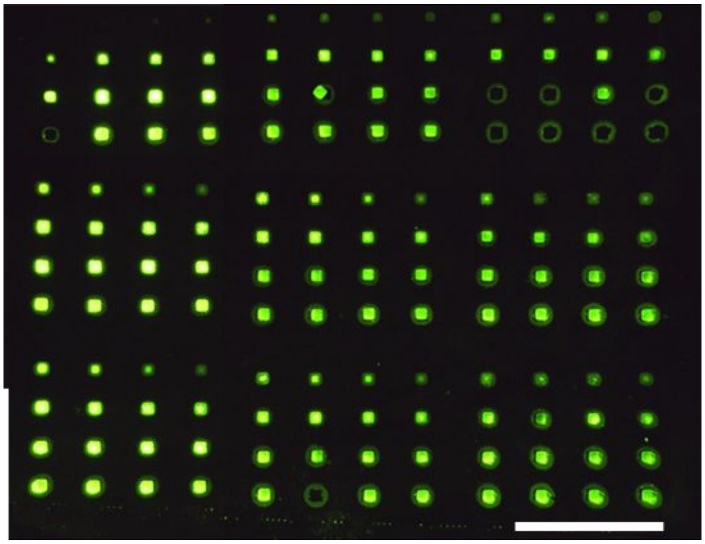

Figure S106. Fluorescence image of patterns printed using [TPO] = 100 mM, [EGDMA] = 1300 mM. Incubation was performed for 1 hour. The pattern printed is 9 repeats (R1-R9) of 16 different time points. The scale bar is 200 µm.

Table S31. Fluorescence intensity (*I*) and heights (*h*) of features printed under conditions in **Table S30**, and shown in **Figure S105** and **Figure S106**, # = feature number.

#	Time (min)	R1	*I*	R2	*I*	R3	*I*	R4	*I*	R5	*I*	R6	*I*	R7	*I*	R8	*I*	R9	*I*	Average Height (µm)
1	3.5	2.2	3.9	3.5	3.0	3.9	2.4	2.0	3.8	2.6	4.1	3.8	2.6	N/A	1.0	1.4	1.4	3.1	1.5	2.8
2	4	1.7	3.0	2.2	2.6	4.4	2.1	1.7	3.2	2.0	3.2	5.5	1.7	N/A	1.0	1.4	1.4	2.8	1.5	2.7
3	4.5	1.5	1.9	1.8	2.2	4.5	2.0	1.4	2.2	1.8	2.3	4.6	1.8	N/A	1.0	0.9	1.3	1.6	1.5	2.3
4	5	1.1	1.5	1.4	1.6	1.7	1.7	1.2	1.6	1.5	1.7	1.9	1.9	N/A	1.0	0.9	1.2	1.2	1.4	1.4
5	5.5	5.8	6.4	5.2	4.2	6.4	4.1	3.8	6.5	5.3	4.9	6.6	3.3	2.2	1.9	4.9	3.5	6.4	3.0	5.2
6	6	5.5	5.6	4.7	4.5	8.4	3.0	3.9	6.0	4.6	4.7	6.0	3.2	2.9	3.9	5.0	4.3	6.7	3.0	5.3
7	6.5	4.7	4.9	4.3	3.7	5.9	4.4	4.1	5.6	4.1	3.7	5.0	3.4	2.8	4.1	4.2	3.4	5.3	3.4	4.5
8	7	5.3	4.1	4.1	3.1	6.0	3.0	3.3	4.9	4.1	3.6	7.0	2.8	2.6	3.8	3.8	2.9	5.1	2.8	4.6
9	7.5	7.3	6.5	6.9	4.8	8.0	4.9	7.0	7.4	7.3	4.4	10.0	4.0	5.4	4.7	7.4	4.1	8.8	N/A	7.6
10	8	7.2	6.4	8.6	3.9	8.0	4.6	6.5	7.2	8.9	3.9	7.3	4.0	6.1	7.2	8.2	3.7	32.3	N/A	10.4
11	8.5	6.8	6.2	6.7	4.3	8.8	4.1	7.0	7.4	7.0	4.0	7.6	4.0	5.9	7.0	6.8	3.8	11.1	N/A	7.5
12	9	6.6	5.9	7.2	4.1	8.1	3.9	5.5	6.2	6.6	3.9	7.8	3.9	5.4	6.3	7.4	3.3	27.3	N/A	9.1
13	9.5	7.5	6.7	7.2	5.2	9.2	5.1	7.5	6.6	8.3	4.7	8.4	4.9	6.4	N/A	10.7	4.9	34.0	N/A	11.0
14	10	7.8	6.3	1.3	N/A	9.3	4.9	7.4	6.7	11.0	4.5	9.3	4.7	6.8	6.5	9.7	4.8	35.2	N/A	10.9
15	10.5	7.1	6.2	7.4	4.8	9.7	4.7	7.1	6.6	8.8	4.8	7.9	4.8	7.2	6.3	10.6	4.5	32.7	N/A	10.9
16	11	6.8	5.6	8.0	4.7	15.0	4.4	6.3	6.1	10.0	4.4	8.0	4.4	8.1	5.2	11.0	4.2	18.2	N/A	10.1

Table S32. Binding conditions for surfaces printed under conditions shown in **Table S13**.

TPO (mM)	PETT (mM)	EGDMA (mM)	Intensity (mW/mm^2)	[**SCR043**] (μM)	[α-**Man-FL**] (M)
1	100	1300	2.53	50.0	$10^{-3.5}$

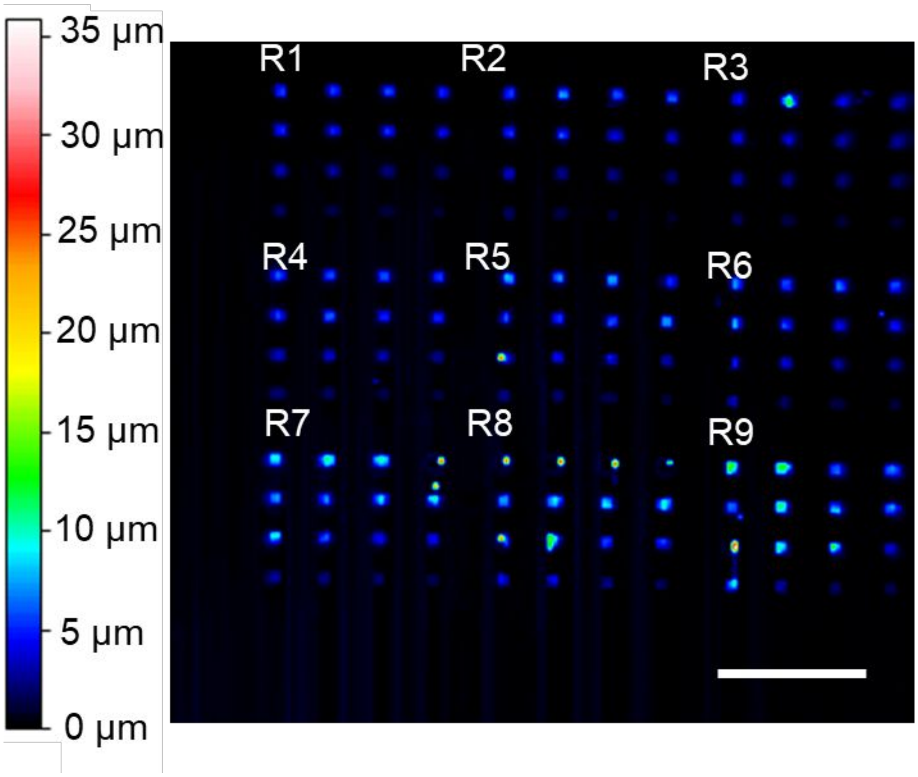

Figure S107. Profilometry image of patterns printed using [TPO] = 100 mM, [EGDMA] = 1300 mM. The pattern printed is 9 repeats (R1-R9) of 16 different time points. The scale bar is 200 μm.

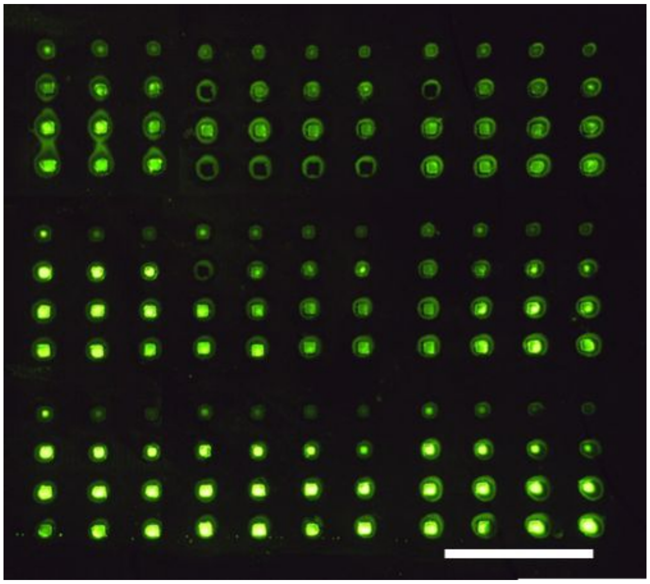

Figure S108. Fluorescence image of patterns printed using [TPO:100mM], [EGDMA 1300mM. Incubation was performed for 1 hour. The pattern printed is 9 repeats (R1-R9) of 16 different time points. The scale bar is 200 μm.

Table S33. Fluorescence intensity (*I*) and heights (*h*) of features printed under conditions in **Table S32**, and shown in **Figure S107** and **Figure S108**, # = feature number.

#	Time (min)	R1	*I*	R2	*I*	R3	*I*	R4	*I*	R5	*I*	R6	*I*	R7	*I*	R8	*I*	R9	*I*	Average Height (μm)
1	3.4	2.5	1.2	0.0	1.2	3.1	1.2	3.7	1.4	3.6	1.3	3.6	1.3	4.3	1.6	3.6	1.6	3.8	1.5	3.1
2	3.9	1.6	1.3	0.1	1.3	3.1	1.3	3.1	1.5	3.7	1.5	2.9	1.5	3.6	1.7	3.3	1.7	3.2	1.7	2.8
3	4.4	1.3	1.4	1.3	1.4	1.3	1.7	2.4	1.6	2.3	1.7	1.3	1.7	2.2	1.9	2.3	1.9	2.8	1.8	1.9
4	4.9	0.8	2.1	0.4	1.6	0.2	2.0	1.3	2.3	1.3	2.1	0.3	1.8	2.2	2.1	2.4	2.1	1.7	2.0	1.2
5	5.4	7.5	2.7	6.2	2.2	5.7	2.1	8.7	2.9	6.6	2.6	6.5	2.4	10.2	2.4	7.7	2.5	7.8	2.4	7.4
6	5.9	4.7	3.8	5.0	3.4	5.0	3.1	5.2	4.1	5.8	2.3	5.6	2.8	6.0	2.8	6.9	2.4	7.0	2.3	5.7
7	6.4	4.1	4.6	4.3	4.2	4.6	4.1	4.7	4.9	4.9	2.6	5.2	2.4	5.1	2.9	5.8	2.8	5.7	2.5	4.9
8	6.9	4.2	5.4	3.6	4.0	5.4	4.9	4.1	5.5	4.3	1.5	4.3	2.3	4.6	2.5	4.9	1.9	4.7	1.6	4.5
9	7.4	29.4	5.0	10.8	5.1	16.1	5.1	18.9	4.2	10.9	2.7	10.5	3.6	13.1	2.9	13.8	2.7	12.2	2.9	15.1
10	7.9	27.9	5.5	12.9	5.6	12.0	5.5	2.0	4.9	7.7	2.8	8.9	4.7	10.8	3.0	10.8	3.2	9.0	2.8	11.3
11	8.4	7.8	5.4	7.0	5.5	16.1	5.6	10.2	5.2	7.9	3.2	7.9	3.7	9.4	4.3	9.2	3.5	10.0	2.9	9.5
12	8.9	6.2	5.5	6.4	5.8	6.6	5.6	7.4	5.2	7.3	4.0	8.3	2.9	8.2	3.9	8.3	3.5	10.0	3.1	7.6
13	9.4	16.9	4.0	12.4	6.0	12.5	6.9	13.7	4.8	18.1	3.3	14.0	4.0	12.7	3.4	17.2	1.9	10.8	3.5	14.3
14	9.9	17.0	5.7	11.8	4.4	11.2	6.4	12.5	3.9	N/A	3.3	11.7	5.1	36.7	3.9	13.8	1.9	17.5	3.3	16.5
15	10.4	15.8	4.7	9.7	6.2	10.7	4.2	13.0	5.3	15.6	4.2	12.0	4.3	13.6	3.6	12.6	1.8	13.1	3.2	12.9
16	10.9	17.5	2.9	9.2	6.3	4.6	5.7	15.2	4.9	12.6	4.2	11.6	3.4	12.0	3.9	11.5	1.8	12.7	3.4	11.9

Table S34. Binding conditions for surfaces printed under conditions shown in **Table S13**.

TPO (mM)	PETT (mM)	EGDMA (mM)	Intensity (mW/mm2)	[**SCR043**] (µM)	[**α-Man-FL**] (M)
1	100	1300	2.53	12.5	$10^{-3.5}$

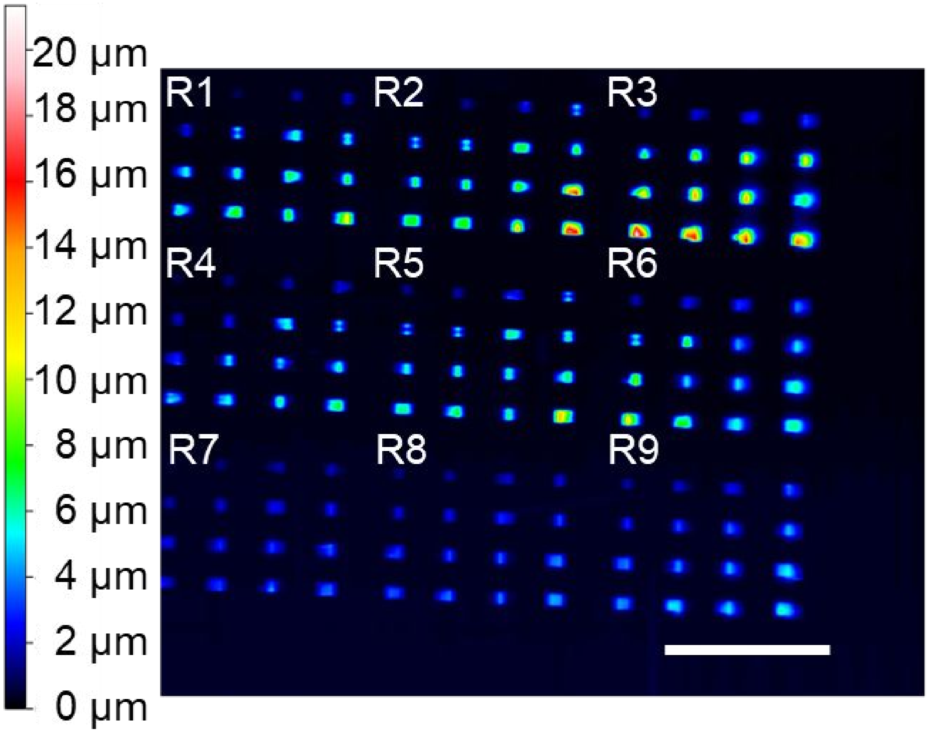

Figure S109. Profilometry image of patterns printed using [TPO] = 100 mM, [EGDMA] = 1300 mM. The pattern printed is 9 repeats (R1-R9) of 16 different time points. The scale bar is 200 µm.

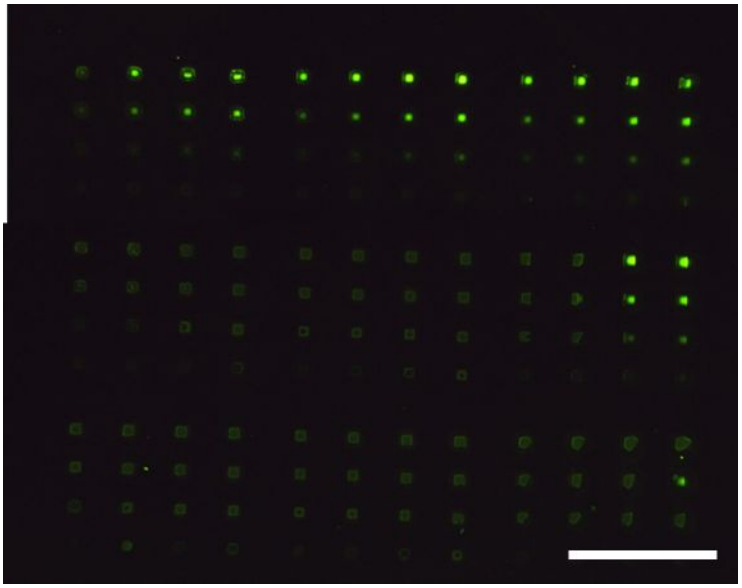

Figure S110. Fluorescence image of patterns printed using [TPO] = 100 mM, [EGDMA] = 1300 mM. Incubation was performed for 1 hour. The pattern printed is 9 repeats (R1-R9) of 16 different time points. The scale bar is 200 µm.

Table S35. Fluorescence intensity (*I*) and heights (*h*) of features printed under conditions in **Table S34**, and shown in **Figure S109** and **Figure S110**, # = feature number.

#	Time (min)	R1	*I*	R2	*I*	R3	*I*	R4	*I*	R5	*I*	R6	*I*	R7	*I*	R8	*I*	R9	*I*	Average Height (µm)
1	3.4	N/A	1.0	0.7	1.2	1.8	1.4	0.5	1.0	1.3	1.2	1.6	1.4	0.5	1.0	1.1	1.1	1.2	1.1	1.1
2	3.9	0.9	1.2	1.5	1.4	2.1	1.4	1.1	1.0	1.6	1.2	2.5	1.4	0.9	1.1	1.3	1.1	1.4	1.1	1.5
3	4.4	1.6	1.1	2.5	1.3	2.6	1.5	1.7	1.0	2.6	1.2	2.8	1.5	1.2	1.1	1.9	1.1	1.3	1.1	2.0
4	4.9	2.1	1.1	3.6	1.4	3.0	1.6	2.4	1.1	4.0	1.4	3.0	1.6	1.5	1.1	2.0	1.1	1.7	1.1	2.6
5	5.4	2.2	1.2	4.6	1.4	4.7	1.7	1.7	1.1	4.5	1.3	5.7	1.4	1.3	1.1	1.4	1.1	1.5	1.1	3.1
6	5.9	3.9	1.3	6.0	1.5	7.1	1.7	2.3	1.1	5.4	1.3	6.8	1.5	1.7	1.1	1.7	1.1	1.8	1.2	4.1
7	6.4	5.7	1.3	6.7	1.5	8.3	1.7	5.6	1.3	6.3	1.4	4.2	1.5	1.7	1.1	2.2	1.1	1.8	1.2	4.7
8	6.9	5.3	1.3	6.5	1.5	9.0	1.8	5.3	1.3	6.2	1.4	5.1	1.5	1.8	1.2	1.6	1.3	2.1	1.4	4.8
9	7.4	5.1	1.3	7.0	1.5	9.8	1.7	3.4	1.2	5.6	1.4	8.8	1.5	2.3	1.2	3.0	1.3	3.8	1.4	5.4
10	7.9	6.4	1.3	7.0	1.5	12.8	1.7	5.5	1.3	6.1	1.4	5.1	1.6	2.8	1.4	3.2	1.4	4.1	1.6	5.9
11	8.4	7.0	1.3	7.8	1.5	12.2	1.7	5.1	1.3	6.2	1.4	5.1	1.8	3.1	1.6	3.6	1.7	4.6	1.9	6.1
12	8.9	7.1	1.3	10.7	1.6	6.4	1.8	5.4	1.3	7.0	1.4	5.9	2.0	3.3	2.0	3.9	2.1	5.3	2.1	6.1
13	9.4	5.7	1.3	7.3	1.5	19.6	1.7	4.9	1.3	6.2	1.4	12.0	1.6	1.8	1.3	3.2	2.1	4.1	2.1	7.2
14	9.9	8.2	1.3	7.6	1.6	15.0	1.8	5.6	1.4	7.1	1.5	7.5	1.6	3.0	2.0	3.6	2.4	4.9	2.4	7.0
15	10.4	7.5	1.3	11.6	1.6	14.8	1.9	6.1	1.3	6.5	1.4	5.8	2.3	3.3	2.0	3.9	2.6	5.0	2.5	7.2
16	10.9	11.0	1.3	15.7	1.6	13.8	1.9	7.1	1.4	12.0	1.5	5.9	2.4	3.5	2.6	4.3	2.9	5.9	2.2	8.8

Table S36. Binding conditions for surfaces printed under conditions shown in **Table S13**.

TPO (mM)	PETT (mM)	EGDMA (mM)	Intensity (mW/mm²)	[**SCR043**] (μM)	[α-**Man-FL**] (M)
1	100	1300	2.53	0	$10^{-3.5}$

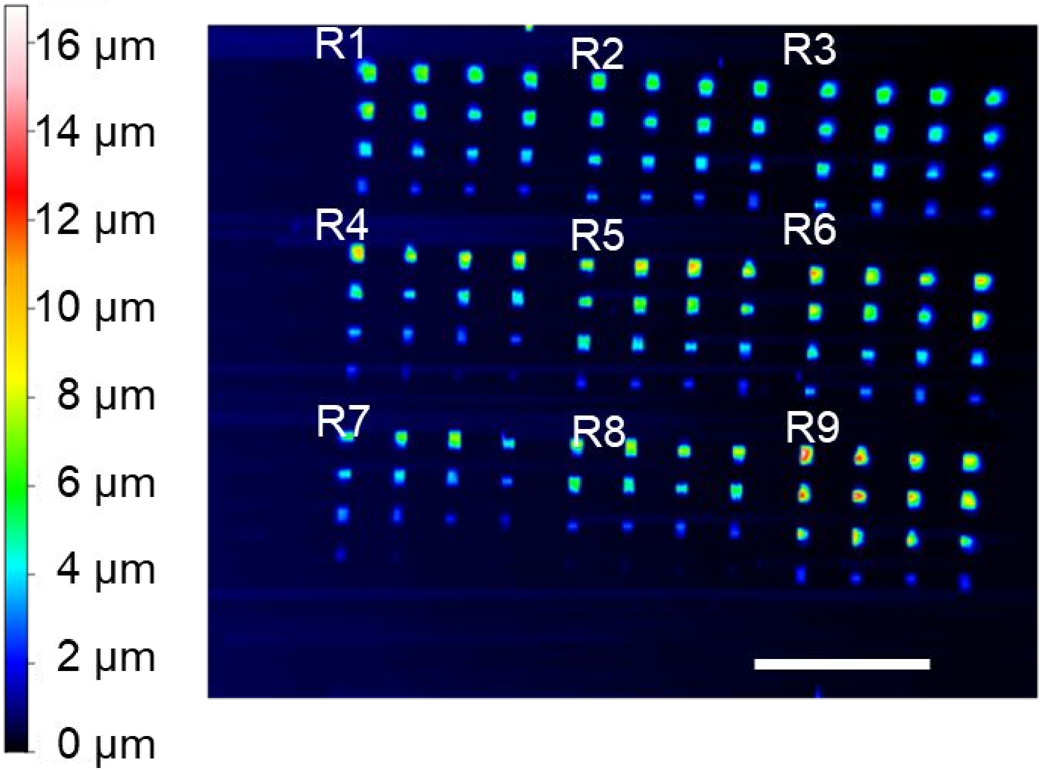

Figure S111. Profilometry image of patterns printed using [TPO] = 100 mM, [EGDMA] = 1300 mM. The pattern printed is 9 repeats (R1-R9) of 16 different time points. The scale bar is 200 μm.

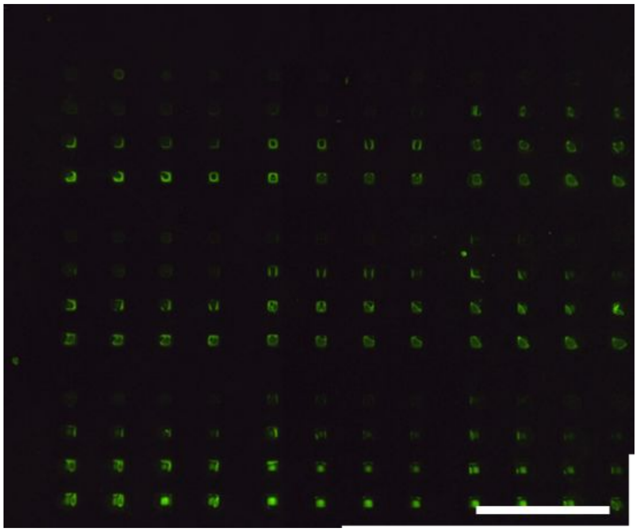

Figure S112. Fluorescence image of patterns printed using [TPO] = 100 mM, [EGDMA] = 1300 mM. Incubation was performed for 1 hour. The pattern printed is 9 repeats (R1-R9) of 16 different time points. The scale bar is 200 μm.

Table S37. Fluorescence intensity (*I*) and heights (*h*) of features printed under conditions in **Table S36**, and shown in **Figure S111** and **Figure S112**, # = feature number.

#	Time (min)	R1	*I*	R2	*I*	R3	*I*	R4	*I*	R5	*I*	R6	*I*	R7	*I*	R8	*I*	R9	*I*	Average Height (μm)
1	3.4	N/A	1.6	N/A	1.1	N/A	1.1	N/A	1.1	N/A	1.1	1.7	1.1	3.1	1.1	N/A	1.0	N/A	1.0	2.4
2	3.9	N/A	1.5	N/A	1.4	0.4	1.1	N/A	1.1	0.7	1.0	0.4	1.1	3.0	1.1	2.6	1.0	2.4	1.1	1.6
3	4.4	2.0	1.5	0.9	1.6	1.6	1.2	1.9	1.1	2.7	1.2	0.4	1.1	2.6	1.1	4.6	1.1	10.5	1.1	3.0
4	4.9	4.3	1.5	N/A	1.0	0.3	1.1	2.3	1.2	3.4	1.4	4.0	1.1	4.0	1.2	4.7	1.2	4.3	1.1	3.4
5	5.4	4.1	1.3	3.6	1.1	3.6	1.1	3.8	1.2	4.9	1.3	5.0	1.1	4.5	1.1	6.0	1.1	5.3	1.2	4.5
6	5.9	5.3	1.3	3.9	1.1	4.7	1.1	4.4	1.2	3.8	1.2	5.3	1.1	6.8	1.2	6.7	1.1	6.5	1.3	5.2
7	6.4	5.0	1.3	4.4	1.1	4.4	1.1	4.1	1.3	5.1	1.3	5.8	1.1	6.3	1.3	6.5	1.3	7.3	1.3	5.4
8	6.9	6.3	1.4	5.5	1.1	4.5	1.2	4.5	1.5	5.5	1.5	6.4	1.2	7.7	1.4	6.6	1.6	6.5	1.4	5.9
9	7.4	7.0	1.5	6.4	1.5	6.1	1.2	8.5	1.9	8.4	1.3	7.6	1.3	6.7	1.3	7.1	1.3	7.4	1.6	7.2
10	7.9	6.6	1.5	8.7	1.2	6.2	1.2	6.8	1.5	6.8	1.4	6.7	1.3	8.0	1.4	9.3	1.4	7.8	1.8	7.4
11	8.4	7.2	1.4	8.3	1.1	6.2	1.3	6.7	1.6	6.9	1.7	7.0	1.4	8.6	1.5	8.0	1.6	8.1	1.8	7.4
12	8.9	6.6	1.4	8.2	1.4	6.5	1.5	7.0	1.5	7.1	1.8	7.3	1.6	9.7	1.7	8.1	1.9	8.5	1.8	7.7
13	9.4	9.7	1.6	8.7	1.5	10.0	1.6	6.9	1.6	7.0	1.4	8.0	1.7	8.5	1.6	9.4	1.6	9.7	2.0	8.7
14	9.9	9.3	1.7	9.4	1.5	9.0	1.9	8.6	1.7	9.0	1.5	8.4	1.8	8.6	1.9	10.3	1.8	11.2	2.2	9.3
15	10.4	9.9	1.5	10.5	1.6	8.9	1.8	7.5	1.7	8.4	1.6	8.7	1.8	9.1	1.9	10.4	2.0	11.1	2.2	9.4
16	10.9		1.5	10.3		10.3	2.0	9.5	1.6	8.0	1.7	11.4	1.8	10.2	1.9	9.9	2.2	10.9	2.1	10.1

Table S38. Binding conditions for surfaces printed under conditions shown in **Table S13**.

TPO (mM)	PETT (mM)	EGDMA (mM)	Intensity (mW/mm^2)	[**SCR043**] (μM)	[α-**Man-FL**] (M)
1	100	1300	2.53	500	10^{-4}

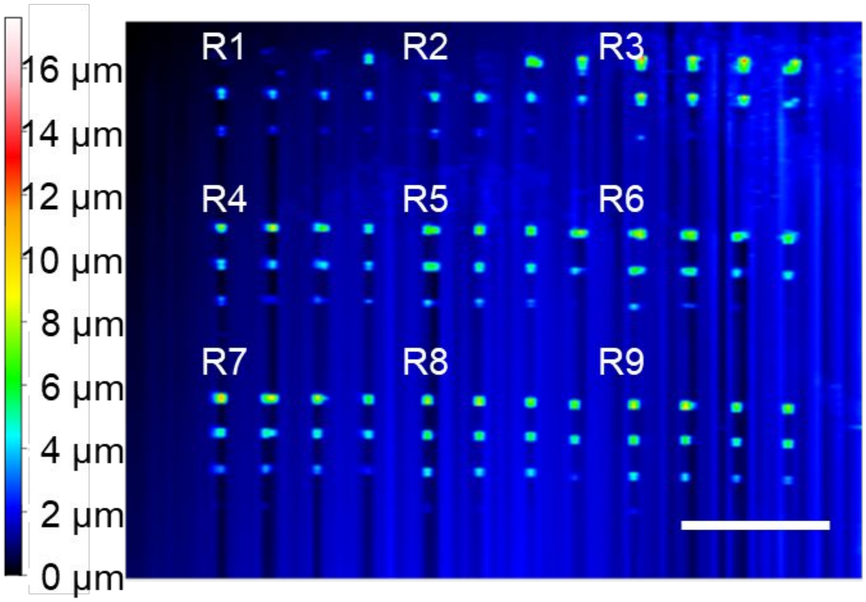

Figure S113. Profilometry image of patterns printed using [TPO] = 100 mM, [EGDMA] = 1300 mM. The pattern printed is 9 repeats (R1-R9) of 16 different time points. The scale bar is 200 μm.

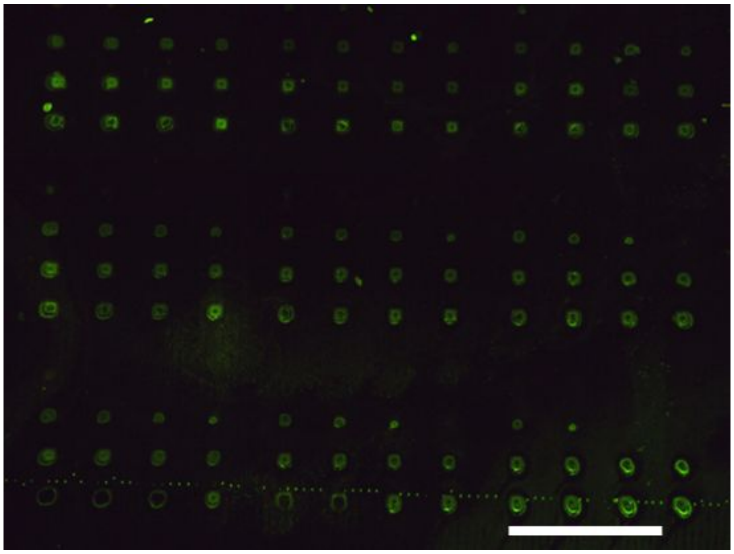

Figure S114. Fluorescence image of patterns printed using [TPO] = 100 mM, [EGDMA] = 1300 mM. Incubation was performed for 1 hour. The pattern printed is 9 repeats (R1-R9) of 16 different time points. The scale bar is 200 µm.

Table S39. Fluorescence intensity (*I*) and heights (*h*) of features printed under conditions in **Table S38**, and shown in **Figure S113** and **Figure S114**, # = feature number.

#	Time (min)	R1	*I*	R2	*I*	R3	*I*	R4	*I*	R5	*I*	R6	*I*	R7	*I*	R8	*I*	R9	*I*	Average Height (µm)
1	4.5	0.6	1.1	N/A	1.4	11.6	2.0	7.6	2.2	5.7	1.6	7.1	1.6	10.5	1.9	7.4	1.5	8.1	1.5	7.3
2	5	1.0	1.2	N/A	1.3	8.8	2.2	9.1	1.5	7.1	1.6	6.4	1.7	8.6	1.5	7.9	1.5	9.1	1.5	7.2
3	5.5	1.5	1.2	6.2	1.7	10.4	2.4	6.1	1.5	5.8	1.6	5.8	1.7	8.1	1.8	6.3	1.7	6.7	1.6	6.3
4	6	5.6	1.6	8.5	1.7	8.1	2.5	4.3	2.3	7.2	1.6	5.5	1.6	5.3	1.7	6.2	1.5	6.7	1.4	6.4
5	6.5	4.9	1.5	4.8	1.5	7.1	1.7	4.8	1.8	4.5	1.4	5.3	1.4	4.5	1.4	5.5	1.2	5.9	1.3	5.3
6	7	4.2	1.5	4.9	1.5	7.7	1.8	3.6	1.5	4.8	1.4	4.6	1.5	4.3	1.6	5.1	1.3	5.3	1.2	5.0
7	7.5	3.3	1.4	4.0	1.4	8.0	1.9	3.6	1.5	4.2	1.4	3.3	1.5	4.1	1.7	3.9	1.3	4.8	1.5	4.4
8	8	3.4	1.3	3.8	1.4	3.0	1.9	3.5	1.8	3.2	1.4	3.8	1.4	3.4	2.1	4.4	1.6	5.1	1.4	3.7
9	8.5	2.6	1.3	2.2	1.2	2.3	1.2	3.0	1.3	3.2	1.2	3.4	1.1	2.3	1.2	3.7	1.1	4.2	1.1	3.0
10	9	2.0	1.2	1.2	1.3	N/A	1.3	1.1	1.2	3.4	1.2	1.5	1.2	2.6	1.3	3.2	1.2	3.4	1.3	2.3
11	9.5	0.6	1.2	0.8	1.3	N/A	1.1	1.9	1.2	2.0	1.2	N/A	1.2	2.5	1.3	2.6	1.2	2.9	1.2	1.9
12	10	1.0	1.2	N/A	1.0	N/A	1.1	1.5	1.3	0.5	1.2	N/A	1.1	0.7	1.4	1.0	1.1	2.2	1.1	1.1
13	10.5	N/A	1.1	N/A	1.2	N/A	1.0	N/A	1.0	N/A	1.0	N/A	1.0	N/A	1.0	N/A	0.9	0.8	1.0	0.8
14	11	N/A	1.0	N/A	1.1	N/A	1.0	N/A	1.0	N/A	1.0	N/A	1.0	N/A	1.0	N/A	1.0	N/A	0.9	N/A
15	11.5	N/A	1.0	N/A	1.1	N/A	1.0	N/A	0.9	N/A	0.9	N/A	1.0	N/A	1.0	N/A	1.0	N/A	1.0	N/A
16	12	N/A	1.2	N/A	1.0	N/A	1.1	N/A	1.0	N/A	1.0	N/A	1.0	N/A	1.1	N/A	1.0	N/A	1.1	N/A

Table S40. Binding conditions for surfaces printed under conditions shown in **Table S13**.

TPO (mM)	PETT (mM)	EGDMA (mM)	Intensity (mW/mm^2)	[**SCR043**] (µM)	[α-**Man-FL**] (M)
1	100	1300	2.53	250	10^{-4}

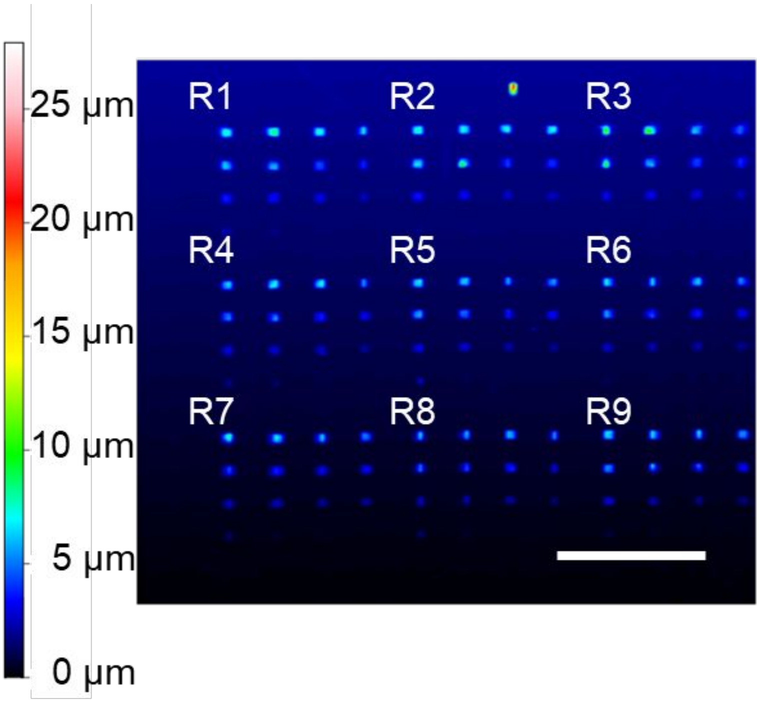

Figure S115. Profilometry image of patterns printed using [TPO] = 100 mM, [EGDMA] = 1300 mM. The pattern printed is 9 repeats (R1-R9) of 16 different time points. The scale bar is 200 µm.

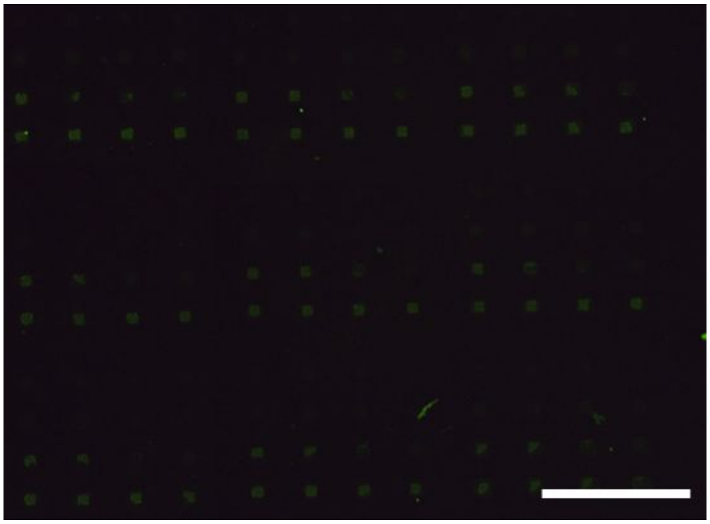

Figure S116. Fluorescence image of patterns printed using [TPO] = 100 mM, [EGDMA] = 1300 mM. Incubation was performed for 1 hour. The pattern printed is 9 repeats (R1-R9) of 16 different time points. The scale bar is 200 µm.

Table S41. Fluorescence intensity (*I*) and heights (*h*) of features printed under conditions in **Table S40**, and shown in **Figure S115** and **Figure S116**, # = feature number.

#	Time	R1	*I*	R2	*I*	R3	*I*	R4	*I*	R5	*I*	R6	*I*	R7	*I*	R8	*I*	R9	*I*	Average Height (µm)
1	4.5	N/A	1.0	N/A	1.0	N/A	1.0	N/A	1.0	N/A	1.0	N/A	1.0	N/A	1.0	N/A	1.0	N/A	0.9	N/A
2	5	N/A	1.0	N/A	1.0	0.2	1.0	N/A	1.0	N/A	1.0	N/A	1.0	N/A	1.0	N/A	1.0	N/A	0.9	0.2
3	5.5	0.2	1.0	0.4	1.0	0.5	1.0	N/A	1.0	0.3	1.0	0.8	1.0	N/A	1.0	0.4	1.0	0.5	0.9	0.4
4	6	0.6	1.0	1.1	1.0	1.1	1.0	0.3	1.0	2.0	1.0	1.0	1.0	0.4	1.0	0.6	1.0	1.0	0.9	0.9
5	6.5	1.4	1.1	1.3	1.0	1.4	1.0	1.2	1.0	1.2	1.0	1.2	1.0	1.2	1.0	1.2	1.1	1.3	1.0	1.3
6	7	1.7	1.0	1.6	1.0	1.8	1.0	1.5	1.1	1.5	1.0	2.1	1.0	1.6	1.0	1.5	1.0	1.5	1.0	1.6
7	7.5	1.9	1.1	1.9	1.0	2.0	1.0	1.6	1.1	1.8	1.0	1.9	1.0	1.7	1.0	1.7	1.0	1.8	1.0	1.8
8	8	2.4	1.0	2.2	1.0	2.2	1.0	1.9	1.0	2.1	1.0	2.0	1.0	1.9	1.0	2.0	1.0	2.2	1.0	2.1
9	8.5	2.7	1.3	2.5	1.2	2.9	1.2	2.2	1.1	2.2	1.1	2.2	1.1	2.3	1.1	2.2	1.0	2.2	1.0	2.4
10	9	7.3	1.2	3.2	1.2	2.7	1.1	2.5	1.1	2.9	1.2	2.5	1.1	2.4	1.1	2.6	1.1	2.6	1.0	3.2
11	9.5	0.0	1.2	4.0	1.2	3.3	1.1	3.2	1.1	3.9	1.1	4.6	1.1	4.3	1.1	8.7	1.1	4.2	1.1	4.0
12	10	4.7	1.1	4.5	1.1	3.9	1.0	4.7	1.1	4.6	1.1	4.2	1.1	8.7	1.1	5.0	1.1	4.8	1.1	5.0
13	10.5	4.6	1.3	4.9	1.2	4.6	1.3	4.5	1.2	4.6	1.2	4.3	1.2	2.9	1.1	5.8	1.2	5.0	1.2	4.6
14	11	6.0	1.3	5.4	1.2	5.8	1.3	5.2	1.2	4.6	1.2	6.0	1.2	4.5	1.2	5.9	1.2	5.3	1.2	5.4
15	11.5	5.9	1.2	5.2	1.3	6.2	1.3	5.2	1.2	5.1	1.2	5.7	1.2	8.9	1.2	5.2	1.2	6.9	1.2	6.0
16	12	6.0	1.3	5.7	1.3	7.7	1.3	6.0	1.2	5.6	1.2	5.4	1.2	10.0	1.2	5.7	1.2	5.7	1.2	6.4

Table S42. Binding conditions for surfaces printed under conditions shown in **Table S13**.

TPO (mM)	PETT (mM)	EGDMA (mM)	Intensity (mW/mm^2)	[**SCR043**] (µM)	[α-**Man-FL**] (M)
1	100	1300	2.53	100	10^{-4}

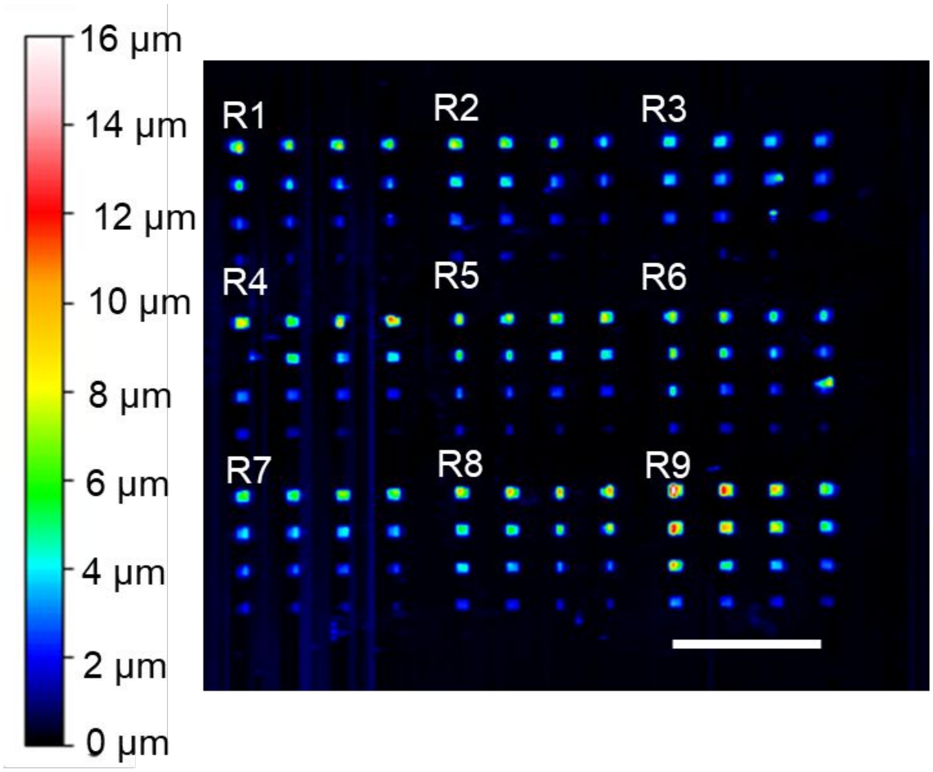

Figure S117. Profilometry image of patterns printed using [TPO] = 100 mM, [EGDMA] = 1300 mM. The pattern printed is 9 repeats (R1-R9) of 16 different time points. The scale bar is 200 µm.

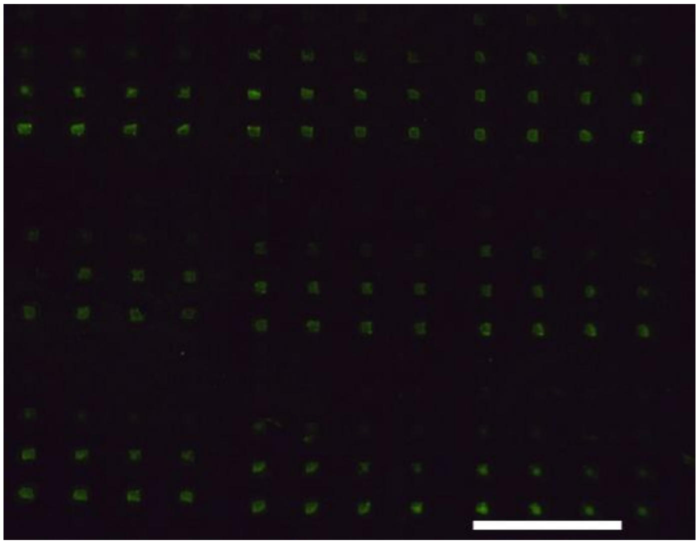

Figure S118. Fluorescence image of patterns printed using [TPO] = 100 mM, [EGDMA] = 1300 mM. Incubation was performed for 1 hour. The pattern printed is 9 repeats (R1-R9) of 16 different time points. The scale bar is 200 µm.

Table S43. Fluorescence intensity (*I*) and heights (*h*) of features printed under conditions in **Table S42**, and shown in **Figure S117** and **Figure S118**, # = feature number.

#	Time (min)	R1	*I*	R2	*I*	R3	*I*	R4	*I*	R5	*I*	R6	*I*	R7	*I*	R8	*I*	R9	*I*	Average Height (µm)
1	3.5	1.6	1.0	1.4	1.0	1.0	1.0	1.7	1.0	1.8	1.1	2.0	1.1	2.0	1.0	2.1	1.1	3.4	1.1	1.9
2	4	1.2	1.0	2.3	1.0	1.3	1.0	1.3	1.0	1.6	1.1	1.5	1.1	0.6	1.1	2.0	1.2	2.6	1.1	1.6
3	4.5	0.9	1.0	1.0	1.0	0.9	1.0	1.1	1.0	1.1	1.1	1.3	1.1	1.4	1.1	1.8	1.2	2.0	1.1	1.3
4	5	0.4	1.1	0.5	1.0	0.2	1.0	0.5	1.1	0.8	1.1	1.2	1.1	1.3	1.2	1.5	1.2	1.4	1.2	0.9
5	5.5	2.8	1.0	3.1	1.0	2.8	1.0	3.0	1.1	3.5	1.1	5.0	1.3	3.1	1.1	4.6	1.3	10.9	1.4	4.3
6	6	2.4	1.1	2.3	1.1	2.6	1.1	2.6	1.1	2.7	1.2	2.8	1.3	3.0	1.3	3.8	1.3	5.2	1.4	3.1
7	6.5	2.0	1.1	2.1	1.2	7.6	1.1	2.3	1.2	2.4	1.2	2.4	1.3	2.7	1.3	3.5	1.4	3.8	1.4	3.2
8	7	3.0	1.1	1.9	1.2	1.7	1.2	2.0	1.3	2.1	1.3	8.6	1.1	2.6	1.2	3.0	1.6	2.9	1.4	3.1
9	7.5	5.9	1.1	4.8	1.2	4.3	1.3	3.2	1.2	7.0	1.3	7.5	1.3	4.1	1.4	5.5	1.4	13.1	1.5	6.1
10	8	4.9	1.2	5.0	1.2	3.5	1.3	6.2	1.3	5.4	1.4	6.1	1.5	3.9	1.4	6.0	1.4	10.5	1.5	5.7
11	8.5	4.1	1.3	3.5	1.4	7.8	1.4	4.3	1.3	4.3	1.4	4.2	1.5	4.4	1.4	7.1	1.5	9.2	1.8	5.4
12	9	3.8	1.4	3.0	1.4	5.1	1.5	4.8	1.3	5.0	1.5	3.1	1.3	3.7	1.4	9.2	1.6	5.7	1.8	4.8
13	9.5	9.6	1.3	8.4	1.5	5.1	1.5	9.0	1.5	8.5	1.3	8.0	1.3	6.5	1.7	10.4	1.4	15.9	N/A	9.0
14	10	7.6	1.4	7.7	1.5	5.0	1.5	6.6	1.5	8.4	1.4	7.8	1.4	6.1	1.5	10.7	1.4	12.4	N/A	8.0
15	10.5	8.2	1.5	7.0	1.5	3.8	1.5	9.8	1.5	7.7	1.4	5.9	1.4	7.2	1.5	11.2	1.4	10.8	N/A	7.9
16	11	7.0	1.7	4.4	1.5	3.3	1.5	11.9	1.5	9.3	1.4	5.1	1.4	7.6	1.5	10.9	1.5	7.2	N/A	7.4

Table S44. Binding conditions for surfaces printed under conditions shown in **Table S13**.

TPO (mM)	PETT (mM)	EGDMA (mM)	Intensity (mW/mm^2)	[**SCR043**] (μM)	[**α-Man-FL**] (M)
1	100	1300	2.53	50.0	10^{-4}

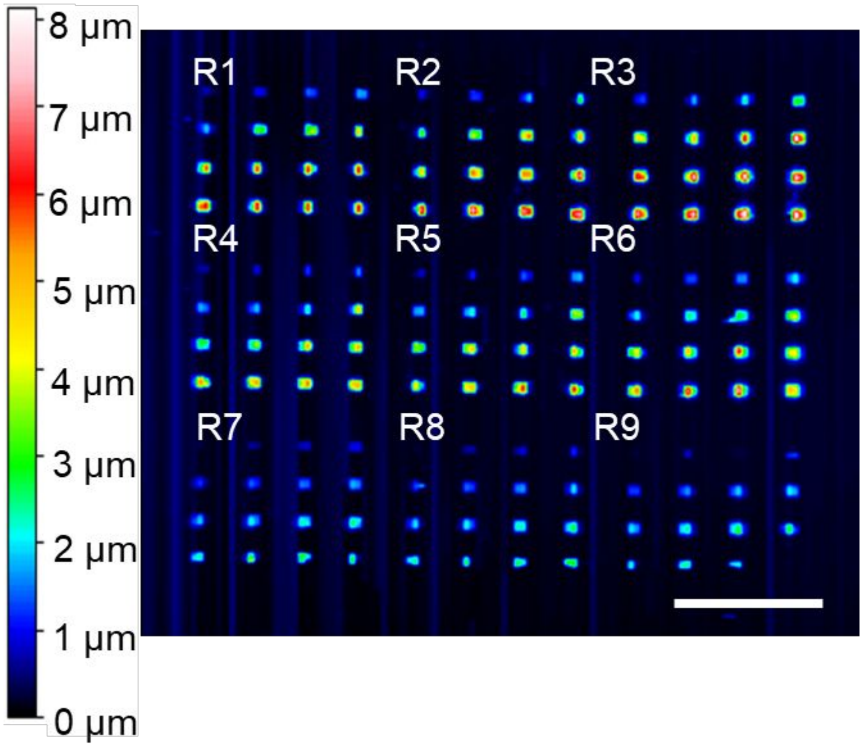

Figure S119. Profilometry image of patterns printed using [TPO] = 100 mM, [EGDMA] = 1300 mM. The pattern printed is 9 repeats (R1-R9) of 16 different time points. The scale bar is 200 μm.

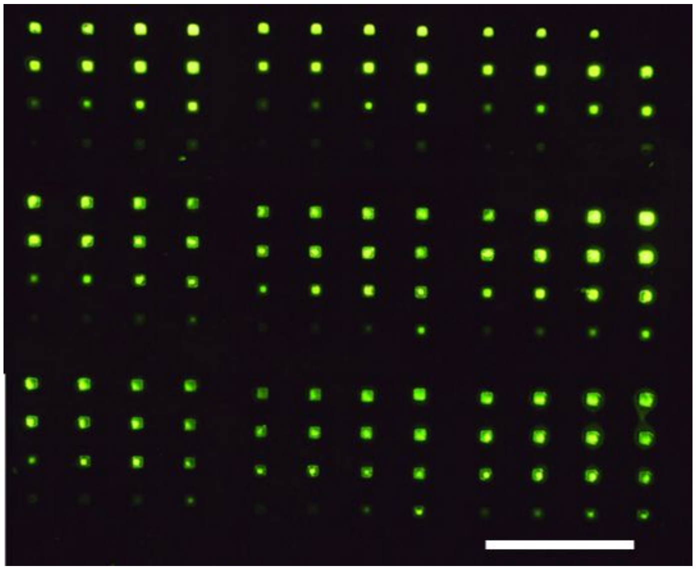

Figure S120. Fluorescence image of patterns printed using [TPO] = 100 mM, [EGDMA] = 1300 mM. Incubation was performed for 1 hour. The pattern printed is 9 repeats (R1-R9) of 16 different time points. The scale bar is 200 µm.

Table S45. Fluorescence intensity (*I*) and heights (*h*) of features printed under conditions in **Table S44**, and shown in **Figure S119** and **Figure S120**, # = feature number.

#	Time (min)	R1	*I*	R2	*I*	R3	*I*	R4	*I*	R5	*I*	R6	*I*	R7	*I*	R8	*I*	R9	*I*	Average Height (µm)
1	3.4	1.9	1.0	1.6	1.0	1.7	1.0	2.3	1.1	2.7	1.0	4.0	1.1	2.9	1.1	4.9	1.1	10.0	1.1	3.5
2	3.9	1.4	1.1	1.4	1.1	1.5	1.1	1.8	1.2	2.5	1.1	2.0	1.1	2.3	1.3	4.9	1.1	3.3	1.1	2.4
3	4.4	1.1	1.1	1.2	1.1	1.2	1.1	3.7	1.6	1.6	1.2	1.5	1.2	1.8	1.7	2.7	1.2	1.9	1.1	1.9
4	4.9	0.8	1.3	1.1	1.2	1.0	1.3	1.4	2.4	1.2	1.9	1.2	1.3	1.5	1.8	2.5	2.0	1.6	1.3	1.4
5	5.4	3.2	1.4	3.9	1.3	3.3	1.4	3.8	2.8	30.4	1.9	5.5	1.8	11.1	2.7	28.7	2.0	39.3	1.6	14.3
6	5.9	3.5	2.2	2.8	1.4	2.8	1.9	3.9	3.7	4.7	3.0	4.2	2.6	6.8	3.3	15.7	2.5	11.2	2.4	6.2
7	6.4	2.5	2.9	2.4	2.2	2.3	2.7	3.9	4.6	5.7	3.6	3.8	3.1	4.8	3.5	6.2	2.3	15.2	3.1	5.2
8	6.9	2.0	3.5	2.0	3.3	2.0	3.5	2.7	4.7	3.4	3.2	3.1	3.0	4.3	2.7	6.6	2.6	3.9	2.0	3.3
9	7.4	5.6	3.6	5.5	3.5	4.3	3.7	6.2	4.3	6.8	3.2	9.1	4.6	7.5	3.7	7.9	2.3	7.2	3.5	6.7
10	7.9	5.6	4.4	7.0	3.8	4.8	4.2	6.7	5.2	5.9	3.8	6.9	4.4	7.3	3.8	10.0	2.8	11.8	2.9	7.3
11	8.4	6.1	5.0	4.7	4.4	3.2	4.9	5.4	6.0	6.6	4.4	5.1	3.9	9.7	4.6	10.1	3.0	9.5	3.2	6.7
12	8.9	5.0	4.4	4.1	5.0	3.0	5.2	5.4	6.9	7.9	3.3	5.0	4.0	28.0	3.4	11.0	3.5	8.3	2.3	8.6
13	9.4	6.1	3.2	6.2	4.0	5.1	3.8	6.4	3.2	8.7	3.1	8.2	4.9	10.2	3.4	31.3	1.9	16.4	3.6	11.0
14	9.9	6.0	3.1	8.2	4.0	14.8	3.7	6.2	4.9	8.2	3.7	6.2	4.7	11.9	4.1	31.6	2.6	14.9	3.6	12.0
15	10.4	6.6	2.6	6.4	4.2	4.7	4.8	6.2	6.4	8.3	4.0	7.6	4.4	11.4	4.3	32.4	3.2	7.2	2.9	10.1
16	10.9	5.6	1.0	6.9	3.9	3.9	5.0	5.8	7.7	5.8	4.0	6.4	3.2	33.6	4.5	19.5	3.5	7.7	2.6	10.6

Table S46. Binding conditions for surfaces printed under conditions shown in **Table S13**.

TPO (mM)	PETT (mM)	EGDMA (mM)	Intensity (mW/mm^2)	[**SCR043**] (µM)	[α-**Man-FL**] (M)
1	100	1300	2.53	12.5	10^{-4}

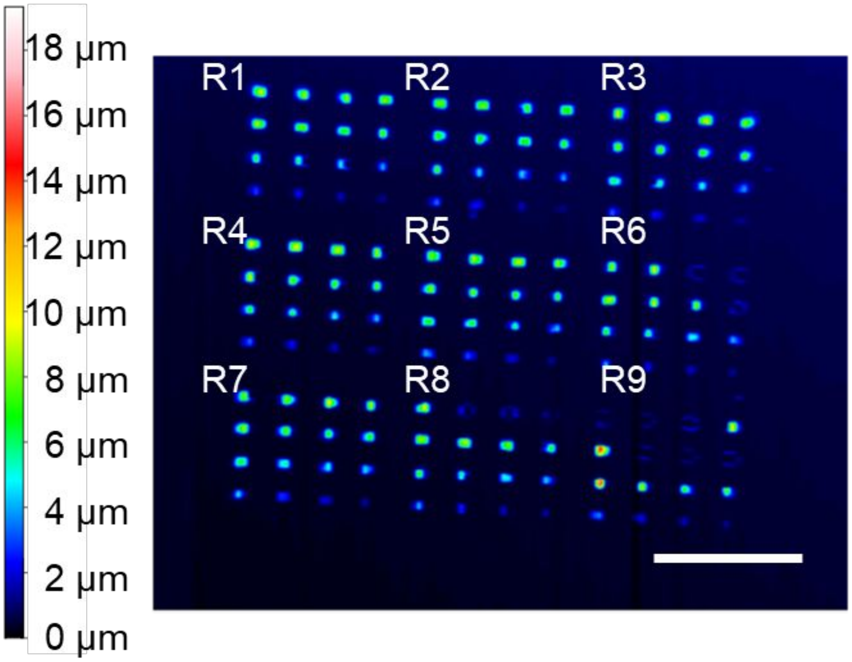

Figure S121. Profilometry image of patterns printed using [TPO] = 100 mM, [EGDMA] = 1300 mM. The pattern printed is 9 repeats (R1-R9) of 16 different time points. The scale bar is 200 µm.

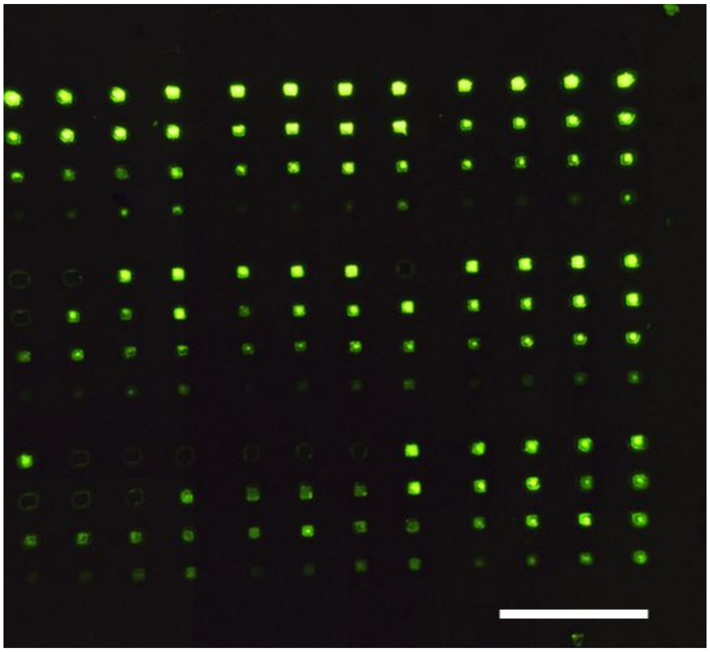

Figure S122. Fluorescence image of patterns printed using [TPO:100mM], [EGDMA 1300mM. Incubation was performed for 1 hour. The pattern printed is 9 repeats (R1-R9) of 16 different time points. The scale bar is 200 μm.

Table S47. Fluorescence intensity (*I*) and heights (*h*) of features printed under conditions in **Table S46**, and shown in **Figure S121** and **Figure S122**, # = feature number.

#	Time (min)	R1	*I*	R2	*I*	R3	*I*	R4	*I*	R5	*I*	R6	*I*	R7	*I*	R8	*I*	R9	*I*	Average Height (μm)
1	3.4	N/A	1.1	N/A	1.0	N/A	1.0	N/A	1.0	N/A	1.0	N/A	1.0	N/A	1.0	N/A	1.0	N/A	1.0	N/A
2	3.9	N/A	1.0	N/A	1.0	N/A	1.0	N/A	1.0	N/A	1.0	N/A	1.0	N/A	1.0	N/A	1.0	N/A	1.0	N/A
3	4.4	N/A	1.1	N/A	1.0	N/A	1.0	N/A	1.0	N/A	1.0	N/A	1.0	N/A	1.0	N/A	1.0	N/A	1.0	N/A
4	4.9	N/A	1.0	N/A	1.0	N/A	1.0	N/A	1.0	N/A	1.0	N/A	1.0	N/A	1.0	N/A	1.0	N/A	1.0	N/A
5	5.4	1.7	1.0	1.5	1.0	1.4	1.0	1.7	1.1	2.4	1.1	1.4	1.1	1.6	1.1	3.1	1.2	1.7	1.2	1.9
6	5.9	1.7	1.1	1.3	1.0	0.5	1.0	1.6	1.1	1.6	1.1	0.3	1.1	2.0	1.2	2.0	1.2	2.7	1.2	1.5
7	6.4	1.0	1.1	0.2	1.1	N/A	1.1	1.1	1.1	0.4	1.1	N/A	1.1	1.5	1.1	N/A	1.1	0.8	1.1	0.8
8	6.9	N/A	1.1	N/A	1.1	N/A	1.1	N/A	1.1	N/A	1.1	N/A	1.1	N/A	1.0	N/A	1.0	N/A	1.0	N/A
9	7.4	4.8	1.2	3.7	1.1	3.9	1.2	4.3	1.4	4.4	1.3	4.1	1.3	5.3	1.5	4.5	1.5	3.8	1.4	4.3
10	7.9	3.8	1.2	4.1	1.2	3.2	1.3	4.2	1.3	3.6	1.3	3.6	1.3	2.7	1.4	4.2	1.4	4.4	1.2	3.8
11	8.4	3.7	1.3	3.0	1.3	2.5	1.3	4.0	1.3	3.8	1.2	2.0	1.3	3.7	1.3	3.7	1.3	2.9	1.4	3.3
12	8.9	3.0	1.4	2.3	1.3	1.6	1.3	3.1	1.3	1.9	1.2	2.1	1.3	3.4	1.2	3.0	1.2	2.6	1.6	2.5
13	9.4	6.2	1.4	4.9	1.4	4.9	1.4	5.8	1.8	6.2	1.5	6.2	1.5	6.3	1.9	5.7	1.8	6.8	1.7	5.9
14	9.9	5.6	1.6	5.1	1.5	5.3	1.4	5.5	1.7	5.6	1.5	5.6	1.6	6.1	1.8	6.3	1.7	6.9	1.6	5.8
15	10.4	4.6	1.7	4.4	1.6	4.3	1.6	5.1	1.7	4.7	1.5	4.6	1.6	5.2	1.7	5.7	1.5	4.9	1.5	4.8
16	10.9	4.8	1.7	4.9	1.7	3.0	1.6	4.3	1.7	5.3	1.5	3.3	1.5	5.1	1.5	5.1	1.4	4.1	1.5	4.4

Table S48. Binding conditions for surfaces printed under conditions shown in **Table S13**.

TPO (mM)	PETT (mM)	EGDMA (mM)	Intensity (mW/mm²)	[**SCR043**] (µM)	[α-**Man-FL**] (M)
1	100	1300	2.53	0	10^{-4}

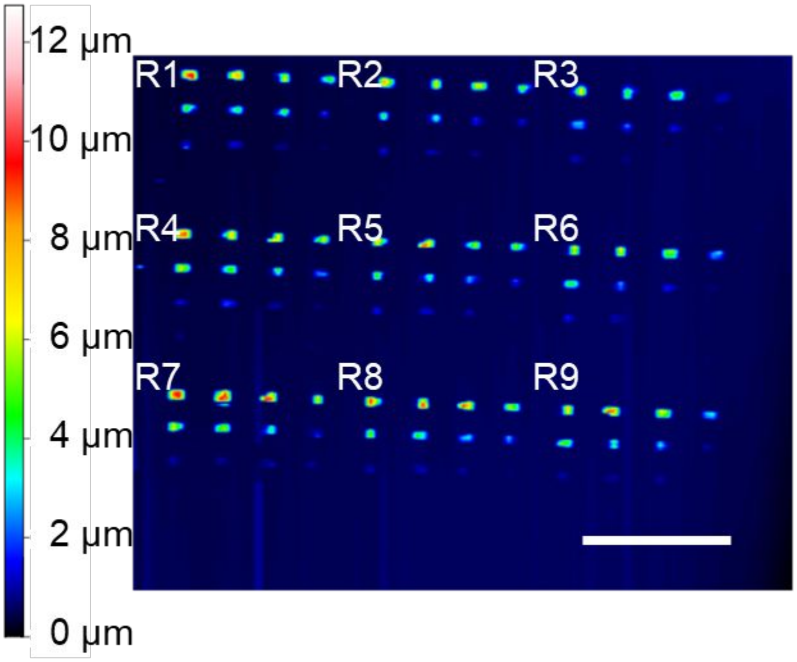

Figure S123. Profilometry image of patterns printed using [TPO] = 100 mM, [EGDMA] = 1300 mM. The pattern printed is 9 repeats (R1-R9) of 16 different time points. The scale bar is 200 µm.

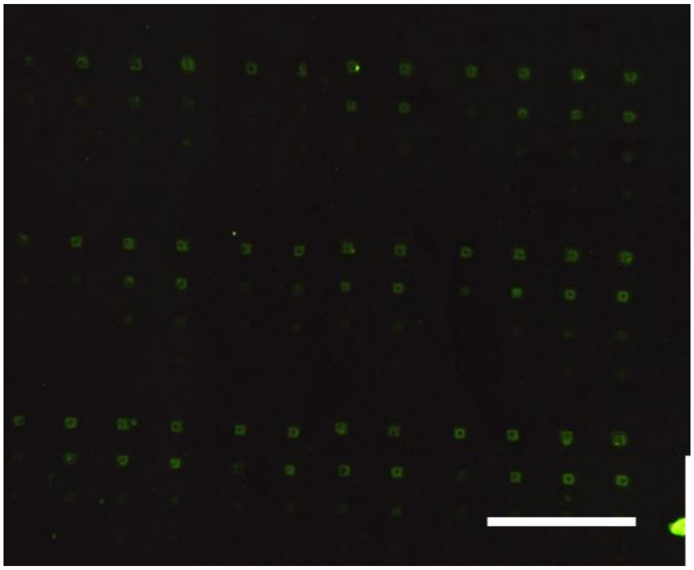

Figure S124. Fluorescence image of patterns printed using [TPO] = 100 mM, [EGDMA] = 1300 mM. Incubation was performed for 1 hour. The pattern printed is 9 repeats (R1-R9) of 16 different time points. The scale bar is 200 μm.

Table S49. Fluorescence intensity (*I*) and heights (*h*) of features printed under conditions in **Table S48**, and shown in **Figure S123** and **Figure S124**, # = feature number.

#	Time (min)	R1	*I*	R2	*I*	R3	*I*	R4	*I*	R5	*I*	R6	*I*	R7	*I*	R8	*I*	R9	*I*	Average Height (μm)
1	3.4	0.9	1.2	N/A	1.1	3.7	0.9	0.9	1.3	3.9	1.1	3.6	1.1	0.6	1.2	2.3	1.3	N/A	1.1	4.0
2	3.9	N/A	0.9	N/A	1.0	0.3	0.9	N/A	1.0	1.0	0.9	1.5	1.0	N/A	0.9	0.9	1.0	0.4	0.9	4.8
3	4.4	N/A	1.0	N/A	1.0	N/A	0.9	N/A	1.0	0.2	0.9	N/A	1.0	N/A	0.9	0.3	1.0	N/A	0.9	4.9
4	4.9	N/A	1.0	N/A	0.9	N/A	1.0	N/A	1.0	N/A	1.0	N/A	0.9	N/A	1.0	N/A	1.0	N/A	1.0	5.2
5	5.4	2.5	1.7	2.2	2.3	N/A	2.2	2.1	2.1	8.6	1.8	N/A	2.1	2.0	2.0	N/A	2.0	N/A	1.5	3.2
6	5.9	2.1	1.9	1.6	2.2	1.7	2.0	1.6	2.0	2.0	1.9	1.8	2.0	1.7	1.8	2.1	2.0	1.6	1.8	3.2
7	6.4	1.1	2.0	1.8	1.7	1.5	1.4	1.4	2.0	1.4	1.8	1.4	1.6	1.6	1.8	1.6	1.9	1.4	1.8	4.1
8	6.9	1.0	1.5	2.1	1.3	1.2	1.1	1.8	1.6	1.1	1.4	1.3	1.2	1.2	1.8	1.3	1.5	1.0	1.4	3.4
9	7.4	6.6	1.8	3.9	2.1	0.4	1.9	5.4	2.0	3.7	1.9	0.6	2.0	8.1	2.8	1.2	1.9	0.6	1.6	1.3
10	7.9	5.6	1.8	3.4	1.6	4.2	2.0	4.8	1.9	3.5	1.7	3.9	1.6	3.9	1.9	3.9	1.8	3.5	1.6	1.5
11	8.4	3.7	1.9	2.9	2.1	3.9	2.0	1.9	1.6	4.2	1.8	1.0	1.9	4.8	1.3	3.5	1.6	3.0	1.7	1.8
12	8.9	2.5	2.3	2.1	2.5	2.4	2.7	6.0	2.0	2.5	2.2	2.6	2.3	3.6	2.0	3.7	1.9	3.2	2.0	3.5
13	9.4	2.6	2.6	5.2	2.4	1.7	3.0	14.3	2.1	5.4	2.2	2.1	2.3	10.0	1.5	3.1	2.1	2.0	2.4	N/A
14	9.9	1.5	2.0	4.5	2.5	4.1	2.7	6.3	2.0	4.9	2.0	4.8	2.5	6.4	2.4	5.7	1.9	5.6	1.8	0.3
15	10.4	6.3	2.2	4.1	2.4	3.8	2.3	4.9	2.1	4.3	2.3	4.4	2.4	5.3	2.2	5.4	2.0	4.3	2.0	0.8
16	10.9	3.5	2.2	3.6	2.6	4.1	2.5	4.2	2.1	3.7	2.2	3.6	2.0	4.0	2.4	4.9	2.2	3.9	2.0	2.3

Table S50. Binding conditions for surfaces printed under conditions shown in **Table S13**.

TPO (mM)	PETT (mM)	EGDMA (mM)	Intensity (mW/mm^2)	[**SCR043**] (μM)	[α-**Man-FL**] (M)
1	100	1300	2.53	500	$10^{-4.5}$

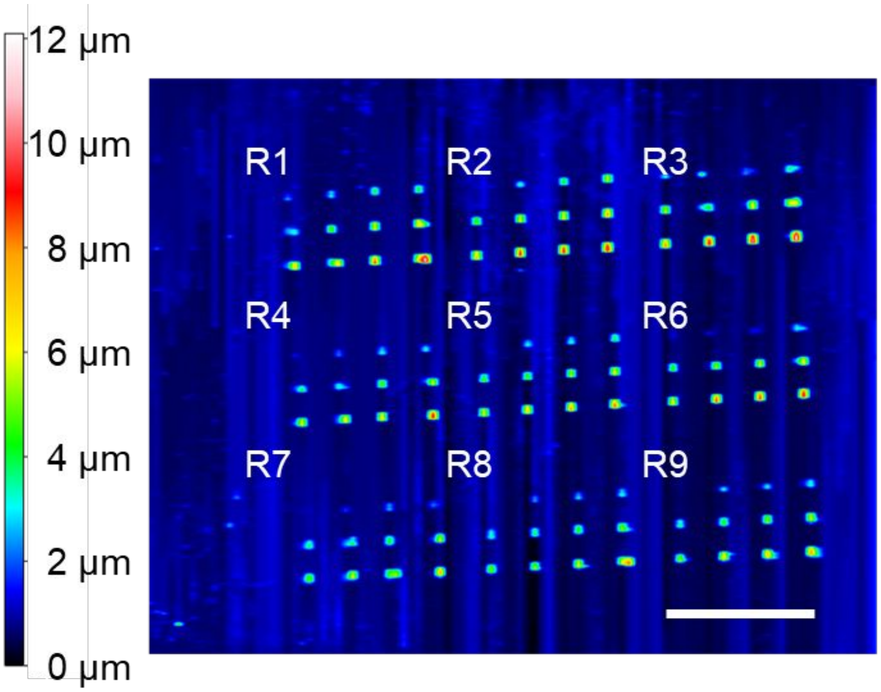

Figure S125. Profilometry image of patterns printed using [TPO] = 100 mM, [EGDMA] = 1300 mM. The pattern printed is 9 repeats (R1-R9) of 16 different time points. The scale bar is 200 μm.

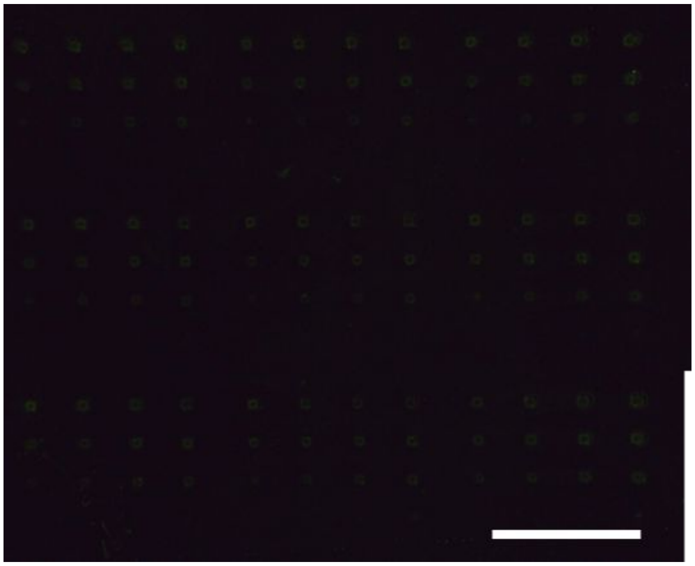

Figure S126. Fluorescence image of patterns printed using [TPO] = 100 mM, [EGDMA] = 1300 mM. Incubation was performed for 1 hour. The pattern printed is 9 repeats (R1-R9) of 16 different time points. The scale bar is 200 μm.

Table S51. Fluorescence intensity (*I*) and heights (*h*) of features printed under conditions in **Table S50**, and shown in **Figure S125** and **Figure S126**, # = feature number.

#	Time (min)	R1	*I*	R2	*I*	R3	*I*	R4	*I*	R5	*I*	R6	*I*	R7	*I*	R8	*I*	R9	*I*	Average Height (μm)
1	4.5	N/A	N/A	N/A	N/A	N/A	N/A	N/A	N/A	N/A	1.0	N/A	1.1	N/A	N/A	N/A	1.0	N/A	1.0	N/A
2	5	N/A	N/A	N/A	N/A	N/A	N/A	N/A	N/A	N/A	1.1	N/A	1.0	N/A	N/A	N/A	1.0	N/A	1.0	N/A
3	5.5	N/A	N/A	N/A	N/A	N/A	N/A	N/A	N/A	N/A	1.1	N/A	1.0	N/A	N/A	N/A	1.0	N/A	1.0	N/A
4	6.0	N/A	N/A	N/A	N/A	N/A	N/A	N/A	N/A	N/A	1.1	N/A	1.1	N/A	N/A	N/A	1.1	N/A	1.0	N/A
5	6.5	0.8	1.0	N/A	1.1	2.1	1.1	0.6	1.0	0.5	1.0	0.7	1.1	N/A	1.0	N/A	1.0	N/A	1.0	1.0
6	7	2.1	1.1	2.3	1.1	3.6	1.1	1.4	1.1	2.6	1.1	0.8	1.1	0.4	1.1	2.0	1.1	2.5	1.1	2.0
7	7.5	3.4	1.1	3.2	1.1	1.4	1.2	2.2	1.1	3.0	1.1	0.8	1.2	1.6	1.1	2.2	1.1	2.1	1.1	2.2
8	8	3.6	1.1	4.7	1.1	2.3	1.2	2.6	1.1	3.5	1.1	2.6	1.2	1.0	1.1	2.8	1.1	2.5	1.2	2.8
9	8.5	1.4	1.1	3.0	1.1	4.2	1.1	3.0	1.1	4.3	1.1	3.9	1.2	1.7	1.1	2.7	1.1	2.9	1.1	3.0
10	9	4.2	1.1	4.4	1.1	3.8	1.2	2.7	1.1	3.8	1.1	4.5	1.2	2.0	1.1	3.2	1.2	3.4	1.2	3.6
11	9.5	5.0	1.1	5.1	1.1	5.4	1.2	4.1	1.1	4.8	1.1	4.8	1.2	2.9	1.1	4.0	1.2	4.0	1.2	4.5
12	10	6.1	1.2	6.1	1.2	5.3	1.3	4.9	1.2	5.1	1.2	5.6	1.2	3.8	1.2	4.6	1.2	3.4	1.3	5.0
13	10.5	5.8	1.2	5.7	1.2	6.2	1.2	5.0	1.2	5.0	1.2	5.1	1.2	3.4	1.2	4.6	1.2	4.4	1.2	5.0
14	11	5.7	1.2	7.4	1.1	8.2	1.2	5.5	1.2	5.4	1.2	7.3	1.2	4.7	1.2	4.6	1.2	5.9	1.2	6.1
15	11.5	7.8	1.1	7.6	1.1	9.5	1.2	6.0	1.2	6.4	1.2	7.6	1.2	4.5	1.2	5.0	1.2	6.6	1.3	6.8
16	12	10.4	1.1	8.4	1.1	10.5	1.3	8.1	1.2	6.3	1.2	8.4	1.1	7.0	1.2	5.1	1.2	7.2	1.3	7.9

Table S52. Binding conditions for surfaces printed under conditions shown in **Table S13**.

TPO (mM)	PETT (mM)	EGDMA (mM)	Intensity (mW/mm^2)	[**SCR043**] (µM)	[α-**Man-FL**] (M)
1	100	1300	2.53	250	$10^{-4.5}$

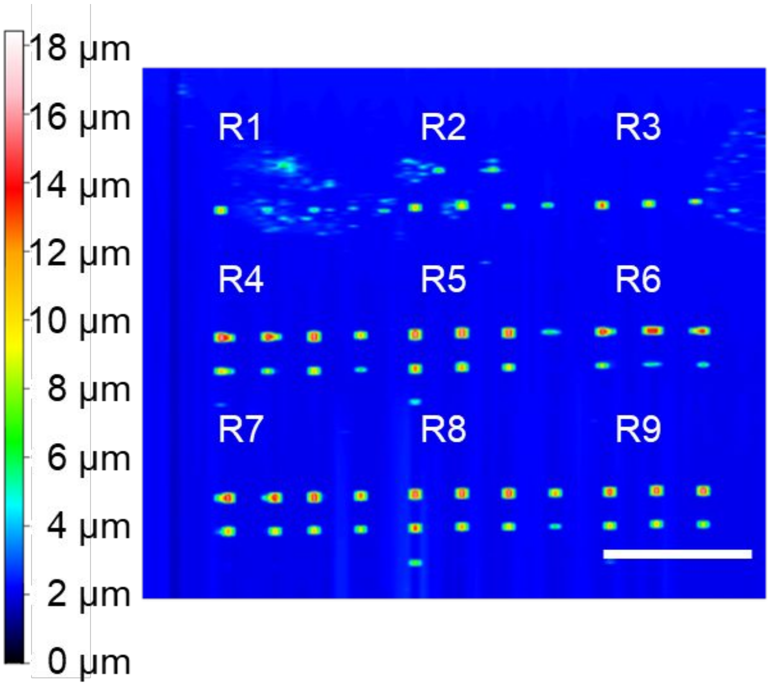

Figure S127. Profilometry image of patterns printed using [TPO] = 100 mM, [EGDMA] = 1300 mM. The pattern printed is 9 repeats (R1-R9) of 16 different time points. The scale bar is 200 µm.

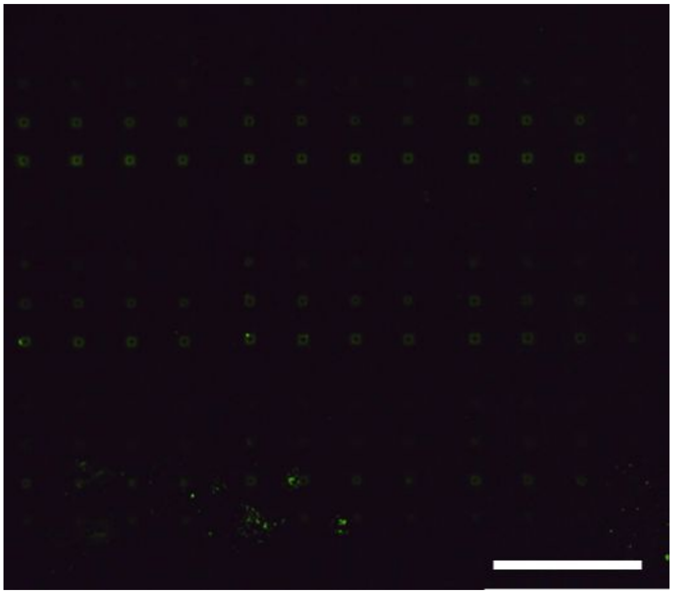

Figure S128. Fluorescence image of patterns printed using [TPO] = 100 mM, [EGDMA] = 1300 mM. Incubation was performed for 1 hour. The pattern printed is 9 repeats (R1-R9) of 16 different time points. The scale bar is 200 μm.

Table S53. Fluorescence intensity (*I*) and heights (*h*) of features printed under conditions in **Table S52**, and shown in **Figure S127** and **Figure S128**, # = feature number.

#	Time (min)	R1	*I*	R2	*I*	R3	*I*	R4	*I*	R5	*I*	R6	*I*	R7	*I*	R8	*I*	R9	*I*	Average Height (μm)
1	4.5	N/A	1.0	N/A	1.0	N/A	N/A	N/A	1.0	N/A	1.0	N/A	N/A	N/A	N/A	N/A	N/A	N/A	N/A	N/A
2	5	N/A	1.0	N/A	1.0	N/A	N/A	N/A	1.1	N/A	1.0	N/A	N/A	N/A	N/A	N/A	N/A	N/A	N/A	N/A
3	5.5	N/A	1.0	N/A	1.0	N/A	N/A	N/A	1.1	N/A	1.0	N/A	N/A	N/A	N/A	N/A	N/A	N/A	N/A	N/A
4	6	N/A	1.0	N/A	1.0	N/A	N/A	N/A	1.1	N/A	1.0	N/A	N/A	N/A	N/A	N/A	N/A	N/A	N/A	N/A
5	6.5	N/A	1.0	N/A	1.1	N/A	1.1	N/A	1.1	N/A	1.1	N/A	1.1	N/A	1.0	N/A	1.1	N/A	N/A	N/A
6	7	N/A	1.1	N/A	1.1	N/A	1.0	N/A	1.1	N/A	1.1	N/A	1.1	N/A	1.1	N/A	1.1	N/A	N/A	N/A
7	7.5	N/A	1.1	N/A	1.1	N/A	1.1	N/A	1.1	N/A	1.1	N/A	1.1	N/A	1.1	N/A	1.1	N/A	N/A	N/A
8	8	N/A	1.2	N/A	1.1	N/A	1.1	N/A	1.1	N/A	1.1	N/A	1.1	N/A	1.1	N/A	1.1	N/A	N/A	N/A
9	8.5	6.9	1.1	4.3	1.2	7.0	1.2	4.1	1.1	8.7	1.2	4.7	1.2	N/A	1.1	N/A	1.1	N/A	1.1	6.0
10	9	8.4	1.2	8.8	1.2	8.8	1.2	3.8	1.2	10.1	1.2	9.2	1.2	N/A	1.1	N/A	1.2	N/A	1.1	8.2
11	9.5	9.3	1.2	9.9	1.2	8.9	1.3	7.5	1.2	10.9	1.2	7.0	1.2	N/A	1.2	N/A	1.2	N/A	1.2	8.9
12	10	N/A	1.2	11.0	1.2	9.8	1.3	N/A	1.2	N/A	1.3	9.0	1.2	N/A	1.2	N/A	1.2	N/A	1.1	9.9
13	10.5	14.9	1.1	10.1	1.3	10.9	1.4	7.1	1.1	3.6	1.2	10.0	1.2	2.8	1.1	4.8	1.1	1.2	1.1	7.3
14	11	13.6	1.3	13.8	1.4	13.8	1.4	11.9	1.2	15.1	1.3	15.3	1.3	9.0	1.0	5.7	1.1	3.2	1.1	11.3
15	11.5	13.6	1.3	14.1	1.3	12.9	1.4	12.8	1.3	14.9	1.3	13.1	1.3	11.8	1.1	8.4	1.1	1.7	1.1	11.5
16	12	N/A	1.3	13.3	1.3	14.0	1.4	N/A	1.3	15.0	1.4	13.9	1.4	N/A	1.1	9.3	1.4	8.8	1.1	12.4

Table S54. Binding conditions for surfaces printed under conditions shown in **Table S13**.

TPO (mM)	PETT (mM)	EGDMA (mM)	Intensity (mW/mm^2)	[**SCR043**] (µM)	[α-**Man-FL**] (M)
1	100	1300	2.53	100	$10^{-4.5}$

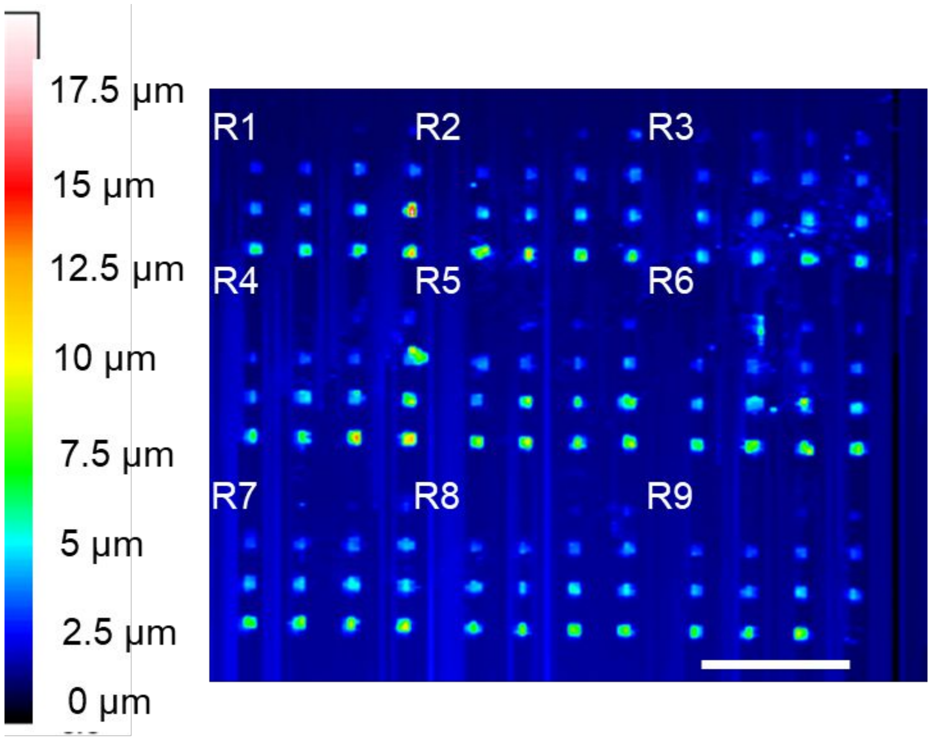

Figure S129. Profilometry image of patterns printed using [TPO] = 100 mM, [EGDMA] = 1300 mM. The pattern printed is 9 repeats (R1-R9) of 16 different time points. The scale bar is 200 µm.

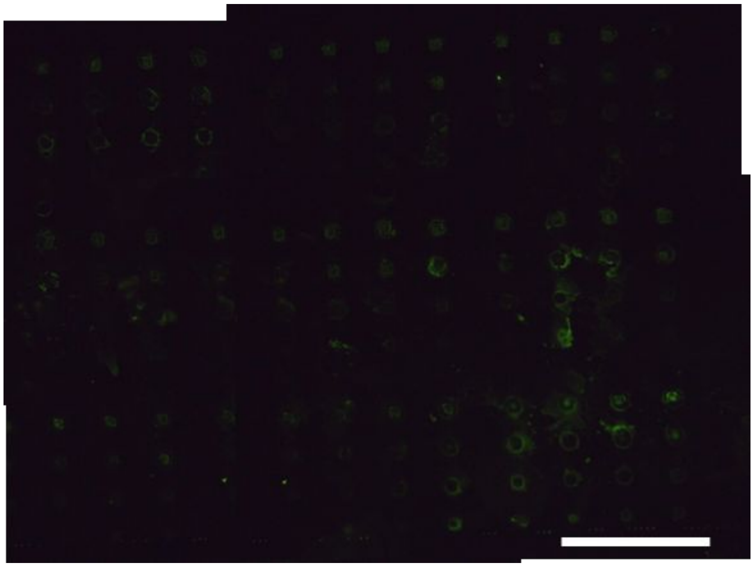

Figure S130. Fluorescence image of patterns printed using [TPO] = 100 mM, [EGDMA] = 1300 mM. Incubation was performed for 1 hour. The pattern printed is 9 repeats (R1-R9) of 16 different time points. The scale bar is 200 µm.

Table S55. Fluorescence intensity (*I*) and heights (*h*) of features printed under conditions in **Table S54**, and shown in **Figure S129** and **Figure S130**, # = feature number.

#	Time (min)	R1	*I*	R2	*I*	R3	*I*	R4	*I*	R5	*I*	R6	*I*	R7	*I*	R8	*I*	R9	*I*	Average Height (µm)
1	3.5	1.3	N/A	2.7	1.4	2.1	1.1	1.9	1.1	1.4	1.1	1.5	1.1	1.8	N/A	1.6	1.1	1.5	1.1	1.7
2	4	0.6	1.0	1.1	1.1	1.4	1.2	1.6	1.1	1.4	1.1	1.7	1.2	0.5	N/A	0.6	1.0	0.8	1.1	1.1
3	4.5	0.2	1.0	0.5	1.2	1.4	1.2	0.2	1.0	1.5	1.3	5.5	2.0	2.6	N/A	0.1	1.0	0.2	1.0	1.3
4	5	0.1	N/A	0.2	1.0	1.3	1.3	N/A	N/A	N/A	1.0	0.5	1.0	N/A	N/A	0.1	1.0	N/A	1.0	0.4
5	5.5	2.7	N/A	3.1	1.3	2.6	1.1	10.1	1.3	3.4	1.1	2.6	1.1	3.0	N/A	3.0	1.2	2.2	1.1	3.6
6	6	3.4	1.1	2.7	1.1	2.6	1.2	2.1	1.1	2.5	1.1	2.2	1.2	2.5	N/A	2.2	1.2	2.6	1.1	2.5
7	6.5	1.9	1.1	2.4	1.1	2.7	1.3	2.2	1.1	2.4	1.2	2.2	1.4	2.6	N/A	2.7	1.1	2.2	1.1	2.4
8	7	1.6	1.0	1.7	1.1	1.7	1.5	2.0	1.0	3.0	1.2	2.2	1.1	1.9	N/A	2.2	1.0	2.5	1.1	2.1
9	7.5	18.5	1.2	3.5	1.2	3.5	1.3	9.4	1.2	7.5	1.6	4.1	1.3	3.9	1.2	3.9	1.3	3.4	1.2	6.4
10	8	4.5	1.1	3.9	1.1	5.1	1.4	5.8	1.1	6.7	1.3	11.2	1.4	3.9	1.2	4.3	1.2	3.5	1.2	5.5
11	8.5	4.1	1.1	4.0	1.1	3.2	1.3	4.1	1.3	8.7	1.2	5.0	1.5	3.6	N/A	3.2	1.1	2.9	1.1	4.3
12	9	3.0	1.0	4.4	1.1	3.9	1.5	2.9	1.1	3.5	1.2	3.7	1.2	3.3	N/A	3.3	1.1	3.2	1.2	3.5
13	9.5	11.8	1.2	7.5	1.3	5.5	1.6	10.2	1.2	8.4	1.4	7.6	1.4	9.6	1.2	6.9	1.3	1.6	1.0	7.7
14	10	6.9	1.2	8.1	1.2	5.6	1.4	12.3	1.2	7.8	1.4	9.6	1.4	8.0	1.3	6.7	1.3	8.2	1.3	8.1
15	10.5	6.0	1.3	10.7	1.2	4.3	1.6	6.1	1.3	9.5	1.3	7.6	1.4	7.6	1.2	7.2	1.2	6.8	1.3	7.3
16	11	5.6	1.1	9.3	1.2	3.8	1.4	6.3	1.3	8.0	1.2	5.5	1.3	6.6	1.1	6.2	1.2	6.3	1.2	6.4

Table S56. Binding conditions for surfaces printed under conditions shown in **Table S13**.

TPO (mM)	PETT (mM)	EGDMA (mM)	Intensity (mW/mm^2)	[**SCR043**] (µM)	[α-**Man-FL**] (M)
1	100	1300	2.53	50.0	$10^{-4.5}$

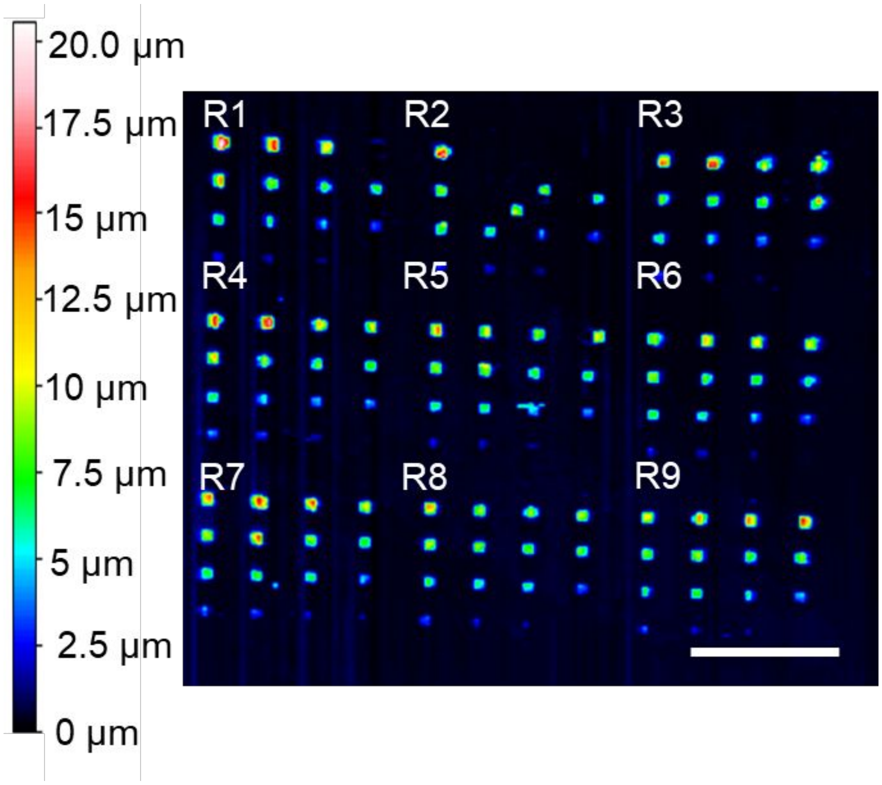

Figure S131. Profilometry image of patterns printed using [TPO] = 100 mM, [EGDMA] = 1300 mM. The pattern printed is 9 repeats (R1-R9) of 16 different time points. The scale bar is 200 µm.

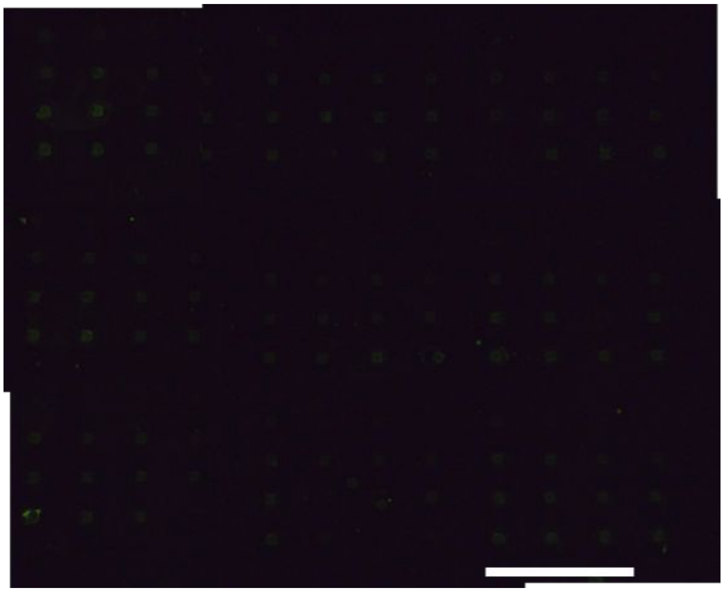

Figure S132. Fluorescence image of patterns printed using [TPO] = 100 mM, [EGDMA] = 1300 mM. Incubation was performed for 1 hour. The pattern printed is 9 repeats (R1-R9) of 16 different time points. The scale bar is 200 µm.

Table S57. Fluorescence intensity (*I*) and heights (*h*) of features printed under conditions in **Table S56**, and shown in **Figure S131** and **Figure S132**, # = feature number.

#	Time (min)	R1	*I*	R2	*I*	R3	*I*	R4	*I*	R5	*I*	R6	*I*	R7	*I*	R8	*I*	R9	*I*	Average Height (µm)
1	3.4	2.3	1.0	3.5	1.0	3.4	1.0	4.7	1.0	2.3	1.0	3.1	1.0	4.7	1.0	3.4	1.0	3.8	1.0	3.4
2	3.9	1.7	1.0	2.4	1.0	2.1	1.0	3.3	1.0	2.2	1.0	1.8	1.0	3.5	1.0	2.8	1.0	2.3	1.0	2.5
3	4.4	1.2	1.0	1.4	1.0	1.2	1.0	2.2	1.0	1.3	1.0	1.5	1.0	2.4	1.0	1.6	1.0	1.4	1.0	1.6
4	4.9	N/A	1.0	N/A	1.0	N/A	1.0	0.3	1.0	0.2	1.0	0.3	1.0	1.8	1.1	0.3	1.0	N/A	1.0	0.6
5	5.4	7.1	1.0	10.7	1.0	6.7	1.0	6.7	1.0	6.4	1.0	7.1	1.0	7.5	1.0	7.5	1.0	7.6	1.0	7.5
6	5.9	8.1	1.1	7.9	1.0	6.4	1.0	6.2	1.0	7.0	1.0	7.3	1.0	8.0	1.0	6.1	1.0	7.4	1.0	7.1
7	6.4	5.3	1.0	4.7	1.0	4.7	1.1	4.5	1.1	8.8	1.0	5.9	1.1	6.5	1.2	6.8	1.0	6.9	1.0	6.0
8	6.9	3.4	1.1	3.8	1.0	4.2	1.1	5.1	1.1	4.1	1.0	4.4	1.1	4.6	1.1	4.1	1.1	4.8	1.0	4.3
9	7.4	14.1	1.0	8.7	1.1	8.8	1.1	12.7	1.1	8.6	1.0	8.7	1.1	8.8	1.1	9.1	1.1	8.4	1.1	9.8
10	7.9	8.9	1.1	0.5	1.1	8.1	1.1	9.4	1.1	11.8	1.0	8.6	1.0	15.0	1.1	8.6	1.1	9.0	1.1	8.9
11	8.4	8.6	1.1	8.7	1.0	8.8	1.1	8.8	1.2	8.1	1.1	7.6	1.1	8.9	1.3	8.2	1.1	8.4	1.1	8.5
12	8.9	8.0	1.1	7.9	1.1	14.6	1.1	7.8	1.2	7.6	1.0	6.9	1.1	7.5	1.2	8.5	1.1	8.7	1.0	8.6
13	9.4	20.2	1.0	18.7	1.0	14.6	1.1	16.7	1.1	14.3	1.1	8.9	1.1	14.3	1.1	13.6	1.1	10.1	1.1	14.6
14	9.9	16.9	1.1	0.4	1.0	16.8	1.1	18.0	1.1	11.0	1.2	12.2	1.1	15.8	1.1	8.9	1.1	13.5	1.1	12.6
15	10.4	11.4	1.1	0.6	1.0	10.8	1.1	12.8	1.3	8.8	1.1	10.4	1.1	15.1	1.1	9.1	1.0	13.6	1.1	10.3
16	10.9	2.0	1.4	N/A	1.1	15.4	1.1	11.5	1.3	10.3	1.1	9.6	1.2	10.2	1.2	9.2	1.1	15.3	1.0	10.5

Table S58. Binding conditions for surfaces printed under conditions shown in **Table S13**.

TPO (mM)	PETT (mM)	EGDMA (mM)	Intensity (mW/mm^2)	[**SCR043**] (μM)	[α-**Man-FL**] (M)
1	100	1300	2.53	12.5	$10^{-4.5}$

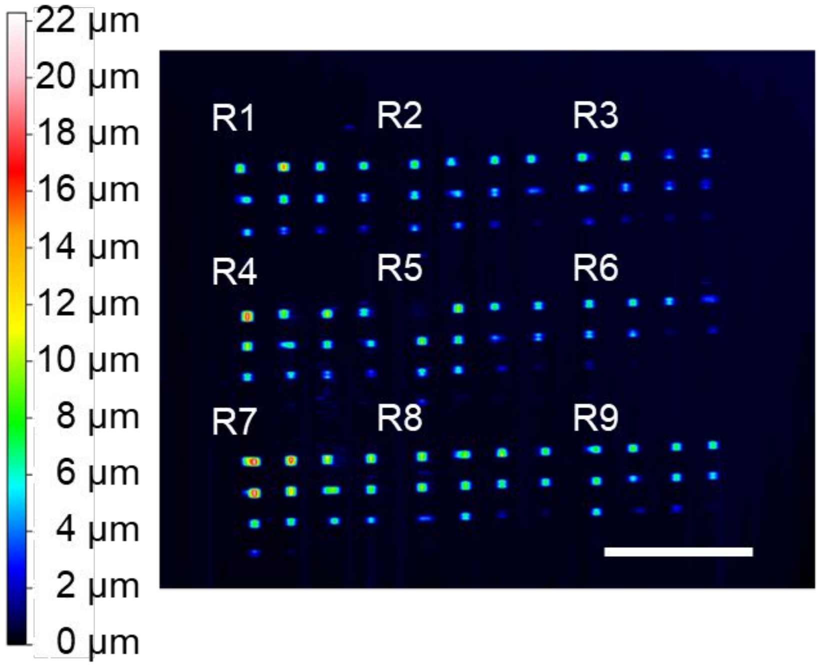

Figure S133. Profilometry image of patterns printed using [TPO] = 100 mM, [EGDMA] = 1300 mM. The pattern printed is 9 repeats (R1-R9) of 16 different time points. The scale bar is 200 μm.

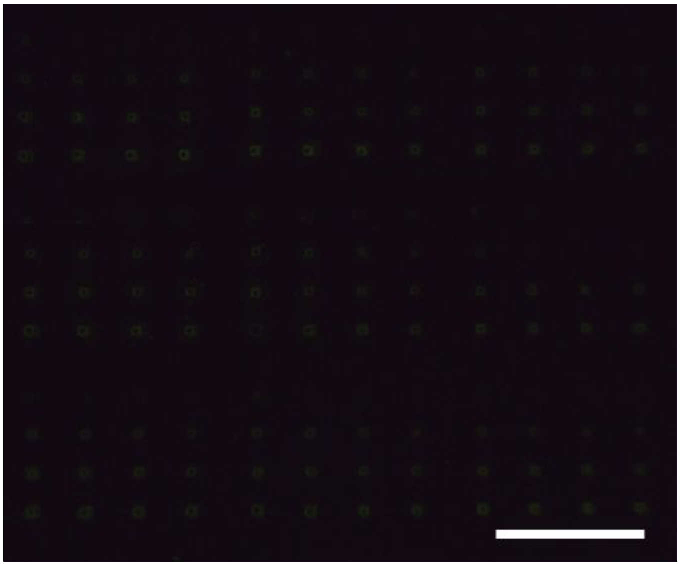

Figure S134. Fluorescence image of patterns printed using [TPO] = 100 mM, [EGDMA] = 1300 mM. Incubation was performed for 1 hour. The pattern printed is 9 repeats (R1-R9) of 16 different time points. The scale bar is 200 μm.

Table S59. Fluorescence intensity (*I*) and heights (*h*) of features printed under conditions in **Table S58**, and shown in **Figure S133** and **Figure S134**, # = feature number.

#	Time (min)	R1	*I*	R2	*I*	R3	*I*	R4	*I*	R5	*I*	R6	*I*	R7	*I*	R8	*I*	R9	*I*	Average Height (μm)
1	3.4	N/A	1.0	N/A	1.0	N/A	N/A	N/A	N/A	N/A	1.1	N/A	1.0	N/A	N/A	N/A	1.0	N/A	1.0	N/A
2	3.9	N/A	1.0	N/A	1.0	N/A	N/A	N/A	N/A	N/A	1.1	N/A	1.0	N/A	N/A	N/A	1.0	N/A	1.0	N/A
3	4.4	N/A	1.0	N/A	1.0	N/A	N/A	N/A	N/A	N/A	1.1	N/A	1.0	N/A	N/A	N/A	1.0	N/A	1.0	N/A
4	4.9	N/A	1.0	N/A	1.1	N/A	N/A	N/A	N/A	N/A	1.1	N/A	1.1	2.6	N/A	N/A	1.0	N/A	1.0	2.6
5	5.4	2.1	1.1	1.1	1.1	0.8	1.1	2.5	1.1	0.7	1.1	N/A	1.0	5.9	1.2	1.0	1.1	0.5	1.0	1.8
6	5.9	2.8	1.1	2.1	1.1	0.9	1.1	4.0	1.1	2.2	1.1	N/A	1.0	7.7	1.2	2.1	1.2	2.3	1.1	3.0
7	6.4	3.4	1.1	4.4	1.1	1.7	1.1	5.9	1.1	6.9	1.1	N/A	1.1	7.5	1.1	6.4	1.2	1.9	1.1	4.7
8	6.9	5.8	1.1	4.6	1.1	3.2	1.1	6.3	1.1	5.6	1.1	1.6	1.1	8.7	1.1	4.1	1.1	7.2	1.1	5.2
9	7.4	4.5	1.1	3.8	1.1	3.0	1.1	6.4	1.2	5.6	1.1	0.7	1.1	9.7	1.3	7.1	1.2	6.0	1.1	5.2
10	7.9	6.1	1.2	4.7	1.1	4.9	1.1	7.0	1.2	2.4	1.2	0.8	1.1	9.6	1.2	6.5	1.2	7.6	1.1	5.5
11	8.4	7.9	1.2	5.6	1.2	4.6	1.2	7.5	1.2	7.7	1.2	5.2	1.1	13.1	1.3	8.9	1.2	5.3	1.1	7.3
12	8.9	7.3	1.2	6.4	1.2	5.2	1.2	10.4	1.2	9.8	1.2	5.8	1.1	20.0	1.2	10.4	1.3	8.4	1.2	9.3
13	9.4	8.4	1.2	7.0	1.2	4.3	1.2	6.0	1.2	7.2	1.2	3.5	1.2	10.1	1.3	8.3	1.3	7.5	1.2	6.9
14	9.9	8.0	1.2	7.6	1.2	3.6	1.2	6.5	1.2	7.6	1.2	6.0	1.2	11.6	1.3	9.1	1.4	7.9	1.2	7.5
15	10.4	14.9	1.2	6.5	1.2	9.3	1.2	9.8	1.2	10.0	1.2	7.5	1.2	18.5	1.3	9.1	1.3	8.9	1.2	10.5
16	10.9	9.5	1.2	8.7	1.2	8.1	1.2	14.1	1.3	0.7	1.2	7.1	1.2	17.0	1.3	10.6	1.3	8.1	1.2	9.3

Table S60. Binding conditions for surfaces printed under conditions shown in **Table S13**.

TPO (mM)	PETT (mM)	EGDMA (mM)	Intensity (mW/mm^2)	[**SCR043**] (μM)	[α-**Man-FL**] (M)
1	100	1300	2.53	0	$10^{-4.5}$

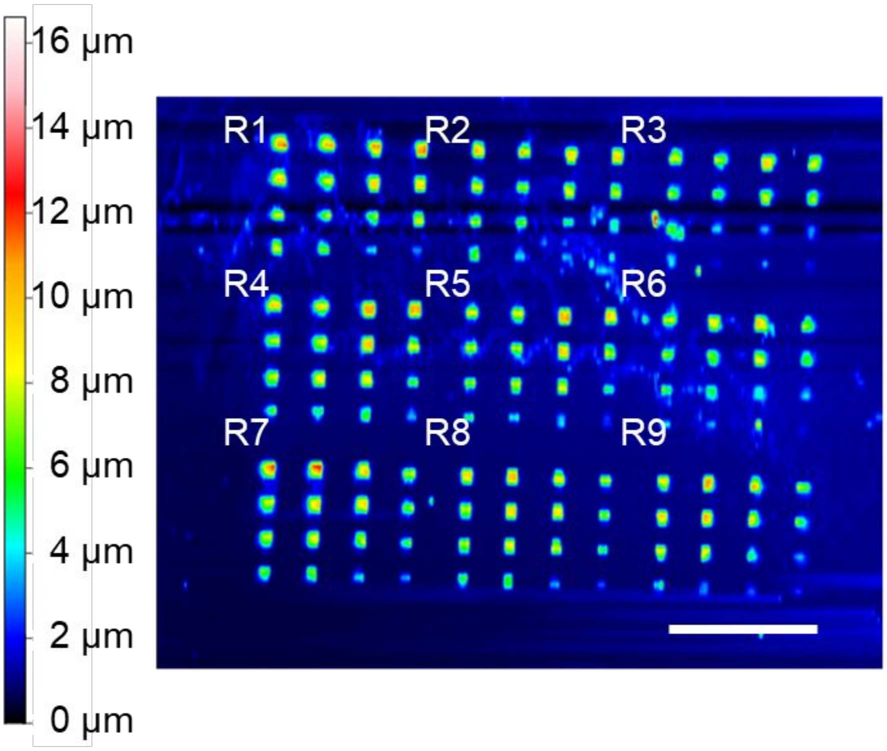

Figure S135. Profilometry image of patterns printed using [TPO] = 100 mM, [EGDMA] = 1300 mM. The pattern printed is 9 repeats (R1-R9) of 16 different time points. The scale bar is 200 μm.

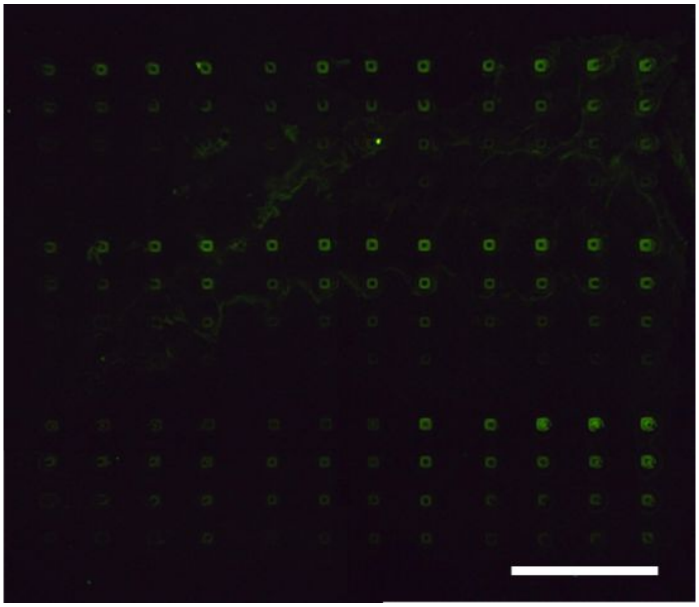

Figure S136. Fluorescence image of patterns printed using [TPO] = 100 mM, [EGDMA] = 1300 mM. Incubation was performed for 1 hour. The pattern printed is 9 repeats (R1-R9) of 16 different time points. The scale bar is 200 µm.

Table S61. Fluorescence intensity (*I*) and heights (*h*) of features printed under conditions in **Table S60**, and shown in **Figure S135** and **Figure S136**, # = feature number.

#	Time (min)	R1	*I*	R2	*I*	R3	*I*	R4	*I*	R5	*I*	R6	*I*	R7	*I*	R8	*I*	R9	*I*	Average Height (µm)
1	3.4	3.7	1.0	2.9	1.1	2.4	1.0	2.1	1.0	1.5	1.0	4.1	1.0	1.4	1.1	0.8	1.0	1.4	1.0	2.3
2	3.9	5.3	1.0	6.2	1.0	4.6	1.0	3.5	1.0	3.1	1.0	2.9	1.0	2.0	1.0	9.4	1.0	3.1	0.9	4.5
3	4.4	6.5	1.0	5.6	1.0	6.9	1.0	5.6	1.0	4.8	0.9	3.9	1.0	2.3	1.0	2.8	1.1	1.0	0.9	4.4
4	4.9	7.5	1.2	7.1	1.0	7.7	0.9	6.9	1.4	6.4	0.9	6.6	0.9	6.1	1.3	3.4	1.1	3.4	0.9	6.1
5	5.4	8.2	1.1	7.1	1.1	7.8	1.1	7.1	1.1	5.6	1.1	3.9	1.2	2.8	1.2	3.0	1.1	3.1	1.2	5.4
6	5.9	8.5	1.0	8.1	1.0	7.6	1.0	8.4	1.1	8.0	1.1	4.9	1.1	6.0	1.2	3.2	1.2	3.6	1.0	6.5
7	6.4	9.0	1.1	8.8	1.0	8.5	1.0	8.0	1.1	7.7	1.0	7.1	1.0	8.1	1.1	4.5	1.1	3.8	1.0	7.3
8	6.9	8.2	1.5	8.8	1.0	8.4	1.0	8.2	1.0	8.0	1.1	7.8	1.0	9.2	1.1	7.1	1.0	6.7	1.0	8.0
9	7.4	9.0	1.3	8.8	1.2	9.1	1.1	8.1	1.3	8.3	1.4	7.7	1.4	7.5	1.5	5.9	1.3	7.4	1.1	8.0
10	7.9	9.4	1.2	9.3	1.1	9.0	1.1	9.0	1.2	9.0	1.3	7.6	1.2	9.8	1.4	8.0	1.1	7.4	1.1	8.7
11	8.4	9.0	1.2	9.3	1.1	9.2	1.0	9.0	1.3	8.0	1.3	7.6	1.1	10.3	1.2	7.0	1.1	6.7	1.1	8.4
12	8.9	9.1	1.8	9.3	1.1	10.2	1.1	8.5	1.3	8.3	1.2	9.4	1.1	9.4	1.2	7.7	1.1	7.8	1.0	8.9
13	9.4	10.4	1.7	9.5	1.4	10.9	1.0	8.4	1.6	9.2	1.6	9.7	1.7	8.2	1.7	7.5	1.5	7.6	1.5	9.0
14	9.9	12.3	1.6	10.3	1.0	10.7	1.1	9.2	1.6	10.5	1.6	9.3	1.4	10.5	1.6	8.9	1.4	8.7	1.3	10.0
15	10.4	13.0	1.5	9.6	1.0	10.6	1.0	9.3	1.6	9.2	1.6	11.3	1.3	10.3	1.7	8.2	1.4	8.5	1.3	10.0
16	10.9			9.8	1.1	11.2	1.1	9.5	1.5	9.7	1.5	10.2	1.2	9.2	1.3	8.4	1.2	8.6	1.1	9.6

Table S62. Binding conditions for surfaces printed under conditions shown in **Table S13**.

TPO (mM)	PETT (mM)	EGDMA (mM)	Intensity (mW/mm^2)	[**SCR043**] (μM)	[α-**Man-FL**] (M)
1	100	1300	2.53	500	10^{-5}

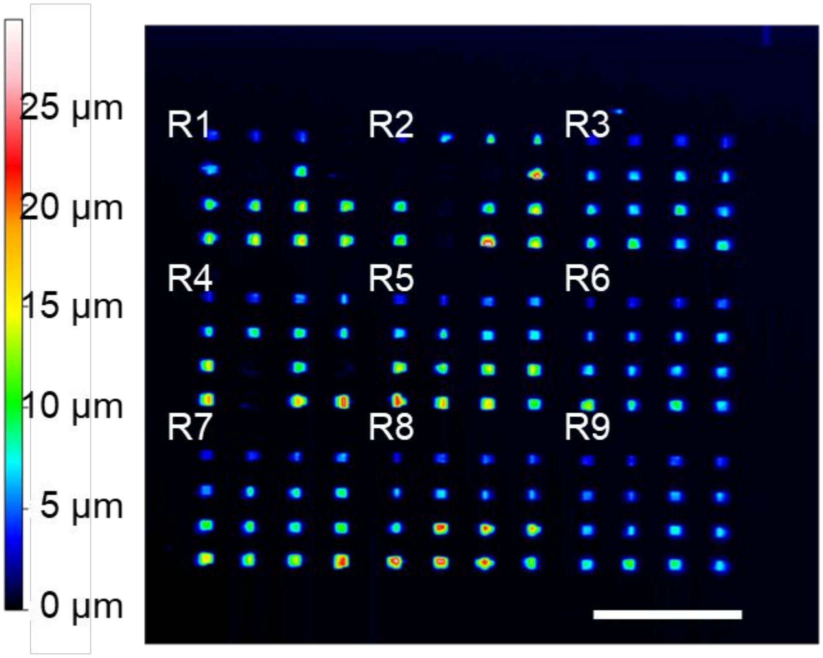

Figure S137. Profilometry image of patterns printed using [TPO:100mM], [EGDMA 1300mM. The pattern printed is 9 repeats (R1-R9) of 16 different time points. The scale bar is 200 μm.

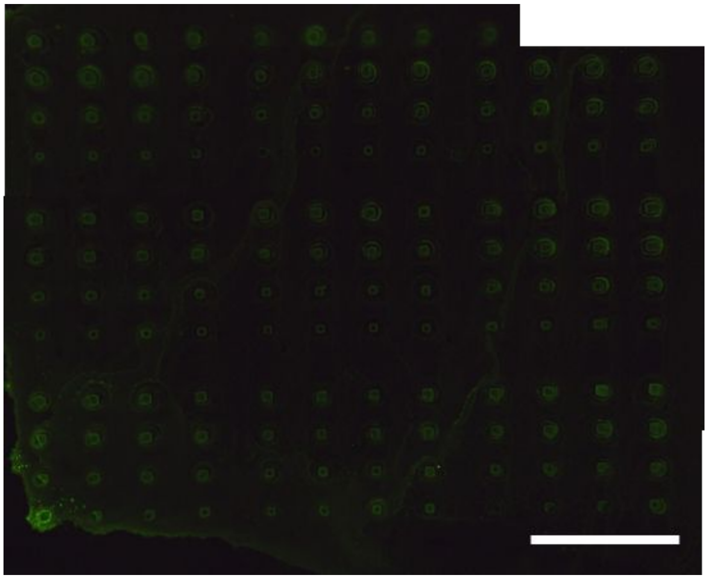

Figure S138. Fluorescence image of patterns printed using [TPO] = 100 mM, [EGDMA] = 1300 mM. Incubation was performed for 1 hour. The pattern printed is 9 repeats (R1-R9) of 16 different time points. The scale bar is 200 µm.

Table S63. Fluorescence intensity (*I*) and heights (*h*) of features printed under conditions in **Table S62**, and shown in **Figure S137** and **Figure S138**, # = feature number.

#	Time (min)	R1	*I*	R2	*I*	R3	*I*	R4	*I*	R5	*I*	R6	*I*	R7	*I*	R8	*I*	R9	*I*	Average Height (µm)
1	4.5	4.7	1.0	3.0	1.2	3.1	1.5	3.2	1.1	3.8	1.1	2.9	1.2	3.3	1.0	3.4	1.0	3.3	1.0	3.4
2	5	4.1	1.0	6.1	1.2	3.5	1.4	3.9	1.1	4.0	1.1	3.4	1.2	4.2	1.0	4.9	1.0	3.9	1.0	4.2
3	5.5	4.6	1.0	9.6	1.4	3.9	1.5	5.1	1.1	5.0	1.1	2.8	1.2	5.5	1.0	4.6	1.0	5.0	1.0	5.1
4	6	N/A	1.0	N/A	1.4	3.6	1.6	7.2	1.1	6.1	1.2	3.6	1.3	6.5	1.0	4.7	1.0	4.4	1.0	5.2
5	6.5	6.5	1.1	N/A	1.3	7.3	1.5	9.1	1.0	6.9	1.1	6.6	1.3	5.6	1.0	7.0	1.0	5.3	1.0	6.8
6	7	N/A	1.0	N/A	1.3	6.9	1.5	8.3	1.1	7.1	1.2	6.6	1.2	9.4	1.1	6.9	1.0	5.3	1.0	7.2
7	7.5	10.6	1.1	N/A	1.2	8.3	1.6	8.5	1.1	7.1	1.1	7.8	1.2	9.0	1.0	6.3	1.0	6.5	1.0	8.0
8	8	N/A	1.0	N/A	1.4	7.0	1.6	8.9	1.1	7.3	1.2	7.2	1.3	9.1	1.0	6.2	1.0	6.7	1.0	7.5
9	8.5	6.8	1.1	8.6	1.2	6.2	1.4	15.6	1.1	13.8	1.1	7.8	1.2	10.7	1.1	9.2	1.1	6.5	1.0	9.5
10	9	6.6	1.1	N/A	1.2	7.1	1.4	N/A	1.0	9.4	1.1	7.3	1.3	11.6	1.1	23.8	1.1	7.8	1.0	10.5
11	9.5	8.1	1.1	8.0	1.2	9.1	1.4	9.9	1.1	13.3	1.1	6.1	1.2	8.7	1.1	21.8	1.1	7.9	1.0	10.3
12	10	9.1	1.1	N/A	1.3	5.9	1.5	N/A	1.0	14.2	1.1	6.8	1.2	10.8	1.1	18.8	1.1	7.0	1.1	10.4
13	10.5	13.2	1.1	11.1	1.2	9.1	1.3	19.3	1.1	22.5	1.1	11.5	1.2	15.8	1.1	26.6	1.1	8.3	1.1	15.3
14	11	15.9	1.1	N/A	1.1	11.3	1.3	N/A	1.1	17.2	1.1	9.6	1.3	14.1	1.1	25.4	1.1	11.4	1.1	15.0
15	11.5	18.2	1.1	26.9	1.2	6.5	1.3	17.7	1.1	15.8	1.1	10.1	1.2	14.0	1.1	23.3	1.1	9.5	1.1	15.8
16	12	13.7	1.1	N/A	1.3	8.6	1.4	24.8	1.1	11.3	1.1	7.5	1.2	22.9	1.2	11.8	1.1	7.8	1.1	13.5

Table S64. Binding conditions for surfaces printed under conditions shown in **Table S13**.

TPO (mM)	PETT (mM)	EGDMA (mM)	Intensity (mW/mm2)	[**SCR043**] (μM)	[α-**Man-FL**] (M)
1	100	1300	2.53	250	10^{-5}

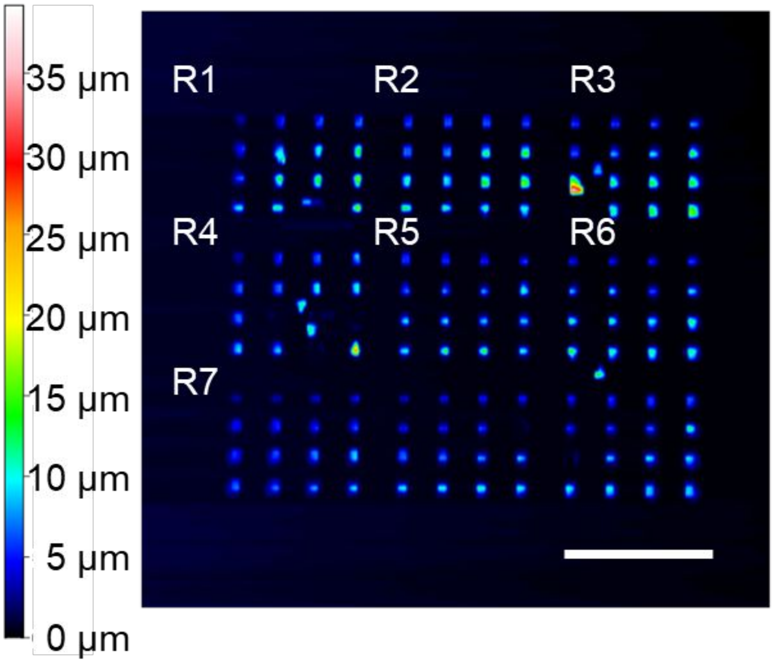

Figure S139. Profilometry image of patterns printed using [TPO] = 100 mM, [EGDMA] = 1300 mM. The pattern printed is 9 repeats (R1-R9) of 16 different time points. The scale bar is 200 μm.

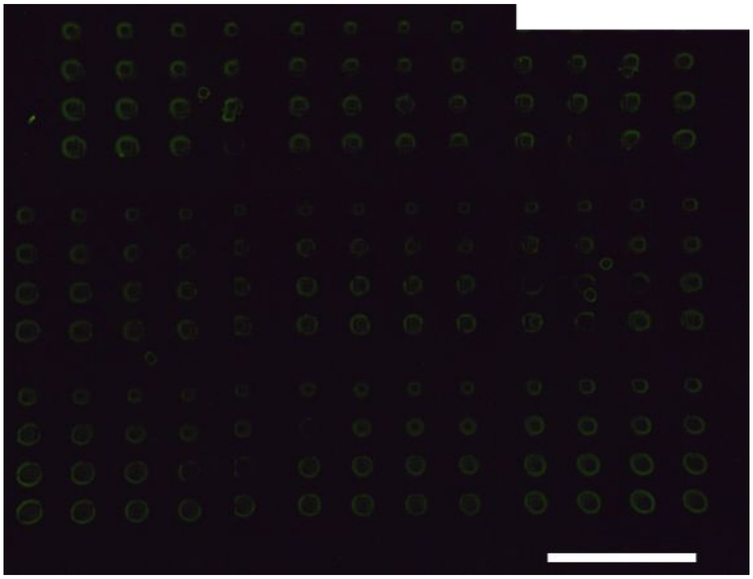

Figure S140. Fluorescence image of patterns printed using [TPO] = 100 mM, [EGDMA] = 1300 mM. Incubation was performed for 1 hour. The pattern printed is 9 repeats (R1-R9) of 16 different time points. The scale bar is 200 μm.

Table S65. Fluorescence intensity (*I*) and heights (*h*) of features printed under conditions in **Table S64**, and shown in **Figure S139** and **Figure S140**, # = feature number.

#	Time (min)	R1	*I*	R2	*I*	R3	*I*	R4	*I*	R5	*I*	R6	*I*	R7	*I*	R8	*I*	R9	*I*	Average Height (μm)
1	4.5	5.0	1.1	3.3	1.5	2.7	1.9	4.7	1.1	5.0	1.2	2.7	1.4	5.0	1.2	5.5	1.2	2.7	1.3	4.1
2	5	5.1	1.1	3.5	1.5	2.8	1.9	4.2	1.2	5.1	1.2	4.5	1.4	5.4	1.3	5.8	1.2	4.2	1.3	4.5
3	5.5	5.5	1.2	4.5	1.5	3.3	2.0	4.3	1.2	4.9	1.2	N/A	1.4	6.5	1.4	5.9	1.2	5.9	1.3	5.1
4	6	6.4	1.2	4.7	1.4	3.7	1.9	6.3	1.3	4.3	1.3	8.8	1.4	8.3	1.4	5.7	1.3	6.5	1.3	6.1
5	6.5	5.4	1.2	4.6	1.6	4.3	2.1	8.7	1.2	5.6	1.3	5.2	1.6	6.2	1.3	6.0	1.3	4.0	1.4	5.6
6	7	5.3	1.2	4.5	1.6	4.3	2.1	8.1	1.2	6.4	1.3	5.8	1.6	8.2	1.4	7.7	1.3	10.1	1.3	6.7
7	7.5	6.6	1.2	6.4	1.7	5.6	2.2	6.2	1.3	8.3	1.4	6.3	1.5	9.6	1.4	12.4	1.3	10.9	1.3	8.0
8	8	13.0	1.2	N/A	1.2	5.5	2.1	10.2	1.3	11.7	1.4	10.7	1.5	14.2	1.4	13.6	1.3	12.3	1.3	11.4
9	8.5	N/A	1.1	6.5	1.7	5.1	2.0	12.7	1.3	10.1	1.4	6.0	1.7	37.0	1.4	10.9	1.3	5.7	1.4	11.8
10	9	8.6	1.2	6.8	1.7	5.6	2.2	10.9	1.3	10.0	1.5	N/A	1.4	12.2	1.4	8.3	1.3	14.6	1.3	9.6
11	9.5	8.8	1.2	8.1	1.7	7.0	2.3	10.1	1.3	8.4	1.5	10.1	1.4	13.1	1.4	14.4	1.3	13.3	1.0	10.4
12	10	9.3	1.2	8.8	1.7	8.6	2.2	12.5	1.3	8.4	1.5	N/A	1.3	14.9	1.4	16.9	1.4	16.4	1.3	12.0
13	10.5	9.5	1.2	9.7	1.6	6.1	2.0	15.1	1.3	10.1	1.5	7.7	1.7	N/A	1.1	12.8	1.3	9.7	N/A	10.1
14	11	9.9	1.2	10.6	1.7	5.9	2.1	11.2	1.3	14.3	1.6	7.8	1.9	11.0	1.4	9.8	1.4	11.3	N/A	10.2
15	11.5	8.1	1.2	10.8	1.8	7.9	2.1	11.3	1.3	11.3	1.6	N/A	1.4	12.6	1.4	11.0	1.4	N/A	N/A	10.4
16	12	8.6	1.2	10.2	1.8	9.2	2.1	11.9	1.3	10.9	1.5	26.5	1.8	16.6	1.4	9.9	1.4	16.5	N/A	13.4

Table S66. Binding conditions for surfaces printed under conditions shown in **Table S13**.

TPO (mM)	PETT (mM)	EGDMA (mM)	Intensity (mW/mm^2)	[**SCR043**] (µM)	[**α-Man-FL**] (M)
1	100	1300	2.53	100	10^{-5}

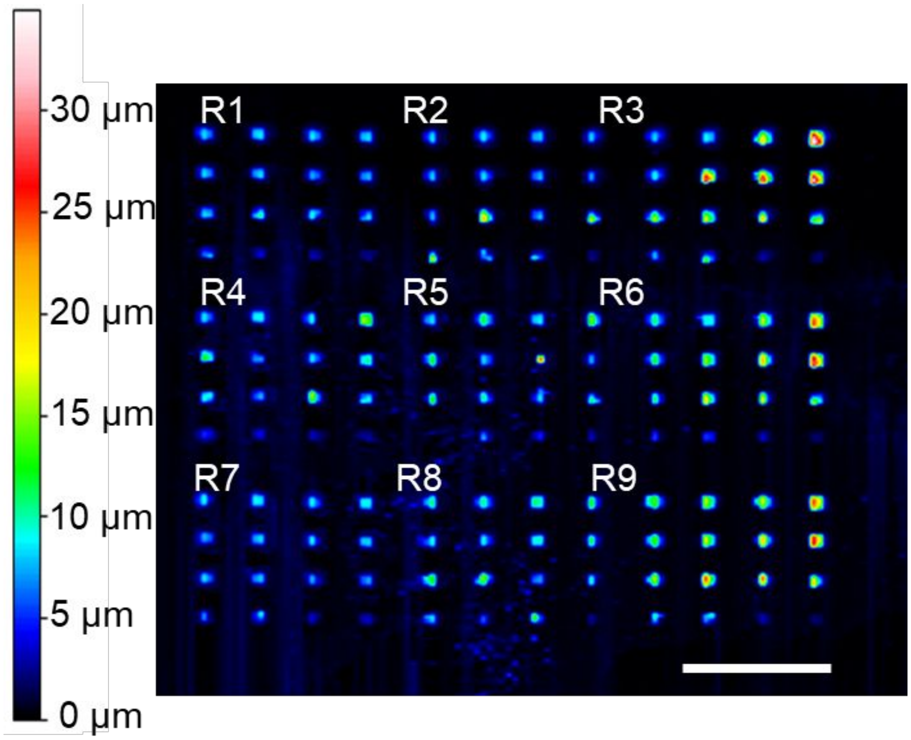

Figure S141. Profilometry image of patterns printed using [TPO] = 100 mM, [EGDMA] = 1300 mM. The pattern printed is 9 repeats (R1-R9) of 16 different time points. The scale bar is 200 µm.

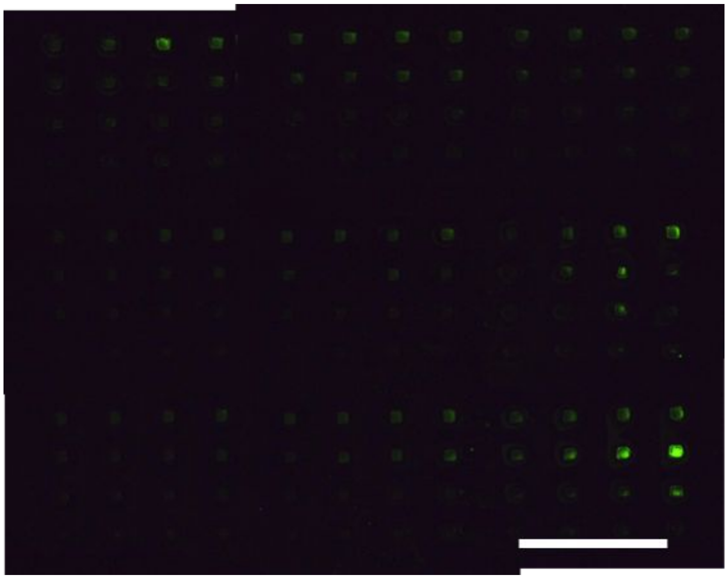

Figure S142. Fluorescence image of patterns printed using [TPO] = 100 mM, [EGDMA] = 1300 mM. Incubation was performed for 1 hour. The pattern printed is 9 repeats (R1-R9) of 16 different time points. The scale bar is 200 µm.

Table S67. Fluorescence intensity (*I*) and heights (*h*) of features printed under conditions in **Table S66**, and shown in **Figure S141** and **Figure S142**, # = feature number.

#	Time (min)	R1	*I*	R2	*I*	R3	*I*	R4	*I*	R5	*I*	R6	*I*	R7	*I*	R8	*I*	R9	*I*	Average Height (µm)
1	3.5	8.5	1.1	16.6	1.1	7.5	1.1	4.8	1.2	2.8	1.0	5.5	1.0	6.7	1.1	8.4	1.1	10.4	1.0	7.9
2	4	4.0	1.1	8.2	1.1	14.7	1.1	3.8	1.1	7.4	1.0	5.0	1.0	9.3	1.1	5.5	1.1	10.5	1.0	7.6
3	4.5	2.9	1.1	11.2	1.0	3.0	1.0	3.4	1.0	4.1	1.0	3.7	1.0	3.4	1.1	13.8	1.0	2.7	1.0	5.4
4	5	2.6	1.0	2.5	1.0	1.8	1.0	2.7	1.0	3.4	1.0	1.6	1.0	3.2	1.0	2.3	1.0	1.7	1.0	2.4
5	5.5	8.1	1.1	7.9	1.1	16.4	1.1	9.1	1.1	12.5	1.0	11.2	1.1	8.6	1.6	12.6	1.1	15.7	1.0	11.3
6	6	10.5	1.1	21.5	1.1	17.0	1.1	7.6	1.4	10.4	1.1	19.8	1.0	7.9	1.2	14.9	1.1	25.2	1.0	15.0
7	6.5	9.8	1.1	8.1	1.1	21.4	1.1	15.6	1.1	7.8	1.0	17.1	1.0	7.4	1.1	7.1	1.0	24.5	1.0	13.2
8	7	8.6	1.0	16.8	1.1	15.9	1.0	9.6	1.1	11.3	1.0	15.8	1.0	7.2	1.1	9.6	1.0	20.7	1.0	12.8
9	7.5	8.3	1.1	8.2	1.3	9.1	1.2	11.8	1.2	12.1	1.1	11.9	1.1	6.7	3.6	9.0	1.3	13.1	1.1	10.0
10	8	8.4	1.2	8.4	1.3	29.6	1.1	6.5	1.5	8.0	1.2	14.9	1.1	7.4	2.3	8.8	1.2	16.8	1.1	12.1
11	8.5	7.5	1.1	7.8	1.1	29.3	1.1	8.6	1.2	32.3	1.0	21.1	1.1	8.9	1.5	9.8	1.1	20.4	1.1	16.2
12	9	7.5	1.1	7.3	1.2	32.8	1.1	10.2	1.1	7.3	1.1	29.0	1.1	7.9	1.2	11.2	1.1	28.0	1.1	15.7
13	9.5	8.1	1.3	8.5	1.4	8.0	1.5	7.9	2.0	8.3	1.4	10.2	1.3	8.9	1.6	10.0	1.3	13.1	1.2	9.2
14	10	8.5	1.3	8.7	1.5	8.9	1.9	8.0	1.6	11.8	1.2	8.8	1.2	8.3	1.4	10.3	1.2	16.0	1.1	9.9
15	10.5	8.6	1.3	8.0	1.4	19.4	1.2	8.9	1.3	8.5	1.2	15.7	1.1	8.5	1.3	10.7	1.2	16.4	1.1	11.6
16	11	8.2	1.3	8.1	1.3	34.7	1.2	15.4	1.1	14.0	1.2	24.8	1.1	9.2	1.2	12.1	1.1	25.1	1.1	16.8

Table S68. Binding conditions for surfaces printed under conditions shown in **Table S13**.

TPO (mM)	PETT (mM)	EGDMA (mM)	Intensity (mW/mm^2)	[**SCR043**] (µM)	[α-**Man-FL**] (M)
1	100	1300	2.53	50.0	10^{-5}

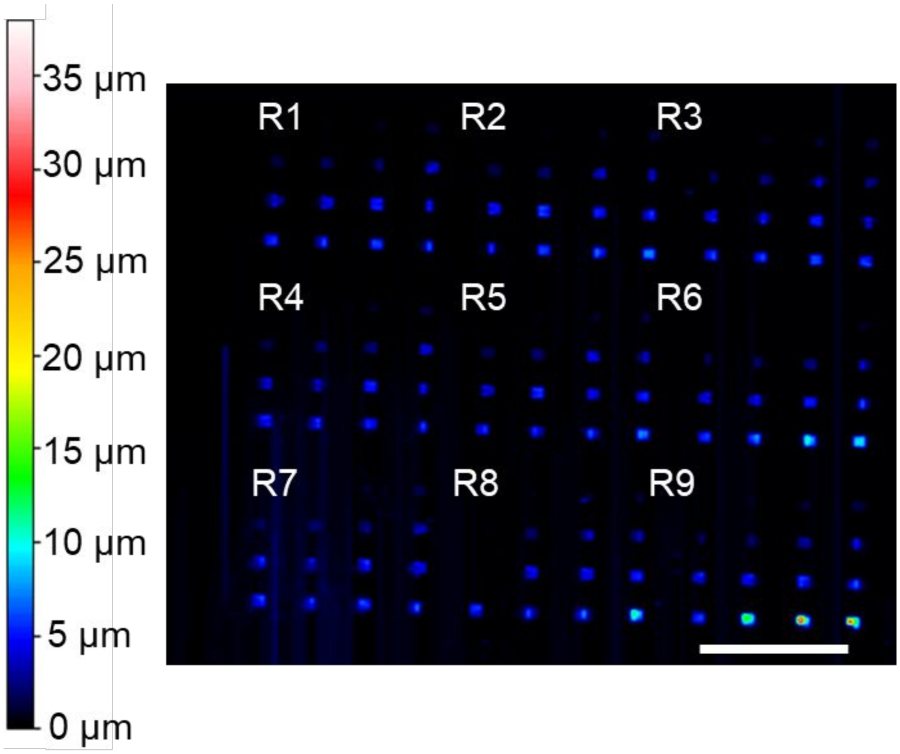

Figure S143. Profilometry image of patterns printed using [TPO] = 100 mM, [EGDMA] = 1300 mM. The pattern printed is 9 repeats (R1-R9) of 16 different time points. The scale bar is 200 µm.

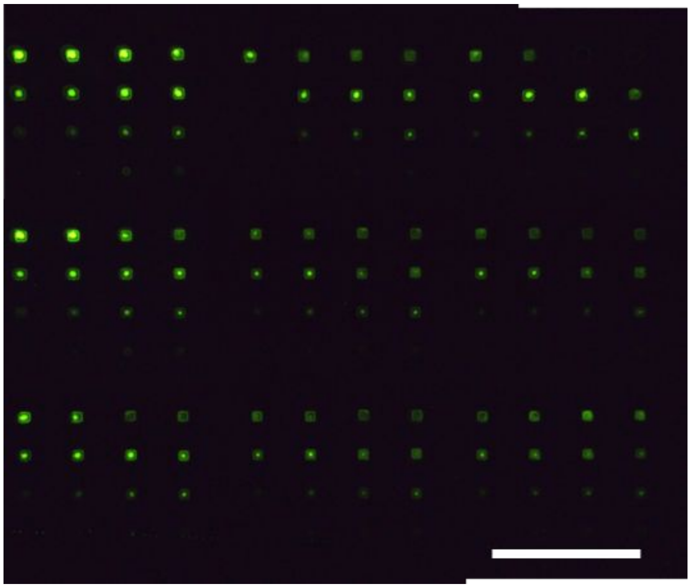

Figure S144. Fluorescence image of patterns printed using [TPO] = 100 mM, [EGDMA] = 1300 mM. Incubation was performed for 1 hour. The pattern printed is 9 repeats (R1-R9) of 16 different time points. The scale bar is 200 μm.

Table S69. Fluorescence intensity (*I*) and heights (*h*) of features printed under conditions in **Table S68**, and shown in **Figure S143** and **Figure S144**, # = feature number.

#	Time (min)	R1	*I*	R2	*I*	R3	*I*	R4	*I*	R5	*I*	R6	*I*	R7	*I*	R8	*I*	R9	*I*	Average Height (μm)
1	3.4	1.0	1.0	1.2	1.0	1.3	1.0	1.2	1.0	1.4	1.0	0.8	1.0	1.1	1.0	1.2	1.0	1.3	1.0	1.2
2	3.9	0.9	1.0	0.9	1.0	1.0	1.0	1.1	1.0	1.0	1.0	N/A	1.0	1.2	1.0	4.0	1.0	1.1	1.0	1.4
3	4.4	0.4	1.0	0.4	1.0	0.6	1.0	N/A	1.0	0.5	1.0	N/A	1.0	N/A	1.1	0.3	1.0	0.7	1.0	0.5
4	4.9	N/A	1.0	0.2	1.0	N/A	1.0	0.1	1.0	0.8	1.0	N/A	1.0	0.1	1.0	0.0	1.0	0.1	1.0	0.2
5	5.4	4.6	1.0	4.5	1.0	2.6	1.0	4.2	1.1	4.4	1.0	4.2	1.0	3.5	1.1	3.6	1.0	4.9	1.0	4.0
6	5.9	2.6	1.1	3.9	1.1	4.3	1.1	2.7	1.1	4.2	1.1	2.9	1.0	3.0	1.2	4.4	1.1	3.4	1.1	3.5
7	6.4	1.7	1.2	2.2	1.1	2.7	1.1	1.9	1.3	1.9	1.2	2.2	1.1	1.8	1.4	1.7	1.2	1.9	1.3	2.0
8	6.9	1.3	1.3	1.4	1.2	1.8	1.1	1.3	1.3	1.5	1.3	1.8	1.2	1.5	1.4	0.6	1.3	1.6	1.5	1.4
9	7.4	5.2	1.7	5.8	1.4	5.1	1.4	5.0	1.8	5.4	1.4	6.3	1.5	5.0	2.1	5.2	1.0	6.4	1.7	5.5
10	7.9	5.6	2.0	5.2	1.6	5.1	1.5	5.4	2.0	4.8	1.7	5.0	1.6	6.1	2.2	5.2	1.9	6.0	2.1	5.4
11	8.4	4.6	2.1	6.5	1.4	5.4	1.5	4.5	1.9	6.0	1.4	4.9	1.5	5.2	2.8	4.8	2.3	5.5	2.6	5.3
12	8.9	4.5	1.7	5.2	1.4	5.5	1.2	4.5	1.8	5.0	1.5	5.0	1.4	5.2	2.8	0.0	1.9	6.2	1.5	4.6
13	9.4	6.4	2.5	7.2	1.4	6.4	1.3	6.1	3.2	7.0	1.6	9.0	1.4	6.9	3.7	12.0	2.3	29.4	1.8	10.0
14	9.9	6.0	1.9	6.7	1.4	6.3	1.5	5.4	3.2	6.4	1.5	10.9	1.3	6.3	3.9	7.1	1.7	37.8	1.4	10.3
15	10.4	6.3	1.3	5.9	1.3	6.1	1.8	5.9	2.0	5.5	1.4	7.7	1.2	6.0	3.7	6.1	1.6	14.5	1.0	7.1
16	10.9	5.7	1.4	5.6	1.3	6.2	1.5	5.4	1.6	5.5	1.3	5.9	1.2	5.9	2.7	5.9	1.5	5.9	1.0	5.8

Table S70. Binding conditions for surfaces printed under conditions shown in **Table S13**.

TPO (mM)	PETT (mM)	EGDMA (mM)	Intensity (mW/mm^2)	[**SCR043**] (µM)	[α-**Man-FL**] (M)
1	100	1300	2.53	12.5	10^{-5}

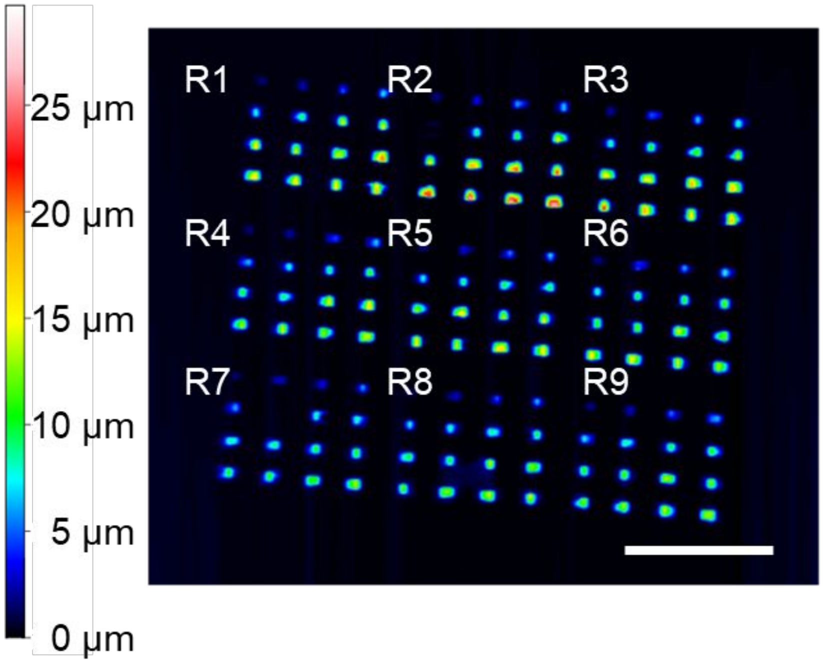

Figure S145. Profilometry image of patterns printed using [TPO] = 100 mM, [EGDMA] = 1300 mM. The pattern printed is 9 repeats (R1-R9) of 16 different time points. The scale bar is 200 µm.

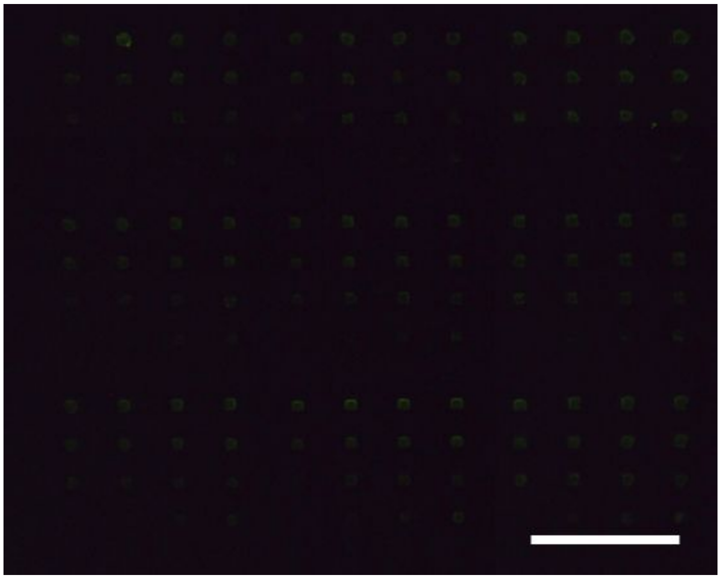

Figure S146. Fluorescence image of patterns printed using [TPO] = 100 mM, [EGDMA] = 1300 mM. Incubation was performed for 1 hour. The pattern printed is 9 repeats (R1-R9) of 16 different time points. The scale bar is 200 µm.

Table S71. Fluorescence intensity (*I*) and heights (*h*) of features printed under conditions in **Table S70**, and shown in **Figure S145** and **Figure S146**, # = feature number.

#	Time (min)	R1	*I*	R2	*I*	R3	*I*	R4	*I*	R5	*I*	R6	*I*	R7	*I*	R8	*I*	R9	*I*	Average Height (µm)
1	3.4	3.6	1.1	3.3	1.1	5.5	1.1	6.3	1.1	6.9	1.1	7.6	1.1	8.4	1.1	8.0	1.2	9.9	1.1	6.6
2	3.9	2.5	1.0	3.1	1.0	3.8	1.0	4.3	1.1	6.0	1.1	5.6	1.1	5.6	1.1	7.0	1.1	7.9	1.1	5.1
3	4.4	0.9	1.0	2.4	1.0	2.6	1.0	2.6	1.0	3.3	1.0	3.7	1.1	4.2	1.0	4.6	1.1	6.2	1.1	3.4
4	4.9	0.5	1.0	1.3	1.0	1.1	1.0	1.8	1.0	2.0	1.0	2.1	1.0	3.5	1.0	3.7	1.0	3.9	1.0	2.2
5	5.4	7.4	1.1	9.4	1.1	10.8	1.2	9.2	1.1	7.8	1.1	11.6	1.1	16.5	1.1	11.6	1.1	13.5	1.1	10.9
6	5.9	8.1	1.1	8.8	1.2	10.1	1.2	8.8	1.1	9.4	1.1	10.5	1.1	16.0	1.1	11.4	1.1	12.0	1.1	10.6
7	6.4	N/A	1.0	8.0	1.1	8.4	1.2	6.7	1.1	8.7	1.1	10.2	1.2	10.1	1.1	9.9	1.1	10.3	1.1	9.0
8	6.9	2.9	1.1	6.6	1.1	6.7	1.2	3.5	1.1	7.0	1.1	7.3	1.2	8.5	1.1	0.0	1.0	8.3	1.2	5.6
9	7.4	11.1	1.2	12.2	1.2	12.0	1.3	16.6	1.2	12.8	1.2	11.0	1.2	20.6	1.2	22.0	1.2	18.9	1.2	15.3
10	7.9	10.4	1.2	15.3	1.1	12.9	1.2	15.0	1.1	12.3	1.2	13.2	1.2	14.9	1.2	22.1	1.2	18.1	1.2	14.9
11	8.4	8.8	1.2	10.9	1.2	12.1	1.2	9.4	1.2	14.6	1.2	12.0	1.2	14.2	1.2	18.8	1.2	17.0	1.2	13.1
12	8.9	8.4	1.1	10.1	1.2	11.1	1.2	10.7	1.1	11.1	1.1	10.2	1.2	17.5	1.1	15.6	1.1	17.7	1.2	12.5
13	9.4	12.0	1.2	10.4	1.2	12.2	1.3	12.7	1.2	15.8	1.2	10.0	1.2	16.3	1.2	24.5	1.3	16.4	1.2	14.5
14	9.9	9.2	1.2	12.0	1.3	12.5	1.3	12.2	1.2	12.8	1.3	12.4	1.3	15.3	1.2	22.3	1.3	16.2	1.2	13.9
15	10.4	8.5	1.4	11.1	1.2	10.7	1.3	14.0	1.2	12.0	1.2	12.2	1.2	16.0	1.2	20.1	1.3	19.4	1.2	13.8
16	10.9	8.2	1.2	10.2	1.2	9.0	1.2	12.1	1.2	13.3	1.2	13.8	1.2	17.0	1.2	19.4	1.3	20.3	1.3	13.7

Table S72. Binding conditions for surfaces printed under conditions shown in **Table S13**.

TPO (mM)	PETT (mM)	EGDMA (mM)	Intensity (mW/mm^2)	[**SCR043**] (μM)	[α-**Man-FL**] (M)
1	100	1300	2.53	0	10^{-5}

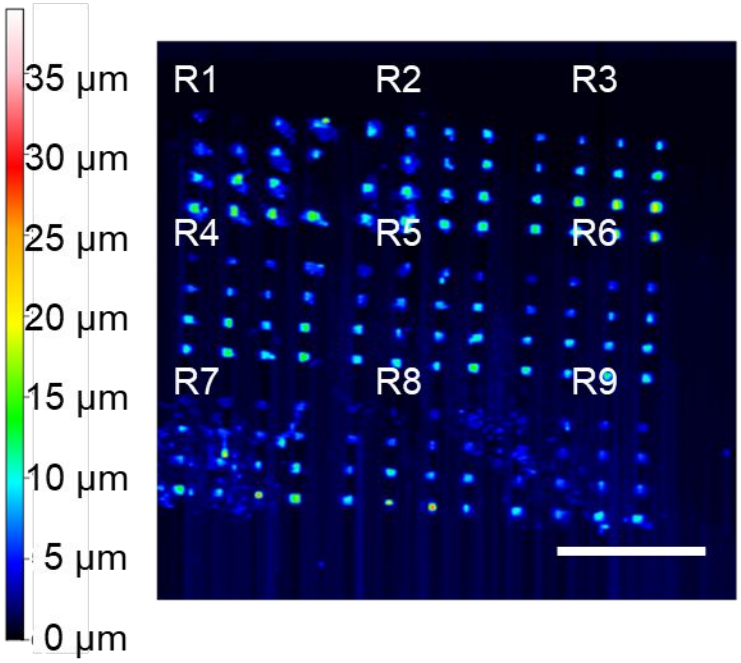

Figure S147. Profilometry image of patterns printed using [TPO] = 100 mM, [EGDMA] = 1300 mM. The pattern printed is 9 repeats (R1-R9) of 16 different time points. The scale bar is 200 μm.

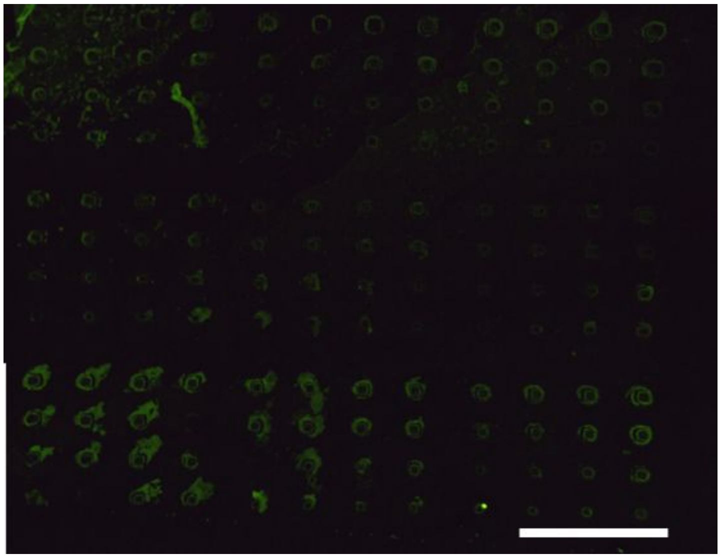

Figure S148. Fluorescence image of patterns printed using [TPO] = 100 mM, [EGDMA] = 1300 mM. Incubation was performed for 1 hour. The pattern printed is 9 repeats (R1-R9) of 16 different time points. The scale bar is 200 µm.

Table S73. Fluorescence intensity (*I*) and heights (*h*) of features printed under conditions in **Table S72**, and shown in **Figure S147** and **Figure S148**, # = feature number.

#	Time (min)	R1	*I*	R2	*I*	R3	*I*	R4	*I*	R5	*I*	R6	*I*	R7	*I*	R8	*I*	R9	*I*	Average Height (µm)
1	3.4	5.3	1.5	3.5	1.6	4.3	1.4	5.7	N/A	4.4	1.6	5.2	1.1	N/A	1.2	N/A	1.2	10.5	1.1	11.5
2	3.9	4.6	1.2	3.5	1.3	5.6	1.2	5.3	N/A	9.1	1.3	4.9	1.1	N/A	1.1	9.8	1.1	8.3	1.1	12.7
3	4.4	2.9	1.1	2.9	1.1	3.5	1.1	6.1	N/A	3.6	1.1	5.0	1.1	8.8	1.0	9.0	1.0	7.9	1.0	13.2
4	4.9	2.6	1.0	3.3	1.0	3.3	1.0	3.0	N/A	3.4	1.0	3.5	1.0	7.3	1.0	8.6	1.0	6.9	0.9	12.9
5	5.4	7.0	1.7	5.4	1.7	6.2	1.8	7.9	N/A	9.9	1.7	8.9	1.1	5.4	1.3	8.5	1.4	13.6	1.1	9.4
6	5.9	6.4	1.3	5.3	1.3	6.1	1.3	7.5	N/A	8.6	1.4	8.7	1.1	10.4	1.1	12.4	1.1	10.4	1.1	11.3
7	6.4	6.1	1.2	6.1	1.1	4.6	1.1	8.2	N/A	7.9	1.1	7.1	1.1	9.7	1.0	8.5	1.1	9.7	1.0	9.6
8	6.9	7.4	1.0	4.7	1.0	4.3	1.0	7.4	N/A	6.0	1.0	6.2	1.0	7.1	1.0	10.7	1.0	9.1	1.0	10.1
9	7.4	10.9	1.7	4.5	1.7	2.1	1.8	13.2	1.4	11.2	1.7	11.4	1.2	6.1	1.4	12.4	1.4	19.5	1.1	7.0
10	7.9	7.9	1.4	5.6	1.3	5.4	1.4	9.0	1.2	10.2	1.4	9.5	1.2	11.4	1.1	10.4	1.2	17.3	1.1	7.5
11	8.4	9.2	1.2	8.4	1.1	4.2	1.1	13.2	1.5	9.8	1.1	11.6	1.1	17.6	1.0	12.0	1.1	15.5	1.1	8.4
12	8.9	8.7	1.1	6.3	1.0	5.2	1.1	11.4	1.0	10.1	1.0	11.4	1.0	10.1	1.0	11.2	1.0	10.0	1.0	8.1
13	9.4	16.2	1.7	8.0	1.8	6.7	1.9	13.4	1.5	14.5	1.6	8.4	N/A	15.6	1.6	13.0	1.8	20.0	1.1	4.7
14	9.9	22.9	1.5	11.7	1.5	9.5	1.6	9.7	1.3	11.2	1.5	9.3	N/A	16.4	1.1	11.8	1.4	16.5	1.1	5.5
15	10.4	11.1	1.3	15.0	1.2	6.5	1.3	13.2	1.2	11.4	1.1	10.2	N/A	17.6	1.2	11.6	1.2	17.3	1.1	6.4
16	10.9	13.6	1.1	9.9	1.1	8.5	1.2	11.0	1.1	9.8	1.0	11.5	N/A	15.7	1.0	9.9	1.0	13.4	1.1	5.5

Table S74. Binding conditions for surfaces printed under conditions shown in **Table S13**.

TPO (mM)	PETT (mM)	EGDMA (mM)	Intensity (mW/mm2)	[**SCR043**] (μM)	[α-**Man-FL**] (M)
1	100	1300	2.53	500	$10^{-5.5}$

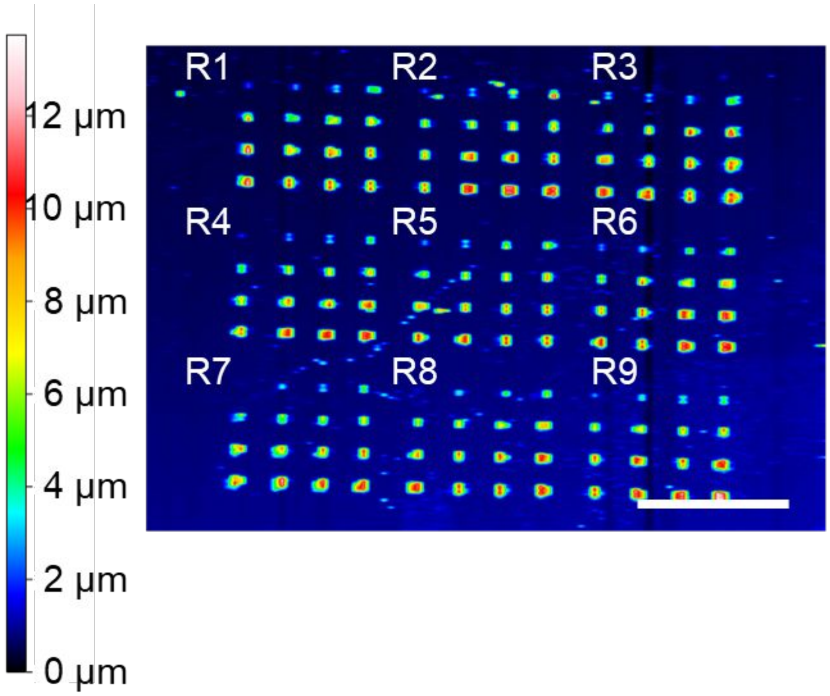

Figure S149. Profilometry image of patterns printed using [TPO] = 100 mM, [EGDMA] = 1300 mM. The pattern printed is 9 repeats (R1-R9) of 16 different time points. The scale bar is 200 μm.

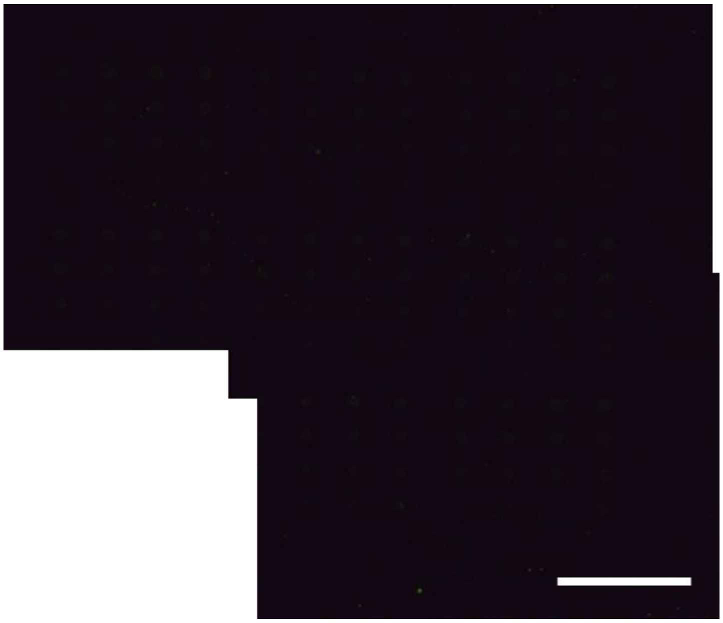

Figure S150. Fluorescence image of patterns printed using [TPO] :100mM, [EGDMA] 1300mM. Incubation was performed for 1 hour. The pattern printed is 9 repeats (R1-R9) of 16 different time points. The scale bar is 200 μm.

Table S75. Fluorescence intensity (*I*) and heights (*h*) of features printed under conditions in **Table S74**, and shown in **Figure S149** and **Figure S150**, # = feature number.

#	Time (min)	R1	*I*	R2	*I*	R3	*I*	R4	*I*	R5	*I*	R6	*I*	R7	*I*	R8	*I*	R9	*I*	Average Height (μm)
1	4.5	1.6	N/A	1.5	1.0	2.1	1.0	1.1	N/A	2.0	1.0	1.5	1.0	0.2	N/A	0.6	1.0	1.7	1.0	1.4
2	5	1.9	N/A	2.0	1.0	2.1	1.0	1.8	N/A	2.0	1.0	2.6	1.0	3.1	N/A	1.2	1.0	2.9	1.0	2.2
3	5.5	3.5	N/A	5.9	1.0	2.7	1.0	2.1	N/A	5.4	1.0	5.3	1.0	2.1	N/A	2.7	1.0	2.6	1.0	3.6
4	6	N/A	N/A	N/A	1.0	4.5	1.0	4.3	N/A	5.8	1.0	5.3	1.0	3.8	N/A	5.0	1.0	4.1	1.0	4.7
5	6.5	9.5	N/A	6.1	1.0	5.3	1.0	4.8	1.1	5.5	1.0	5.8	1.0	2.5	1.0	5.4	1.0	6.4	1.0	5.7
6	7	4.4	N/A	6.6	1.0	7.9	1.0	6.3	1.0	5.6	1.1	8.9	1.0	4.2	1.1	6.6	1.1	5.8	1.0	6.3
7	7.5	6.1	N/A	7.5	1.0	10.1	1.1	7.2	1.1	6.7	1.0	5.8	1.1	6.5	1.1	6.4	1.1	7.0	1.1	7.0
8	8	7.4	N/A	8.4	1.0	11.2	1.0	7.8	1.1	8.2	1.1	8.0	1.1	6.5	1.1	7.2	1.0	7.1	1.1	8.0
9	8.5	12.7	N/A	4.5	1.1	8.1	1.0	6.1	1.1	6.4	1.1	8.4	1.1	5.2	1.0	8.1	1.0	8.4	1.0	7.6
10	9	7.7	N/A	6.2	1.0	6.6	1.1	8.4	1.1	8.2	1.0	9.1	1.1	6.7	1.1	9.0	1.1	9.3	1.1	7.9
11	9.5	8.9	N/A	8.5	1.0	8.3	1.1	9.0	1.1	9.0	1.1	10.4	1.1	7.4	1.1	9.3	1.1	7.5	1.1	8.7
12	10	9.3	N/A	9.2	1.1	9.1	1.1	9.3	1.1	9.5	1.1	10.5	1.1	8.2	1.1	10.1	1.1	9.5	1.1	9.4
13	10.5	12.0	N/A	8.0	1.1	10.5	1.1	8.1	1.1	9.4	1.1	9.7	1.1	7.4	1.1	10.3	1.1	7.9	1.1	9.3
14	11	8.8	N/A	10.6	1.1	9.6	1.1	9.8	1.1	9.6	1.1	7.9	1.1	11.3	1.1	8.8	1.1	10.0	1.1	9.6
15	11.5	8.6	N/A	11.2	1.1	8.2	1.1	10.2	1.1	8.7	1.1	10.2	1.1	10.3	1.1	9.6	1.1	10.4	1.1	9.7
16	12	7.8	N/A	10.9	1.1	8.3	1.1	10.7	1.1	8.2	1.1	9.9	1.1	9.8	1.1	9.6	1.1	11.6	1.1	9.7

Table S76. Binding conditions for surfaces printed under conditions shown in **Table S13**.

TPO (mM)	PETT (mM)	EGDMA (mM)	Intensity (mW/mm^2)	[**SCR043**] (µM)	[α-**Man-FL**] (M)
1	100	1300	2.53	250	$10^{-5.5}$

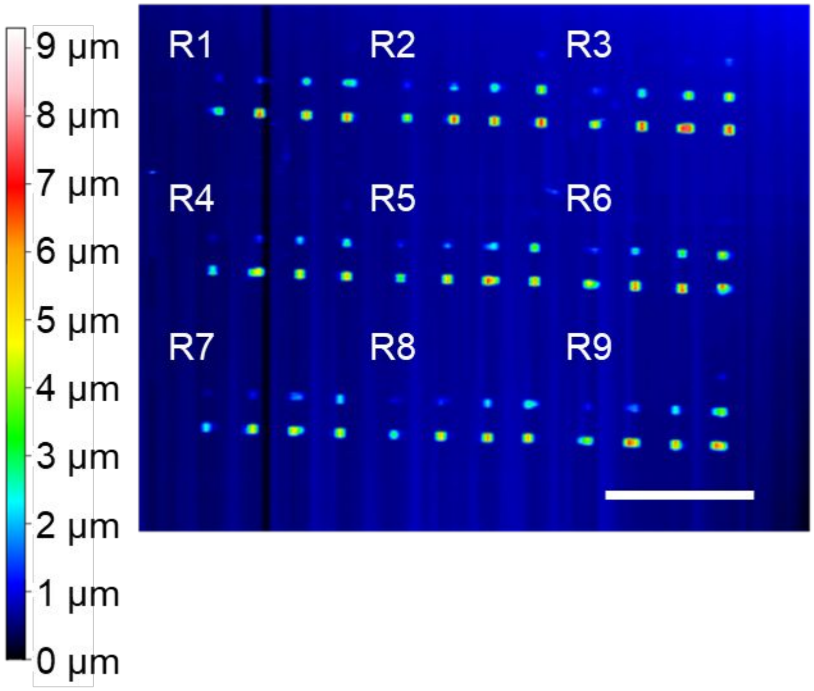

Figure S151. Profilometry image of patterns printed using [TPO] = 100 mM, [EGDMA] = 1300 mM. The pattern printed is 9 repeats (R1-R9) of 16 different time points. The scale bar is 200 µm.

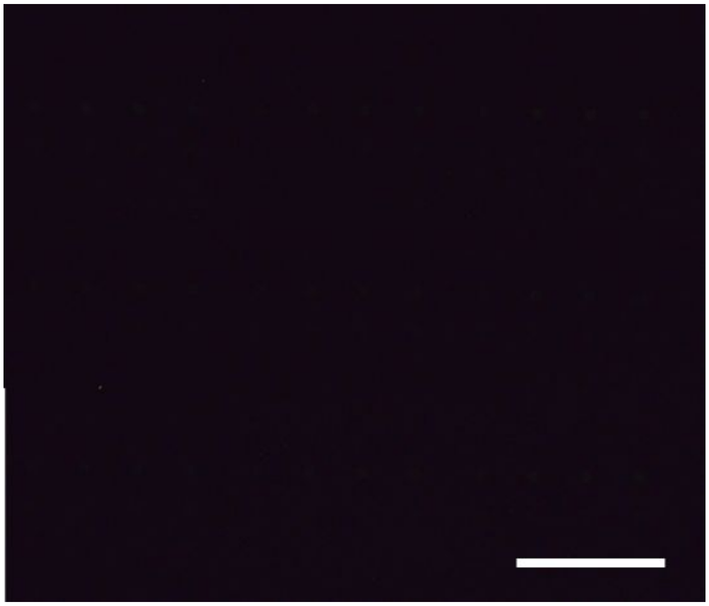

Figure S152. Fluorescence image of patterns printed using [TPO] = 100 mM, [EGDMA] = 1300 mM. Incubation was performed for 1 hour. The pattern printed is 9 repeats (R1-R9) of 16 different time points. The scale bar is 200 μm.

Table S77. Fluorescence intensity (*I*) and heights (*h*) of features printed under conditions in **Table S76**, and shown in **Figure S151** and **Figure S152**, # = feature number.

#	Time (min)	R1	*I*	R2	*I*	R3	*I*	R4	*I*	R5	*I*	R6	*I*	R7	*I*	R8	*I*	R9	*I*	Average Height (μm)
1	4.5	N/A	N/A	N/A	N/A	N/A	N/A	N/A	N/A	N/A	N/A	N/A	N/A	N/A	N/A	N/A	N/A	N/A	N/A	N/A
2	5	N/A	N/A	N/A	N/A	N/A	N/A	N/A	N/A	N/A	N/A	N/A	N/A	N/A	N/A	N/A	N/A	N/A	N/A	N/A
3	5.5	N/A	N/A	N/A	N/A	N/A	N/A	N/A	N/A	N/A	N/A	N/A	N/A	N/A	N/A	N/A	N/A	N/A	N/A	N/A
4	6	N/A	N/A	N/A	N/A	N/A	N/A	N/A	N/A	N/A	N/A	N/A	N/A	N/A	N/A	N/A	N/A	N/A	N/A	N/A
5	6.5	N/A	N/A	N/A	N/A	N/A	N/A	N/A	N/A	N/A	N/A	N/A	N/A	N/A	N/A	N/A	N/A	N/A	N/A	N/A
6	7	N/A	N/A	N/A	N/A	N/A	N/A	N/A	N/A	N/A	N/A	N/A	N/A	N/A	N/A	N/A	N/A	N/A	N/A	N/A
7	7.5	N/A	N/A	N/A	N/A	N/A	N/A	N/A	N/A	N/A	N/A	N/A	N/A	N/A	N/A	N/A	N/A	N/A	N/A	N/A
8	8	N/A	N/A	N/A	N/A	N/A	N/A	N/A	N/A	N/A	N/A	N/A	N/A	N/A	N/A	N/A	N/A	N/A	N/A	N/A
9	8.5	0.5	N/A	0.6	N/A	0.5	N/A	0.4	N/A	0.3	N/A	0.6	N/A	N/A	N/A	N/A	N/A	N/A	N/A	0.5
10	9	0.9	N/A	2.1	N/A	2.0	N/A	0.6	N/A	0.8	N/A	2.2	N/A	0.8	N/A	0.5	N/A	0.6	N/A	1.2
11	9.5	2.3	N/A	2.1	N/A	3.6	N/A	2.1	N/A	1.8	N/A	2.4	N/A	1.3	N/A	1.6	N/A	2.2	N/A	2.2
12	10	2.3	N/A	3.7	N/A	4.6	N/A	2.0	N/A	3.0	N/A	4.2	N/A	1.7	N/A	2.1	N/A	3.6	N/A	3.0
13	10.5	2.6	N/A	3.6	1.0	5.2	1.0	2.2	1.0	3.5	1.0	4.1	1.0	2.0	1.0	1.8	1.0	3.7	1.0	3.2
14	11	5.4	N/A	6.4	1.0	6.3	1.0	4.2	1.0	5.4	1.0	5.6	1.0	4.7	1.0	4.6	1.0	5.9	1.0	5.4
15	11.5	5.3	N/A	6.1	1.0	6.4	1.0	4.4	1.0	5.0	1.0	6.0	1.0	4.6	1.0	5.1	1.0	6.0	1.0	5.4
16	12	6.1	N/A	6.5	1.0	6.6	1.0	5.2	1.0	5.1	1.0	6.1	1.0	4.0	1.0	5.1	1.0	5.6	1.0	5.6

Table S78. Binding conditions for surfaces printed under conditions shown in **Table S13**.

TPO (mM)	PETT (mM)	EGDMA (mM)	Intensity (mW/mm^2)	[**SCR043**] (µM)	[α-**Man-FL**] (M)
1	100	1300	2.53	12.5	$10^{-5.5}$

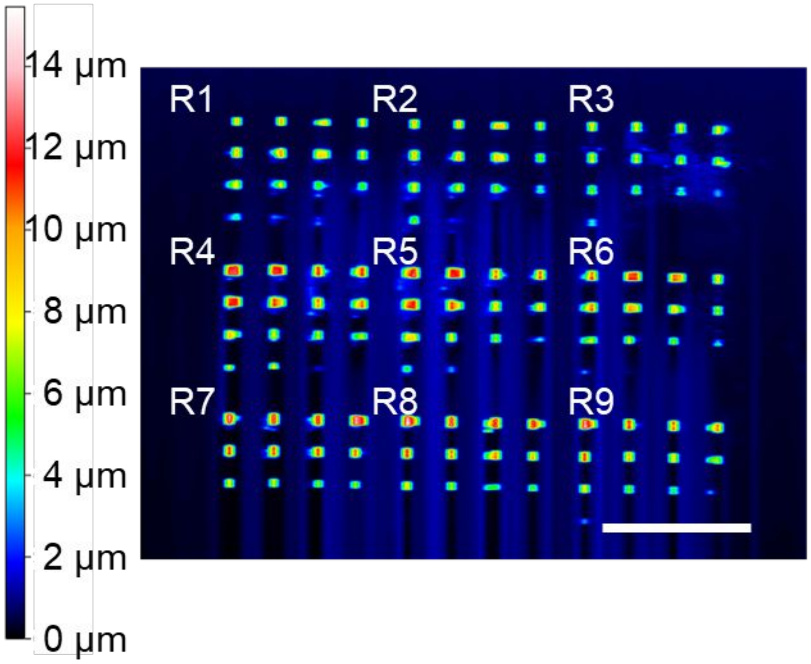

Figure S153. Profilometry image of patterns printed using [TPO] = 100 mM, [EGDMA] = 1300 mM. The pattern printed is 9 repeats (R1-R9) of 16 different time points. The scale bar is 200 µm.

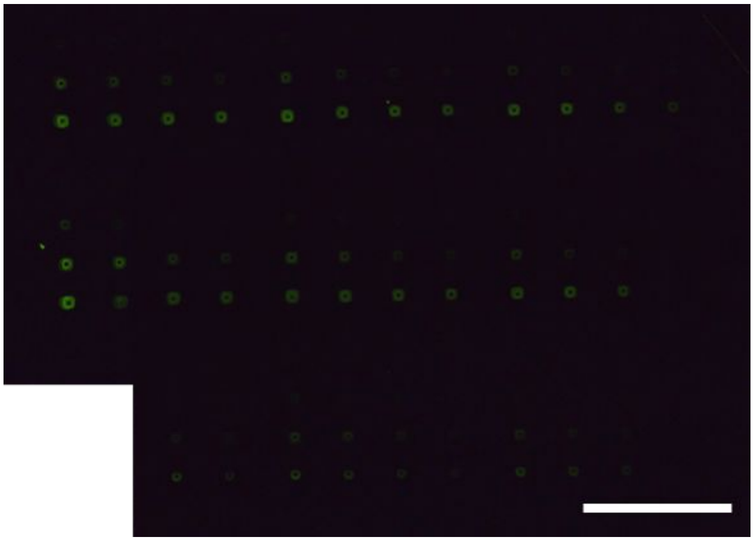

Figure S154. Fluorescence image of patterns printed using [TPO] :100 mM, [EGDMA]: 1300 mM. Incubation was performed for 1 hour. The pattern printed is 9 repeats (R1-R9) of 16 different time points. The scale bar is 200 µm.

Table S79. Fluorescence intensity (*I*) and heights (*h*) of features printed under conditions in **Table S78**, and shown in **Figure S153** and **Figure S154**, # = feature number.

#	Time (min)	R1	*I*	R2	*I*	R3	*I*	R4	*I*	R5	*I*	R6	*I*	R7	*I*	R8	*I*	R9	*I*	Average Height (µm)
1	3.4	N/A	N/A	N/A	N/A	N/A	N/A	N/A	N/A	N/A	N/A	N/A	N/A	N/A	N/A	N/A	N/A	N/A	N/A	N/A
2	3.9	2.2	N/A	N/A	N/A	N/A	N/A	N/A	N/A	N/A	N/A	N/A	N/A	N/A	N/A	N/A	N/A	N/A	N/A	2.2
3	4.4	2.5	N/A	1.9	N/A	N/A	N/A	7.7	N/A	3.0	N/A	N/A	N/A	N/A	N/A	N/A	N/A	N/A	N/A	3.8
4	4.9	4.6	N/A	6.3	N/A	3.8	N/A	4.1	N/A	5.4	N/A	2.5	N/A	N/A	N/A	N/A	N/A	N/A	N/A	4.5
5	5.4	6.0	1.0	4.0	N/A	3.4	N/A	5.7	1.0	1.6	1.0	2.2	1.0	6.8	N/A	6.2	1.0	3.0	1.0	4.3
6	5.9	5.9	1.0	6.7	1.0	4.4	N/A	7.5	1.0	6.7	1.0	5.9	1.0	6.5	N/A	5.8	1.0	5.1	1.0	6.1
7	6.4	7.7	1.0	7.5	1.0	6.2	N/A	7.8	1.0	8.3	1.0	7.2	1.0	7.3	N/A	7.0	1.0	6.8	1.0	7.3
8	6.9	7.9	1.0	7.8	1.0	6.0	N/A	7.8	1.0	7.5	N/A	5.7	1.0	7.9	N/A	8.5	N/A	8.5	1.0	7.5
9	7.4	8.0	1.0	7.2	1.0	6.8	1.0	8.1	1.1	5.2	1.0	2.8	1.0	10.6	1.1	9.2	1.0	6.6	1.0	7.2
10	7.9	8.1	1.1	9.1	1.0	6.2	1.0	9.6	1.2	7.4	1.1	6.1	1.0	11.3	1.1	10.2	1.1	7.9	1.0	8.4
11	8.4	10.1	1.1	9.3	1.1	7.2	1.1	9.5	1.3	9.1	1.2	8.3	1.1	11.1	1.2	9.7	1.1	9.0	1.0	9.2
12	8.9	9.4	1.2	9.1	1.1	7.1	1.1	8.8	1.5	10.3	1.5	9.3	1.2	11.6	1.3	11.5	1.2	11.0	1.1	9.8
13	9.4	7.9	1.1	7.1	1.0	8.2	1.0	8.8	1.4	6.9	1.3	9.0	1.1	13.9	1.4	12.1	1.3	10.4	1.1	9.4
14	9.9	9.3	1.1	9.2	1.1	6.9	1.1	9.8	1.5	7.7	1.4	6.8	1.3	12.7	1.5	12.3	1.5	10.1	1.2	9.4
15	10.4	9.7	1.1	9.0	1.1	7.8	1.1	7.8	1.3	6.7	1.5	4.7	1.4	13.1	1.5	13.7	1.5	11.5	1.4	9.3
16	10.9	8.8	1.2	8.7	1.1	7.6	1.2	11.5	1.7	11.2	1.7	7.2	1.4	13.0	1.8	14.8	1.6	14.0	1.5	10.8

Table S80. Binding conditions for surfaces printed under conditions shown in **Table S13**.

TPO (mM)	PETT (mM)	EGDMA (mM)	Intensity (mW/mm^2)	[**SCR043**] (μM)	[α-**Man-FL**] (M)
1	100	1300	2.53	0	$10^{-5.5}$

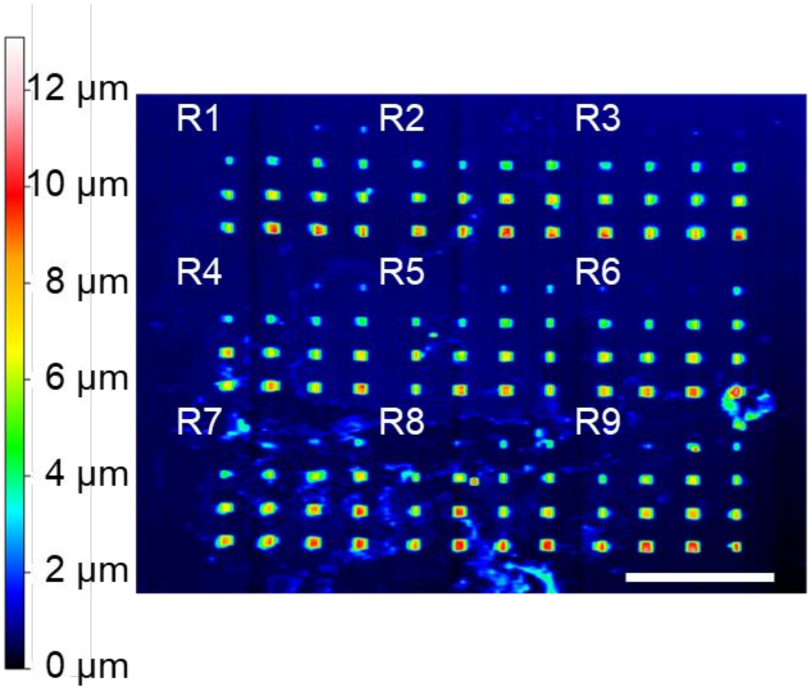

Figure S155. Profilometry image of patterns printed using [TPO] = 100 mM, [EGDMA] = 1300 mM. The pattern printed is 9 repeats (R1-R9) of 16 different time points. The scale bar is 200 μm.

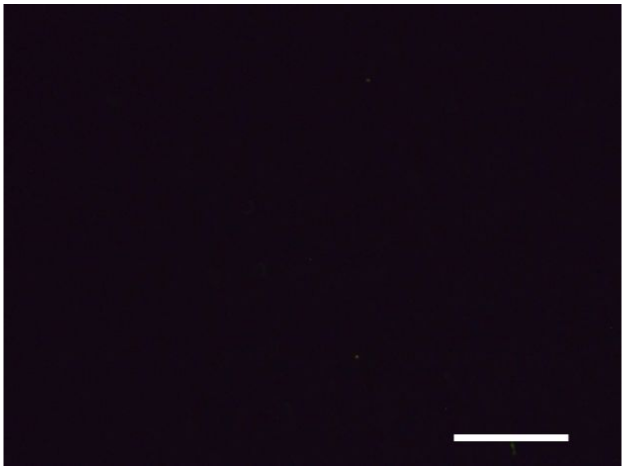

Figure S156. Fluorescence image of patterns printed using [TPO] = 100 mM, [EGDMA] = 1300 mM. Incubation was performed for 1 hour. The pattern printed is 9 repeats (R1-R9) of 16 different time points. The scale bar is 200 µm.

Table S81. Binding conditions for surfaces printed under conditions shown in **Table S13**.

TPO (mM)	PETT (mM)	EGDMA (mM)	Intensity (mW/mm^2)	[**SCR043**] (μM)	[α-**Man-FL**] (M)
1	100	1300	2.53	500	10^{-6}

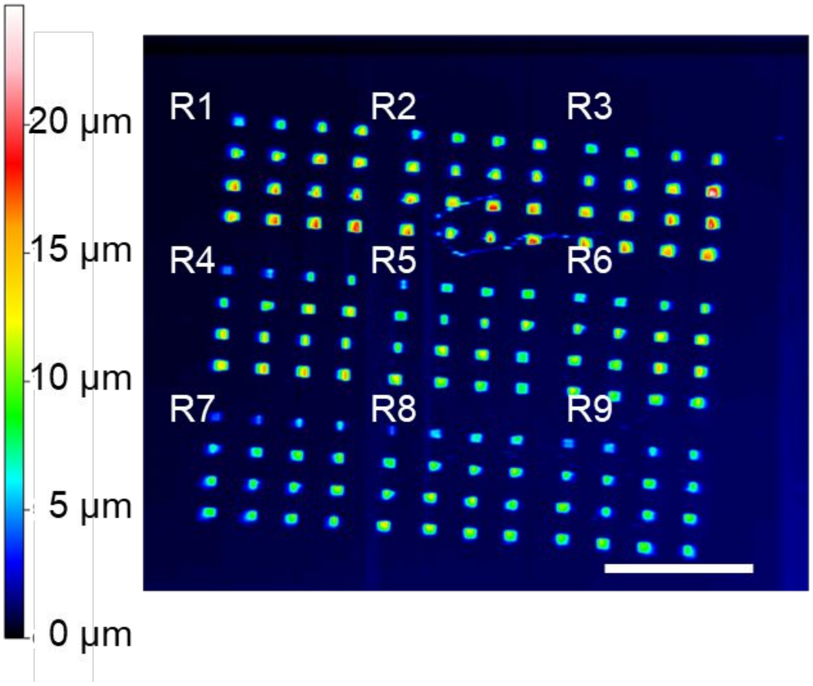

Figure S157. Profilometry image of patterns printed using [TPO] = 100 mM, [EGDMA] = 1300 mM. The pattern printed is 9 repeats (R1-R9) of 16 different time points. The scale bar is 200 μm.

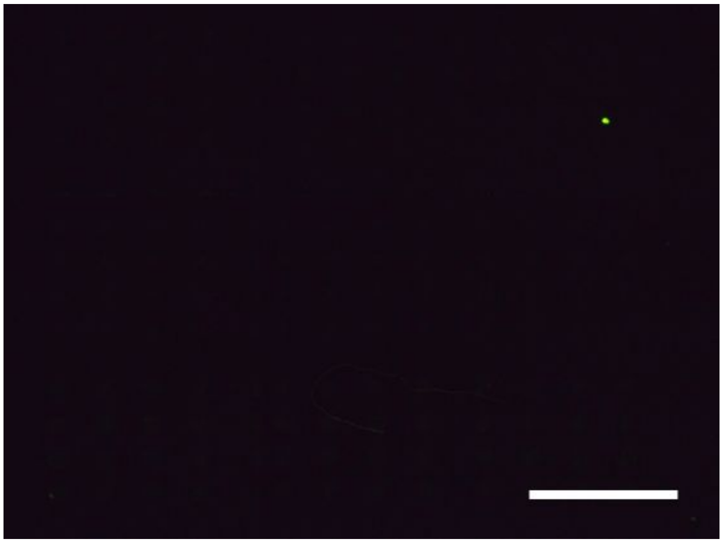

Figure S158. Fluorescence image of patterns printed using [TPO] = 100 mM, [EGDMA] = 1300 mM. Incubation was performed for 1 hour. The pattern printed is 9 repeats (R1-R9) of 16 different time points. The scale bar is 200 μm.

Table S82. Binding conditions for surfaces printed under conditions shown in **Table S13**.

TPO (mM)	PETT (mM)	EGDMA (mM)	Intensity (mW/mm2)	[**SCR043**] (μM)	[α-**Man-FL**] (M)
1	100	1300	2.53	250	10^{-6}

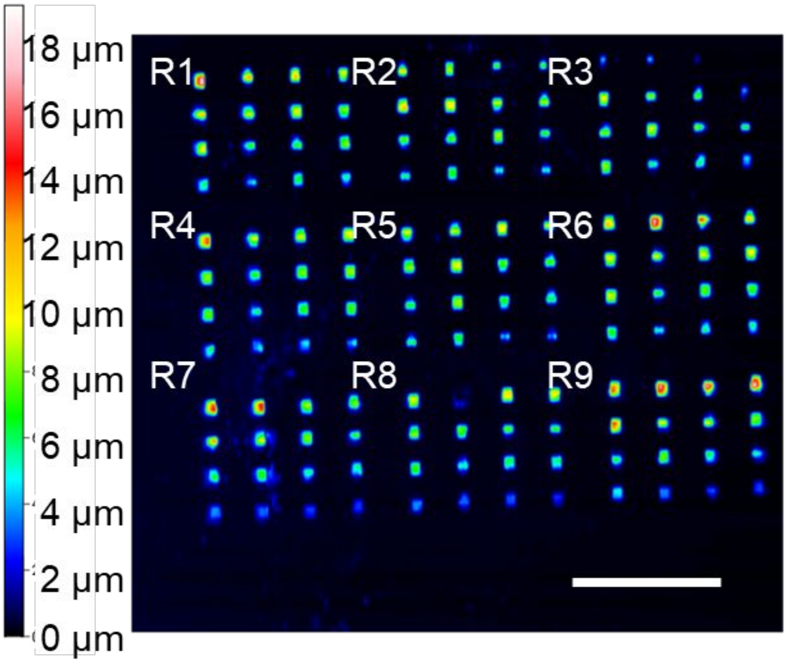

Figure S159. Profilometry image of patterns printed using [TPO] = 100 mM, [EGDMA] = 1300 mM. The pattern printed is 9 repeats (R1-R9) of 16 different time points. The scale bar is 200 μm.

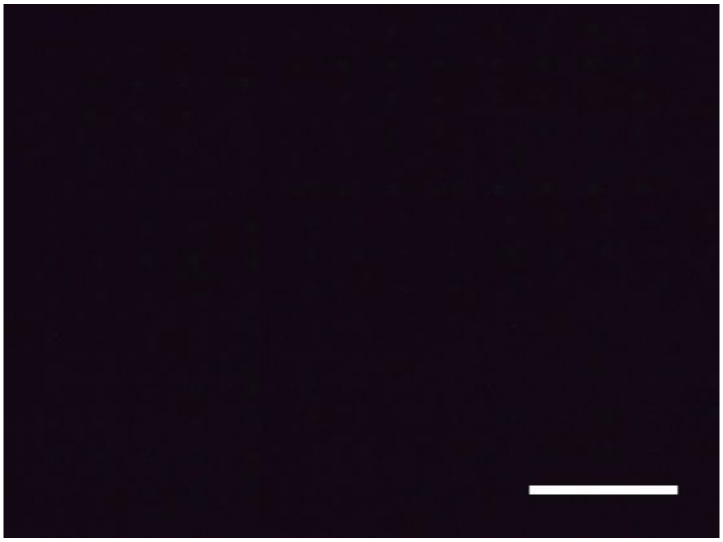

Figure S160. Fluorescence image of patterns printed using [TPO] = 100 mM, [EGDMA] = 1300 mM. Incubation was performed for 1 hour. The pattern printed is 9 repeats (R1-R9) of 16 different time points. The scale bar is 200 μm.

Table S83. Binding conditions for surfaces printed under conditions shown in **Table S13**.

TPO (mM)	PETT (mM)	EGDMA (mM)	Intensity (mW/mm2)	[**SCR043**] (µM)	[α-**Man-FL**] (M)
1	100	1300	2.53	12.5	10^{-6}

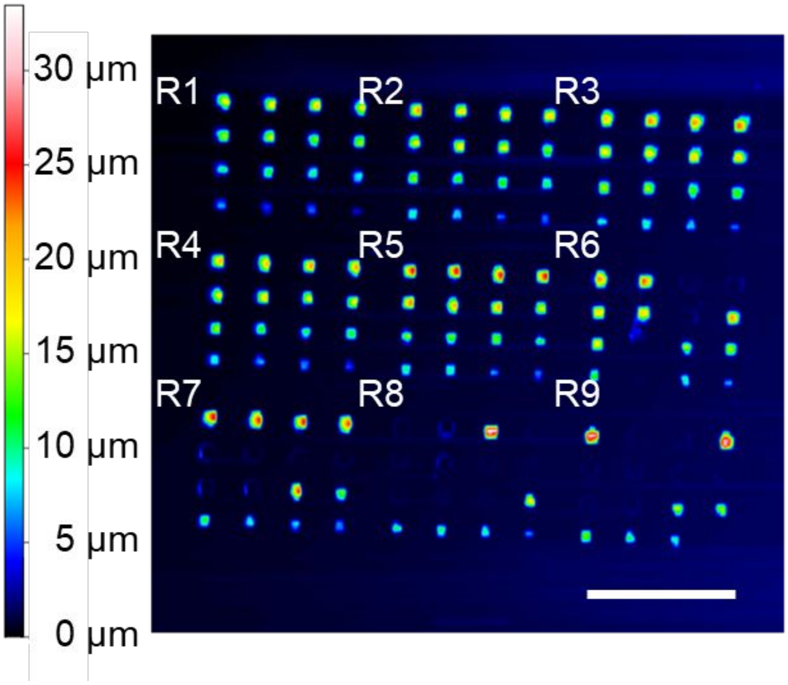

Figure S161. Profilometry image of patterns printed using [TPO] = 100 mM, [EGDMA] = 1300 mM. The pattern printed is 9 repeats (R1-R9) of 16 different time points. The scale bar is 200 µm.

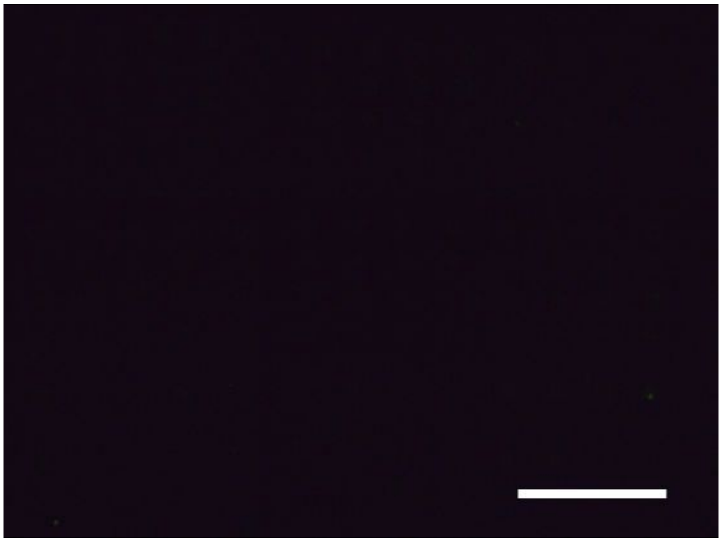

Figure S162. Fluorescence image of patterns printed using [TPO] = 100 mM, [EGDMA] = 1300 mM. Incubation was performed for 1 hour. The pattern printed is 9 repeats (R1-R9) of 16 different time points. The scale bar is 200 µm.

Table S84. Binding conditions for surfaces printed under conditions shown in **Table S13**.

TPO (mM)	PETT (mM)	EGDMA (mM)	Intensity (mW/mm^2)	[**SCR043**] (μM)	[α-**Man-FL**] (M)
1	100	1300	2.53	0	10^{-6}

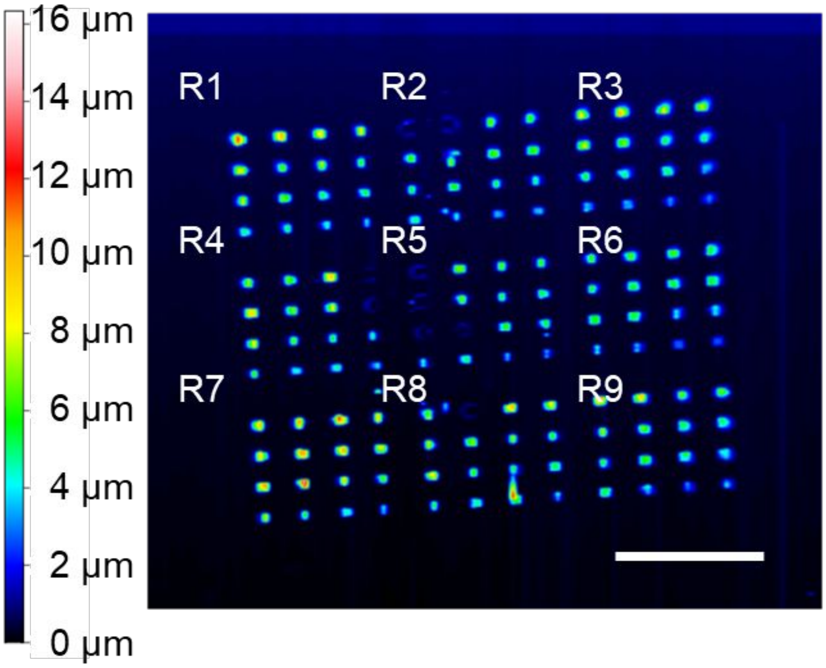

Figure S163. Profilometry image of patterns printed using [TPO] = 100 mM, [EGDMA] = 1300 mM. The pattern printed is 9 repeats (R1-R9) of 16 different time points. The scale bar is 200 μm.

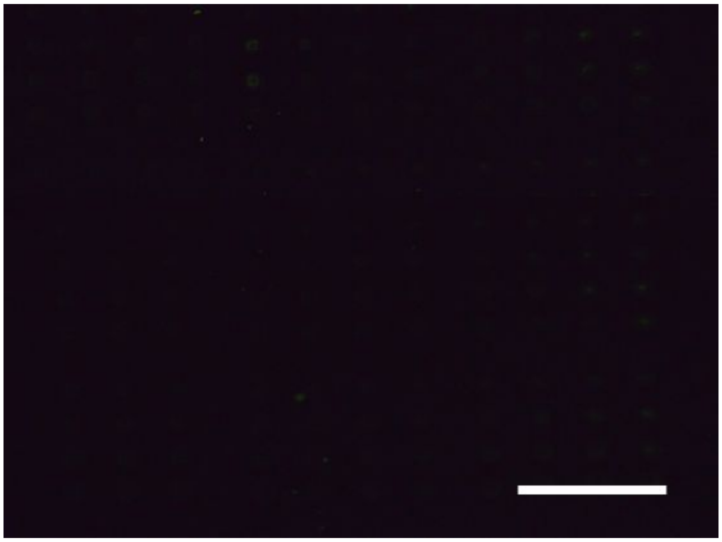

Figure S164. Fluorescence image of patterns printed using [TPO] = 100 mM, [EGDMA] = 1300 mM. Incubation was performed for 1 hour. The pattern printed is 9 repeats (R1-R9) of 16 different time points. The scale bar is 200 μm.

Table S85. Binding conditions for surfaces printed under conditions shown in **Table S13**.

TPO (mM)	PETT (mM)	EGDMA (mM)	Intensity (mW/mm^2)	[**SCR043**] (μM)	[**FL**] (M)
1	100	1300	2.53	500	10^{-3}

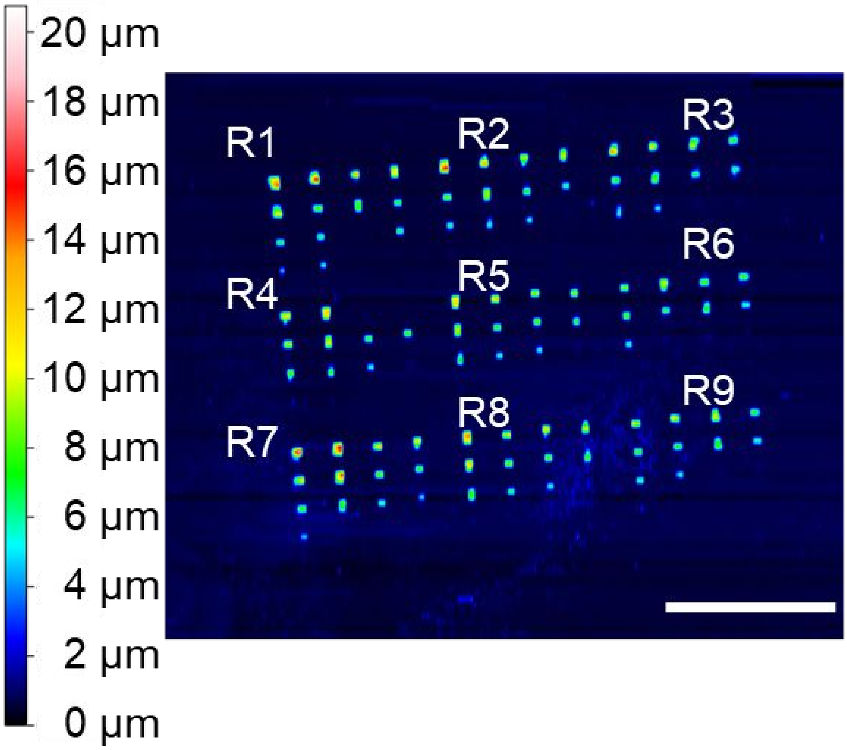

Figure S165. Profilometry image of patterns printed using [TPO] = 100 mM, [EGDMA] = 1300 mM. The pattern printed is 9 repeats (R1-R9) of 16 different time points. The scale bar is 200 µm.

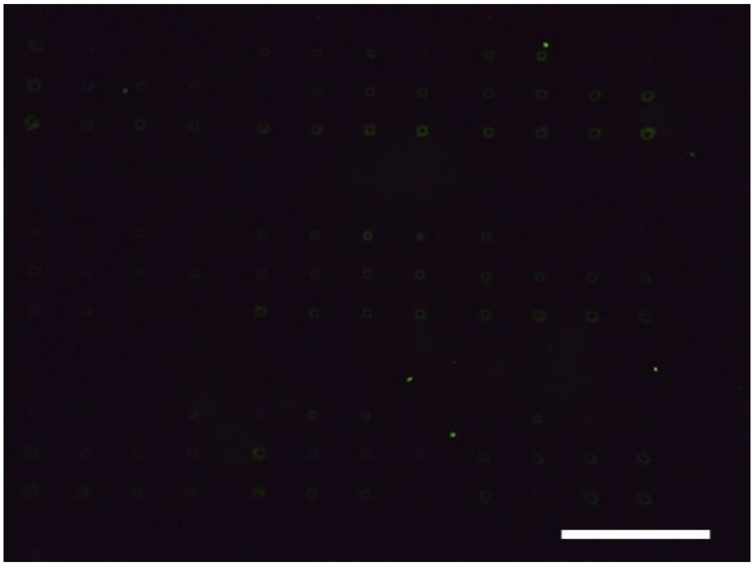

Figure S166. Fluorescence image of patterns printed using [TPO] = 100 mM, [EGDMA] = 1300 mM. Incubation was performed for 1 hour. The pattern printed is 9 repeats (R1-R9) of 16 different time points. The scale bar is 200 μm.

Table S86. Fluorescence intensity (*I*) and heights (*h*) of features printed under conditions in **Table S85**, and shown in **Figure S165** and **Figure S166**, # = feature number.

#	Time (min)	R1	*I*	R2	*I*	R3	*I*	R4	*I*	R5	*I*	R6	*I*	R7	*I*	R8	*I*	R9	*I*	Average Height (μm)
5	6.5	2.8	1.0	2.9	1.0	2.3	1.1	2.6	1.0	2.3	1.1	N/A	1.1	2.1	1.0	0.9	1.0	N/A	1.0	2.3
6	7.0	3.0	1.0	3.1	1.0	2.5	1.2	3.1	1.0	2.5	1.1	N/A	1.0	2.4	1.0	0.6	1.1	N/A	1.1	2.5
7	7.5	3.6	1.0	3.6	1.1	2.6	1.0	3.4	1.0	2.5	1.2	N/A	1.0	2.4	1.0	0.9	1.0	0.6	1.0	2.5
8	8.0	4.6	1.0	4.3	1.0	2.3	1.0	3.9	1.0	2.3	1.1	0.5	1.0	2.4	1.1	2.1	1.0	1.4	1.0	2.6
9	8.5	4.4	1.1	4.4	1.0	8.5	1.1	4.2	1.0	5.3	1.1	2.6	1.1	3.8	1.0	2.8	1.2	1.6	1.0	4.2
10	9.0	4.4	1.0	5.3	1.0	10.9	1.1	4.4	1.0	5.7	1.1	2.9	1.1	5.2	1.0	2.5	1.0	2.0	1.1	4.8
11	9.5	5.0	1.0	5.9	1.1	11.4	1.1	5.0	1.0	5.2	1.1	2.7	1.1	5.0	1.0	3.2	1.0	2.2	1.1	5.1
12	10.0	6.5	1.0	6.3	1.1	11.8	1.2	6.3	1.0	6.0	1.1	2.6	1.1	4.6	1.0	3.8	1.0	3.0	1.1	5.6
13	10.5	5.4	1.2	9.4	1.1	9.9	1.1	5.0	1.0	8.5	1.2	7.0	1.1	6.5	1.1	5.2	1.1	3.4	1.0	6.7
14	11.0	5.2	1.0	6.9	1.1	11.8	1.1	5.5	1.0	7.5	1.1	6.3	1.2	5.4	1.1	6.4	1.1	3.4	1.0	6.5
15	11.5	5.9	1.1	7.1	1.2	16.2	1.1	5.7	1.0	7.9	1.1	5.3	1.1	6.6	1.1	5.6	1.1	5.1	1.1	7.3
16	12.0	6.0	1.0	10.3	1.3	14.7	1.3	5.7	1.0	7.9	1.2	6.8	1.1	7.9	1.0	6.3	1.0	5.4	1.1	7.9

Table S87. Binding conditions for surfaces printed under conditions shown in **Table S13**.

TPO (mM)	PETT (mM)	EGDMA (mM)	Intensity (mW/mm^2)	[**SCR043**] (μM)	[**FL**] (M)
1	100	1300	2.53	500	10^{-4}

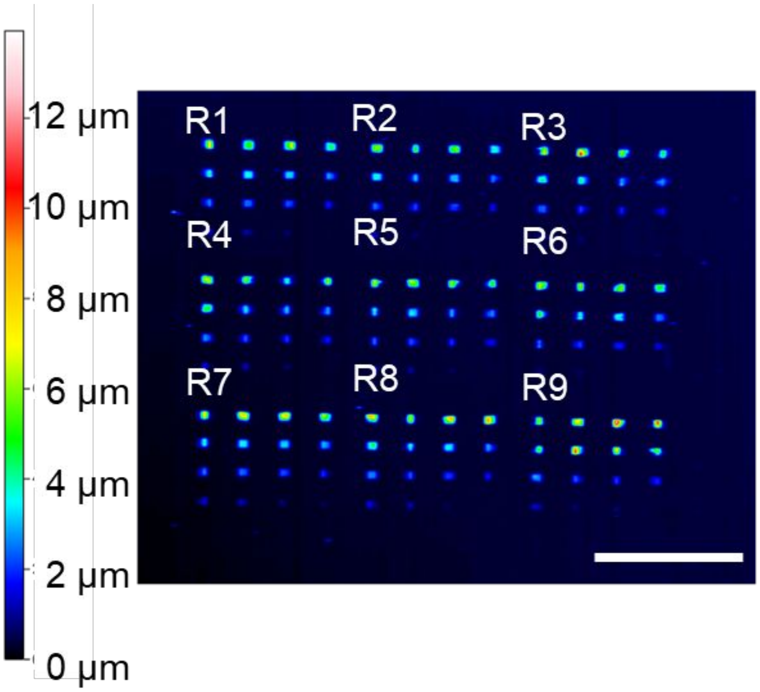

Figure S167. Profilometry image of patterns printed using [TPO] = 100 mM, [EGDMA] = 1300 mM. The pattern printed is 9 repeats (R1-R9) of 16 different time points. The scale bar is 200 μm.

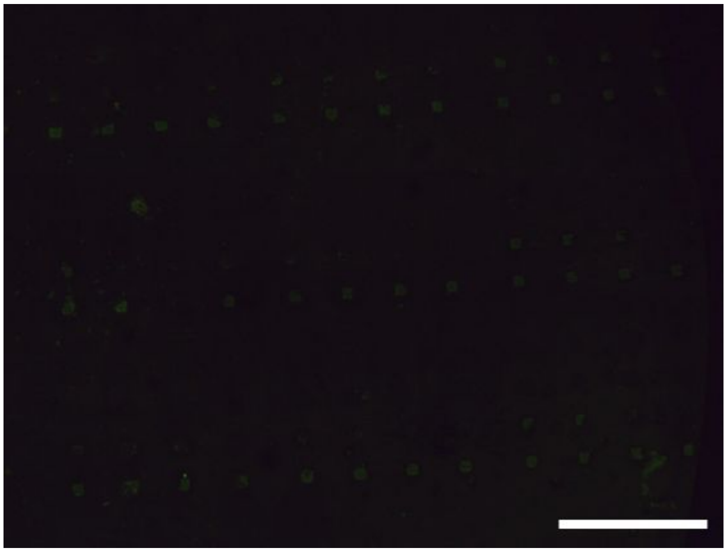

Figure S168. Fluorescence image of patterns printed using [TPO] = 100 mM, [EGDMA] = 1300 mM. Incubation was performed for 1 hour. The pattern printed is 9 repeats (R1-R9) of 16 different time points. The scale bar is 200 µm.

Table S88. Fluorescence intensity (*I*) and heights (*h*) of features printed under conditions in **Table S87**, and shown in **Figure S167** and **Figure S168**, # = feature number.

#	Time (min)	R1	*I*	R2	*I*	R3	*I*	R4	*I*	R5	*I*	R6	*I*	R7	*I*	R8	*I*	R9	*I*	Average Height (µm)
5	6.5	1.5	1.0	1.3	1.0	1.3	1.0	1.5	1.0	1.4	1.0	1.1	0.9	1.7	1.0	1.4	1.0	1.5	0.9	1.4
6	7.0	1.9	1.0	1.9	1.0	1.5	1.0	1.8	1.0	1.7	1.0	1.4	1.0	2.0	1.0	1.7	1.0	1.7	1.0	1.7
7	7.5	2.1	1.0	1.8	1.0	1.7	1.0	2.1	1.0	2.0	1.0	1.7	1.0	2.3	1.0	2.1	1.0	2.0	1.0	2.0
8	8.0	2.3	1.0	2.2	1.0	2.0	1.0	2.5	1.0	2.3	1.0	2.1	1.0	2.4	1.0	2.3	1.0	2.8	1.0	2.3
9	8.5	2.5	1.0	2.2	1.0	2.4	1.0	2.5	1.0	2.3	1.0	2.1	1.0	2.8	1.0	2.5	1.0	5.6	1.0	2.8
10	9.0	2.9	1.0	2.6	1.0	2.4	1.1	2.9	1.0	2.6	1.0	3.5	1.0	2.9	1.0	3.4	1.0	7.5	1.0	3.4
11	9.5	3.2	1.0	2.8	1.0	3.9	1.1	3.1	1.0	3.3	1.0	3.5	1.0	3.2	1.0	3.7	1.0	10.0	1.0	4.1
12	10.0	3.7	1.0	3.4	1.0	3.9	1.1	4.6	1.1	3.9	1.0	4.4	1.1	3.6	1.0	4.5	1.0	5.4	1.0	4.2
13	10.5	3.8	1.1	3.5	1.1	4.4	1.1	6.0	1.1	5.0	1.1	5.1	1.0	6.2	1.1	8.9	1.1	11.2	1.0	6.0
14	11.0	6.4	1.1	5.3	1.1	5.8	1.1	3.8	1.0	5.9	1.1	5.6	1.1	8.2	1.1	8.7	1.1	13.1	1.1	7.0
15	11.5	4.9	1.1	4.7	1.1	10.7	1.1	6.0	1.2	6.3	1.1	6.2	1.1	7.8	1.1	6.2	1.1	6.5	1.1	6.6
16	12.0	7.2	1.1	5.7	1.1	7.1	1.1	6.1	1.2	6.6	1.1	5.9	1.1	7.9	1.2	8.7	1.1	5.9	1.1	6.8

Table S89. Binding conditions for surfaces printed under conditions shown in **Table S13**.

TPO (mM)	PETT (mM)	EGDMA (mM)	Intensity (mW/mm^2)	[**SCR043**] (μM)	[α-**Gal-FL**] (M)
1	100	1300	2.53	100	10^{-3}

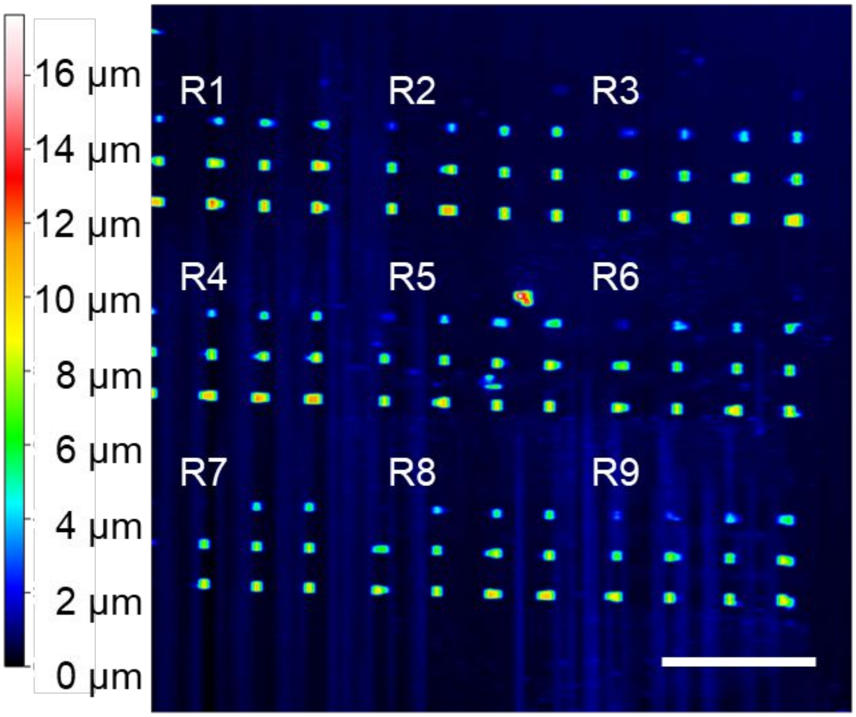

Figure S169. Profilometry image of patterns printed using [TPO] = 100 mM, [EGDMA] = 1300 mM. The pattern printed is 9 repeats (R1-R9) of 16 different time points. The scale bar is 200 μm.

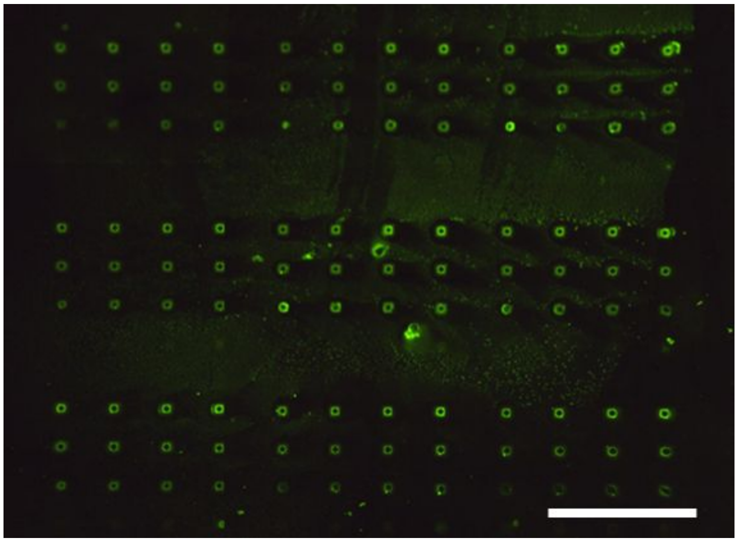

Figure S170. Fluorescence image of patterns printed using [TPO] = 100 mM, [EGDMA] = 1300 mM. Incubation was performed for 1 hour. The pattern printed is 9 repeats (R1-R9) of 16 different time points. The scale bar is 200 μm.

Table S90. Fluorescence intensity (*I*) and heights (*h*) of features printed under conditions in **Table S89**, and shown in **Figure S169** and **Figure S170**, # = feature number.

#	Time (min)	R1	*I*	R2	*I*	R3	*I*	R4	*I*	R5	*I*	R6	*I*	R7	*I*	R8	*I*	R9	*I*	Average Height (μm)
5	6.5	2.4	0.9	N/A	1.2	N/A	1.4	1.0	1.2	1.6	1.3	4.2	1.3	2.2	1.1	2.1	1.0	4.1	0.9	2.5
6	7	2.5	0.9	4.8	1.3	N/A	1.4	4.0	1.2	4.8	1.3	4.9	1.3	3.4	1.1	4.3	1.0	5.1	1.0	4.2
7	7.5	4.6	1.0	5.7	1.3	6.0	1.2	5.0	1.3	4.8	1.3	6.2	1.3	4.8	1.0	6.8	1.0	5.8	1.0	5.5
8	8	5.7	1.1	5.8	1.2	6.1	1.6	6.7	1.4	5.8	1.4	7.2	1.4	5.6	0.9	7.2	1.0	7.0	1.1	6.4
9	8.5	6.5	1.0	6.2	1.2	N/A	1.4	6.8	1.2	7.1	1.2	7.7	1.2	7.5	1.2	7.2	1.1	8.6	1.1	7.2
10	9	7.1	1.1	7.0	1.2	7.7	1.3	6.6	1.2	8.0	1.2	8.2	1.2	7.7	1.1	8.4	1.1	8.8	1.1	7.7
11	9.5	7.2	1.1	8.3	1.2	8.6	1.3	7.6	1.2	8.9	1.3	8.3	1.3	8.3	1.1	8.8	1.1	9.7	1.1	8.4
12	10	8.4	1.1	8.8	1.2	8.7	1.3	7.4	1.3	9.4	1.3	9.2	1.3	8.0	1.1	9.0	1.1	10.5	1.2	8.8
13	10.5	9.3	1.1	9.2	1.3	N/A	2.1	9.0	1.4	8.8	1.5	9.5	1.6	8.1	1.5	10.0	1.4	10.5	1.3	9.3
14	11	8.3	1.2	9.6	1.3	9.3	1.6	8.2	1.5	10.0	1.5	10.1	1.4	9.0	1.3	10.9	1.3	12.6	1.3	9.8
15	11.5	8.9	1.2	10.8	1.4	10.2	1.5	9.7	1.4	10.0	1.5	11.5	1.6	9.6	1.3	10.6	1.4	11.1	1.3	10.3
16	12	9.4	1.2	9.7	1.4	10.2	1.3	9.7	1.4	10.5	1.4	11.0	1.5	9.1	1.3	10.1	1.4	11.3	1.5	10.1

Table S91**.** Binding conditions for surfaces printed under conditions shown in **Table S13**.

TPO (mM)	PETT (mM)	EGDMA (mM)	Intensity (mW/mm^2)	[**SCR043**] (µM)	[**α-Gal-FL**] (M)
1	100	1300	2.53	100	10^{-4}

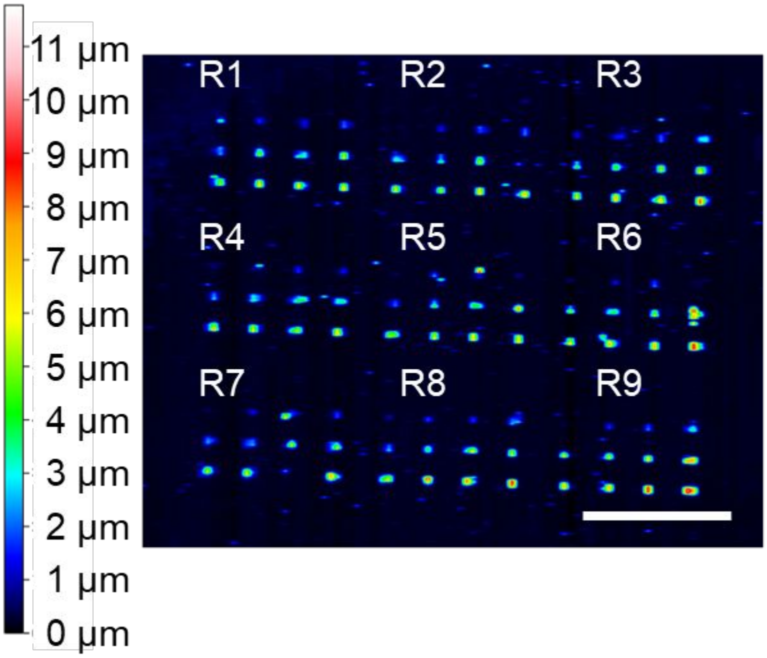

Figure S171. Profilometry image of patterns printed using [TPO] = 100 mM, [EGDMA] = 1300 mM. The pattern printed is 9 repeats (R1-R9) of 16 different time points. The scale bar is 200 µm.

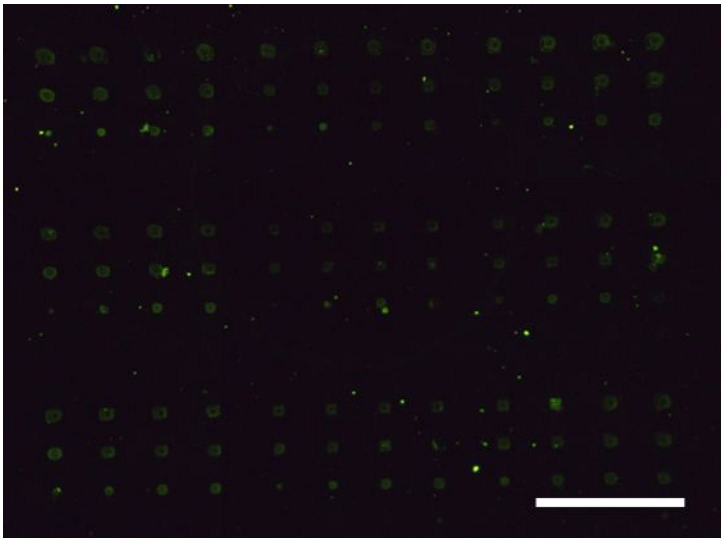

Figure S172. Fluorescence image of patterns printed using [TPO] = 100 mM, [EGDMA] = 1300 mM. Incubation was performed for 1 hour. The pattern printed is 9 repeats (R1-R9) of 16 different time points. The scale bar is 200 µm.

Table S92. Fluorescence intensity (*I*) and heights (*h*) of features printed under conditions in **Table S91**, and shown in **Figure S171** and **Figure S172**, # = feature number.

#	Time (min)	R1	*I*	R2	*I*	R3	*I*	R4	*I*	R5	*I*	R6	*I*	R7	*I*	R8	*I*	R9	*I*	Average Height (µm)
5	6.5	N/A	1.3	0.5	1.1	0.4	1.3	N/A	1.1	N/A	1.1	N/A	1.1	1.2	1.2	0.6	1.2	3.7	1.1	1.3
6	7.0	1.5	1.3	1.6	1.2	1.2	1.2	1.3	1.2	3.4	1.3	4.3	1.2	1.3	1.3	1.3	1.2	1.4	1.2	1.9
7	7.5	2.1	1.4	1.5	1.2	6.1	1.2	1.7	1.3	7.5	1.3	1.4	1.3	1.9	1.2	2.0	1.2	1.3	1.3	2.8
8	8.0	3.6	1.3	2.3	1.1	1.6	1.1	N/A	1.2	N/A	1.1	1.3	1.1	3.0	1.1	1.9	1.2	1.8	1.3	2.2
9	8.5	5.3	1.5	1.9	1.3	2.2	1.4	3.1	1.3	1.6	1.3	2.6	1.4	4.4	1.2	3.2	1.4	2.4	1.3	3.0
10	9.0	5.6	1.4	3.8	1.1	2.4	1.3	2.2	1.3	3.7	1.2	3.2	1.2	4.7	1.3	2.3	1.3	5.1	1.2	3.7
11	9.5	6.1	1.3	5.7	1.1	5.4	1.2	5.3	1.5	3.4	1.3	4.1	1.2	5.6	1.2	5.5	1.3	3.8	1.3	5.0
12	10.0	7.6	1.3	4.9	1.3	5.6	1.2	10.3	1.3	7.7	1.2	3.7	1.2	5.9	1.2	1.8	1.3	5.6	1.2	5.9
13	10.5	6.0	1.4	5.2	1.4	5.4	1.5	6.3	1.3	4.8	1.3	4.7	1.3	5.9	1.2	5.7	1.3	5.3	1.2	5.5
14	11.0	6.3	1.3	10.8	1.3	5.5	1.5	6.9	1.2	5.2	1.2	5.5	1.3	7.6	1.2	5.3	1.3	6.2	1.2	6.6
15	11.5	9.3	1.2	7.1	1.3	2.2	1.5	7.2	1.3	6.2	1.2	4.4	1.3	6.5	1.2	6.8	1.4	5.9	1.2	6.2
16	12.0	9.1	1.4	9.5	1.4	7.1	1.4	9.1	1.2	6.2	1.2	6.8	1.2	7.8	1.2	7.2	1.3	6.6	1.2	7.7

Table S92. Binding conditions for surfaces printed under conditions shown in **Table S13**.

TPO (mM)	PETT (mM)	EGDMA (mM)	Intensity (mW/mm^2)	[**SCR043**] (μM)	[**α-Gluc-FL**] (M)
1	100	1300	2.53	100	10^{-3}

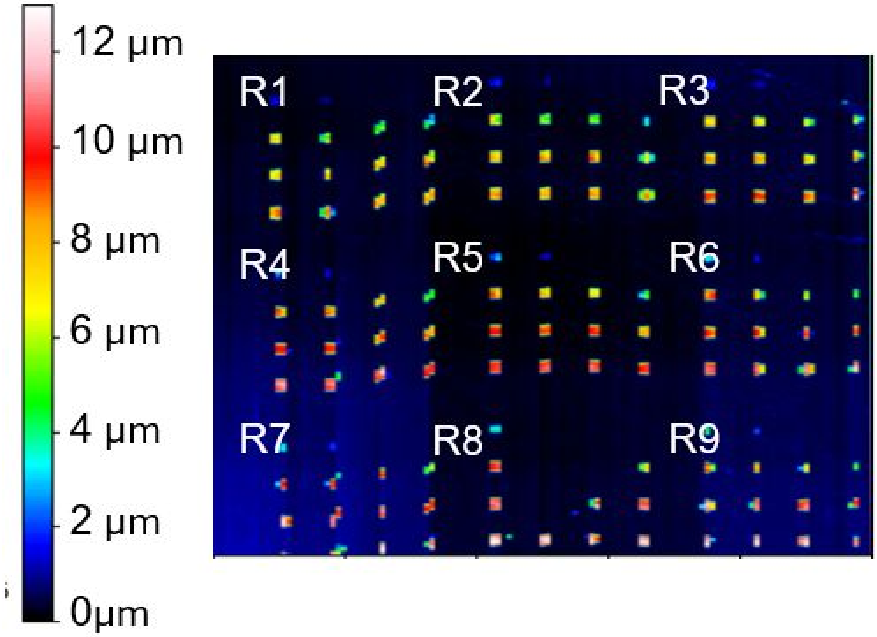

Figure S173. Profilometry image of patterns printed using [TPO] = 100 mM, [EGDMA] = 1300 mM. The pattern printed is 9 repeats (R1-R9) of 16 different time points. The scale bar is 200 μm.

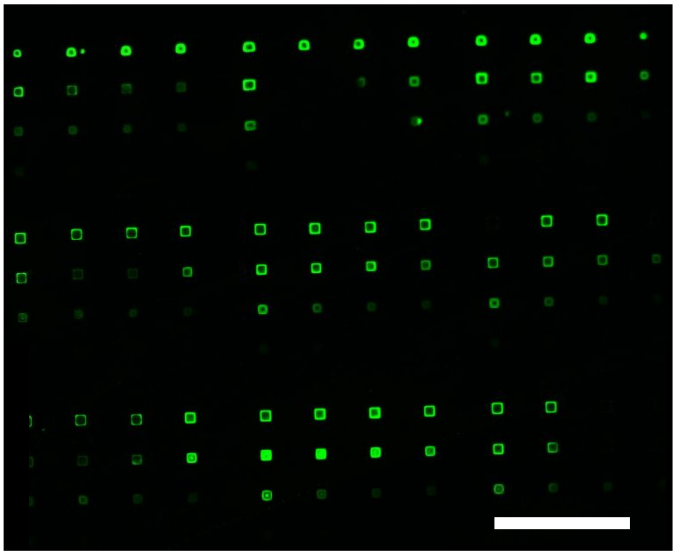

Figure S174. Fluorescence image of patterns printed using [TPO] = 100 mM, [EGDMA] = 1300 mM. Incubation was performed for 1 hour. The pattern printed is 9 repeats (R1-R9) of 16 different time points. The scale bar is 200 µm.

Table S93. Fluorescence intensity (*I*) and heights (*h*) of features printed under conditions in **Table S92**, and shown in **Figure S173** and **Figure S174**, # = feature number.

#	Time (min)	R1	*I*	R2	*I*	R3	*I*	R4	*I*	R5	*I*	R6	*I*	R7	*I*	R8	*I*	R9	*I*	Average Height (µm)
1	3.5	1.1	N/A	3.2	N/A	3.1	1.2	1.6	N/A	3.2	1.8	3.7	N/A	1.4	2.5	3.4	2.2	3.8	2.0	2.7
2	4	0.7	N/A	1.4	N/A	1.4	0.9	0.9	1.0	1.3	1.1	0.1	N/A	0.9	1.2	1.3	0.9	1.6	1.0	1.1
3	4.5	0.1	N/A	0.2	N/A	0.1	0.9	0.1	1.2	0.2	0.9	0.2	N/A	N/A	1.1	0.1	1.1	0.1	1.1	0.1
4	5	0.1	N/A	0.2	N/A	0.1	0.9	0.1	1.1	0.4	1.0	0.2	N/A	N/A	1.2	0.1	1.0	0.1	0.8	0.2
5	5.5	7.7	N/A	9.2	22.6	9.5	13.9	7.5	7.8	9.6	17.2	10.6	14.7	8.5	6.4	9.4	12.6	9.4	13.3	9.0
6	6	8.1	6.2	9.2	18.2	9.6	4.7	6.1	5.5	8.6	7.7	0.4	10.6	7.6	6.2	8.0	1.1	8.3	7.1	7.3
7	6.5	5.5	3.9	8.1	17.1	9.2	2.8	5.4	4.1	7.3	4.2	0.2	6.8	6.4	5.1	6.3	1.3	6.8	4.7	6.1
8	7	4.6	2.9	5.0	12.9	5.8	1.5	3.8	3.2	5.2	2.9	7.4	3.4	5.9	3.6	4.9	12.4	0.1	1.7	4.8
9	7.5	7.7	N/A	9.4	18.3	11.5	21.9	7.9	17.9	9.8	25.4	10.8	9.0	8.2	20.6	9.5	24.4	11.2	29.3	9.6
10	8	7.8	4.9	9.3	16.9	10.5	14.1	8.8	6.3	9.2	23.7	0.1	19.4	8.2	11.2	9.5	1.2	10.4	21.6	8.2
11	8.5	8.0	9.0	9.1	14.9	10.5	1.0	9.6	4.9	10.2	23.5	12.1	16.7	7.5	8.6	9.1	6.1	10.6	28.0	9.6
12	9	9.6	26.1	8.6	10.1	9.9	1.1	7.6	16.2	8.6	13.0	11.1	3.3	8.3	6.8	9.0	16.2	9.7	9.9	9.2
13	9.5	8.7	N/A	10.9	20.2	N/A	23.3	8.7	25.0	10.2	25.9	12.7	1.7	9.1	16.0	10.4	23.3	12.2	27.7	10.4
14	10	8.6	13.7	10.7	18.2	N/A	18.8	8.1	21.9	10.7	30.3	11.8	12.9	9.2	26.3	N/A	28.7	12.1	27.9	N/A
15	10.5	7.9	13.8	11.5	16.8	11.9	1.5	8.3	22.6	10.8	27.3	12.0	12.6	8.7	31.4	10.6	26.1	12.0	27.0	10.4
16	11	8.4	28.0	9.8	11.9	11.7	1.0	8.6	26.8	10.2	24.5	12.0	1.1	0.3	31.3	10.8	30.1	10.6	10.9	9.2

Table S94. Binding conditions for surfaces printed under conditions shown in **Table S13**.

TPO (mM)	PETT (mM)	EGDMA (mM)	Intensity (mW/mm^2)	[**SCR043**] (µM)	[**α-Gluc-FL**] (M)
1	100	1300	2.53	100	$10^{-3.5}$

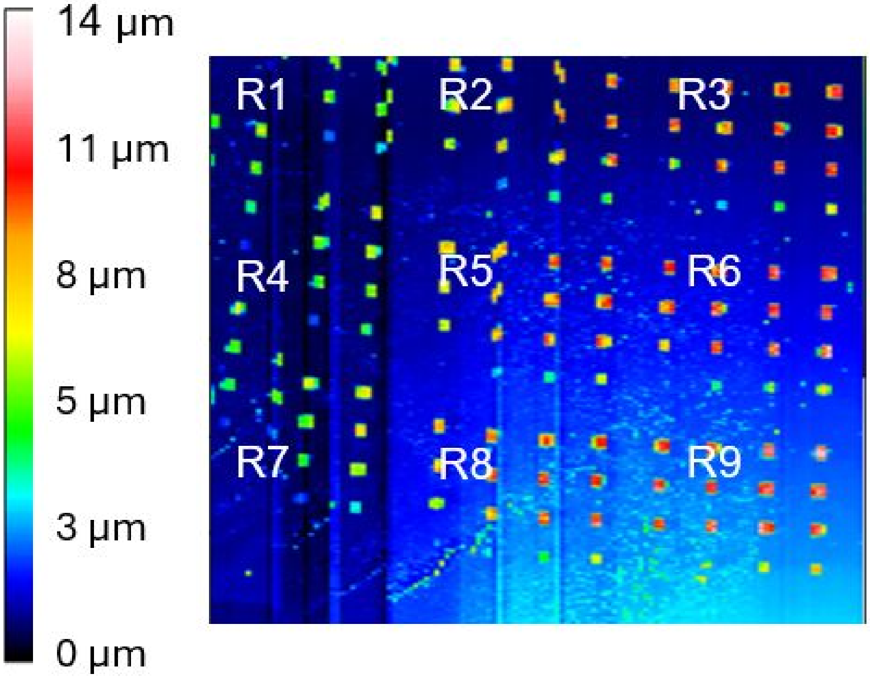

Figure S175. Profilometry image of patterns printed using [TPO] = 100 mM, [EGDMA] = 1300 mM. The pattern printed is 9 repeats (R1-R9) of 16 different time points. The scale bar is 200 µm.

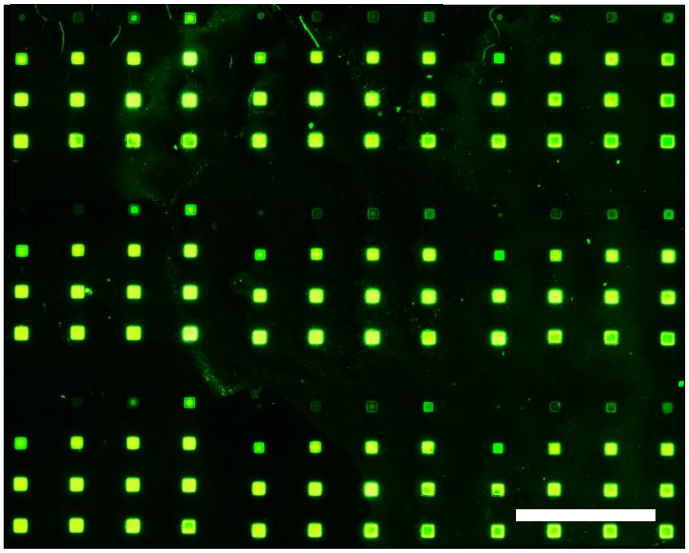

Figure S176. Fluorescence image of patterns printed using [TPO] = 100 mM, [EGDMA] = 1300 mM. Incubation was performed for 1 hour. The pattern printed is 9 repeats (R1-R9) of 16 different time points. The scale bar is 200 μm.

Table S95. Fluorescence intensity (*I*) and heights (*h*) of features printed under conditions in **Table S94**, and shown in **Figure S175** and **Figure S176**, # = feature number.

#	Time (min)	R1	*I*	R2	*I*	R3	*I*	R4	*I*	R5	*I*	R6	*I*	R7	*I*	R8	*I*	R9	*I*	Average Height (μm)
1	3.5	4.8	26.3	5.9	21.4	6.1	14.5	7.6	22.5	8.3	23.6	7.9	21.7	8.9	18.6	9.5	22.1	9.4	20.5	7.6
2	4	5.5	27.8	5.5	20.3	7.1	16.3	8.1	23.1	9.1	24.6	8.3	18.0	9.5	16.0	10.2	21.8	9.6	19.8	8.1
3	4.5	6.9	28.8	6.0	21.1	7.6	16.0	8.3	23.7	9.8	24.8	8.4	16.6	10.1	16.9	11.0	20.8	10.0	19.0	8.7
4	5	7.5	24.6	7.5	17.0	7.3	15.4	9.2	28.6	9.1	20.5	8.9	15.2	10.8	16.4	10.7	18.0	10.8	16.4	9.1
5	5.5	4.8	24.7	8.5	19.9	4.8	12.8	8.2	20.4	8.0	20.6	9.3	19.1	11.3	17.6	10.3	20.6	10.3	20.4	8.4
6	6	5.4	26.3	6.7	20.3	5.2	15.0	9.1	22.0	8.6	21.9	10.0	20.4	9.2	19.3	11.6	21.9	9.0	18.4	8.3
7	6.5	6.0	26.7	7.0	24.1	6.1	15.6	9.5	22.3	8.8	22.8	9.4	19.8	9.7	22.4	10.5	20.3	9.8	20.3	8.5
8	7	6.4	28.0	6.4	18.1	7.1	15.2	9.5	25.5	9.6	22.3	8.8	18.7	10.1	21.0	10.4	17.8	10.2	16.4	8.7
9	7.5	2.9	13.1	5.0	7.9	3.3	5.7	6.9	11.7	6.4	8.5	6.5	6.6	7.3	10.1	7.1	10.3	8.0	8.1	5.9
10	8	5.2	18.9	5.3	12.3	5.3	10.8	8.9	15.6	9.1	13.4	8.6	12.1	9.7	13.8	10.3	14.6	11.0	15.2	8.1
11	8.5	6.0	21.4	6.5	17.4	5.8	13.6	9.3	18.0	10.2	16.4	10.1	16.2	11.2	16.5	11.4	16.1	10.0	18.9	8.9
12	9	7.2	24.0	7.1	16.5	6.5	13.6	11.1	21.6	10.4	13.1	10.9	18.5	10.7	21.0	12.6	16.5	9.7	14.9	9.6
13	9.5	1.5	0.6	N/A	0.6	1.3	0.9	1.2	0.6	1.3	1.0	1.4	1.2	1.8	1.0	1.5	1.9	N/A	2.5	1.4
14	10	1.6	1.2	1.6	1.6	1.4	1.9	2.7	1.3	2.1	1.9	2.4	2.4	2.8	1.5	2.7	1.8	5.9	2.8	2.6
15	10.5	2.1	3.4	2.0	2.8	2.0	3.0	4.3	5.8	3.0	2.9	2.5	4.0	4.9	3.9	5.1	2.3	5.1	3.0	3.4
16	11	4.3	9.8	5.0	4.9	3.8	3.0	5.3	9.3	5.4	4.1	4.7	5.2	6.6	7.8	7.3	3.7	6.4	4.2	5.4

Table S96. Binding conditions for surfaces printed under conditions shown in **Table S13**.

TPO (mM)	PETT (mM)	EGDMA (mM)	Intensity (mW/mm²)	[**SCR043**] (μM)	[**α-Gluc-FL**] (M)
1	100	1300	2.53	100	10^{-4}

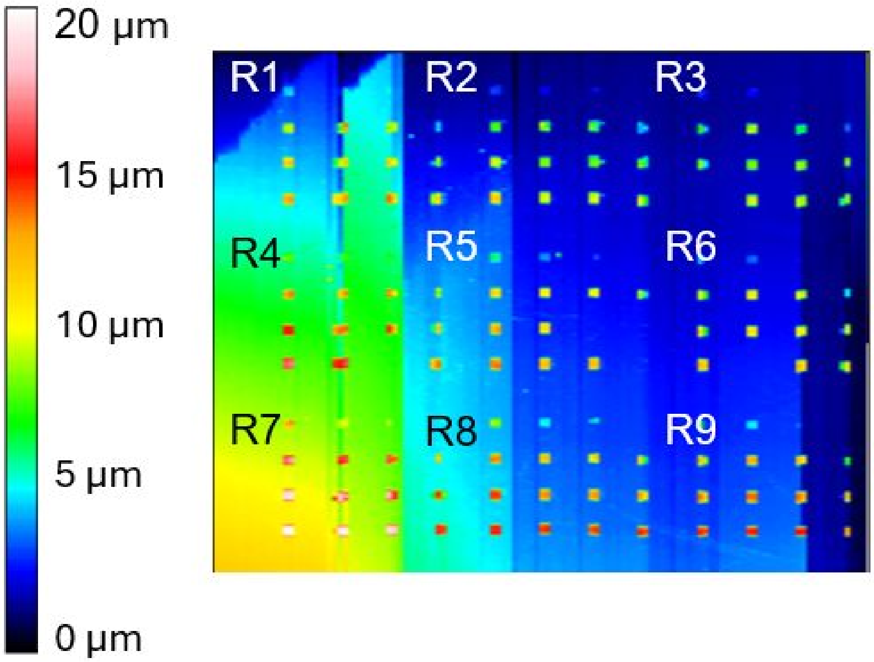

Figure S177. Profilometry image of patterns printed using [TPO] = 100 mM, [EGDMA] = 1300 mM. The pattern printed is 9 repeats (R1-R9) of 16 different time points. The scale bar is 200 μm.

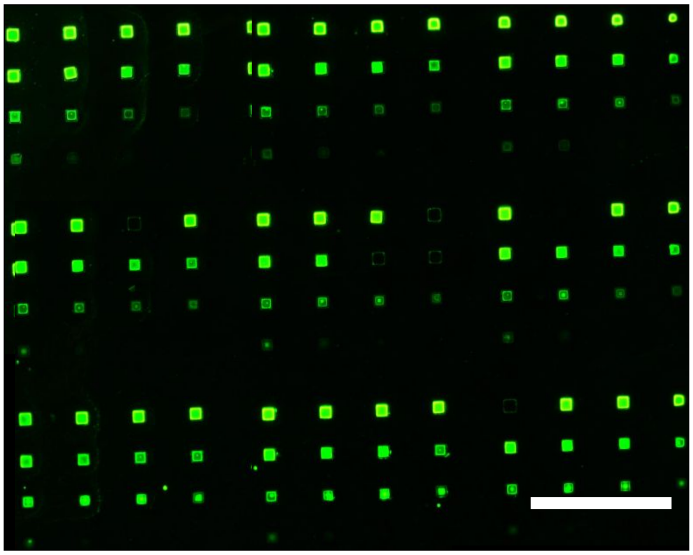

Figure S178. Fluorescence image of patterns printed using [TPO] = 100 mM, [EGDMA] = 1300 mM. Incubation was performed for 1 hour. The pattern printed is 9 repeats (R1-R9) of 16 different time points. The scale bar is 200 µm.

Table S97. Fluorescence intensity (I) and heights (h) of features printed under conditions in **Table S96**, and shown in **Figure S177** and **Figure S178**, # = feature number.

#	Time (min)	R1	I	R2	I	R3	I	R4	I	R5	I	R6	I	R7	I	R8	I	R9	I	Average Height (µm)
1	3.5	2.8	3.7	2.9	3.6	5.0	1.9	1.9	4.1	2.9	4.6	5.1	3.9	1.6	8.1	3.6	4.4	5.4	3.9	3.5
2	4	N/A	1.3	4.9	1.2	1.8	1.1	1.1	2.1	1.7	1.9	2.2	1.5	1.6	2.7	1.8	2.2	2.3	2.6	2.2
3	4.5	1.0	1.1	1.6	0.7	1.6	0.6	1.6	1.0	1.2	1.0	4.1	0.8	0.3	1.5	0.4	1.3	0.8	1.3	1.4
4	5	N/A	0.9	N/A	0.6	0.6	0.7	0.3	1.0	0.2	0.6	N/A	0.9	0.3	1.1	0.5	0.5	0.2	1.0	0.4
5	5.5	6.1	16.2	7.2	14.0	8.2	13.6	6.2	11.7	7.8	11.4	9.0	9.9	6.0	16.6	7.9	12.9	9.4	10.9	7.5
6	6	9.0	15.9	6.5	14.3	7.4	13.6	8.4	9.3	8.1	9.9	9.1	11.0	8.3	12.4	8.7	9.0	9.8	10.7	8.4
7	6.5	6.5	15.8	7.4	16.3	8.3	12.3	7.1	6.6	8.3	8.0	9.1	6.0	5.2	6.6	8.2	6.2	9.2	8.9	7.7
8	7	3.6	11.6	6.7	11.0	7.8	4.5	5.0	6.1	7.6	4.8	8.4	3.8	2.0	4.4	4.6	4.5	7.0	4.2	5.9
9	7.5	7.8	23.4	8.4	25.5	9.3	25.4	7.3	25.3	9.4	26.3	9.9	29.0	7.2	30.9	9.0	27.9	10.6	30.3	8.8
10	8	6.1	19.0	6.5	21.1	10.1	20.8	6.9	21.0	8.6	22.6	9.9	22.5	6.6	27.8	8.6	21.9	10.6	24.8	8.2
11	8.5	6.5	16.8	7.8	20.9	9.8	19.4	6.7	17.5	0.9	3.0	9.8	18.7	7.1	19.8	8.4	19.1	10.5	21.6	7.5
12	9	6.6	15.6	7.8	16.1	9.7	13.1	6.5	14.9	0.8	2.7	9.8	14.8	7.3	16.7	8.9	14.6	11.0	12.9	7.6
13	9.5	8.3	26.3	9.3	36.8	9.6	2.9	8.8	28.8	9.4	36.2	10.9	38.4	0.5	30.7	9.9	32.2	11.4	33.4	8.7
14	10	7.2	29.8	8.4	32.9	9.8	35.5	7.6	31.1	9.3	36.0	11.0	0.9	7.7	34.1	N/A	31.8	11.3	32.3	9.0
15	10.5	8.2	30.1	N/A	33.6	10.4	33.4	7.8	2.9	9.5	32.8	10.8	35.4	7.5	30.7	9.8	31.9	13.6	29.9	9.7
16	11	9.1	30.2	9.3	32.8	10.2	27.0	7.9	30.8	0.4	3.5	10.8	29.0	7.3	31.8	10.3	33.3	11.8	16.8	8.6

Table S98. Binding conditions for surfaces printed under conditions shown in **Table S13**.

TPO (mM)	PETT (mM)	EGDMA (mM)	Intensity (mW/mm^2)	[**SCR043**] (μM)	[**α-Gluc-FL**] (M)
1	100	1300	2.53	100	$10^{-4.5}$

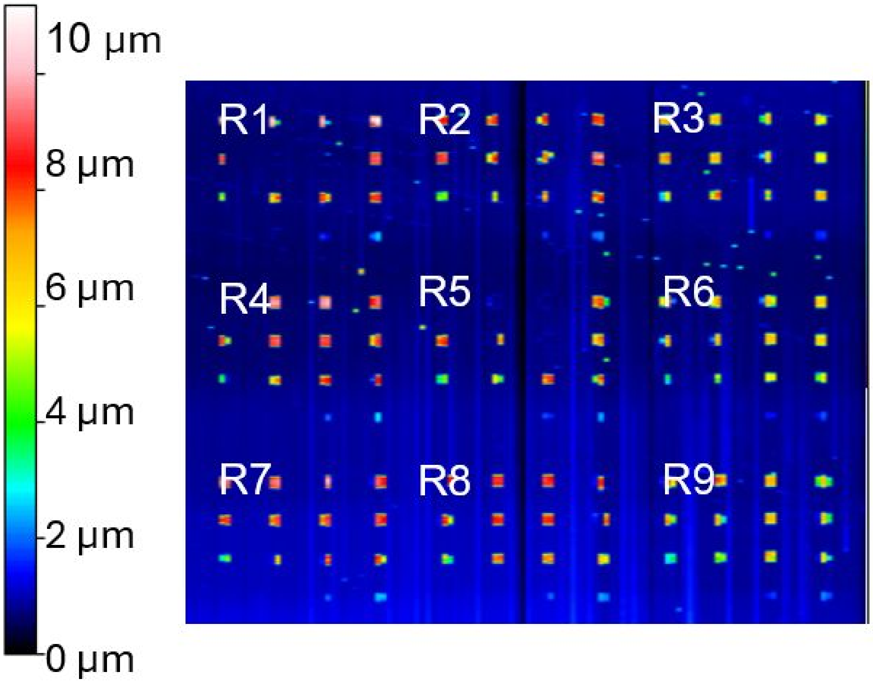

Figure S179. Profilometry image of patterns printed using [TPO] = 100 mM, [EGDMA] = 1300 mM. The pattern printed is 9 repeats (R1-R9) of 16 different time points. The scale bar is 200 μm.

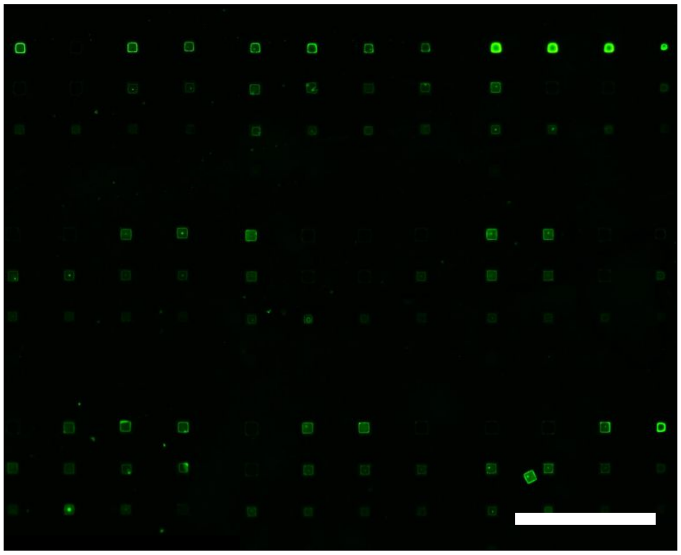

Figure S180. Fluorescence image of patterns printed using [TPO] = 100 mM, [EGDMA] = 1300 mM. Incubation was performed for 1 hour. The pattern printed is 9 repeats (R1-R9) of 16 different time points. The scale bar is 200 μm.

Table S99. Fluorescence intensity (*I*) and heights (*h*) of features printed under conditions in **Table S98**, and shown in **Figure S179** and **Figure S180**, # = feature number.

#	Time (min)	R1	*I*	R2	*I*	R3	*I*	R4	*I*	R5	*I*	R6	*I*	R7	*I*	R8	*I*	R9	*I*	Average Height (μm)
1	3.5	9.9	11.6	N/A	8.0	8.8	11.5	7.7	1.9	N/A	1.6	7.5	14.2	6.4	17.8	7.0	2.0	7.5	13.9	7.8
2	4	9.8	26.8	9.3	11.2	8.5	16.7	7.9	1.6	N/A	1.6	7.9	13.3	6.0	14.2	6.5	13.8	7.3	14.9	7.9
3	4.5	9.2	31.6	9.4	16.5	9.2	1.4	8.0	13.3	N/A	2.3	8.0	2.2	5.7	1.8	6.3	11.8	6.4	9.7	7.8
4	5	10.5	32.4	8.8	15.3	8.4	20.3	7.4	16.4	7.5	16.6	7.4	1.6	5.8	1.8	6.0	2.8	6.1	2.1	7.5
5	5.5	8.5	3.4	8.3	6.8	7.9	4.9	8.6	4.0	7.8	4.6	7.4	5.2	6.9	3.2	6.8	4.7	6.6	11.1	7.6
6	6	N/A	1.9	8.6	5.1	8.1	5.7	7.6	2.0	6.7	1.8	7.3	6.1	6.2	4.8	6.4	5.8	6.2	7.5	7.1
7	6.5	N/A	2.1	8.5	9.3	8.1	1.6	6.9	6.4	N/A	1.9	7.3	7.8	5.8	10.5	6.3	7.1	6.5	6.0	7.0
8	7	8.5	11.1	8.3	9.9	8.2	2.0	9.3	9.3	7.1	6.9	7.1	8.7	5.0	9.7	5.9	2.5	5.9	7.8	7.3
9	7.5	4.4	1.8	3.8	4.5	3.9	2.5	4.2	1.6	4.1	2.3	5.4	2.4	5.5	1.8	3.4	2.8	2.9	2.7	4.2
10	8	7.4	3.6	7.0	4.8	5.8	2.9	6.0	2.6	5.3	2.3	7.0	3.2	7.2	2.1	5.0	3.1	4.5	4.7	6.1
11	8.5	8.3	5.3	7.8	4.6	7.8	3.8	7.2	3.2	8.6	5.8	6.7	3.6	6.0	5.7	5.4	4.4	6.7	12.3	7.2
12	9	8.2	6.3	7.8	7.6	0.0	2.7	7.8	3.8	7.5	4.5	6.0	4.4	6.5	5.0	5.5	5.5	5.4	4.1	6.1
13	9.5	0.4	0.9	0.1	0.9	0.0	1.2	0.2	1.0	N/A	0.9	0.3	1.0	N/A	0.0	N/A	N/A	N/A	0.0	0.2
14	10	0.9	1.3	N/A	1.0	N/A	1.5	0.1	1.1	0.2	1.0	0.1	1.0	N/A	0.0	N/A	N/A	N/A	0.0	0.3
15	10.5	1.0	0.9	1.2	1.5	1.0	1.7	0.9	1.3	0.9	1.2	0.9	1.1	0.8	0.0	0.7	N/A	0.8	0.0	0.9
16	11	1.9	1.8	2.0	1.7	1.6	1.2	1.5	1.3	1.6	1.5	1.4	1.6	1.0	0.0	1.2	N/A	1.4	0.0	1.5

Table S100. Binding conditions for surfaces printed under conditions shown in **Table S13**.

TPO (mM)	PETT (mM)	EGDMA (mM)	Intensity (mW/mm^2)	[**SCR043**] (μM)	[**α-Gluc-FL**] (M)
1	100	1300	2.53	100	10^{-5}

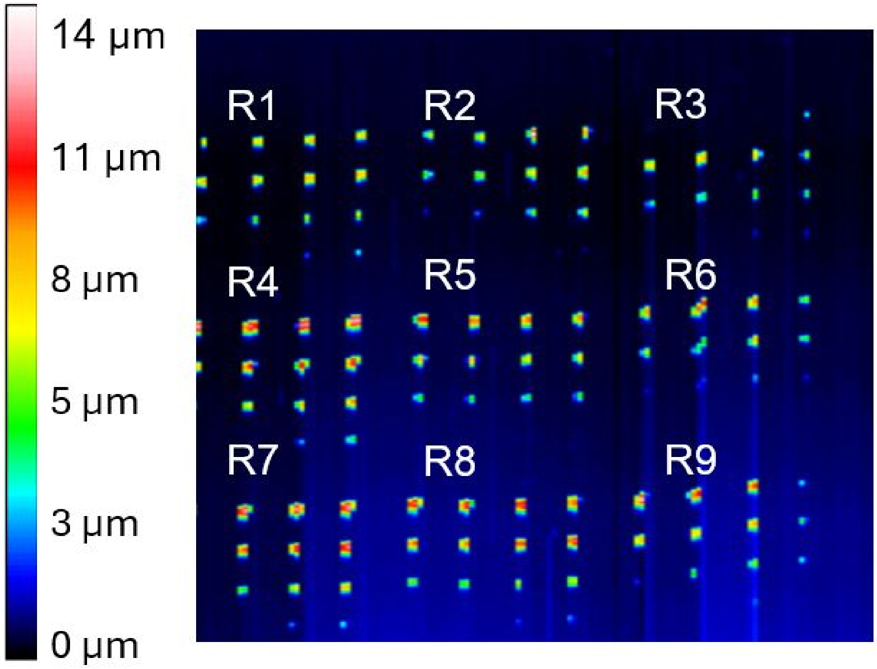

Figure S181. Profilometry image of patterns printed using [TPO] = 100 mM, [EGDMA] = 1300 mM. The pattern printed is 9 repeats (R1-R9) of 16 different time points. The scale bar is 200 μm.

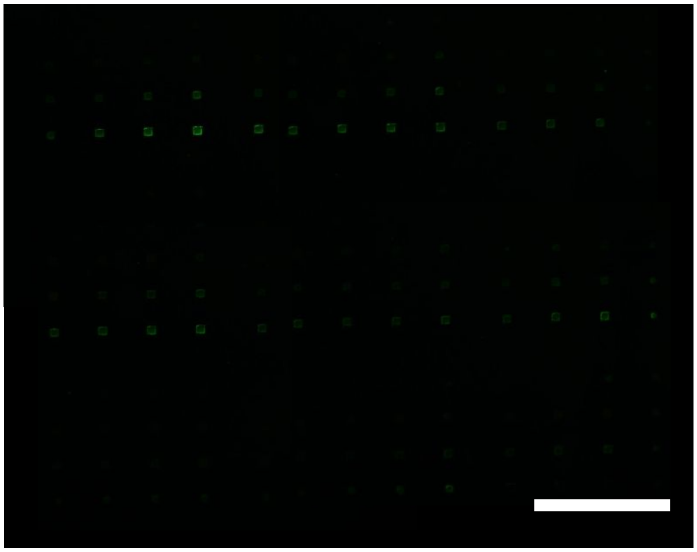

Figure S182. Fluorescence image of patterns printed using [TPO] = 100 mM, [EGDMA] = 1300 mM. Incubation was performed for 1 hour. The pattern printed is 9 repeats (R1-R9) of 16 different time points. The scale bar is 200 µm.

Table S101. Fluorescence intensity (*I*) and heights (*h*) of features printed under conditions in **Table S100**, and shown in **Figure S181** and **Figure S182**, # = feature number.

#	Time (min)	R1	*I*	R2	*I*	R3	*I*	R4	*I*	R5	*I*	R6	*I*	R7	*I*	R8	*I*	R9	*I*	Average Height (µm)
1.0	3.5	N/A	0.9	N/A	1.0	N/A	1.1	N/A	0.7	N/A	1.0	N/A	1.0	0.0	N/A	0.0	N/A	N/A	N/A	N/A
2.0	4.0	0.1	1.2	N/A	1.1	N/A	0.8	N/A	0.7	N/A	0.9	0.2	0.8	0.1	N/A	0.3	N/A	N/A	N/A	0.2
3.0	4.5	1.0	1.3	N/A	1.3	0.7	0.9	N/A	1.0	0.2	0.9	1.0	1.2	1.9	N/A	2.5	N/A	2.1	N/A	0.7
4.0	5.0	1.2	1.3	0.0	1.2	N/A	0.9	0.8	1.2	0.1	1.0	1.9	1.2	3.3	N/A	4.1	N/A	2.8	N/A	0.8
5.0	5.5	4.5	1.2	0.8	1.2	1.3	1.2	1.3	0.9	4.4	1.0	5.2	1.1	4.1	N/A	5.0	N/A	3.7	N/A	2.9
6.0	6.0	4.3	1.3	1.0	1.2	4.4	1.2	2.3	1.0	4.8	1.0	4.8	1.6	5.5	N/A	7.0	N/A	5.4	N/A	3.6
7.0	6.5	5.5	1.5	1.9	1.5	4.6	1.8	5.8	1.3	4.5	1.2	5.9	1.4	6.2	N/A	8.3	N/A	7.4	N/A	4.7
8.0	7.0	4.6	1.7	1.2	1.9	3.2	1.9	6.8	1.5	5.1	1.2	6.1	1.4	6.8	N/A	9.1	N/A	8.0	N/A	4.5
9.0	7.5	7.8	1.5	6.1	1.9	8.4	1.8	5.3	1.1	8.3	1.3	9.3	1.5	9.1	N/A	10.0	N/A	8.7	N/A	7.5
10.0	8.0	8.4	1.9	4.2	2.2	7.4	3.3	6.2	1.7	7.7	1.4	9.0	2.1	8.7	N/A	10.5	N/A	9.7	N/A	7.2
11.0	8.5	7.7	2.9	7.9	2.6	7.6	3.7	7.6	2.2	7.6	1.5	9.5	2.0	8.6	N/A	10.8	N/A	11.1	N/A	8.0
12.0	9.0	6.4	4.3	4.8	3.8	4.2	3.3	9.3	2.8	7.4	1.8	11.2	1.8	8.3	N/A	11.0	N/A	11.4	N/A	7.2
13.0	9.5	0.3	2.6	9.0	5.3	12.7	N/A	6.8	3.0	10.3	2.2	11.0	2.5	7.6	N/A	12.4	N/A	9.5	N/A	8.4
14.0	10.0	0.2	4.6	10.1	5.9	9.9	N/A	7.6	3.5	9.3	2.1	10.6	3.6	8.2	N/A	11.9	N/A	12.3	N/A	8.0
15.0	10.5	0.2	7.0	9.1	5.8	9.2	N/A	14.3	4.1	9.5	1.9	10.8	5.0	8.8	N/A	12.1	N/A	12.0	N/A	8.9
16.0	11.0	3.7	8.7	5.8	5.2	3.0	N/A	8.5	4.8	9.4	3.3	9.3	2.8	8.1	N/A	12.4	N/A	11.6	N/A	6.6

Table S102. Binding conditions for surfaces printed under conditions shown in **Table S13**.

TPO (mM)	PETT (mM)	EGDMA (mM)	Intensity (mW/mm^2)	[**SCR043**] (μM)	[**α-Gluc-FL**] (M)
1	100	1300	2.53	100	$10^{-5.5}$

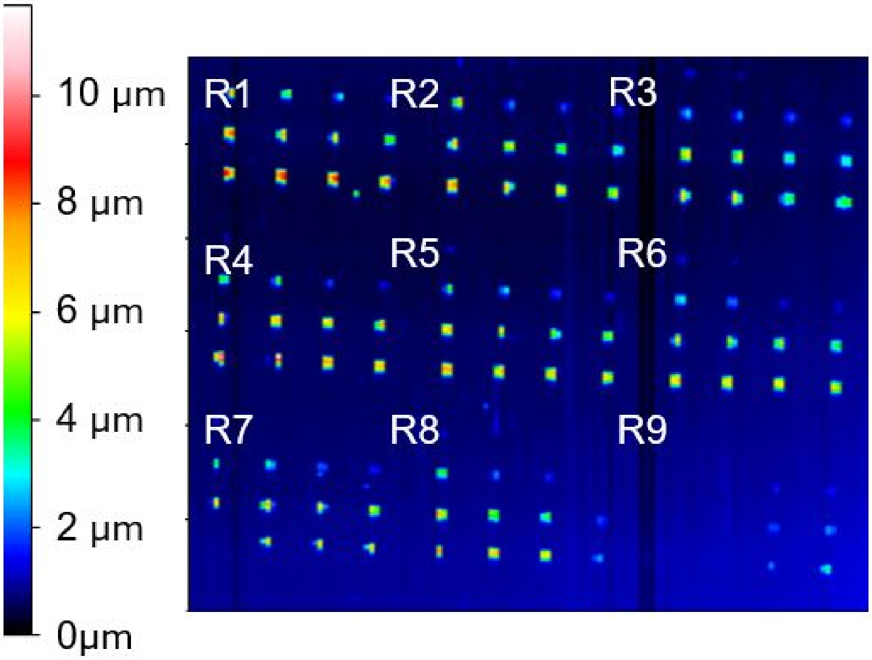

Figure S183. Profilometry image of patterns printed using [TPO] = 100 mM, [EGDMA] = 1300 mM. The pattern printed is 9 repeats (R1-R9) of 16 different time points. The scale bar is 200 μm.

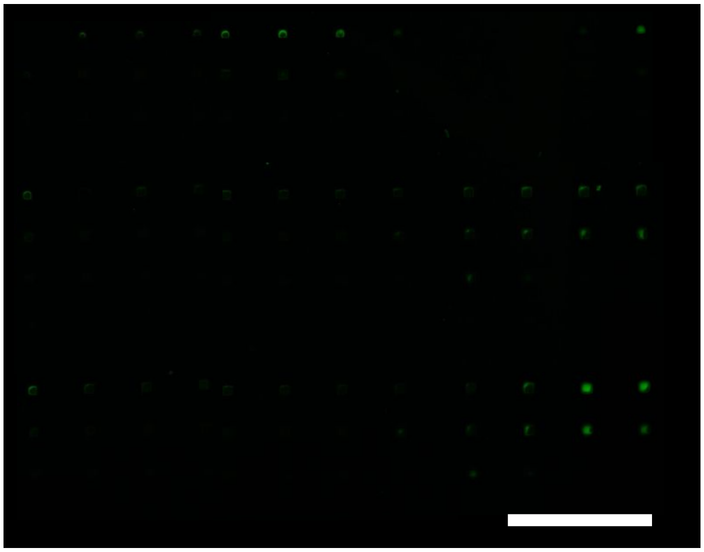

Figure S184. Fluorescence image of patterns printed using [TPO] = 100 mM, [EGDMA] = 1300 mM. Incubation was performed for 1 hour. The pattern printed is 9 repeats (R1-R9) of 16 different time points. The scale bar is 200 μm.

Table S103. Fluorescence intensity (*I*) and heights (*h*) of features printed under conditions in **Table S102**, and shown in **Figure S183** and **Figure S184**, # = feature number

Table S104. Binding conditions for surfaces printed under conditions shown in **Table S13**.

TPO (mM)	PETT (mM)	EGDMA (mM)	Intensity (mW/mm^2)	[**SCR043**] (μM)	[**β-Gluc-FL**] (M)
1	100	1300	2.53	100	10^{-3}

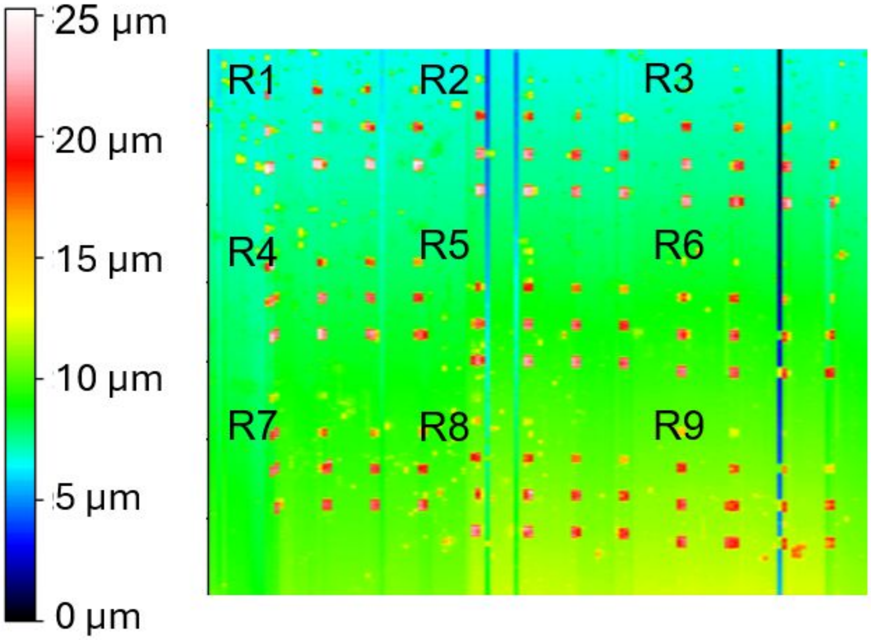

Figure S185. Profilometry image of patterns printed using [TPO] = 100 mM, [EGDMA] = 1300 mM. The pattern printed is 9 repeats (R1-R9) of 16 different time points. The scale bar is 200 μm.

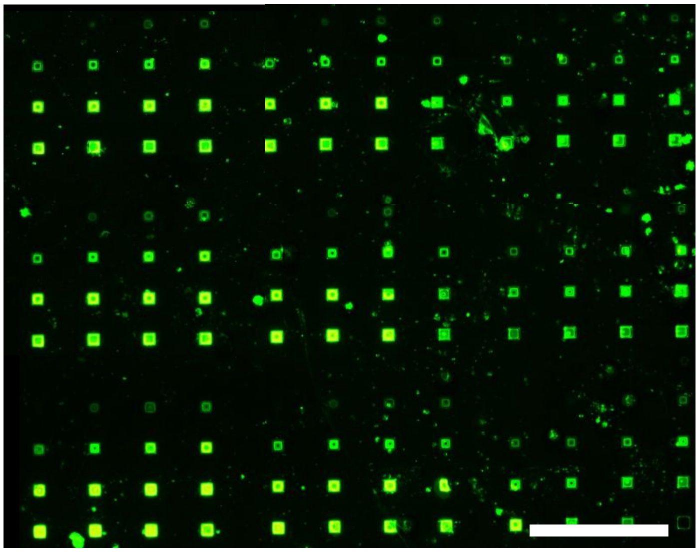

Figure S186. Fluorescence image of patterns printed using [TPO] = 100 mM, [EGDMA] = 1300 mM. Incubation was performed for 1 hour. The pattern printed is 9 repeats (R1-R9) of 16 different time points. The scale bar is 200 μm.

Table S105. Fluorescence intensity (*I*) and heights (*h*) of features printed under conditions in **Table S104**, and shown in **Figure S185** and **Figure S186**, # = feature number

#	Time (min)	R1	*I*	R2	*I*	R3	*I*	R4	*I*	R5	*I*	R6	*I*	R7	*I*	R8	*I*	R9	*I*	Average Height (μm)
1	3.5	6.9	2.1	7.7	2.4	7.0	3.2	N/A	3.0	N/A	1.1	6.2	5.8	6.0	3.7	7.5	3.1	5.1	4.5	N/A
2	4	6.5	3.0	5.7	2.1	3.4	2.6	8.6	1.6	5.2	3.2	4.8	3.7	2.3	3.6	4.7	3.4	2.8	3.0	4.9
3	4.5	N/A	1.2	N/A	1.5	N/A	1.5	N/A	1.6	N/A	1.8	N/A	2.0	N/A	1.3	N/A	2.0	1.3	1.8	N/A
4	5	N/A	1.0	N/A	0.8	N/A	1.0	N/A	3.7	N/A	1.0	N/A	1.0	N/A	1.1	N/A	0.9	N/A	0.6	N/A
5	5.5	14.5	6.0	15.6	5.6	11.9	11.5	13.3	10.6	11.5	6.1	10.8	13.4	13.2	9.6	10.4	6.7	9.8	14.4	12.4
6	6	13.3	5.4	11.7	6.9	9.3	9.4	12.0	7.8	11.8	11.9	8.5	10.9	11.3	6.1	10.6	9.2	8.0	11.4	10.7
7	6.5	12.6	6.5	10.4	8.7	8.1	7.5	10.7	6.2	9.5	7.9	7.2	8.5	10.2	4.4	8.3	8.1	6.7	8.3	9.3
8	7	9.6	4.5	8.8	7.2	7.1	6.0	9.0	3.7	8.1	8.2	5.7	6.3	7.9	4.1	6.7	6.4	4.2	5.2	7.5
9	7.5	16.8	11.6	14.9	12.3	12.1	20.8	15.3	13.8	12.6	8.5	10.6	20.8	15.5	9.1	12.6	16.9	10.7	20.9	13.5
10	8	16.8	9.7	14.1	17.7	14.2	19.6	15.6	10.3	13.4	15.3	13.2	20.4	13.9	9.7	12.5	20.8	9.6	21.0	13.7
11	8.5	15.4	9.4	13.1	18.5	11.2	18.3	14.3	8.8	13.0	17.8	10.8	18.9	14.5	9.1	10.7	17.9	9.3	19.0	12.5
12	9	14.2	8.3	12.3	15.3	11.5	15.5	14.1	7.8	11.8	16.1	9.6	15.9	12.0	10.8	10.8	16.6	9.3	16.5	11.7
13	9.5	18.4	12.8	15.5	11.8	11.2	19.5	16.4	11.2	12.7	10.3	11.5	20.5	15.0	2.8	12.2	18.1	10.0	20.2	13.6
14	10	17.3	11.3	16.5	17.7	10.9	18.7	16.0	10.4	13.6	20.6	11.8	19.4	14.3	9.0	11.7	19.0	9.8	23.3	13.5
15	10.5	17.0	11.7	14.4	17.0	11.3	19.4	15.0	10.8	13.3	21.8	10.7	19.0	15.2	12.0	11.1	19.2	8.4	23.0	12.9
16	11	17.9	15.1	12.8	18.9	12.5	17.8	15.4	7.4	12.7	21.5	10.0	17.8	14.7	21.8	11.5	22.2	8.2	22.0	12.9

Table S106. Binding conditions for surfaces printed under conditions shown in **Table S13**.

TPO (mM)	PETT (mM)	EGDMA (mM)	Intensity (mW/mm^2)	[**SCR043**] (μM)	[**β-Gluc-FL**] (M)
1	100	1300	2.53	100	$10^{-3.5}$

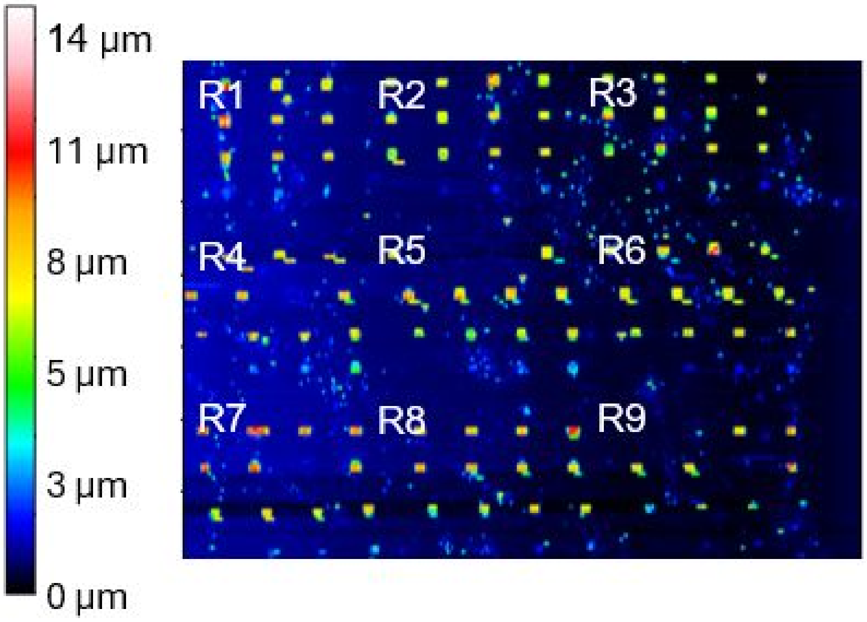

Figure S187. Profilometry image of patterns printed using [TPO] = 100 mM, [EGDMA] = 1300 mM. The pattern printed is 9 repeats (R1-R9) of 16 different time points. The scale bar is 200 μm.

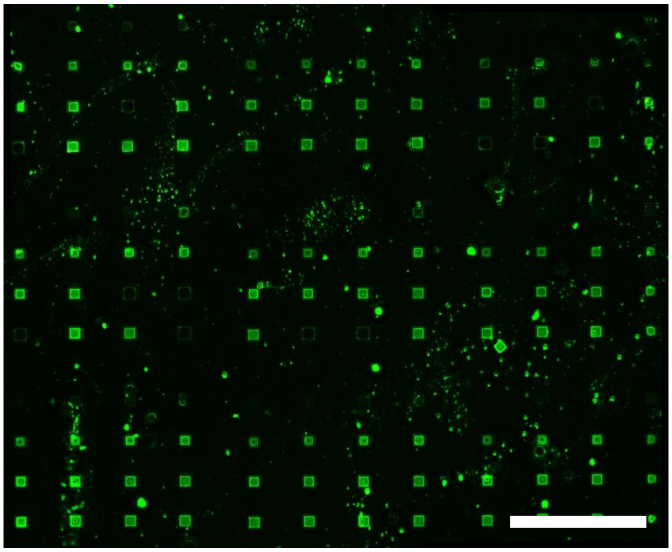

Figure S188. Fluorescence image of patterns printed using [TPO] = 100 mM, [EGDMA] = 1300 mM. Incubation was performed for 1 hour. The pattern printed is 9 repeats (R1-R9) of 16 different time points. The scale bar is 200 μm.

Table S107. Fluorescence intensity (*I*) and heights (*h*) of features printed under conditions in **Table S106**, and shown in **Figure S187** and **Figure S188**, # = feature number

#	Time (min)	R1	*I*	R2	*I*	R3	*I*	R4	*I*	R5	*I*	R6	*I*	R7	*I*	R8	*I*	R9	*I*	Average Height (μm)
1	3.5	N/A	11.8	N/A	8.4	9.8	8.0	6.7	1.7	7.8	6.8	8.3	9.1	6.8	1.4	8.0	6.4	N/A	2.4	7.9
2	4	10.1	13.1	8.8	8.8	11.3	8.1	7.0	9.0	N/A	2.0	8.2	9.0	7.1	6.0	8.7	6.1	N/A	2.2	8.8
3	4.5	7.2	9.5	8.1	12.3	8.9	6.7	11.3	7.0	N/A	2.1	8.3	9.4	7.1	4.7	13.1	6.4	8.0	7.5	9.0
4	5	7.7	10.0	8.1	8.2	9.4	5.1	7.1	1.7	7.5	7.3	11.1	6.0	13.3	5.7	8.2	7.6	10.1	7.3	9.2
5	5.5	N/A	10.3	8.5	7.9	9.6	10.6	7.4	7.2	9.4	8.1	8.7	8.0	10.4	4.3	8.3	5.3	8.2	6.0	8.8
6	6	13.7	13.2	8.0	7.8	9.2	8.4	7.1	7.9	9.2	6.9	8.9	6.7	8.0	4.7	7.9	6.1	8.2	6.2	8.9
7	6.5	7.6	9.2	N/A	9.8	N/A	8.0	8.5	1.9	9.2	7.3	8.6	7.3	7.4	1.3	8.3	5.6	N/A	1.8	8.3
8	7	8.7	9.2	7.9	7.9	8.6	5.4	7.1	1.9	9.2	6.7	8.0	6.4	8.7	5.2	8.7	5.5	12.1	8.5	8.8
9	7.5	N/A	6.2	9.5	4.9	7.7	4.7	7.1	6.3	6.7	4.1	7.8	4.4	6.8	3.9	9.5	2.8	5.1	3.1	7.5
10	8	11.8	11.6	9.8	6.2	8.1	7.7	7.7	7.6	7.8	4.4	8.4	5.1	8.9	2.9	8.8	3.5	7.6	5.9	8.8
11	8.5	9.1	9.2	8.9	7.2	8.8	7.3	8.9	7.0	8.4	6.7	8.9	6.2	8.8	3.4	9.2	3.9	6.9	3.6	8.7
12	9	8.5	8.5	8.8	8.0	9.4	4.5	8.9	6.9	9.1	6.3	8.9	5.4	8.2	3.6	11.1	5.0	4.7	1.8	8.6
13	9.5	N/A	1.1	N/A	1.2	0.0	1.2	N/A	0.8	N/A	1.0	N/A	1.3	N/A	0.6	N/A	1.9	N/A	1.1	0.0
14	10	N/A	3.8	2.3	1.4	3.8	1.6	0.8	1.4	N/A	4.6	N/A	1.5	1.6	2.4	1.8	2.0	0.9	1.3	1.9
15	10.5	1.7	2.6	2.8	1.8	1.9	5.1	1.6	2.3	3.8	3.6	5.5	1.8	9.8	1.0	1.8	2.3	1.4	1.0	3.4
16	11	3.3	2.9	5.8	3.3	3.7	1.3	5.2	3.4	4.1	2.9	4.1	1.6	1.7	1.2	2.3	1.7	2.5	2.0	3.6

Table S108. Binding conditions for surfaces printed under conditions shown in **Table S13**.

TPO (mM)	PETT (mM)	EGDMA (mM)	Intensity (mW/mm^2)	[**SCR043**] (μM)	[**β-Gluc-FL**] (M)
1	100	1300	2.53	100	10^{-4}

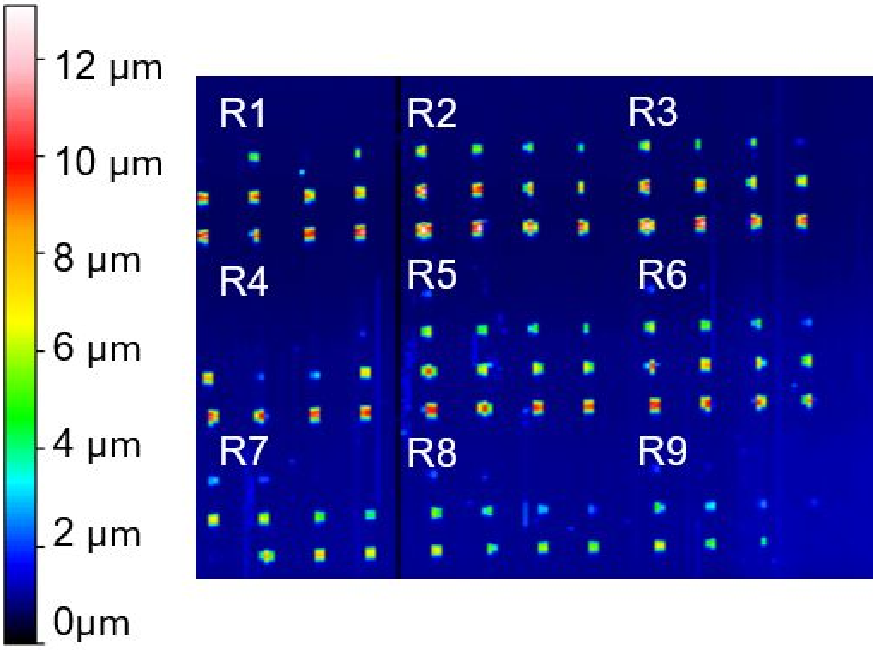

Figure S189. Profilometry image of patterns printed using [TPO] = 100 mM, [EGDMA] = 1300 mM. The pattern printed is 9 repeats (R1-R9) of 16 different time points. The scale bar is 200 μm.

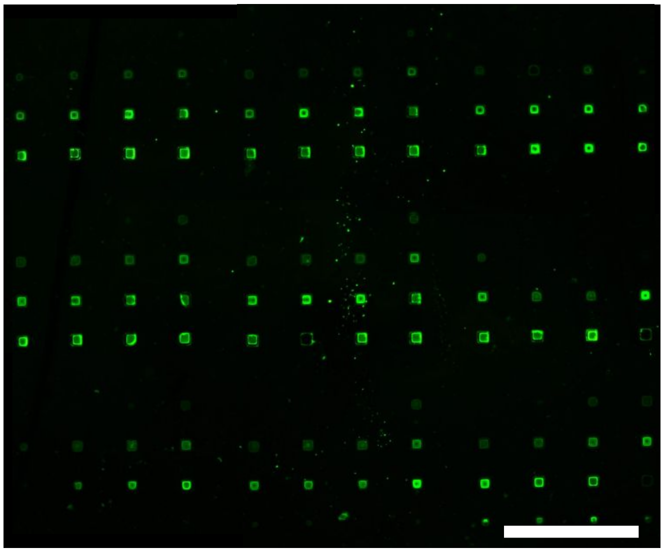

Figure S190. Fluorescence image of patterns printed using [TPO] = 100 mM, [EGDMA] = 1300 mM. Incubation was performed for 1 hour. The pattern printed is 9 repeats (R1-R9) of 16 different time points. The scale bar is 200 µm.

Table S109. Fluorescence intensity (*I*) and heights (*h*) of features printed under conditions in **Table S108**, and shown in **Figure S189** and **Figure S190**, # = feature number

#	Time (min)	R1	*I*	R2	*I*	R3	*I*	R4	*I*	R5	*I*	R6	*I*	R7	*I*	R8	*I*	R9	*I*	Average Height (µm)
1.0	3.5	N/A	1.0	1.1	1.2	N/A	1.0	N/A	1.0	N/A	1.1	N/A	0.9	N/A	1.0	N/A	0.8	N/A	1.3	1.1
2.0	4.0	N/A	1.4	0.8	1.1	N/A	1.3	N/A	0.9	N/A	1.4	N/A	0.8	N/A	1.1	N/A	0.9	N/A	1.4	0.8
3.0	4.5	N/A	1.4	N/A	1.5	N/A	1.0	N/A	1.6	N/A	1.7	N/A	0.7	N/A	1.3	0.9	1.3	0.5	2.6	0.7
4.0	5.0	N/A	1.4	N/A	1.7	0.2	0.9	N/A	2.8	1.7	2.6	0.4	0.6	N/A	2.0	1.6	3.0	N/A	2.5	1.0
5.0	5.5	N/A	2.3	6.5	2.8	5.6	2.6	N/A	3.1	4.0	3.5	3.9	2.3	N/A	1.8	1.6	3.0	1.1	3.8	3.8
6.0	6.0	N/A	3.0	2.5	3.1	3.0	2.2	N/A	3.9	4.3	3.9	4.8	0.9	N/A	3.7	4.1	4.5	4.9	5.0	3.9
7.0	6.5	1.3	3.8	1.5	3.8	5.3	3.4	1.2	5.1	4.5	4.0	5.3	0.7	0.6	5.2	4.7	4.7	4.4	7.4	3.2
8.0	7.0	2.1	4.6	7.7	5.2	0.6	1.4	1.5	7.3	6.0	6.9	7.4	0.8	1.1	6.6	5.8	6.6	6.4	9.1	4.3
9.0	7.5	3.5	6.6	9.3	7.9	8.1	8.8	1.5	8.9	6.1	8.8	7.0	8.1	0.8	0.7	5.5	7.6	6.8	12.5	5.4
10.0	8.0	4.7	8.9	10.4	12.8	8.4	10.9	2.1	9.0	6.3	10.3	7.9	4.8	1.5	4.0	6.6	7.2	6.9	12.1	6.1
11.0	8.5	5.7	12.7	9.0	12.0	9.0	11.4	4.4	9.4	6.4	11.6	9.9	3.7	2.7	7.4	7.8	6.4	7.9	10.6	7.0
12.0	9.0	6.2	10.0	10.0	8.3	9.9	8.0	5.1	8.0	8.7	8.8	11.6	10.6	4.0	11.0	8.4	12.0	10.5	1.6	8.3
13.0	9.5	6.0	10.9	N/A	11.9	11.0	10.7	4.8	11.9	8.5	7.8	10.5	10.5	N/A	N/A	7.7	N/A	10.2	4.1	8.4
14.0	10.0	6.8	10.8	N/A	12.7	9.5	15.3	5.1	12.5	8.7	3.3	10.9	10.6	3.0	N/A	8.5	N/A	10.4	3.7	7.9
15.0	10.5	7.4	15.0	N/A	13.9	8.5	13.1	4.6	11.9	9.3	10.5	12.3	15.3	4.5	N/A	9.2	N/A	11.4	5.0	8.4
16.0	11.0	0.4	15.4	N/A	13.9	10.9	10.9	6.7	12.2	9.5	10.9	12.4	3.1	5.9	N/A	9.7	N/A	11.5	1.8	8.4

Table S110. Binding conditions for surfaces printed under conditions shown in **Table S13**.

TPO (mM)	PETT (mM)	EGDMA (mM)	Intensity (mW/mm^2)	[**SCR043**] (μM)	[**β-Gluc-FL**] (M)
1	100	1300	2.53	100	$10^{-4.5}$

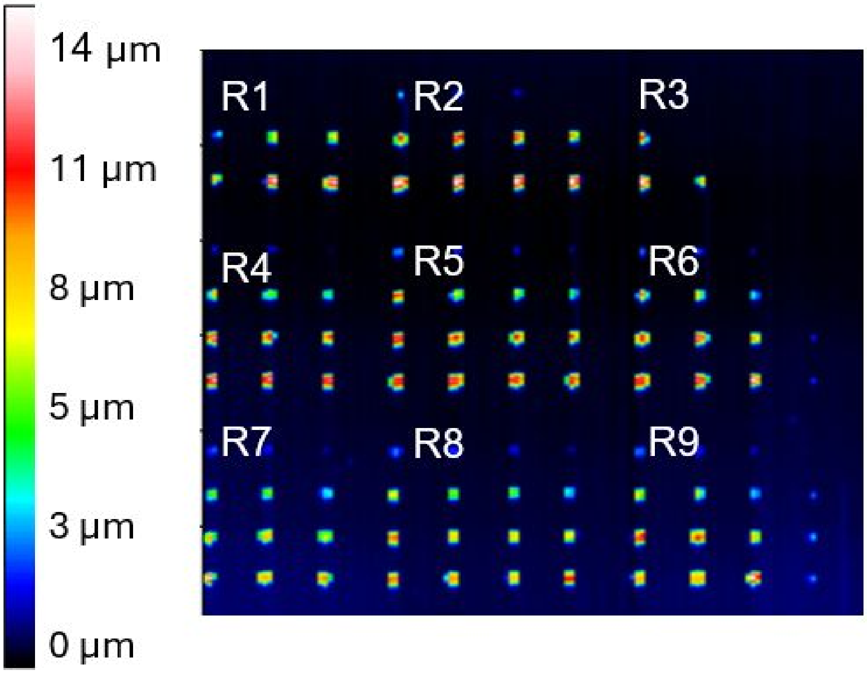

Figure S191. Profilometry image of patterns printed using [TPO] = 100 mM, [EGDMA] = 1300 mM. The pattern printed is 9 repeats (R1-R9) of 16 different time points. The scale bar is 200 μm.

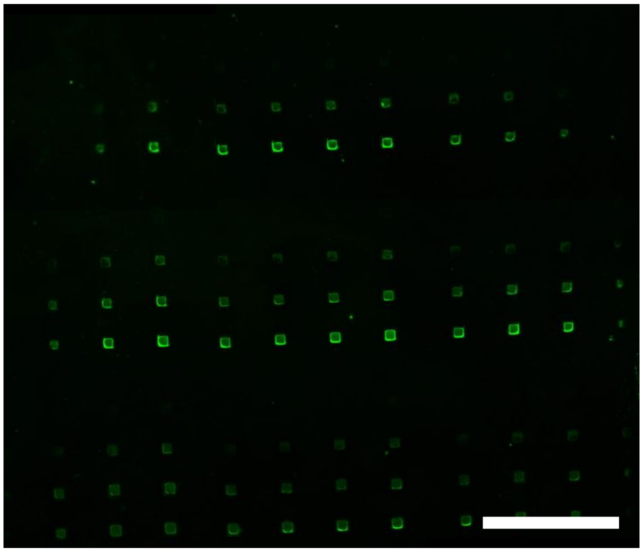

Figure S192. Fluorescence image of patterns printed using [TPO] = 100 mM, [EGDMA] = 1300 mM. Incubation was performed for 1 hour. The pattern printed is 9 repeats (R1-R9) of 16 different time points. The scale bar is 200 μm.

Table S111. Fluorescence intensity (*I*) and heights (*h*) of features printed under conditions in **Table S110**, and shown in **Figure S191** and **Figure S192**, # = feature number

#	Time (min)	R1	*I*	R2	*I*	R3	*I*	R4	*I*	R5	*I*	R6	*I*	R7	*I*	R8	*I*	R9	*I*	Average Height (μm)
1.0	3.5	0.7	N/A	0.4	1.4	N/A	1.3	0.8	1.3	0.9	1.7	N/A	0.9	0.2	1.3	N/A	1.0	N/A	1.1	0.6
2.0	4.0	1.2	N/A	1.1	1.3	N/A	1.8	1.1	1.4	1.4	1.8	N/A	1.0	0.9	1.0	0.9	1.2	N/A	0.9	1.1
3.0	4.5	1.7	N/A	1.5	1.9	N/A	1.6	1.6	1.8	1.7	1.5	N/A	1.0	1.4	1.2	2.0	1.1	N/A	1.0	1.6
4.0	5.0	N/A	N/A	N/A	1.3	N/A	1.2	2.1	2.0	2.3	1.3	N/A	1.1	2.0	1.4	2.1	1.2	N/A	1.2	2.1
5.0	5.5	2.9	N/A	4.2	1.4	0.0	1.7	3.2	1.2	4.6	2.3	N/A	1.9	1.6	0.9	N/A	1.4	0.0	1.4	2.4
6.0	6.0	4.8	1.4	5.2	1.5	0.0	1.9	5.0	1.9	5.9	2.2	1.2	2.3	2.5	1.5	3.5	1.8	N/A	2.5	3.5
7.0	6.5	4.6	1.3	7.4	1.4	N/A	1.3	5.0	3.2	6.4	2.7	2.2	2.3	5.5	2.8	7.1	2.6	0.0	2.7	4.8
8.0	7.0	N/A	1.1	N/A	1.7	N/A	1.2	6.2	4.6	9.1	3.3	2.8	1.5	6.3	3.6	8.4	2.6	N/A	1.5	6.6
9.0	7.5	5.7	N/A	8.8	4.0	6.7	5.0	6.2	1.6	8.9	4.2	8.2	4.2	2.4	0.9	1.5	3.3	N/A	3.0	6.1
10.0	8.0	7.1	1.6	9.5	4.9	6.3	4.4	7.0	3.6	9.3	4.0	10.3	5.9	6.9	3.1	8.0	3.3	N/A	3.8	8.1
11.0	8.5	7.3	2.1	8.4	5.2	3.0	2.0	6.7	5.7	9.4	4.8	10.3	5.6	9.2	4.4	10.5	3.4	N/A	3.6	8.1
12.0	9.0	N/A	4.0	N/A	6.1	N/A	1.3	8.2	7.8	10.0	5.9	9.5	2.8	9.3	5.1	9.5	3.9	8.8	1.0	9.2
13.0	9.5	7.8	N/A	10.1	11.0	11.1	9.8	9.1	1.7	9.7	7.5	12.3	9.0	1.9	0.6	1.5	5.7	N/A	6.1	7.9
14.0	10.0	7.2	1.4	10.3	12.7	11.7	7.9	6.8	5.3	9.7	8.5	11.9	11.2	11.5	3.1	11.6	6.0	N/A	5.2	10.1
15.0	10.5	11.1	3.5	10.7	12.3	7.5	4.2	7.7	9.1	10.0	9.6	11.9	9.3	7.1	4.3	10.0	6.3	7.4	3.1	9.3
16.0	11.0	N/A	8.2	N/A	12.5	N/A	2.0	8.0	11.9	9.8	10.6	11.9	2.1	7.1	4.9	9.7	7.0	12.2	0.8	9.8

Table S112. Binding conditions for surfaces printed under conditions shown in **Table S13**.

TPO (mM)	PETT (mM)	EGDMA (mM)	Intensity (mW/mm²)	[**SCR043**] (μM)	[**β-Gluc-FL**] (M)
1	100	1300	2.53	100	10^{-5}

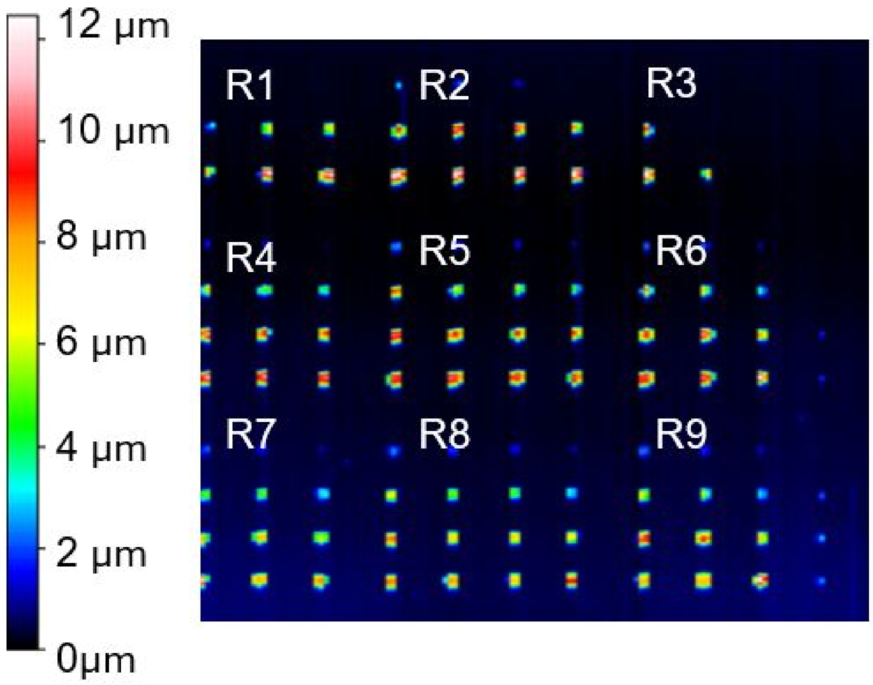

Figure S193. Profilometry image of patterns printed using [TPO] = 100 mM, [EGDMA] = 1300 mM. The pattern printed is 9 repeats (R1-R9) of 16 different time points. The scale bar is 200 μm.

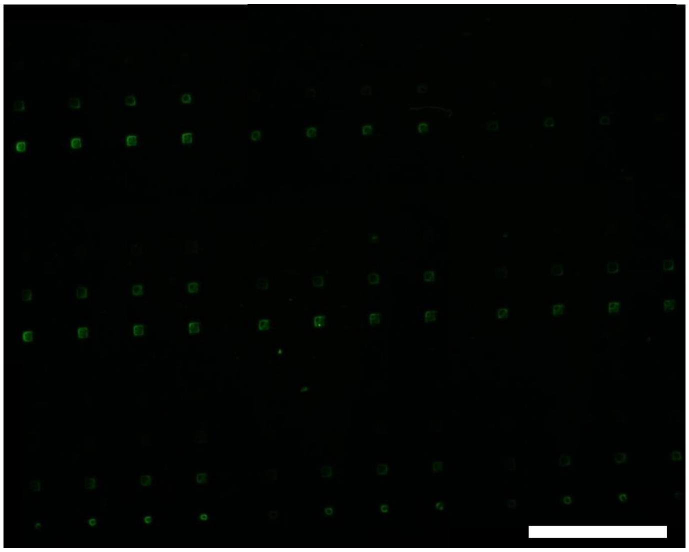

Figure S194. Fluorescence image of patterns printed using [TPO] = 100 mM, [EGDMA] = 1300 mM. Incubation was performed for 1 hour. The pattern printed is 9 repeats (R1-R9) of 16 different time points. The scale bar is 200 μm.

Table S113. Fluorescence intensity (*I*) and heights (*h*) of features printed under conditions in **Table S112**, and shown in **Figure S193** and **Figure S194**, # = feature number

#	Time (min)	R1	*I*	R2	*I*	R3	*I*	R4	*I*	R5	*I*	R6	*I*	R7	*I*	R8	*I*	R9	*I*	Average Height (μm)
1.0	3.5	N/A	0.7	N/A	1.0	N/A	0.9	N/A	0.9	N/A	1.0	N/A	1.0	N/A	0.8	3.2	1.1	N/A	1.0	3.2
2.0	4.0	N/A	0.9	N/A	1.0	N/A	0.8	N/A	1.0	N/A	1.0	N/A	0.9	N/A	0.8	N/A	1.2	N/A	1.2	N/A
3.0	4.5	N/A	1.2	N/A	1.3	N/A	1.5	N/A	1.3	N/A	1.0	N/A	1.0	N/A	0.8	N/A	0.9	N/A	1.0	N/A
4.0	5.0	N/A	1.2	N/A	1.0	N/A	0.9	N/A	1.1	N/A	0.9	N/A	0.8	N/A	1.0	N/A	0.9	N/A	0.7	N/A
5.0	5.5	N/A	0.9	0.8	0.9	N/A	1.3	0.2	1.0	N/A	1.0	N/A	1.2	1.4	0.9	0.8	0.9	1.6	0.8	1.0
6.0	6.0	1.7	1.0	N/A	0.9	N/A	0.9	1.7	1.2	1.9	1.0	N/A	1.0	1.9	0.9	2.1	0.8	1.0	0.9	1.7
7.0	6.5	3.1	1.1	0.0	1.2	N/A	1.2	2.7	1.3	5.4	1.9	N/A	0.9	2.6	1.2	2.1	0.9	2.1	0.7	2.6
8.0	7.0	2.5	1.2	N/A	1.0	N/A	0.9	3.4	1.6	3.3	1.1	N/A	0.9	3.7	1.5	2.0	1.1	1.6	0.7	2.8
9.0	7.5	3.4	2.7	3.9	1.3	0.1	1.0	3.9	2.7	3.9	1.9	2.4	1.5	3.5	2.3	5.6	1.4	4.8	1.3	3.5
10.0	8.0	9.0	2.9	4.0	1.7	N/A	1.4	4.4	3.5	6.4	2.6	2.9	1.8	6.3	3.3	5.8	2.4	4.3	2.0	5.4
11.0	8.5	10.6	3.6	5.0	1.9	N/A	1.3	6.9	4.4	8.5	3.0	3.9	2.1	7.8	3.8	6.4	3.0	5.9	2.2	6.9
12.0	9.0	7.4	4.2	7.2	2.0	N/A	0.8	10.1	4.9	10.4	3.3	4.5	2.3	7.6	4.2	8.9	2.9	7.0	2.4	7.9
13.0	9.5	4.4	8.3	12.0	4.6	4.0	2.4	1.9	6.9	9.4	5.1	9.1	3.4	4.1	2.2	8.6	1.4	9.3	1.4	7.0
14.0	10.0	7.2	7.8	11.0	5.1	6.5	2.8	5.7	6.1	11.8	6.2	8.2	3.7	5.5	4.3	10.4	3.2	8.3	2.8	8.3
15.0	10.5	7.6	7.7	11.6	5.1	6.0	2.4	7.1	6.3	12.9	4.3	9.8	4.2	7.0	4.7	9.1	3.7	10.7	2.8	9.1
16.0	11.0	5.7	6.8	13.4	4.8	N/A	1.3	8.1	6.3	13.6	4.0	10.0	3.0	5.6	4.6	11.3	3.3	11.3	1.0	9.9

Table S114. Binding conditions for surfaces printed under conditions shown in **Table S13**.

TPO (mM)	PETT (mM)	EGDMA (mM)	Intensity (mW/mm^2)	[**SCR043**] (μM)	[**β-Gluc-FL**] (M)
1	100	1300	2.53	100	$10^{-5.5}$

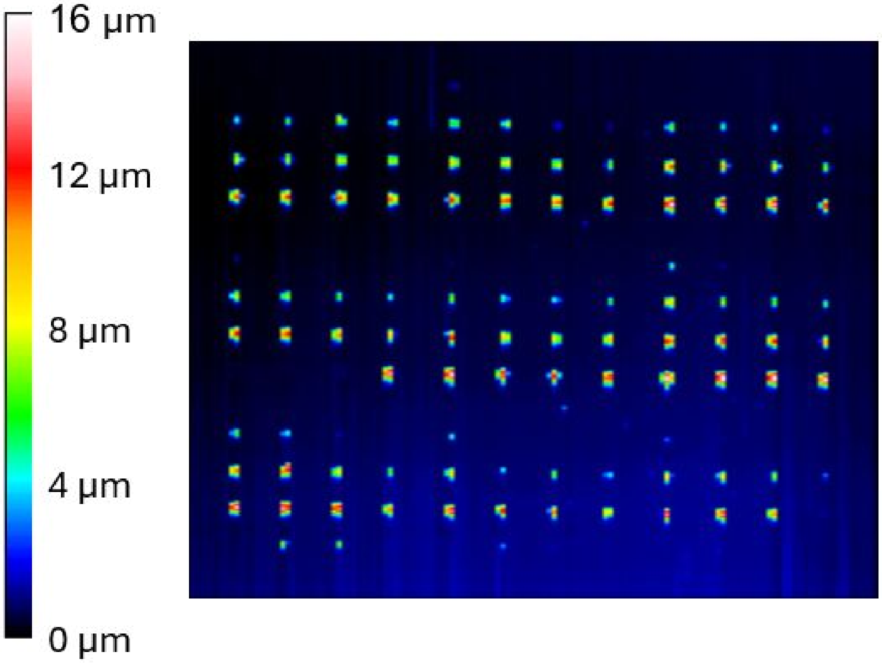

Figure S195. Profilometry image of patterns printed using [TPO] = 100 mM, [EGDMA] = 1300 mM. The pattern printed is 9 repeats (R1-R9) of 16 different time points. The scale bar is 200 μm.

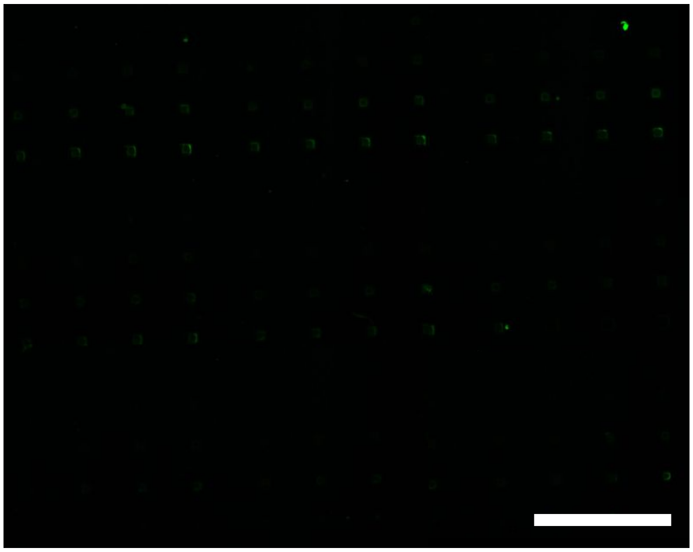

Figure S196. Fluorescence image of patterns printed using [TPO] = 100 mM, [EGDMA] = 1300 mM. Incubation was performed for 1 hour. The pattern printed is 9 repeats (R1-R9) of 16 different time points. The scale bar is 200 μm.

Table S115. Fluorescence intensity (*I*) and heights (*h*) of features printed under conditions in **Table S114**, and shown in **Figure S195** and **Figure S196**, # = feature number

Table S105. Binding conditions for surfaces printed under conditions shown in **Table S13**.

TPO (mM)	PETT (mM)	EGDMA (mM)	Intensity (mW/mm^2)	[**SCR043**] (μM)	[**β-Gal-FL**] (M)
1	100	1300	2.53	100	10^{-3}

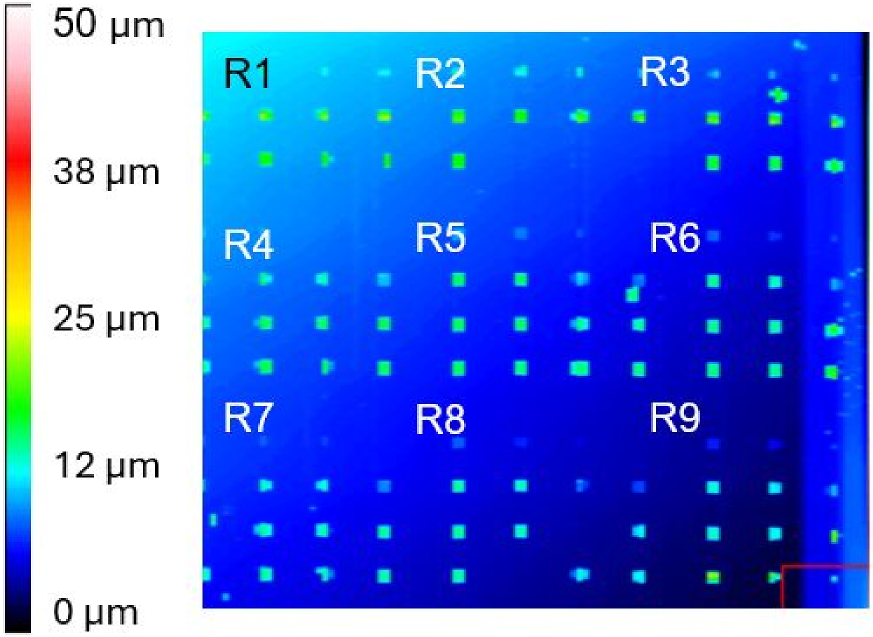

Figure S197. Profilometry image of patterns printed using [TPO] = 100 mM, [EGDMA] = 1300 mM. The pattern printed is 9 repeats (R1-R9) of 16 different time points. The scale bar is 200 µm.

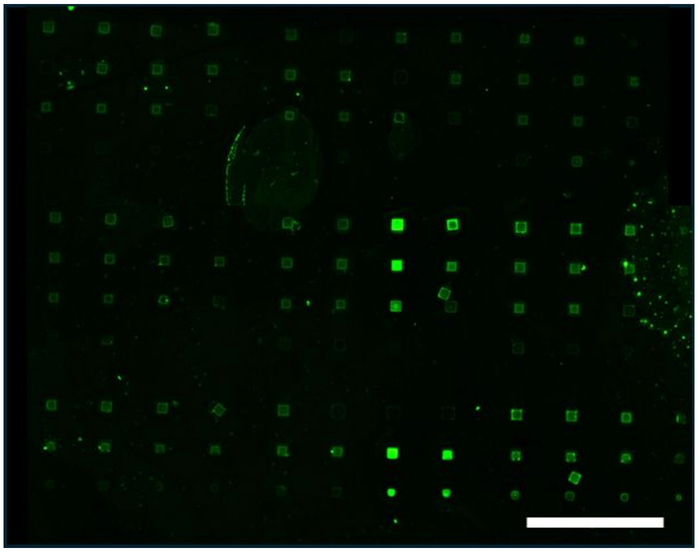

Figure S198. Fluorescence image of patterns printed using [TPO] = 100 mM, [EGDMA] = 1300 mM. Incubation was performed for 1 hour. The pattern printed is 9 repeats (R1-R9) of 16 different time points. The scale bar is 200 µm.

Table S116, Fluorescence intensity (*I*) and heights (*h*) of features printed under conditions in **Table S115**, and shown in **Figure S197** and **Figure S198**, # = feature number.

#	Time (min)	R1	*I*	R2	*I*	R3	*I*	R4	*I*	R5	*I*	R6	*I*	R7	*I*	R8	*I*	R9	*I*	Average Height (µm)
1	3.5	N/A	0.8	1.7	1.2	1.8	0.8	N/A	1.1	2.5	1.4	2.7	2.1	N/A	N/A	2.9	1.1	3.2	0.8	N/A
2	4	N/A	0.9	1.1	1.1	1.5	0.8	N/A	1.8	1.7	1.3	1.8	1.4	N/A	N/A	2.2	0.8	3.0	1.5	N/A
3	4.5	N/A	1.3	N/A	1.9	1.1	0.9	N/A	1.0	1.3	1.0	N/A	1.5	N/A	N/A	1.6	0.8	3.0	0.7	N/A
4	5	N/A	1.2	N/A	0.9	N/A	0.7	N/A	1.0	N/A	0.7	N/A	2.5	N/A	N/A	1.6	0.4	N/A	N/A	N/A
5	5.5	N/A	1.3	7.3	2.0	7.8	3.4	3.9	2.1	8.4	2.5	9.1	3.6	3.7	1.5	9.7	2.7	10.0	1.6	N/A
6	6	2.3	1.6	6.9	1.7	7.8	2.6	6.0	2.1	8.8	2.8	9.0	3.7	3.3	1.7	10.2	1.6	10.0	1.5	7.1
7	6.5	1.8	1.8	7.2	6.3	7.7	2.7	5.2	2.7	5.8	6.2	6.8	2.1	3.3	1.8	4.3	2.4	4.8	0.9	5.2
8	7	1.9	1.5	4.6	5.3	4.5	2.1	3.0	1.5	9.0	2.3	3.9	3.5	1.0	1.2	6.2	0.9	2.0	N/A	4.0
9	7.5	14.0	4.2	4.8	4.7	7.9	5.5	10.1	2.4	8.8	3.0	9.8	4.5	12.9	0.7	10.1	2.3	10.8	1.7	9.9
10	8	11.0	4.3	8.0	5.1	8.5	5.4	9.1	2.8	8.9	3.5	9.5	5.2	14.5	2.2	10.2	2.1	11.0	1.6	10.1
11	8.5	11.5	3.1	8.3	14.3	8.6	5.5	11.9	2.8	9.4	10.5	N/A	6.4	14.1	2.3	12.9	0.8	14.5	1.5	11.4
12	9	12.6	3.2	8.4	9.1	9.0	2.4	13.2	2.4	9.6	4.0	10.2	3.9	4.7	2.0	6.9	1.6	3.8	N/A	8.7
13	9.5	3.8	3.9	8.0	4.4	8.5	6.9	8.9	2.5	9.4	4.4	9.7	6.4	10.0	2.0	10.6	1.7	19.8	1.8	9.8
14	10	7.5	4.1	8.1	1.9	8.8	6.4	N/A	3.6	9.3	3.0	N/A	5.3	10.1	2.2	10.9	0.8	17.7	1.5	N/A
15	10.5	7.6	3.8	8.3	1.3	9.0	6.0	N/A	3.5	10.0	14.1	10.1	5.1	10.6	2.2	12.5	1.8	7.3	0.6	N/A
16	11	8.1	4.0	8.7	1.2	9.4	3.3	N/A	0.9	10.1	6.9	10.3	3.7	7.5	2.4	7.6	1.4	N/A	N/A	N/A

Table S117. Binding conditions for surfaces printed under conditions shown in **Table S13**.

TPO (mM)	PETT (mM)	EGDMA (mM)	Intensity (mW/mm^2)	[**SCR043**] (μM)	[**β-Gal-FL**] (M)
1	100	1300	2.53	100	$10^{-3.5}$

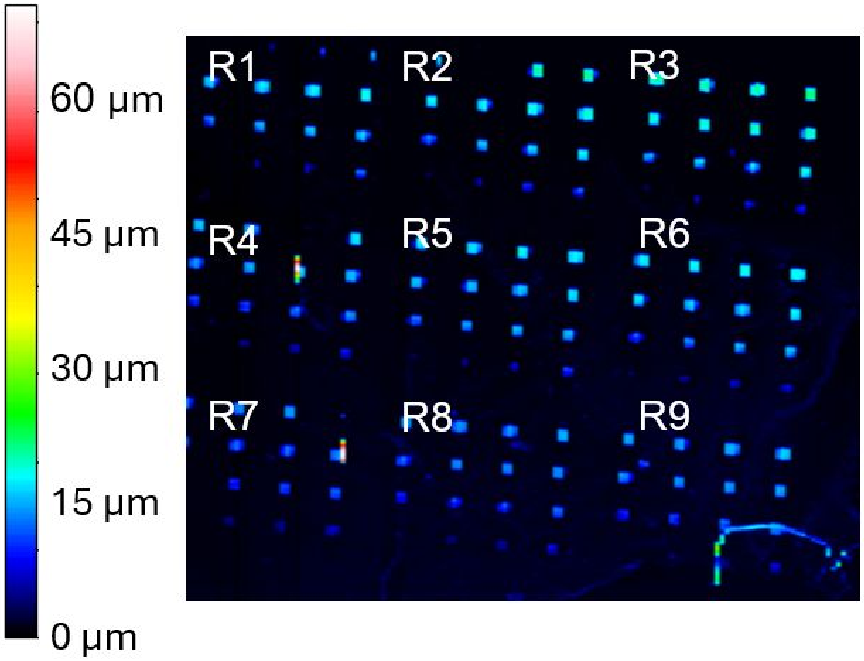

Figure S199. Profilometry image of patterns printed using [TPO] = 100 mM, [EGDMA] = 1300 mM. The pattern printed is 9 repeats (R1-R9) of 16 different time points. The scale bar is 200 μm.

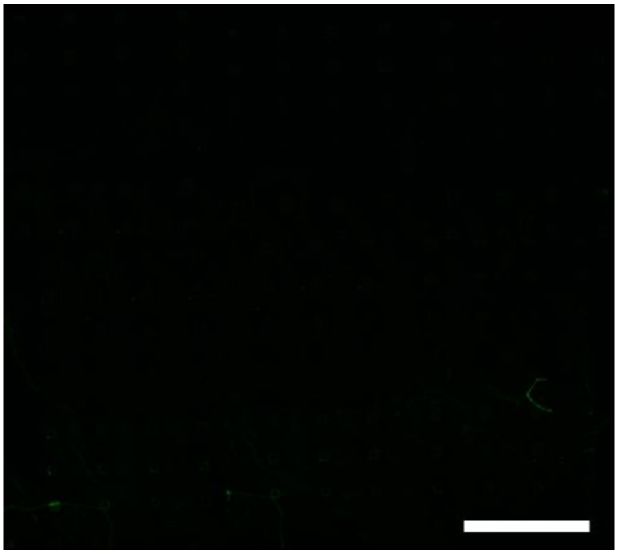

Figure S200. Fluorescence image of patterns printed using [TPO] = 100 mM, [EGDMA] = 1300 mM. Incubation was performed for 1 hour. The pattern printed is 9 repeats (R1-R9) of 16 different time points. The scale bar is 200 μm.

Table S118. Fluorescence intensity (*I*) and heights (*h*) of features printed under conditions in **Table S117**, and shown in **Figure S199** and **Figure S200**, # = feature number

Table S119. Binding conditions for surfaces printed under conditions shown in **Table S13**.

TPO (mM)	PETT (mM)	EGDMA (mM)	Intensity (mW/mm^2)	[**SCR043**] (µM)	[**β-Gal-FL**] (M)
1	100	1300	2.53	100	10^{-4}

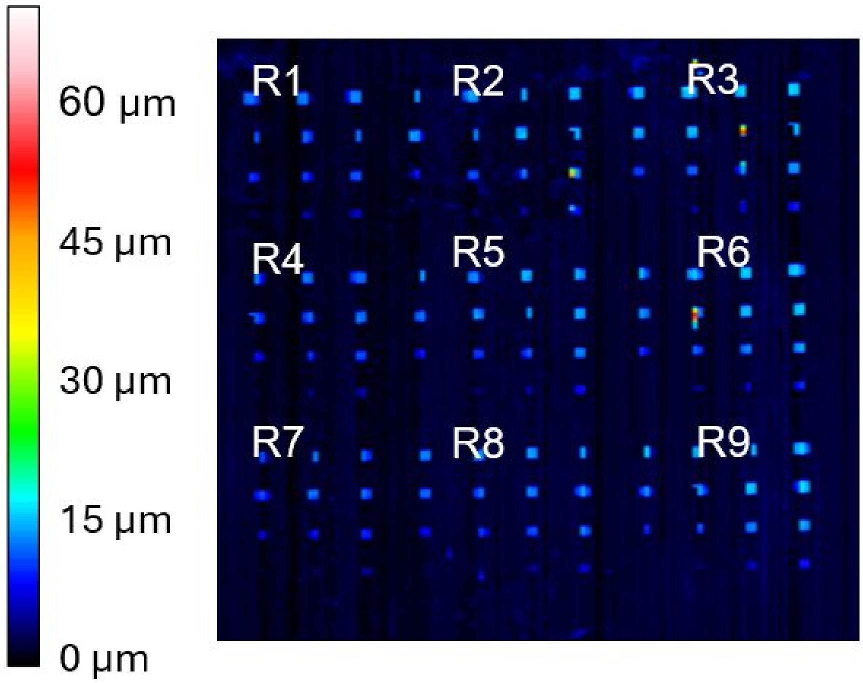

Figure S201. Profilometry image of patterns printed using [TPO] = 100 mM, [EGDMA] = 1300 mM. The pattern printed is 9 repeats (R1-R9) of 16 different time points. The scale bar is 200 µm.

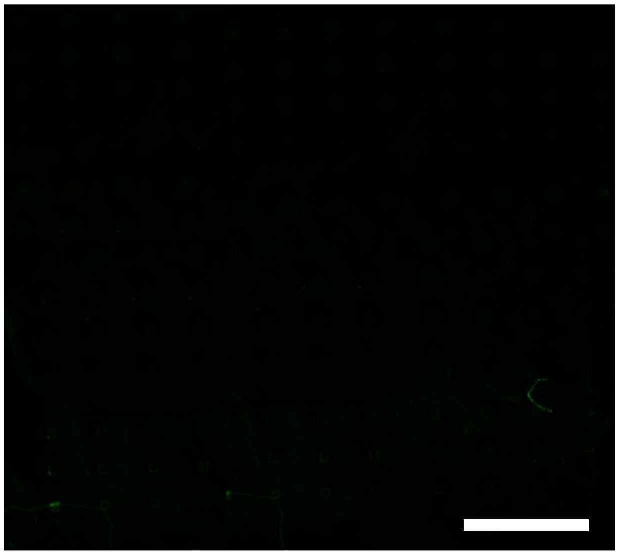

Figure S202. Fluorescence image of patterns printed using [TPO] = 100 mM, [EGDMA] = 1300 mM. Incubation was performed for 1 hour. The pattern printed is 9 repeats (R1-R9) of 16 different time points. The scale bar is 200 µm.

Table S120. Fluorescence intensity (*I*) and heights (*h*) of features printed under conditions in **Table S119**, and shown in **Figure S201** and **Figure S202**, # = feature number

8. Determination of K_ds

Here we report multiple combinations of I_{max} which were used to calculate K_ds. This is based on the 3 different variables being changed during the experiment, – *h*, [**SCR043**] in the printing solution, and [glycan] in the binding solution. They report as follows – I_{max}(h, Γ[**SCR043**], [glycan]) where underline indicates which parameter was varied in the determination of the particular Imax. Whereas I_{max}(h, Γ[**SCR043**], [glycan]) correspond to the maximum *I* observed when all heights and [sugar] are all equal. By segmenting the data like this we can draw different interpretations of our binding models by comparing the different trends. From the fluorescence data, we determined quantitatively the K_d values between **α-Man-FL** and the **SCR043-**functionalized polymer brushes, using the Langmuir isotherm model[10-16] to determine the K_d of binding events on the surface using the fluorescence data from each channel of the incubation mask.

$$\boldsymbol{I} = \frac{[\boldsymbol{L}]\boldsymbol{I_{max}}}{[\boldsymbol{L}]+\boldsymbol{K_d}} \qquad \textbf{(Eq. S1.)}$$

where [L] is the concentration of the **SCR043**, *I* is the median fluorescence counts of a particular feature, and I_{max} is the maximum fluorescence observed when **α-Man-FL** binds to a **SCR043** containing polymer of a particular height.

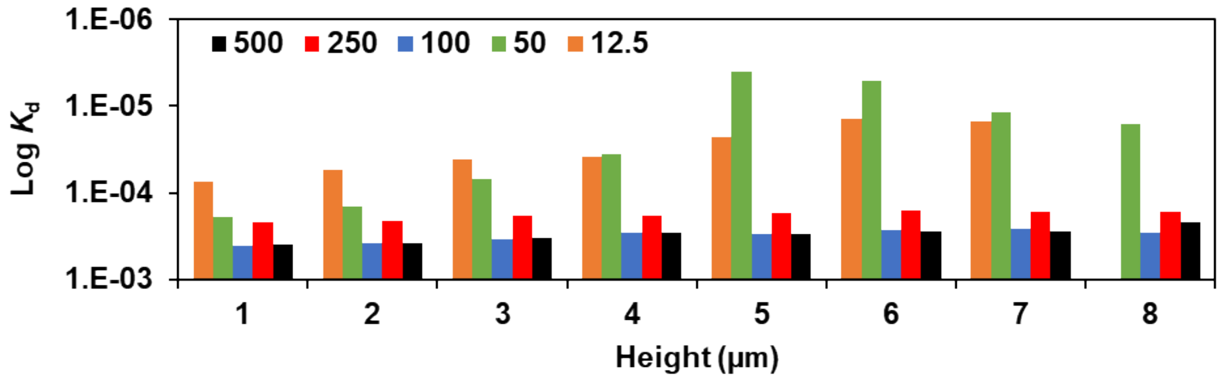

Figure S203. Graph of K_d(h, Γ[**SCR043**], [glycan] for 10^{-4} M [**a-Man-FL**]

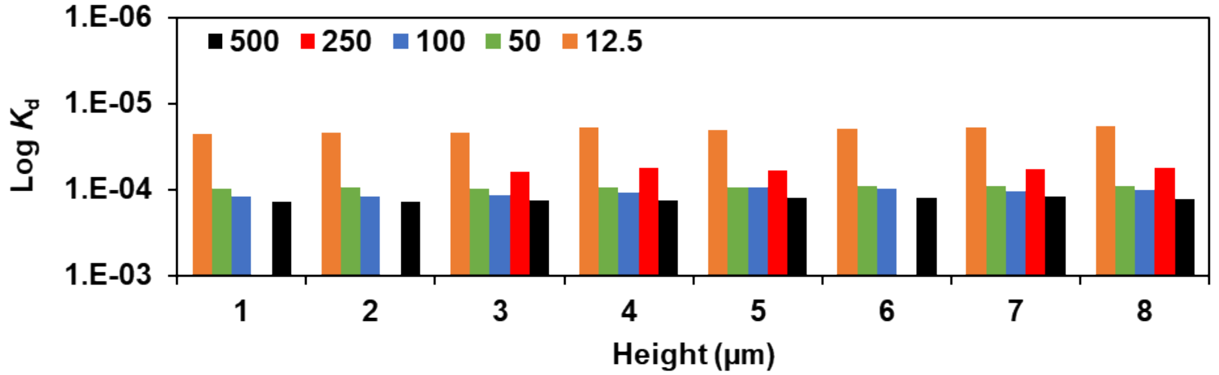

Figure S204. Graph of K_d(h, Γ[**SCR043**], [glycan] for $10^{-4.5}$ M [**a-Man-FL**]

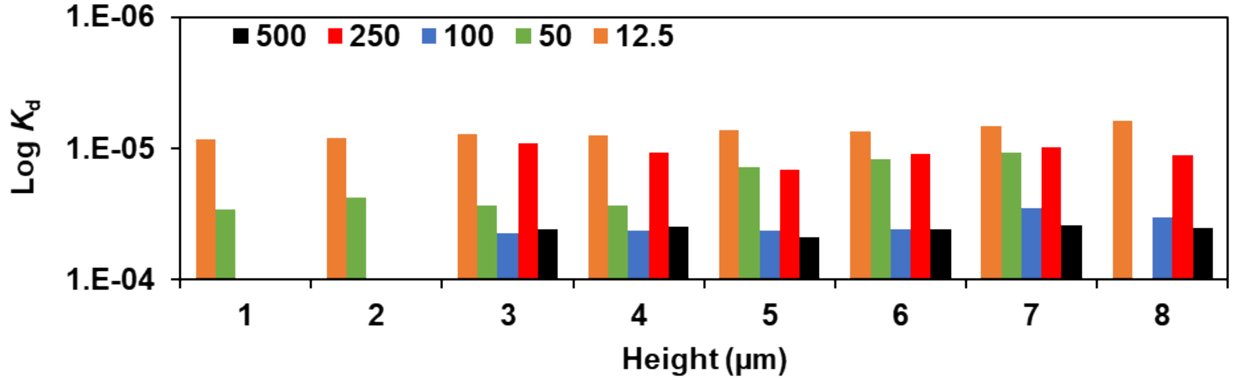

Figure S205. Graph of K_d(h, Γ[**SCR043**], [glycan] for 10^{-5} M [**a-Man-FL**]

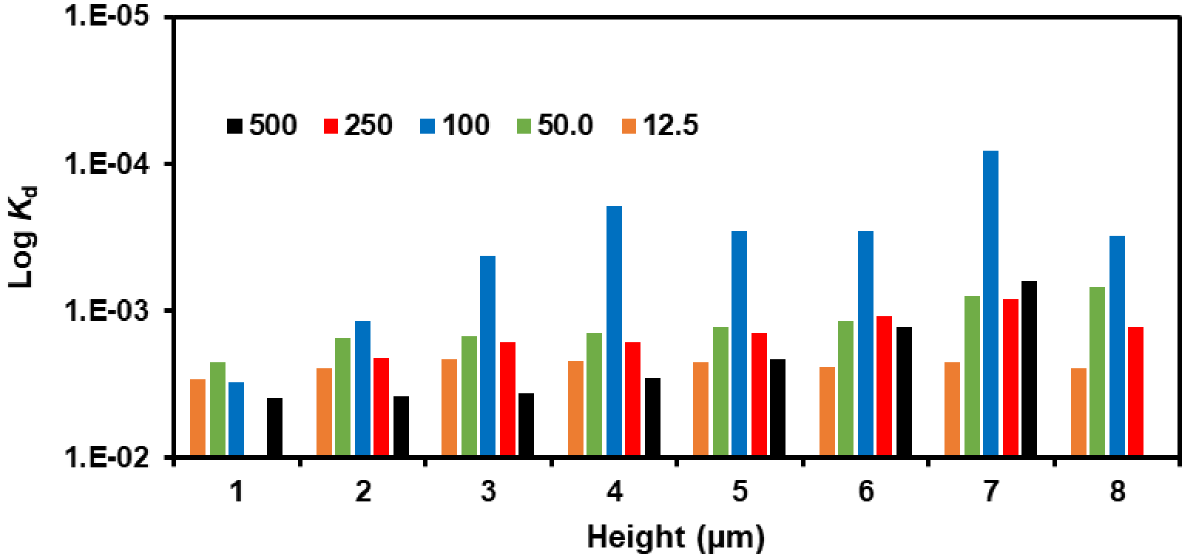

Figure S206. I_{max}(h, Γ[**SCR043**], [glycan]) – observed when none of the values are equal at a single point.

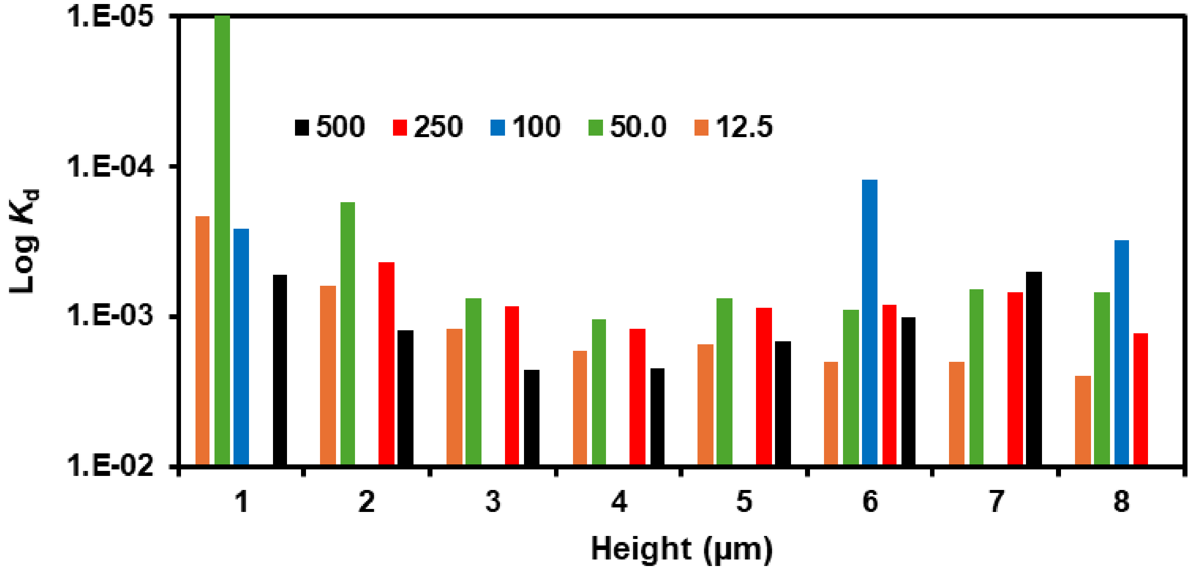

Figure S207. I_{max}(h, Γ[**SCR043**], [glycan]) – corresponds to the maximum *I* observed when all heights in the virtual array are equal.

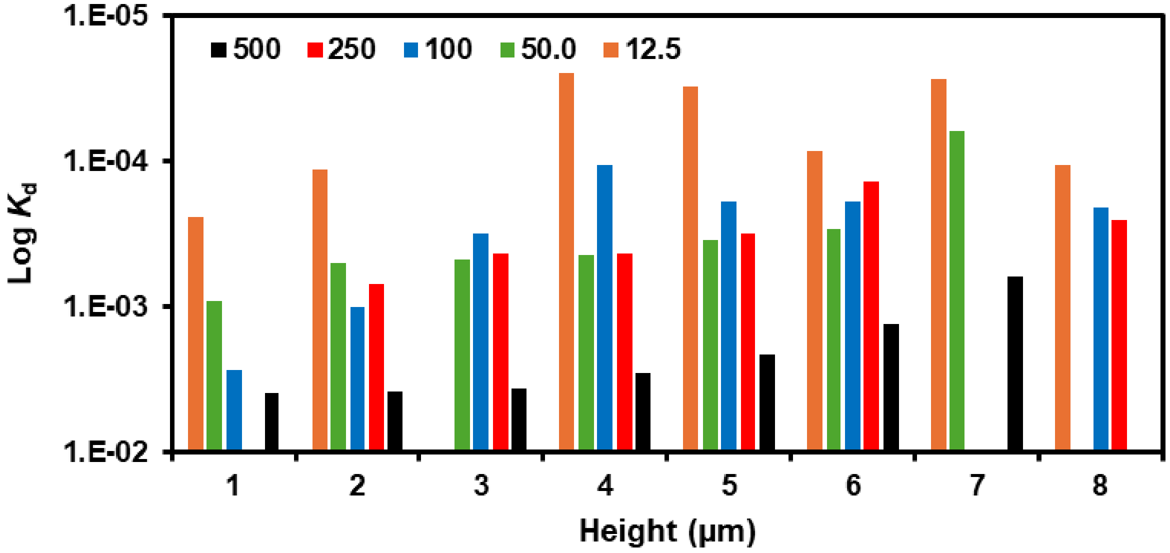

Figure S208. I_{max}(h, Γ[**SCR043**], [glycan]) – observed when [**SCR043**] and [sugar] are equal at a single point.

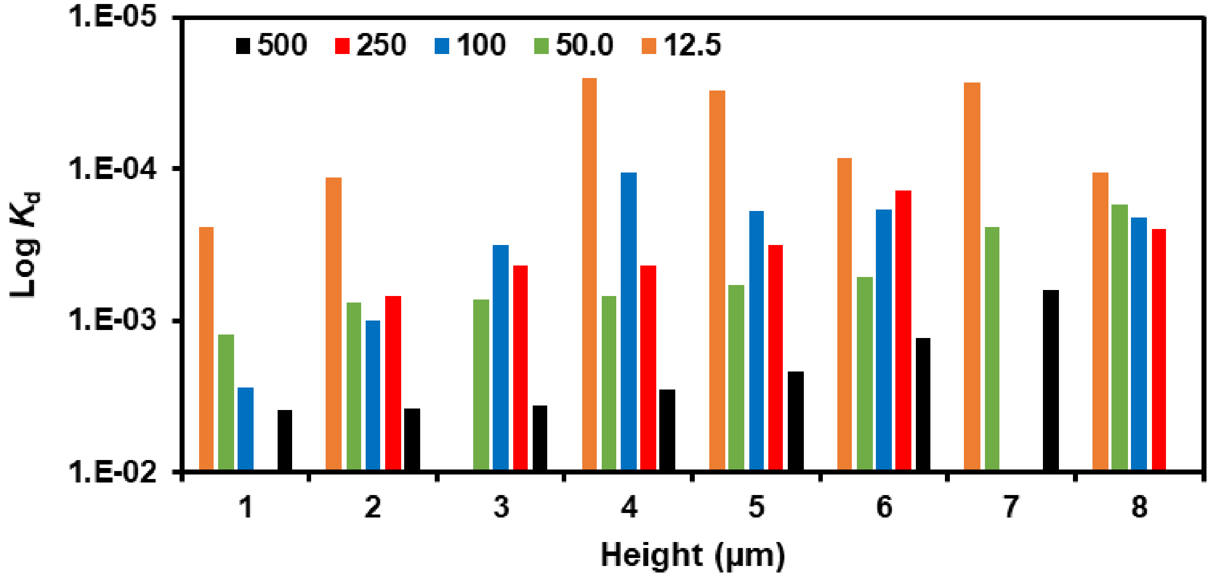

Figure S209. I_{max}(h, Γ[**SCR043**], [glycan]) – observed when [**SCR043**] is equal at a single point.

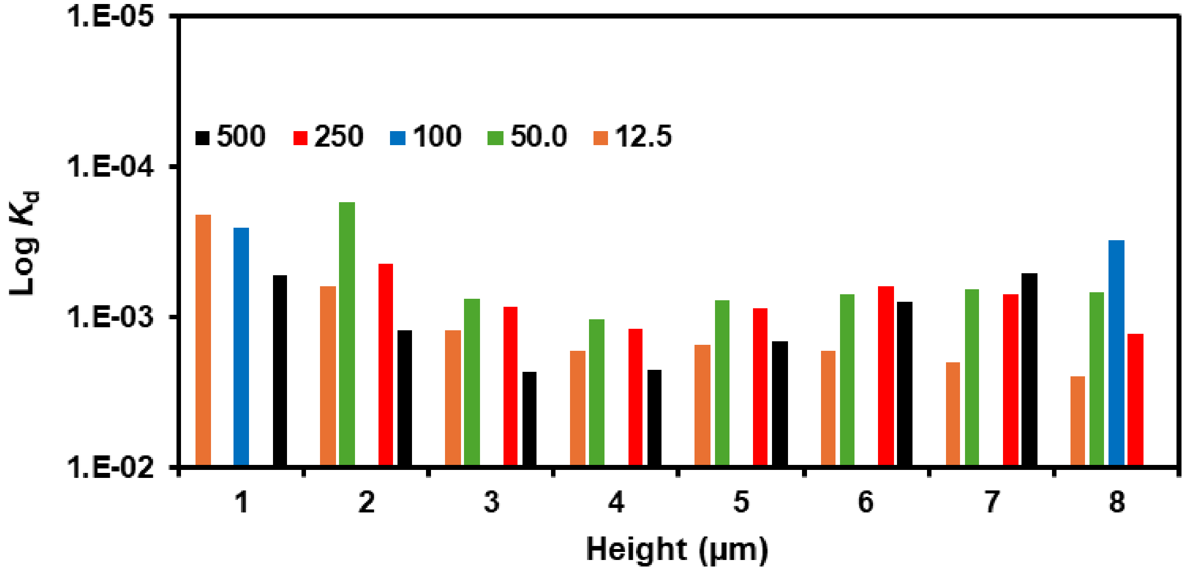

Figure S210. I_{max}(h, Γ[**SCR043**], [glycan]) – observed when *h* and [sugar] are equal at a single point.

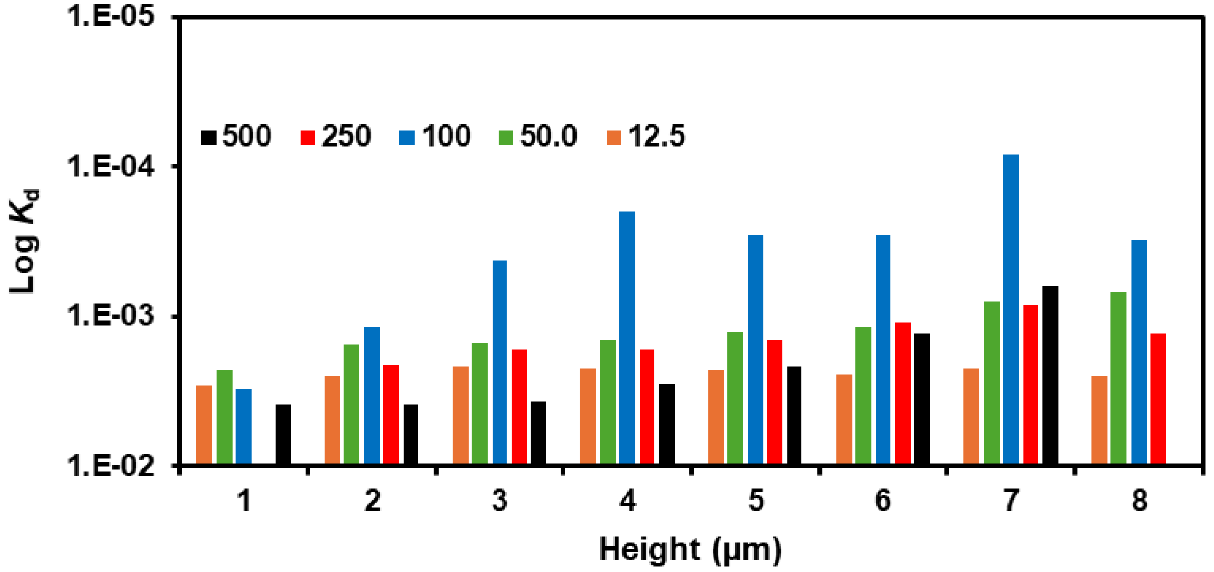

Figure S211. I_{max}(h, Γ[**SCR043**], [glycan]) – observed when [sugar] is equal at a single point.

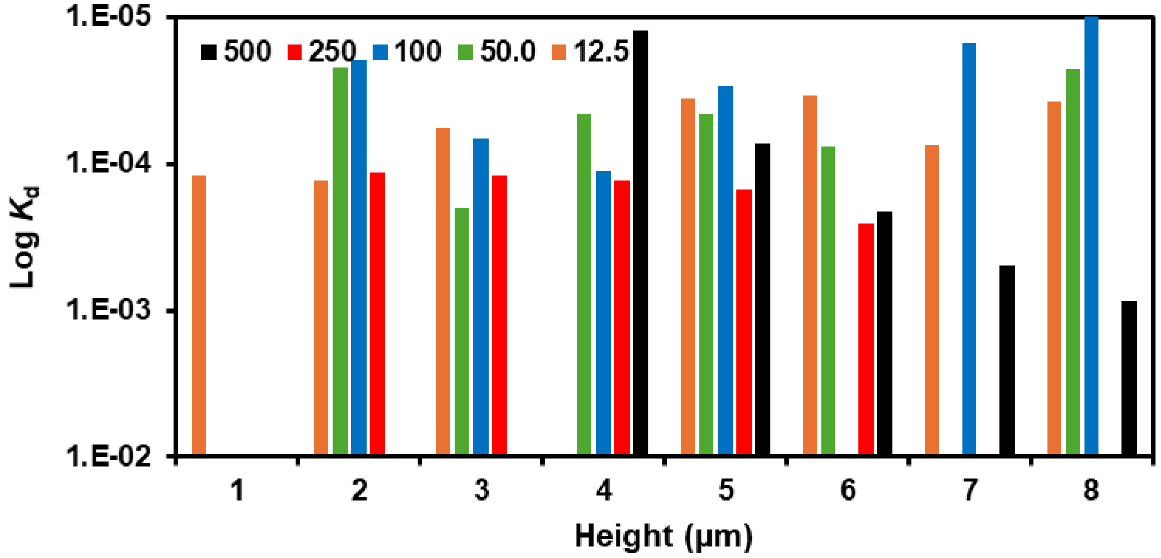

Figure S212. I_{max}(h, Γ[**SCR043**], [glycan]) – observed when *h* and [**SCR043**] are equal at a single point.

9. Determination of Hill coefficients (H_c)

The data were fit to a 4-parameter logistic (4PL) binding isotherm that was calculated by the following equation:[10]

$$H_c = \log\left(\frac{2I_{min} - I_{max} - I}{\frac{EC_{50}}{L}}\right) \qquad \textbf{(Eq. S2.)}$$

where I_{max} is the maximum normalized fluorescence value, I_{min} is the minimum normalized fluorescence value, EC_{50} is the concentration of **α-Man-FL** that is half of the I_{max}, [L] is the concentration of **α-Man-FL**, y is the *I* output dependent on **α-Man-FL** and the Hill coefficient (H_c), which is a constant that dictates whether binding is negatively or positively cooperative. The values for I_{max}(h, Γ[**SCR043**], [glycan] were found in **Table S98** were used for this fitting which was performed with an excel calculator.[11]

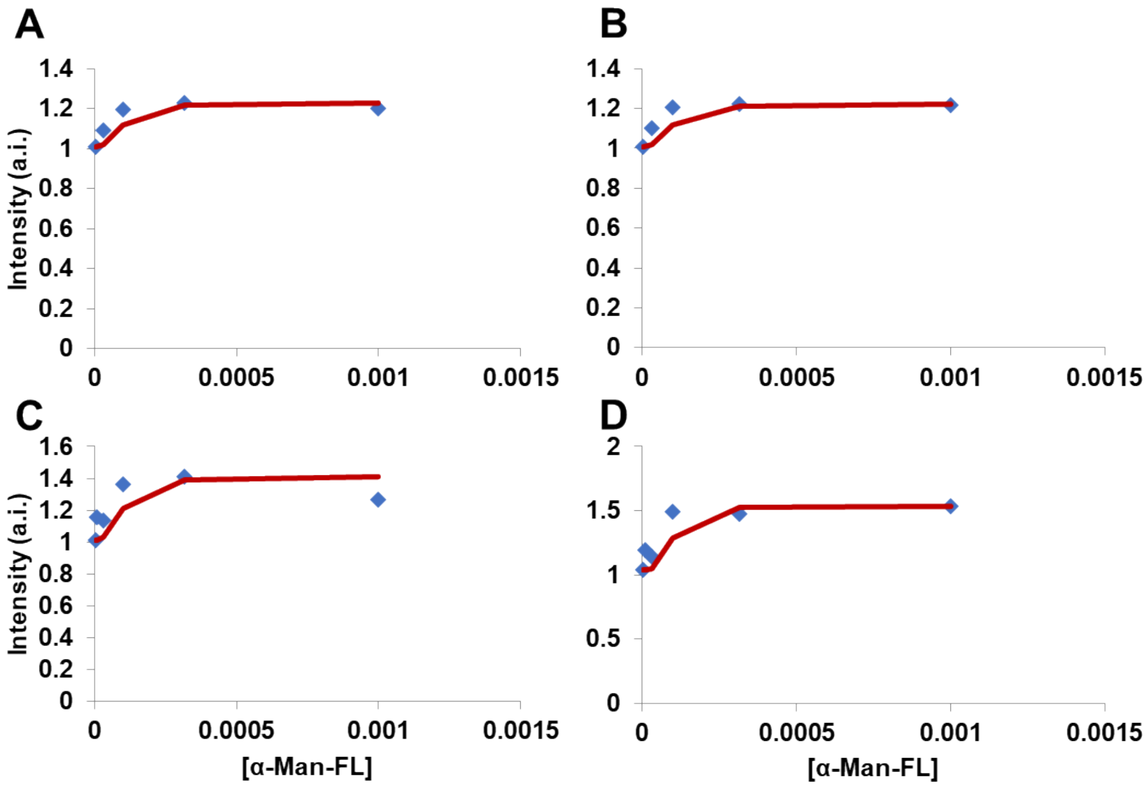

Figure S213. Hill plots for **SCR043** 500 μM for (A) 1, (B) 2, (C) 3, and (D) 4 μm.

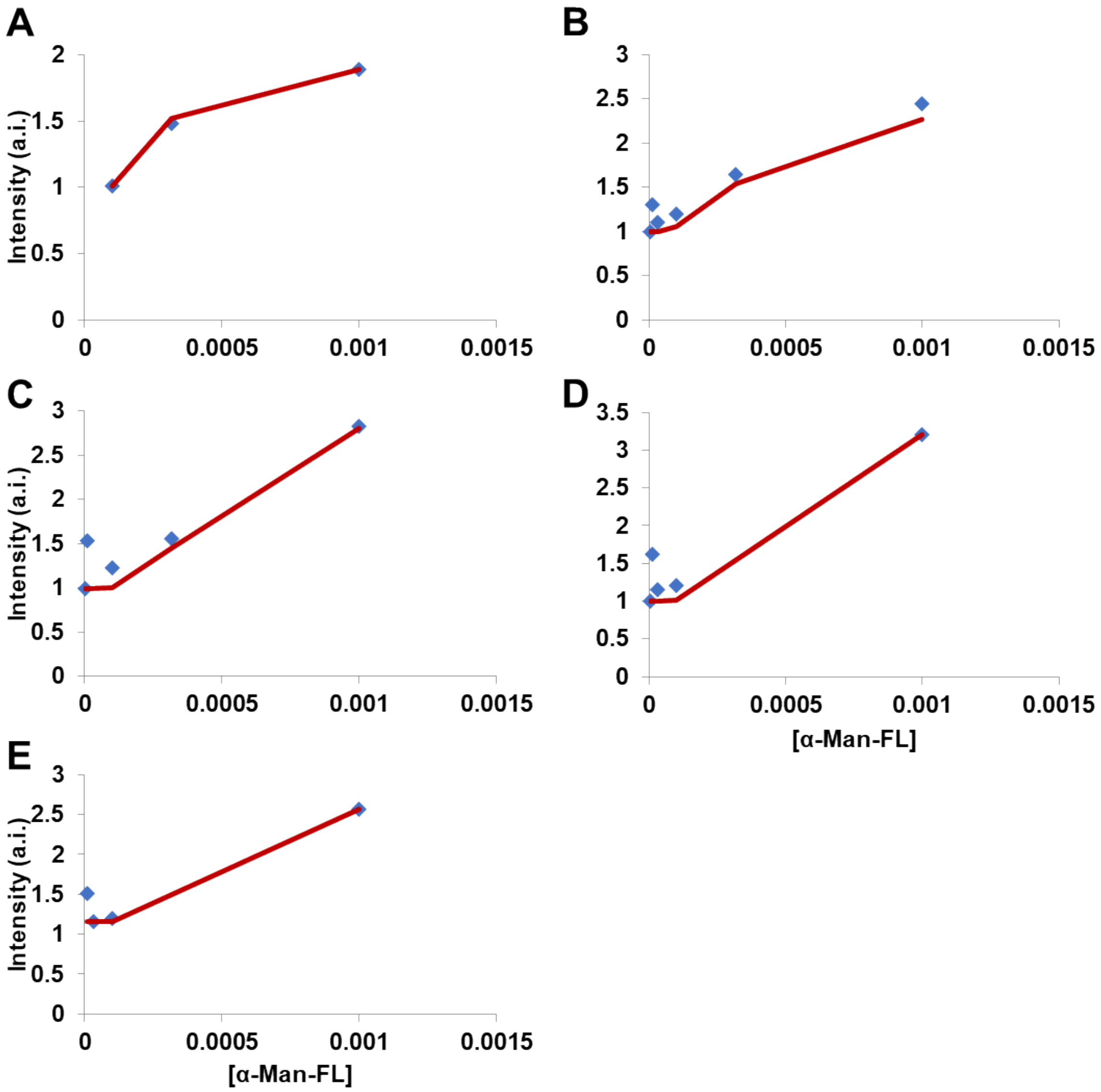

Figure S214. Hill plots for **SCR043** 250 μM for (A) 1, (B) 5, (C) 6, (D) 7, and (E) 8 μm.

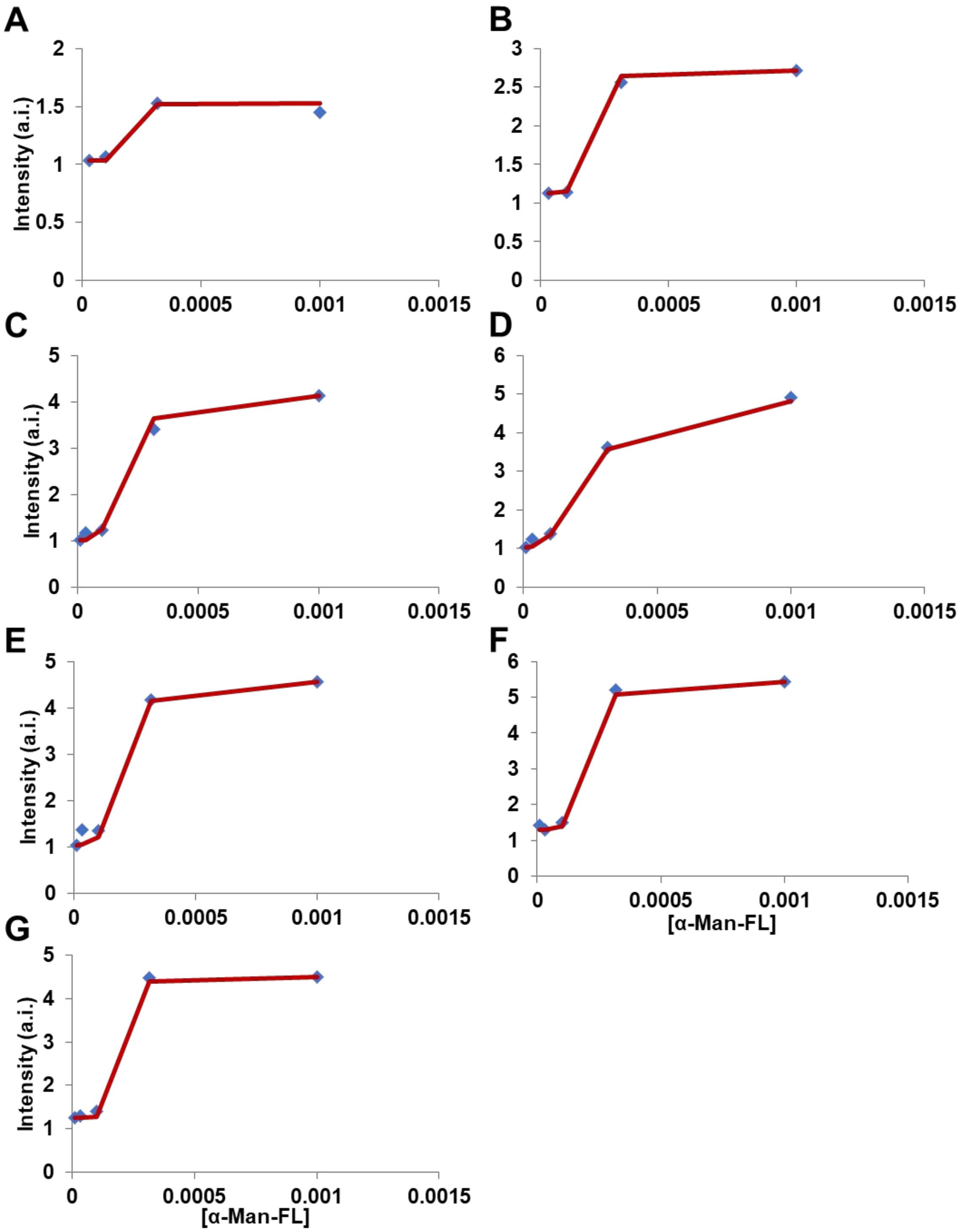

Figure S215. Hill plots for **SCR043** 100 μM for (A) 1, (B) 2, (C) 3, (D) 4, (E) 5, (F) 7, and (G) 8 μm.

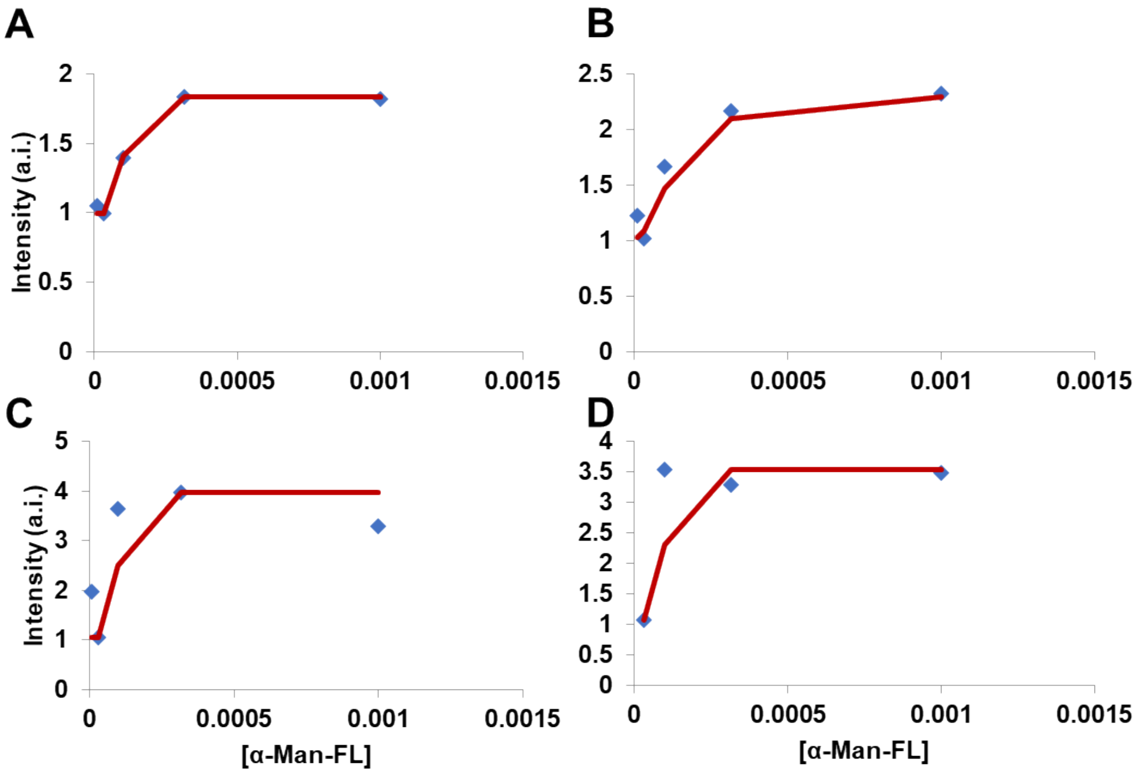

Figure S216. Hill plots for **SCR043** 50 μM for (A) 1, (B) 2, (C) 7, and (D) 8 μm.

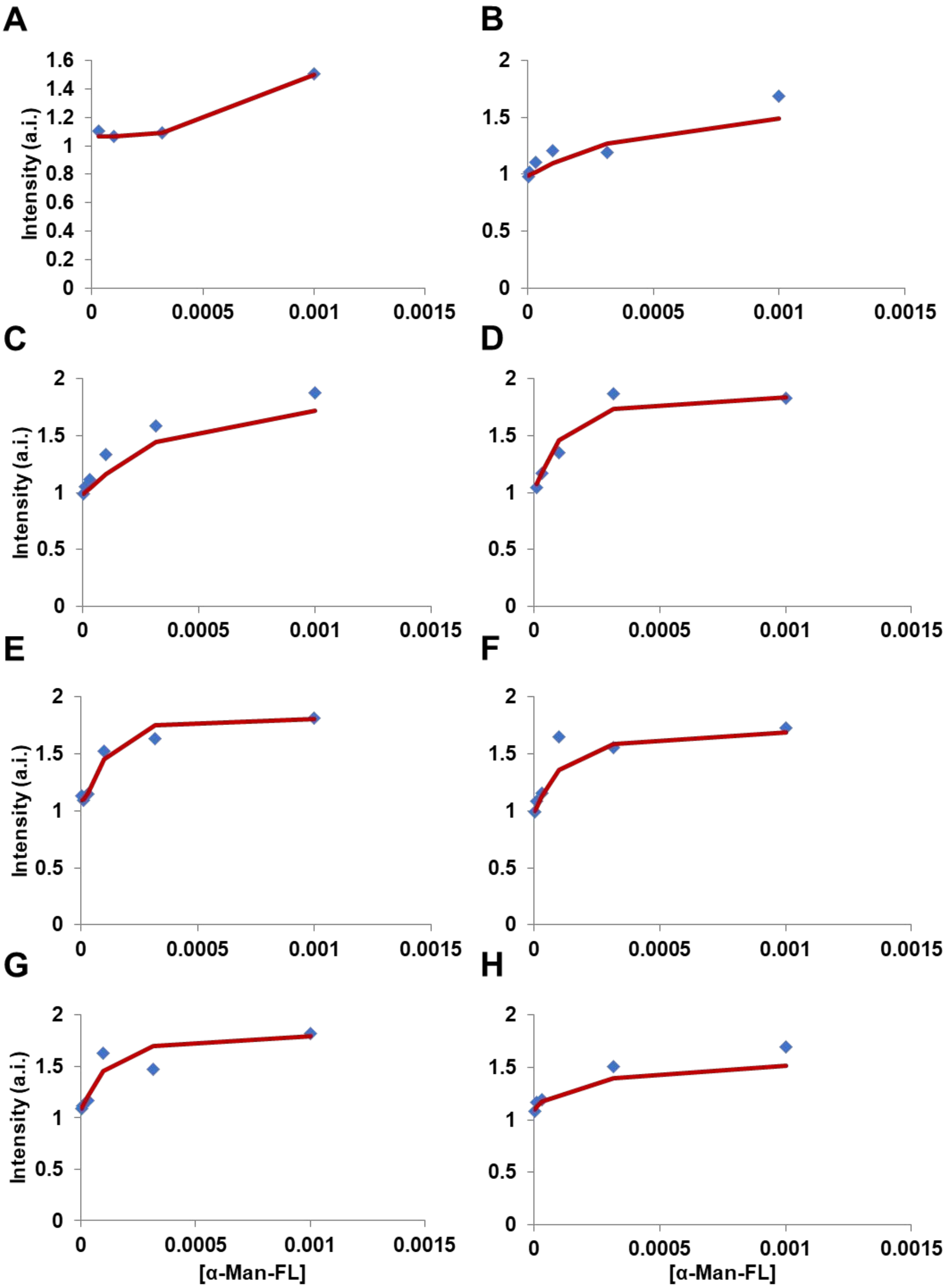

Figure S217. Hill plots for **SCR043** 12.5 µM for (A) 1, (B) 2, (C) 3, (D) 4, (E) 5, (F) 6, (G) 7, and (h) 8 µm.

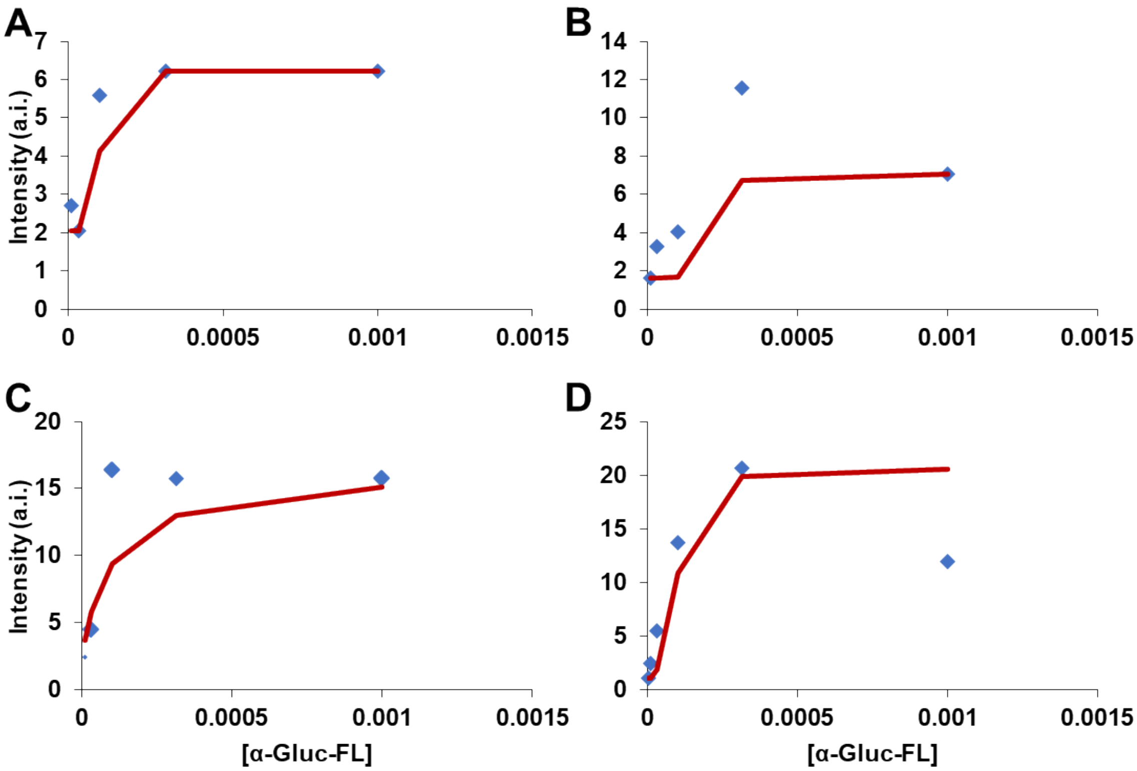

Figure S218. Hill plots for **SCR043** 100 µM for (A) 4, (B) 5, (C) 7, and (D) 8 µm and **α-Gluc-FL**.

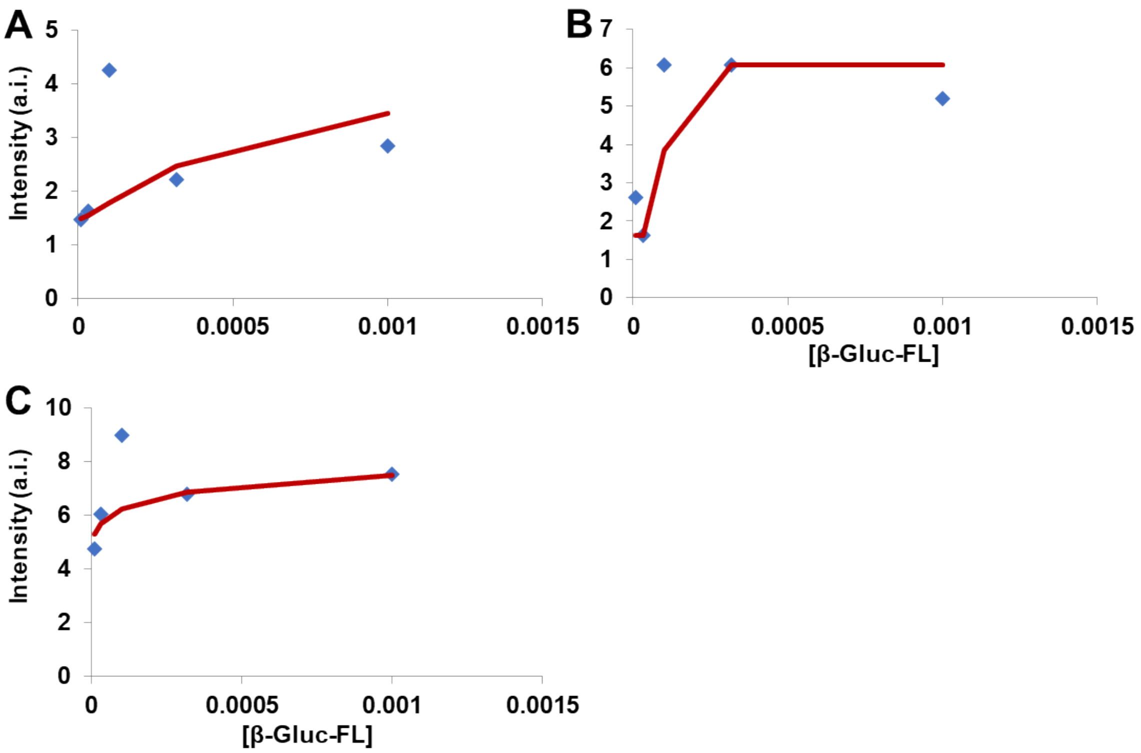

Figure S219. Hill plots for **SCR043** 100 µM for (A) 3, (B) 4, and (C) 8 µm and **β-Gluc-FL**.

Table S121. Table of Hill coefficients (H_c) reported for the various concentrations of **SCR043**-functionalized polymer brushes and their corresponding R^2 values.

**SCR043** / **Height**	500 µM	R^2	250 µM	R^2	100 µM	R^2	50 µM	R^2	12.5 µM	R^2
8 µm	–	–	6.80	0.99	7.46	1.00	8.84	0.94	0.75	0.99
7 µm	–	–	7.41	0.95	5.18	1.00	14.04	0.88	1.41	0.96
6 µm	–	–	4.74	0.95	–	–	–	–	1.23	0.96
5 µm	–	–	2.20	0.95	4.40	0.99	–	–	1.97	0.99
4 µm	5.42	0.96	–	–	2.64	1.00	–	–	1.43	0.99
3 µm	2.65	0.95	–	–	3.60	0.99	–	–	1.28	0.97
2 µm	2.65	0.99	2.20	1.00	6.48	1.00	1.93	0.99	1.14	0.96
1 µm	2.56	1.00	6.00	1.00	12.38	0.97	7.62	1.00	5.99	0.99

Note only values where R^2 is greater than 0.94 are shown. Incubations performed in Tris buffer 20 mM, $MnCl_2$ 0.9 mM, $CaCl_2$ 0.5 mM, pH = 7.4, 0.01% Tween20.**–** means a fit ≥ 0.94 R^2 was not achieved.

Table S122. Table of Hill coefficients (H_c) reported for the various concentrations of **SCR043**-functionalized polymer brushes and their corresponding R^2 values.

Glycan	α-Man-FL			α-Gluc-FL			β-Gluc-FL		
**Height (μm)**	**IC_{50} (μM)**	**H_c**	**R^2**	**IC_{50} (μM)**	**H_c**	**R^2**	**IC_{50} (μM)**	**H_c**	**R^2**
**1**	**200**	**12.4**	**0.97**	**–**	**–**	**–**	**–**	**–**	**–**
**2**	**200**	**6.5**	**1**	**–**	**–**	**–**	**–**	**–**	**–**
**3**	**200**	**3.6**	**0.99**	**–**	**–**	**–**	**500**	**1.3**	**0.94**
**4**	**250**	**2.6**	**1**	**100**	**10.4**	**0.99**	**100**	**17.3**	**0.99**
**5**	**200**	**4.4**	**0.99**	**200**	**6.2**	**0.99**	**–**	**–**	**–**
**6**	**–**	**–**	**–**	**–**	**–**	**–**	**–**	**–**	**–**
**7**	**200**	**5.2**	**1**	**66**	**1.0**	**0.98**	**–**	**–**	**–**
**8**	**200**	**7.5**	**1**	**100**	**2.8**	**0.99**	**300**	**0.54**	**0.99**

Note only values where R^2 is greater than 0.94 are shown. Incubations performed in Tris buffer 20 mM, $MnCl_2$ 0.9 mM, $CaCl_2$ 0.5 mM, pH = 7.4, 0.01% Tween20. $\Gamma_{[scr] = 100}$. **–** means a fit ≥ 0.94 R^2 was not achieved.

10. Python code for profilometry and fluorescence data processing

A python code was developed to allow efficient data processing for both fluorescence and profilometry data. The fluorescence code allows the user to place a 52 x 47 box around a feature that they want to measure the fluorescence of. It then takes ['Num', 'Area', 'Mean', 'StdDev', 'Min', 'Max'] measurements using the numpy module with 'Num' corresponding to the feature number. After taking these measurements, it outputs them into an Excel spreadsheet of the user's choosing. Additionally, the user is able to input '# of features,' which is the number of entries after which the code will insert a break in Excel, signifying a new array of features. The Profilometry code works in a similar way, allowing the user to drag a rectangle around any feature that they would like to measure. Using the OPDX_reader file, the code displays a heatmap of the surface which allows the user to see where their features are. After a rectangle is dragged, the code will find the slice of the array of heights that goes through the highest point inside the rectangle. After this, the user must click where they would like the bottom of the feature to be recorded and the code will push ['Num', 'Top', 'Bottom', 'Difference'] into Excel in the same format as the fluorescence code. This program also allows the user to level their OPDX file. Both scripts can be found available for access at https://github.com/marianski-lab/SCR_uarrays

11. Statistical validation

The florescence intensity data for [**α-Man-FL**] or [**α-Gal-FL**], and [**SCR043**] = 500 μM were first subjected to a Grubbs' test[12] or extreme studentized deviant to determine any outliers present in the datasets. Once removed the datasets were matched for height, [glycan] and Γ([**SCR043**]). The two datasets were compared using an unpaired *t*-test[13], available on https://www.graphpad.com/quickcalcs/ttest1/

Table S123. Fluorescence intensity for [**α-Man-FL**] or [**α-Gal-FL**] at varying heights used in Grubbs' test and t-test.

**Mannose**	**Galactose**
5.89	1.14
3.62	1.30
2.56	1.41
1.87	1.36
1.53	1.25
1.26	1.22
1.22	1.18
1.20	1.00
1.56	1.27
1.40	1.32
1.53	1.27
1.52	1.46
1.47	1.46
1.41	2.20
1.22	2.47
1.23	1.41
1.86	1.35
1.57	1.29
1.56	1.28
1.48	1.35
1.50	1.21
1.37	1.30
1.21	1.30
1.20	1.21

The following results for both Mannose and Galactose showed:

Table S124. t-test of [**α-Man-FL**] or [**α-Gal-FL**].

Group	Mannose	Galactose
Mean (*I*)	1.58	1.33
Standard Deviation	0.54	0.22
Standard Error of the Mean	0.11	0.05
N	23	23

Along with a two-tailed *P* value equals 0.0414. The mean of Mannose (1.58) minus Galactose (1.33) equals 0.25. 95% confidence interval of this difference: From 0.01 to 0.47.

12. Computational Analysis

The initial conformational search has been performed using iMTD-GC algorithm implemented in CREST[14]. The search used GFN2-xTB semi-empirical energy function[15], which includes specific parametrization for noncovalent interactions, and ALPB implicit solvation model with parameters for water[16]. For the stoichiometric complexes (**SCR019:α-Gal, SCR019:β-Gal, SCR019:α-Gluc**, **SCR019:β-Gluc, SCR019:α-Man**) the search was repeated two times, starting from different, randomly generated structures. For the non-stoichiometric complexes (**SCR019$_2$:α-Gal**, **SCR019:α-Gal$_2$, SCR019$_2$:β-Gal**, **SCR019:β-Gal$_2$, SCR019$_2$:α-Gluc**, **SCR019:α-Gluc$_2$, SCR019$_2$:β-Gluc**, **SCR019:β-Gluc$_2$, SCR019$_2$:α-Man,** and **SCR019:α-Man$_2$**), the conformational search, which was repeated eight times for each complex, was initiated from either of the two most stable conformers of the respective stoichiometric complex in a presence of additional glycan or receptor. For each search, CREST saved stable minima within a 6 kcal mol^{-1} energy window above the most stable conformer. For each complex, these conformers were merged and clustered (RMSD cutoff of 1.5 Å) to remove any duplicates. Then, either all unique structures or structures within 3.5 kcal mol^{-1} above the most stable conformer (**SCR019:α-Gal$_2$, SCR019:α-Man$_2$**) were reoptimized using dispersion-corrected PBE+vdW^{TS}[17, 18] functional and *light* basis set settings of Numerical Atomic Orbitals implemented in FHI-aims[14]. The most stable structures of the **SCR019:α-Man, SCR019$_2$:α-Man,** and **SCR019:α-Man$_2$** shown in Figure 5, and rest of them are available as a part of the SI. The total number of structures is listed in Table **S131**, which adds up to 2735 unique DFT geometry optimizations. For each of these complexes, we selected 50 lowest energy structures to account for solvent effects by calculating a difference in single point energies of complexes in a gas phase and in PCM model[19] (solvent=water, UFF atomic radii) available in Gaussian16[20] (PBE0/6-31(d,p) level of theory), and we used the same set of structures to evaluate the binding energy which was calculated as single point energies at PBE0+MBD level of theory[21, 22]. This level of theory appends the hybrid PBE0 functional with dispersion energy calculated using Tkatchenko's Many-Body Dispersion scheme available in FHI-aims to provide accurate evaluation of non-pairwise dispersion contribution. These calculations were performed using a larger *intermediate* basis set settings and *LVL_fast* Resolution of Identity scheme for the two-electron

interactions. For the reference energies of **SCR019**, we performed two conformational searches, followed by same DFT level of theory. For the two glycans, we adopted chair conformation with fully extended alkyl chair and most stable H-bonding patterns and reference structures.

Table S125. Number of structures evaluated at each level of theory. For **SCR019:α-Man$_2$** and **SCR019:α-Gal$_2$**, we selected a set of most stable conformers within 3.5 kcal mol^{-1} energy window (out of the total given in parenthesis).

	**CREST**	**PBE+vdW^{TS}**	**PBE0+MBD**
**SCR019:α-Man**	306	141	50
**SCR019:α-Gal**	481	252	50
**SCR019:β-Gal**	590	305	50
**SCR019:α-Gluc**	123	77	50
**SCR019:β-Gluc**	115	72	50
**SCR019:α-Man$_2$**	2216	396/864	50
**SCR019:α-Gal$_2$**	2317	398/815	50
**SCR019:β-Gal$_2$**	417	190	50
**SCR019:α-Gluc$_2$**	272	170	50
**SCR019:β-Gluc$_2$**	219	144	50
**SCR019$_2$:α-Man**	469	144	50
**SCR019$_2$:α-Gal**	654	220	50
**SCR019$_2$:β-Gal**	113	74	50
**SCR019$_2$:α-Gluc**	643	107	50
**SCR019$_2$:β-Gluc**	1671	93	50

13. References

1. Bravo, M. F.; Palanichamy, K.; Shlain, M. A.; Schiro, F.; Naeem, Y.; Marianski, M.; Braunschweig, A. B., Synthesis and Binding of Mannose-Specific Synthetic Carbohydrate Receptors. *Chemistry – A European Journal* **2020,** *26* (51), 11782-11795.
2. Valles, D. J.; Naeem, Y.; Carbonell, C.; Wong, A. M.; Mootoo, D. R.; Braunschweig, A. B., Maskless Photochemical Printing of Multiplexed Glycan Microarrays for High-Throughput Binding Studies. *ACS Biomaterials Science & Engineering* **2019,** *5* (6), 3131-3138.
3. Carbonell, C.; Valles, D.; Wong, A. M.; Carlini, A. S.; Touve, M. A.; Korpanty, J.; Gianneschi, N. C.; Braunschweig, A. B., Polymer brush hypersurface photolithography. *Nature Communications* **2020,** *11* (1), 1244.
4. Valles, D. J.; Zholdassov, Y. S.; Braunschweig, A. B., Evolution and applications of polymer brush hypersurface photolithography. *Polymer Chemistry* **2021,** *12* (40), 5724-5746.
5. Valles, D. J.; Zholdassov, Y. S.; Korpanty, J.; Uddin, S.; Naeem, Y.; Mootoo, D. R.; Gianneschi, N. C.; Braunschweig, A. B., Glycopolymer Microarrays with Sub-Femtomolar Avidity for Glycan Binding Proteins Prepared by Grafted-To/Grafted-From Photopolymerizations. *Angewandte Chemie International Edition* **2021,** *60* (37), 20350-20357.
6. Zholdassov, Y. S.; Valles, D. J.; Uddin, S.; Korpanty, J.; Gianneschi, N. C.; Braunschweig, A. B., Orthogonal Images Concealed Within a Responsive 6-Dimensional Hypersurface. *Advanced Materials* **2021,** *33* (21), e2100803.
7. Bonda, L.; Valles, D. J.; Wigger, T. L.; Meisner, J.; Braunschweig, A. B.; Hartmann, L., TIRP–Thiol-Induced, Light-Activated Controlled Radical Polymerization. *Macromolecules* **2023,** *56* (14), 5512-5523.
8. Maeng, S.; Park, S. J.; Lee, J.; Lee, H.; Choi, J.; Kang, J. K.; Cho, H., Direct photocatalytic patterning of colloidal emissive nanomaterials. *Science Advances* **2023,** *9* (33), eadi6950.
9. Weyer, L.; Lo, S., Spectra-structure correlations in the near-infrared. *Handbook of vibrational spectroscopy* **2002,** *3*, 1817-1837.
10. Sebaugh, J. L., Guidelines for accurate EC50/IC50 estimation. *Pharmaceutical Statistics* **2011,** *10* (2), 128-34.
11. Gadagkar, S. R.; Call, G. B., Computational tools for fitting the Hill equation to dose-response curves. *Journal of Pharmacological and Toxicological Methods* **2015,** *71*, 68-76.
12. Frank, E. G., Sample Criteria for Testing Outlying Observations. *The Annals of Mathematical Statistics* **1950,** *21* (1), 27-58.
13. Mishra, P.; Singh, U.; Pandey, C. M.; Mishra, P.; Pandey, G., Application of student's t-test, analysis of variance, and covariance. *Annals of Cardiac Anaesthesia* **2019,** *22* (4), 407-411.
14. Pracht, P.; Bohle, F.; Grimme, S., Automated exploration of the low-energy chemical space with fast quantum chemical methods. *Physical Chemistry Chemical Physics* **2020,** *22* (14), 7169-7192.
15. Bannwarth, C.; Ehlert, S.; Grimme, S., GFN2-xTB-An Accurate and Broadly Parametrized Self-Consistent Tight-Binding Quantum Chemical Method with Multipole Electrostatics and Density-Dependent Dispersion Contributions. *Journal of Chemical Theory and Computation* **2019,** *15* (3), 1652-1671.
16. Ehlert, S.; Stahn, M.; Spicher, S.; Grimme, S., Robust and Efficient Implicit Solvation Model for Fast Semiempirical Methods. *Journal of Chemical Theory and Computation* **2021,** *17* (7), 4250-4261.
17. Perdew, J. P.; Burke, K.; Ernzerhof, M., Generalized Gradient Approximation Made Simple. *Physical Review Letters* **1996,** *77* (18), 3865-3868.
18. Tkatchenko, A.; Scheffler, M., Accurate molecular van der Waals interactions from ground-state electron density and free-atom reference data. *Physical Review Letters* **2009,** *102* (7), 073005.
19. Tomasi, J.; Mennucci, B.; Cammi, R., Quantum Mechanical Continuum Solvation Models. *Chemical Reviews* **2005,** *105* (8), 2999-3094.

20. Frisch, M. J.; Trucks, G. W.; Schlegel, H. B.; Scuseria, G. E.; Robb, M. A.; Cheeseman, J. R.; Scalmani, G.; Barone, V.; Petersson, G. A.; Nakatsuji, H.; Li, X.; Caricato, M.; Marenich, A. V.; Bloino, J.; Janesko, B. G.; Gomperts, R.; Mennucci, B.; Hratchian, H. P.; Ortiz, J. V.; Izmaylov, A. F.; Sonnenberg, J. L.; Williams; Ding, F.; Lipparini, F.; Egidi, F.; Goings, J.; Peng, B.; Petrone, A.; Henderson, T.; Ranasinghe, D.; Zakrzewski, V. G.; Gao, J.; Rega, N.; Zheng, G.; Liang, W.; Hada, M.; Ehara, M.; Toyota, K.; Fukuda, R.; Hasegawa, J.; Ishida, M.; Nakajima, T.; Honda, Y.; Kitao, O.; Nakai, H.; Vreven, T.; Throssell, K.; Montgomery Jr., J. A.; Peralta, J. E.; Ogliaro, F.; Bearpark, M. J.; Heyd, J. J.; Brothers, E. N.; Kudin, K. N.; Staroverov, V. N.; Keith, T. A.; Kobayashi, R.; Normand, J.; Raghavachari, K.; Rendell, A. P.; Burant, J. C.; Iyengar, S. S.; Tomasi, J.; Cossi, M.; Millam, J. M.; Klene, M.; Adamo, C.; Cammi, R.; Ochterski, J. W.; Martin, R. L.; Morokuma, K.; Farkas, O.; Foresman, J. B.; Fox, D. J. *Gaussian 16 Rev. C.01*, Wallingford, CT, 2016.

21. Perdew, J. P.; Ernzerhof, M.; Burke, K., Rationale for mixing exact exchange with density functional approximations. *The Journal of Chemical Physics* **1996,** *105* (22), 9982-9985.

22. Tkatchenko, A.; DiStasio, R. A., Jr.; Car, R.; Scheffler, M., Accurate and efficient method for many-body van der Waals interactions. *Physical Review Letters* **2012,** *108* (23), 236402.
